# Synthesis, In Vitro Antimicrobial and Cytotoxic Activities of Some New Pyrazolo[1,5-*a*]pyrimidine Derivatives

**DOI:** 10.3390/molecules24061080

**Published:** 2019-03-19

**Authors:** Ahmed M. Fouda, Hebat-Allah S. Abbas, Eman H. Ahmed, Ali A. Shati, Mohammad Y. Alfaifi, Serag Eldin I. Elbehairi

**Affiliations:** 1 Chemistry Department, Faculty of Science, King Khalid University, Abha 9004, Saudi Arabia; amfouda@hotmail.com (A.M.F.); em1433an@gmail.com (E.H.A.); 2 Photochemistry Department, National Research Centre, Dokki, Cairo 12622, Egypt; 3 Biology Department, Faculty of Science, King Khalid University, Abha 9004, Saudi Arabia; aaalshati@kku.edu.sa (A.A.S.); alfaifi@kku.edu.sa (M.Y.A.); serag@kku.edu.sa (S.E.I.E.); 4 Cell Culture Lab, Egyptian Organization for Biological Products and Vaccines (VACSERA Holding Company), 51 Wezaret El-Zeraa St., Agouza, Giza 12311, Egypt

**Keywords:** pyrazole, 3,5-diaminopyrazole, pyrazolo[1,5-*a*]pyrimidine, microwave, antimicrobial, antitumor activities

## Abstract

A new series of pyrazole **4**–**7** and pyrazolo[1,5-*a*]pyrimidine **8**–**13** were synthesized by using a simple, efficient procedure, and screened for their in-vitro antimicrobial and antitumor activities. Symmetrical and asymmetrical 3,6-diarylazo-2,5,7-triaminopyrazolo[1,5-*a*]pyrimidine were synthesized by the conventional method and also subjected to microwave irradiation and under ultrasound conditions. The biological results revealed that most of the tested compounds proved to be active as antibacterial and antifungal agents. The antitumor activity of the synthesized compounds was evaluated against human cancer cell lines, MCF-7, HCT-116, and HepG-2, as compared with Doxorubicin as a control.

## 1. Introduction

**Figure 1:**
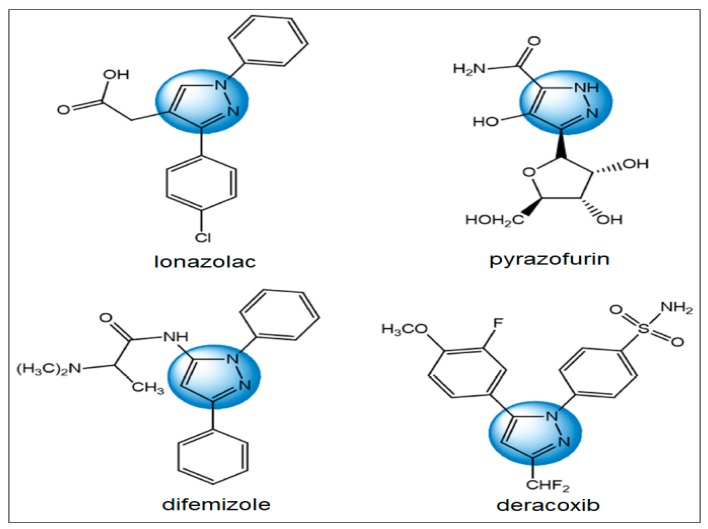
Several commercial drugs containing pyrazole ring.

Pyrazole, a heterocyclic five-membered ring system, is one of the most significant heterogeneous compounds. Pyrazole derivatives have been found to possess a broad spectrum of biological properties including antitumor activity [1,2]. Pyrazoles have attracted much attention from researchers due to their high antibacterial and antifungal values [3]. In addition, pyrazoles exhibit anti-inflammatory [4], anti-HIV [5], antiviral [6], anti-diabetic [7], and anti-tubercular activities [8]. It gained great attention since the privileged structure was commonly found as an active constituent in commercial drugs (Figure 1), such as lonazolac nonsteroidal anti-inflammatory drug (NSAID), pyrazofurin (anticancer), difenamizole (analgesic), and deracoxib (NSAID). A great deal of interest has been paid to pyrazole derivatives in the last few years in agrochemical and chemical industries [9], as some pyrazole derivatives have exhibited insecticidal [10] and herbicidal activities [11]. The great importance of pyrazole is due to its ability to construct fused pyrazole compounds that have been used as intermediates for the synthesis of dyestuffs mainly for heterocyclic azo pyrazole disperse dyes [12]. Also, it has gained considerable attention from researchers in the past few decades since it is a major component in some marketing drugs such as Ocinaplon and Lorediplon [12]. Based on the above facts, the purpose of this paper is to design and synthesize new compounds containing pyrazole moieties and study their antimicrobial activity.

## 2. Results and Discussion

### 2.1. Chemistry

**Scheme 1:**
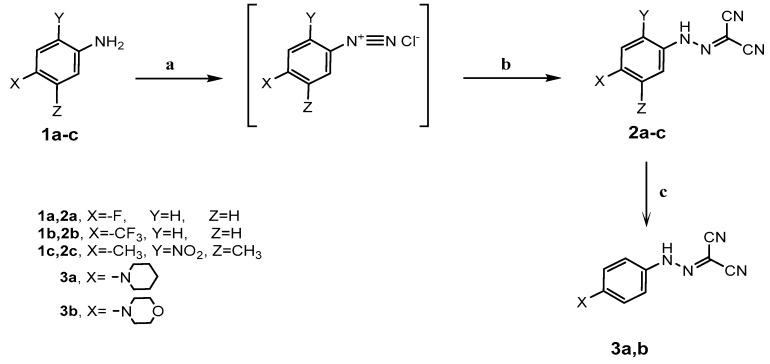
Synthetic route for 2-arylazomalononitrile derivatives **2a**–**c** and **3a**,**b**. Reagents and Conditions: (**a**) NaNO_2_/HCl/stirring at 0–5 °C 1 h; (**b**) CH_2_(CN)_2_/CH_3_COONa/50% eq. EtOH/stirring at 0–5 °C 2 h; (**c**) **2a**/piperidine or morpholine/EtOH/reflux 1 h.

2-Arylazomalononitriles **2a**–**c** were synthesized by diazotization of aniline derivatives **1a**–**c** followed by coupling with malononitrile according to the reported method [13] (Scheme 1). The structure of 2-[4-(trifluoromethyl) phenylazo]malononitrile (**2b**) was established by analytical and spectral data. The IR spectrum of (**2b**) revealed the presence of absorption bands at 3141 and 2227 cm^−1^ characteristic for NH and CN groups, respectively. The ^1^H-NMR spectrum revealed two doublets at δ 7.64 and 7.76 ppm assignable to the aromatic protons and a broad signal at δ 10.36 ppm indicating the presence of the NH_2_ group, please find more detailed data in the Supplementary Materials.

Refluxing of arylazomalononitrile **2a** with piperidine or morpholine in ethanol afforded the amino analogs **3a**,**b** (Scheme 1). The structures for **3a**,**b** were confirmed by spectral data. IR spectra of compounds **3a**,**b** showed the appearance of NH at 3301–3303 cm^−1^ and CN stretch at 2179–2184 cm^−1^. The ^1^H and ^13^C-NMR spectrum of compound **3b** revealed the presence of two characteristic signals aliphatic groups *N*-(CH_2_)_2_ and *O*-(CH_2_)_2_ of morpholine moiety.

**Scheme 2:**
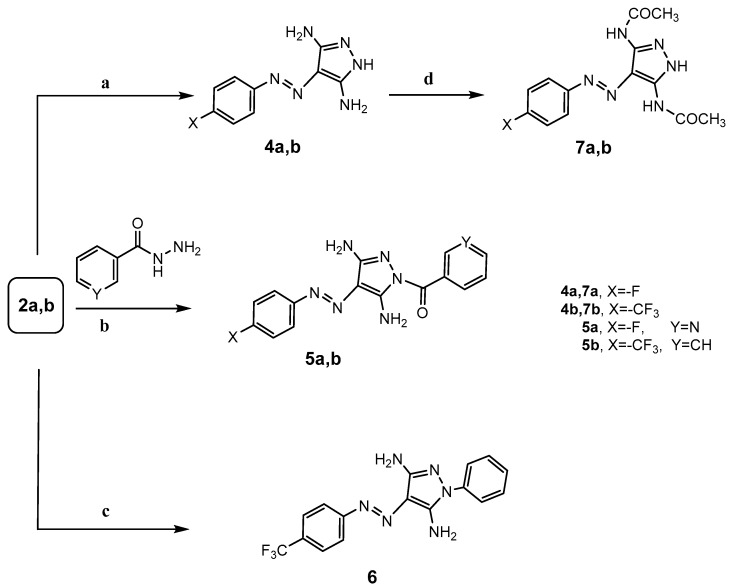
Synthetic route for 3,5-diaminopyrazole derivatives **4**–**7**. Reagents and Conditions: (**a**) N_2_H_4_·H_2_O/EtOH-pyridine/reflux 2 h; (**b**) EtOH-pyridine/reflux 15 h; (**c**) **2b**/PhNHNH_2_/EtOH-pyridine/reflux 3 h; (**d**) AcOH/reflux 10 h.

Several publications in recent years reported that various pyrazole derivatives were afforded by the reaction of arylazomalononitriles with different hydrazines [14]. The reactivity of the arylazomalononitriles **2a**,**b** with hydrazine hydrate, phenylhydrazine, and acid hydrazide derivatives were investigated. Thus, treatment of arylazomalononitriles **2a**,**b** with hydrazine hydrate or hydrazide derivatives—namely, nicotinohydrazide or benzohydrazide in ethanol—provided the corresponding 3,5-diaminopyrazole derivatives **4a**,**b** or *N*-substituted diaminopyrazole derivatives **5a**,**b**, respectively (Scheme 2). Furthermore, condensation of 2-[4-(trifluoromethyl)phenylazo]malononitrile (**2b**) with phenylhydrazine in refluxing ethanol furnished the corresponding 3,5-diamino-*N*-phenyl-4-[4-(trifluoromethyl)phenylazo]-1*H*-pyrazole (**6**) (Scheme 2). Spectral data confirmed the assignment of 3,5-diamino-4-[4-(trifluoromethyl)phenylazo]-1*H*-pyrazole (**4b**). The IR spectrum shows the absence of the characteristic absorption band of CN and the appearance of absorption bands at 3465, 3389, and 3296 corresponding to NH_2_ groups. Its ^1^H-NMR revealed the presence of NH_2_ signal at δ 6.29 ppm, a pair of doublets at δ 7.71 and 7.83 ppm corresponding to aromatic protons, and NH signal at δ 10.87 ppm. Also, the mass spectrum showed a molecular ion peak at *m*/*z* 270 (M^+^, 100%) which confirmed its molecular formula C_10_H_9_F_3_N_6_.

Acetylation of 3,5-diamino-1*H*-pyrazole derivatives **4a**,**b** in refluxing acetic acid afforded the corresponding 4-arylazo-3,5-diacetamidopyrazole derivatives **7a**,**b**; (Scheme 2). The structures of 1*H*-pyrazole derivatives **7a**,**b** were supported by elemental and spectral data. The ^1^H-NMR spectrum for compound **7a** exhibited two singlet signals at δ 2.12 and 2.17 ppm corresponding to the presence of methyl protons and two broad signals at δ 6.56 and 10.27 ppm related to two (NH) protons, while its mass spectrum revealed a molecular ion peak at *m*/*z* 304 (M^+^, 100%).

**Scheme 3:**
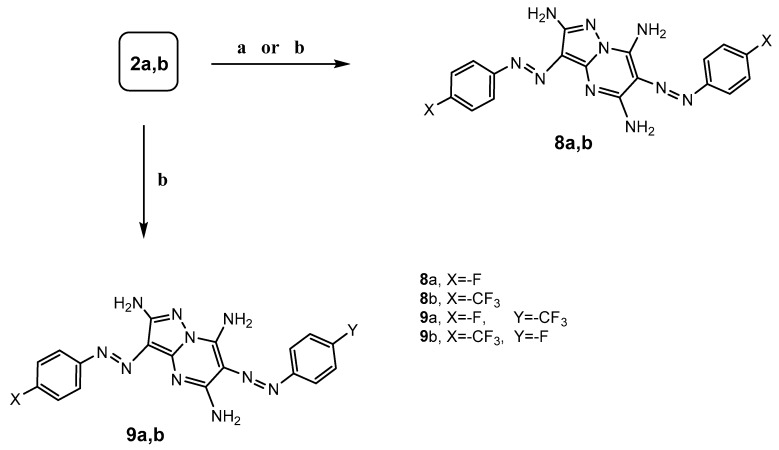
Synthetic route for symmetrical and asymmetrical 3,6-diarylazo-pyrazolo[1,5-*a*] pyrimidine derivatives **8a**,**b** and **9a**,**b**. Reagents and Conditions: (**a**) 2-arylazomalononitrile **2a**,**b**: N_2_H_4_·H_2_O (2:1)/EtOH-pyridine/reflux 5 h; (**b**) 2-arylazomalononitrile **2a**,**b**: 5-diaminopyrazoles **4a**,**b** (1:1)/EtOH-pyridine/reflux 4–7 h or heated under MW at 140 °C 2 min or sonicated at room temperature 1 h.

3,5-Diaminopyrazoles **4** was employed as a key intermediate for the synthesis of several poly substituted fused pyrazolopyrimidines. Pyrazolo[1,5-*a*]pyrimidines are of considerable pharmacological important as purine analogs [15,16,17]. Thus, it was important to study the reactivity of diaminopyrazole derivatives **4a**,**b** toward a variety of nucleophilic reagents. Symmetrical 3,6-diarylazo-2,5,7-triaminopyrazolo[1,5-*a*]pyrimidine (**8a**,**b**) were synthesized by the reaction of hydrazone **2a**,**b** [13,18] with hydrazine hydrate in the molar ratio 2:1 in ethanol containing 0.5 mL of pyridine under reflux for 4–5 h, or via the reaction of 3,5-diaminopyrazole **4a**,**b** with the equivalent from the hydrazone derivatives **2a**,**b** (Scheme 3). Asymmetrical 2,5,7-triaminopyrazolo[1,5-*a*]pyrimidine **9a**,**b** were synthesized by the cyclization of diaminopyrazole **4a**,**b** with equimolar of different hydrazone derivatives **2a**,**b** in ethanol containing 0.5 mL of pyridine under reflux for 6–7 h (Scheme 3).

Moreover, the synthesis of pyrazolopyrimidine derivatives **8a**,**b** and **9a**,**b** were achieved under microwave irradiation and ultrasound reactions conditions. TLC monitored the progress of the reactions, and the yields of the products were noted. Comparisons between conventional, microwave, and ultrasound techniques are given in (Table 1).

The analytical and spectral data of the newly synthesized compounds **8a**,**b** and **9a**,**b** were compatible with their structures. For instance, IR spectrum for 6-[(4-trifluoromethyl)phenylazo]-3-(4-fluorophenylazo)-2,5,7-triaminopyrazolo[1,5-*a*] pyrimidine (**9a**) showed absorption bands at 3474, 3276, and 3123 cm^−1^, representing the presence of the amino groups, while ^1^H-NMR spectrum showed signals at δ 6.98 and 8.22 ppm referring to two amino groups, and its mass spectrum showed a molecular ion peak at *m*/*z* 458 (M^+^, 100%), which corresponds with its molecular formula C_19_H_14_F_4_N_10_.

**Scheme 4:**
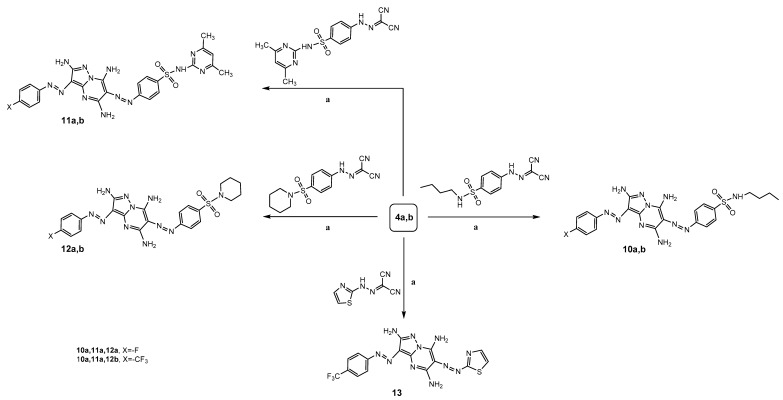
Synthetic route for 2,5,7-triaminopyrazolo[1,5-*a*]pyrimidine derivatives **10**–**13**. Reagents and Conditions: (**a**) EtOH-pyridine/reflux 7–12 h or heated under MW at 140 °C 3–8 min.

Sulfonamide group can be considered as the main component in some drugs used in clinical treatment. It is reported that many of the sulfonamide derivatives act as antibacterial, antifungal, antihypertensive, anticancer, antimalarial, and antiprotozoal agents [13]. Thus, the reaction of 3,5-diaminopyrazole **4a**,**b** with several hydrazones of sulfa drugs (*N*-[4-(*N*-butylsulfamoyl)phenyl]carbonohydrazonoyl dicyanide, *N*-{4-[*N*-(4,6-dimethylpyrimidin-2-yl)sulfamoyl]phenyl}carbono-hydrazonoyl dicyanide, and *N*-[4-(piperidin-1-ylsulfonyl)phenyl]carbono-hydrazonoyl dicyanide) in ethanol under reflux in the presence of a catalytic amount of pyridine afforded pyrazolo[1,5-*a*]pyrimidine derivatives **10a**,**b**–**12a**,**b**, respectively (Scheme 4). Aiming to modify the yields and reduce the reactions time, we applied microwave irradiation conditions to resynthesize the targeted compounds **10a**,**b**–**12a**,**b**, and the comparison of the reactions times and yields between the conventional and microwave irradiation conditions are summarized in Table 2.

The structures of **10a**,**b**–**12a**,**b** were confirmed by their elemental analysis and spectral data (MS, IR, ^1^H-NMR, and ^13^C-NMR). The IR spectrum revealed an absorption band at 3474–3207 cm^−1^ that corresponds to NH and NH_2_ groups and at 2960–2835 and 1369–1347 cm^−1^ characteristic to aliphatic CH_2_ and SO_2_ groups, respectively. ^1^H-NMR spectrum of (**12b**), as an example, revealed signals at δ 1.36, 1.55, and 2.91 which refer to the presence of (3CH_2_) and (2NCH_2_) groups, respectively, while its ^13^C-NMR spectrum exhibited signals representing the presence of piperidine protons at δ 22.85, 24.66, and 46.58. Mass spectrum of (**12b**), as an example, gives the molecular ion peak at *m*/*z* 587 (M^+^; 44%), corresponding to the molecular formula C_24_H_24_FN_11_O_2_S.

Also, 6-(thiazol-2-yl-azo)-3-(4-(trifluoromethyl)phenylazo)-2,5,7-triamino pyrazolo [1,5-*a*]pyrimidine (**13**) was synthesized via the treatment of 3,5-diamine-4-[4-(trifluoromethyl)phenylazo]-1*H*-pyrazole (**4b**) with *N*-(thiazol-2-yl) carbonohydrazonoyl dicyanide in ethanol under reflux or microwave irradiation (Scheme 4).

^1^H-NMR spectrum of **13** showed singlet signals at δ 7.47 and 7.76 ppm due to the presence of two protons of thiazole ring, as well as signals at δ 7.11 and 8.24 ppm for amino groups. The mass spectrum revealed a molecular ion peak at 447 (M^+^; 100%), which confirms its molecular formula C_16_H_12_F_3_N_11_S.

### 2.2. Biological Evaluation

#### 2.2.1. In Vitro Antimicrobial Evaluation

The newly synthesized compounds were evaluated for their antimicrobial activity toward six bacterial strains, three Gram-positive (*Staphylococcus aureus*, *Bacillus subtilis*, and *Staphylococcus mutants*), and three Gram-negative (*Enterococcus faecalis*, *Proteus vulgaris*, and *Escherichia coli*), using the standard antibiotic Gentamycin (5 mg/mL) as a reference. Also, their antifungal activity was evaluated against three fungal strains (*Aspergillus fumigates*, *Aspergillus flavus*, and *Candida albicans*) to determine the zone of inhibition using the standard antibiotic Ketoconazole (5 mg/mL). Their antibacterial activities were tested for their activities at a concentration of (5 mg/mL) using inhibition zone diameter in mm as a criterion for the antimicrobial activity [19,20], and the results are shown in (Table 3).

Compounds **2a**, **2c**, and **11b** demonstrated more potent significant activity against tested *Enterococcus faecalis* and *Proteus vulgaris* with a diameter of inhibition zone between 25 and 40 mm; further, compound **2a** showed high antimicrobial activity on *Bacillus subtilis* with a zone of inhibition of 40 mm, and these compounds had moderate antimicrobial activity on other bacterial strains with an inhibition zone ranging from 13 to 25 mm. Moreover, compound **2a** has significant antifungal activity towards three fungal strains, and compound **11b** showed a moderate effect on fungal strains, while compound **2c** was not active on fungal strains. Also, compound **4b** had a moderate effect on bacterial strains with an inhibition zone of 20–24 mm diameter, as well as on fungal strains with an inhibitory region ranging from 13 to 20 mm.

Furthermore, compounds **5a**, **7a**, **7b**, and **11a** showed mild antibacterial activity on the tested Gram-positive and Gram-negative bacterial strains with an inhibition zone of 9 to 18 mm, except compound **5a** was NA on *Streptococcus mutants* strain. Meanwhile, compounds **5a**, **7b**, and **11a** had no antifungal activity towards the three fungal strains.

Compounds **8a**, **8b**, **9a**, **9b**, **10a**, **10b**, **12a**, **12b**, and **13** were NA on all evaluated bacterial and fungal strains, except for compounds **8a**, **8b**, and **9b**, which exhibited low antibacterial activity on *Escherichia coli* (10 mm), *Bacillus subtilis* (8 mm), and *Staphylococcus aureus* (10 mm), respectively.

#### 2.2.2. In Vitro Cytotoxic Screening

**Figure 2:**
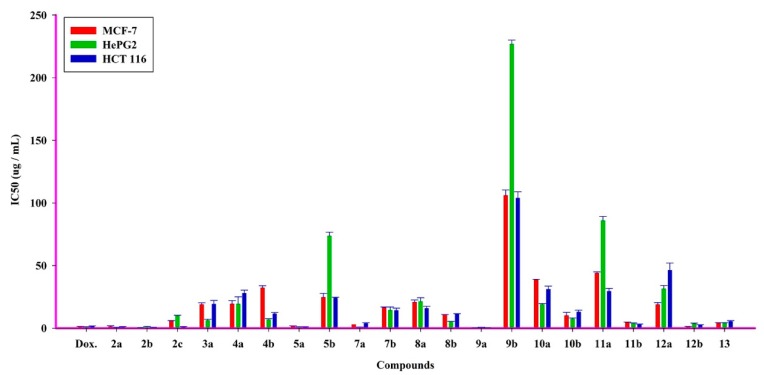
The differences of IC_50_ % of new tested chemicals compounds effects on different tumor cells (MCF-7, HePG2, and HCT 116 cells).

The antitumor activity of the target compounds was screened against three human cancer cell lines—breast adenocarcinoma (MCF-7), hepatocellular carcinoma (HepG2), and human colon carcinoma (HCT-116). The IC_50_ values of the tested compounds are listed in Table 4 and Figure 2.

From the obtained results, compounds **2a**,**b**, **4b**, **5b**, **8b**, and **12b** showed the most potent cytotoxic profile on cancer cells (MCF-7, HepG2, and HCT-116) with IC_50_ values ranging from 0.3 to 3.4 µg while the compound **8b** is most effective among all of them on all types of cancer cells.

Compounds **2c**, **11b**, and **13** have demonstrated a significant effect on MCF-7 and HePG2 cells with IC_50_ values ranging from 2.7 to 9.8 µg, and **2c** and **11b** compounds significantly increased on HCT 116 with IC_50_ of 1.2 μg and 2.4, respectively. Moreover, the compounds **4a**, **8a**, and **10a** showed moderate effects on cancer cells with IC_50_ values ranging from 6.7 to 12.9 µg, while the compound **8a** was the most significant on HePG2 cells with IC_50_ of 4.9 µg; on the other hand, the effect of the **4a** compound was low on MCF-7 cells. In addition, the compounds **3a**,**b**, **5a**, **7a**,**b**, **9b**, **11a**, and **12a** had slightly significant effects with IC_50_ values ranging from 14.1 to 38.5 µg, the compound **3a** indicated a slightly noticeable effect on HePG2 cells with IC_50_ of 6.1 µg, and the compound **12a** had weak toxicity on HCT 116 cells with IC_50_ of 46.1 µg. Furthermore, the **10b** compound had a low cytotoxic effect on MCF-7 and HePG2 cells with IC_50_ of 43.9 and 85.9 µg, respectively, and a moderate effect with IC_50_ of 29.4 µg. Compound **9a** showed very low cellular toxicity with IC_50_ higher than 100 μg compared to all other compounds on cancer cells.

## 3. Materials and Methods

### 3.1. General Information

All melting points were determined with a Stuart Digital Melting Point apparatus SMP10 and are uncorrected. Elemental analyses were performed on a Perkin-Elmer 240 microanalyser, PE 2400 Series II CHNS/O Analyzer, carried out at the regional center for mycology and biotechnology, Al-Azhar University, cairo, Egypt. IR spectra were determined as KBr pellets on a Thermo Nicolet apparatus (Thermo Scientific, Madison, WI, USA) at Postgraduate campus for Girls at Lassan, King Khalid University, Abha, Saudi Arabia. The NMR spectra were recorded on a Bruker NMR spectrometer (Bruker, Billerica, MA, USA) in DMSO-*d*_6_, as solvent at 300, 500 MHz for ^1^H-NMR and 75, 125 MHz for ^13^C-NMR at Faculty of Science, Cairo University, Egypt and King Khalid University, Abha, respectively. The chemical shifts (δ) are reported in parts per million (ppm). Mass spectra were measured on GC/MS-QP5 spectrometer at regional center for mycology and biotechnology, Al-Azhar University, Egypt. Antimicrobial activity was measured at the regional center for mycology and biotechnology, Al-Azhar University, Egypt. Follow-up of the reactions and checking the purity of the compounds were carried out using TLC on silica gel-precoated aluminum sheets (Fluorescent indicator 254 nm, Fluka, Germany) and the spots were detected by exposure to UV lamp at λ 254/366 nm for a few seconds or under iodine vapor. Compounds 2-(4-fluorphenylazo)malononitrile **2a** and 3,5-diamino-4-(4-fluorophenylazo)-1*H*-pyrazole **4b** were prepared according to the reported procedure [21,22,23].

#### 3.1.1. General Procedure for the Synthesis of 2-Arylazomalononitrile **2a–c**:

To a solution of aniline derivatives **1a–c** (0.01 mol) in hydrochloric acid (6 mL), a solution of sodium nitrite (0.72 g, 0.0105 mol) in water (3 mL) was added portion wise with stirring at 0–5 °C for 1 h. The clear diazonium salt was added to a stirred solution of malononitrile (0.66 g, 0.01 mol) and sodium acetate (4.6 g) in aqueous ethanol 50% (50 mL) with continued stirring at 0–5 °C for 2 h. The reaction mixture was allowed to stand overnight at room temperature, then the precipitate that formed was filtered off, washed several times with water, dried, and recrystallized from ethanol to afford 2-arylazomalononitrile **2a–c**.

*2-[4-(Trifluoromethyl)phenylazo]malononitrile (***2b***)*: Yield: 93%; (orange powder): mp 179–180 °C; IR (KBr) ν_max_ in cm^−1^: 3141 (NH), 2227 (C≡N), 1618 (C=N) ^1^H-NMR (500 MHz, DMSO-*d*_6_): δ 7.64 (d, 2H, Ar–H, *J* = 6.9 Hz), 7.76 (d, 2H, Ar–H, *J* = 7.1 Hz), 10.36 (s, br, 1H, NH); ^13^C-NMR (125 MHz, DMSO-*d*_6_): δ 86.98 (*C*(CN)_2_), 109.52 (C≡N), 113.88, 116.69 (Ar–C), 123.03, 125.19 (q, C–F_3_, *J* = 270 Hz), 126.72, 127.35, 144.52 (Ar–C); MS (*m*/*z*), 239 (M^+^ + 1; 6%), 238 (M^+^; 10%), 94 (95%), 67 (100%). Anal. Calcd. for C_10_H_5_F_3_N_4_ (238.17): C, 50.43; H, 2.12; N, 23.52%. Found C, 50.47; H, 2.34; N, 23.64%.

*2-(4,5-Dimethyl-2-nitrophenylazo)malononitrile (***2c***)*: Yield: 45%; (yellow powder): mp 160–162 °C; IR (KBr) ν_max_ in cm^−1^: 3231 (NH), 2989, 2954 (C-H aliphatic), 2215 (C≡N), 1617 (C=N); ^1^H-NMR (500 MHz, DMSO-*d*_6_): δ 2.30, 2.36 (2s, 6H, 2CH_3_), 7.60 (s, 1H, Ar–H), 7.98 (s, 1H, Ar–H), 9.88 (s, br, 1H, NH); ^13^C-NMR (125 MHz, DMSO-*d*_6_): δ 18.52, 19.69 (CH_3_), 89.05 (*C*(CN)_2_), 109.05 (C≡N), 113.34, 118.97, 125.61, 134.62, 135.11, 146.70 (Ar–C); MS (*m*/*z*), 244 (M^+^ + 1; 11%), 243 (M^+^; 25%), 61 (100%). Anal. Calcd. for C_11_H_9_N_5_O_2_ (243.23): C, 54.32; H, 3.73; N, 28.79%. Found C, 54.19; H, 3.59; N, 28.86%.

#### 3.1.2. General Procedures for the Synthesis of 2-[(4-Substituted)arylazo] malononitrile **3a**,**b**:

A mixture of 2-arylazomalononitrile **2a** (0.01 mol) and piperidine or morpholine (0.01 mol) in ethanol (30 mL) was refluxed for 1 h and then left to cool to room temperature. The reaction mixture was poured onto cooled water with continuous stirring. The precipitate that formed was filtered, dried, and recrystallized from toluene to afford 2-[(4-substituted)arylazo] malononitrile **3a**,**b**.

*2-[4-(Piperidino)phenylazo]malononitrile (***3a***):* Yield: 81%; (yellow plates): mp 197–199 °C; IR (KBr) ν_max_ in cm^−1^: 3301 (NH), 2942, 2856 (C-H aliphatic), 2184 (C≡N), 1621 (C=N); ^1^H-NMR (300 MHz, DMSO-*d*_6_): δ 1.64 (m, 6H, 3CH_2_), 3.55 (m, 4H, 2N–CH_2_), 7.13 (d, 2H, Ar–H, *J* = 6.9 Hz), 7.50 (m, 3H, 2H Ar–H + NH); ^13^C-NMR (75 MHz, DMSO-*d*_6_): δ 23.61, 25.62 (CH_2_), 49.31 (N–CH_2_), 92.58 (*C*(CN)_2_), 115.12, 115.41 (C≡N), 116.95, 121.75, 121.86 (Ar–C); MS (*m*/*z*), 253 (M^+^; 7%), 242 (88%), 72 (100%). Anal. Calcd. for C_14_H_15_N_5_ (253.31): C, 66.38; H, 5.97; N, 27.65%. Found C, 66.33; H, 5.80; N, 27.54%.

*2-(4-Morpholinophenylazo)malononitrile (***3b***):* Yield: 81%; (yellow crystals): mp 166–167 °C; IR (KBr) ν_max_ in cm^−1^: 3303 (NH), 2934, 2861 (C–H aliphatic), 2179 (C≡N), 1618 (C=N); ^1^H-NMR (300 MHz, DMSO-*d*_6_): δ 3.60 (t, 4H, 2N–CH_2_), 3.70 (t, 4H, 2O–CH_2_), 7.15 (d, 2H, 2 Ar–H, *J* = 4.8 Hz), 7.51(d, 2H, 2 Ar–H, *J* = 5.4 Hz), 7.69 (s, 1H, NH); ^13^C-NMR (75 MHz, DMSO-*d*_6_): δ 48.75 (N–CH_2_), 65.99 (O–CH_2_), 92.64 (*C*(CN)_2_), 115.19, 115.48 (C≡N), 116.87, 121.91, 122.02 (Ar–C); MS (*m*/*z*), 255 (M^+^; 21%), 111 (56%), 43 (100%). Anal. Calcd. for C_13_H_13_N_5_O (255.28): C, 61.17; H, 5.13; N, 27.43%. Found C, 61.28; H, 5.22; N, 27.48%.

#### 3.1.3. General Procedure for the Synthesis of 4-Arylazo-3,5-diaminopyrazole **4a**,**b**

To a solution of 2-arylazomalononitrile **2a**,**b** (0.01 mol) in ethanol (30 mL), hydrazine hydrate (0.01 mol) and pyridine (0.5 mL) were added. The reaction mixture was heated under reflux for 2 h (monitored by TLC). After completion of the reaction, the precipitate that formed was filtered off, dried, and recrystallized from ethanol to afford 3,5-diamino-4-(arylazo)pyrazole **4a**,**b**.

*3,5-Diamino-4-[4-(trifluoromethyl)phenylazo]-1H-pyrazole (***4b***):* Yield: 98%; (orange crystals): mp 231–232 °C; IR (KBr) ν_max_ in cm^−1^: 3465, 3389, 3296 (NH_2_,NH), 1622 (C=N), 1421 (N=N); ^1^H-NMR (500 MHz, DMSO-*d*_6_): δ 6.29 (br, 4H, 2NH_2_), 7.71 (d, 2H, Ar–H, *J* = 8.5 Hz), 7.83 (d, 2H, Ar–H, *J* = 8.0 Hz), 10.87 (br, 1H, NH); ^13^C-NMR (125 MHz, DMSO-*d*_6_): δ 115.61, 120.62, 120.94 (Ar–C), 123.47, 125.63 (q, C–F_3_, *J* = 270 Hz), 125.80, 125.83, 127.79 (Ar–C), 156.37 (C=N); MS (*m*/*z*), 271 (M^+^ + 1; 14%), 270 (M^+^; 100%). Anal. Calcd. for C_10_H_9_F_3_N_6_ (270.22): C, 44.45; H, 3.36; N, 31.10%. Found C, 44.39; H, 3.23; N, 31.19%.

#### 3.1.4. General Procedure for the Synthesis of 4-Arylazo-3,5-diamino-*N*-substitutedpyrazole **5a**,**b**

To a solution of 2-arylazomalononitrile **2a**,**b** (0.01 mol) in ethanol (30 mL), hydrazide derivatives, namely nicotinohydrazide or benzohydrazide (0.01 mol) and pyridine (0.5 mL), were added. The reaction mixture was heated under reflux for 15 h (monitored by TLC). After completion of the reaction, the precipitate that formed was filtered off, dried, and recrystallized from ethanol to afford *N*-substituted-3,5-diamino-4-(arylazo)pyrazole **5a**,**b**.

*{3,5-Diamino-4-[(4-fluorophenyl)azo]-1H-pyrazol-1-yl}(pyridin-3-yl)methanone (***5a***):* Yield: 69%; (orange plates): mp >3 00 °C; IR (KBr) ν_max_ in cm^−1^: 3477, 3274, 3127 (NH_2_), 1672 (C=O), 1593 (C=N), 1423 (N=N); ^1^H-NMR (500 MHz, DMSO-*d*_6_): δ 6.92 (s, 2H, NH_2_), 7.28–7.32 (m, 4H, Ar–H), 7.72–7.75 (m, 2H, Ar–H), 7.95–7.98 (m, 2H, Ar–H), 8.08 (br, s, 2H, NH_2_); ^13^C-NMR (125 MHz, DMSO-*d*_6_): δ 107.82, 112.32, 120.54, 121.24, 125.82, 125.89, 126.92, 127.16, 128.35, 129.02, 131.04 (Ar–C), 153.15, 157.93 (C=N), 160.52 (C–F), 166.38 (C=O); MS (*m*/*z*), 327 (M^+^ + 2; 3.3%), 325 (M^+^; 12%), 95 (100%). Anal. Calcd. for C_15_H_12_FN_7_O (325.31): C, 55.38; H, 3.72; N, 30.14%. Found C, 55.28; H, 3.68; N, 30.24%.

*{3,5-Diamino-4-[(4-(trifluoromethyl)phenylazo]-1H-pyrazol-1-yl}(phenyl)methanone (***5b***):* Yield: 71%; (orange powder): mp 284–286 °C; IR (KBr) ν_max_ in cm^−1^: 3506, 3353, (NH_2_), 1689 (C=O), 1618 (C=N), 1411 (N=N); ^1^H-NMR (500 MHz, DMSO-*d*_6_): δ 7.02 (s, 2H, NH_2_), 7.67–7.73 (m, 4H, Ar–H), 7.77–7.86 (m, 7H, Ar–H+NH_2_); ^13^C-NMR (125 MHz, DMSO-*d*_6_): δ 107.46, 121.02, 121.16, 121.65 (Ar–C), 123.63, 125.81 (q, C–F_3_, *J* = 272.5 Hz), 126.15, 127.02, 127.68, 149.08 (Ar–C), 150.11 (C=N), 167.31 (C=O); MS (*m*/*z*), 375 (M^+^ + 1; 5%), 374 (M^+^; 12%), 145 (100%). Anal. Calcd. for C_17_H_13_F_3_N_6_O (374.33): C, 54.55; H, 3.50; N, 22.45%. Found C, 54.75; H, 3.58; N, 22.35%.

#### 3.1.5. Synthesis of 3,5-Diamino-*N*-phenyl-4-[4-(trifluoro-methyl)phenylazo]-1*H*-pyrazole (**6**)

Phenylhydrazine (0.98 mL, 0.01 mol) and pyridine (0.5 mL) were added to a solution of 2-arylazomalononitrile **2b** (2.7 g, 0.01 mol) in ethanol (30 mL). The reaction mixture was heated under reflux for 3 h (monitored by TLC). After completion of the reaction, the precipitate that formed was filtered off, dried, and recrystallized from ethanol to give 3,5-diamino-1-phenyl-4-[4-(trifluoro-methyl)phenylazo]-1*H*-pyrazole **6**. Yield: 71%; (yellow crystals): mp >300 °C; IR (KBr) ν_max_ in cm^−1^: 3400, 3304, 3197 (NH_2_, NH), 1616 (C=N), 1423 (N=N); ^1^H-NMR (500 MHz, DMSO-*d*_6_): δ 6.99 (br, s, 2H, NH_2_), 7.33 (d, 2H, Ar–H, *J* = 7.5 Hz), 7.50 (d, 2H, Ar–H, *J* = 8.0 Hz), 7.53–7.76 (m, 5H, Ar–H), 7.95 (br, s, 2H, NH_2_); ^13^C-NMR (125 MHz, DMSO-*d*_6_): δ 101.00, 121.07, 121.26, 122.26 (Ar–C), 123.42, 125.59, 125.95 (q, C–F_3_, *J* = 270 Hz), 126.16, 126.41, 129.27, 149.07 (Ar–C), 156.07 (C=N). Anal. Calcd. for C_16_H_13_F_3_N_6_ (346.32): C, 55.49; H, 3.78; N, 24.27%. Found C, 55.68; H, 3.91; N, 24.22%.

#### 3.1.6. Synthesis of 4-Arylazo-3,5-diacetamido-1*H*-pyrazole **7a**,**b**

A solution of 3,5-diamine-4-arylazo-1*H*-pyrazole **4a**,**b** (0.01 mol) in acetic acid (30 mL) was heated under reflux for 10 h (monitored by TLC). The reaction mixture was left to cool to room temperature, and then poured onto cooled water (50 mL) portion wise with continuous stirring for 1 h. The separated yellow compound was filtered off, washed with water, and, finally, dried and recrystallized from ethanol to afford 3,5-diacetamio-4-arylazo-1*H*-pyrazole **7a**,**b**.

*3,5-Diacetamido-4-[(4-fluorophenyl)azo]-1H-pyrazole (***7a***):* Yield: 86%; (yellow powder): mp 234–236 °C; IR (KBr) ν_max_ in cm^−1^: 3277, 3144 (NH), 1698 (C=O), 1605 (C=N), 1418 (N=N); ^1^H-NMR (300 MHz, DMSO-*d*_6_): δ 2.12, 2.17 (2s, 6H, 2CH_3_), 6.56 (br, 1H, NH), 7.77–7.92 (m, 5H, Ar–H+NH), 10.27 (br, 1H, NH); ^13^C-NMR (75 MHz, DMSO-*d*_6_): δ 23.12 (CH_3_), 115.82, 116.08, 120.56, 122.74, 122.85, 123.68, 123.80 (Ar–C), 149.12 (C=N), 161.04 (C–F), 169.24 (2C=O): MS (*m*/*z*), 305 (M^+^ + 1; 28%), 304 (M^+^; 100%), 42 (76%). Anal. Calcd. for C_13_H_13_FN_6_O_2_ (304.29): C, 51.31; H, 4.31; N, 27.62%. Found C, 51.38; H, 4.41; N, 27.53%.

*3,5-Diacetamido-4-[4-(trifluoromethyl)phenylazo]-1H-pyrazole (***7b***):* Yield: 80%; (Yellow plates): mp 236–238 °C; IR (KBr) ν_max_ in cm^−1^: 3238 (NH), 1678 (C=O), 1595 (C=N), 1426 (N=N); ^1^H-NMR (300 MHz, DMSO-*d*_6_): δ 2.24 (s, 6H, 2CH_3_), 7.82–7.96 (m, 5H, Ar–H+NH), 8.40, 10.11 (2br, s, 2H, 2NH); ^13^C-NMR (125 MHz, DMSO-*d*_6_): δ 23.38 (CH_3_), 100.33, (Ar–C), 123.10, 125.26 (q, C–F_3_, *J* = 270 Hz), 126.07, 126.24, 126.26, 128.75, 129.00 (Ar–C), 155.61 (C=N), 167.33 (C=O): MS (*m*/*z*), 354 (M^+^; 19%), 312 (72%), 70 (100%). Anal. Calcd. for C_14_H_13_F_3_N_6_O_2_ (354.29): C, 47.46; H, 3.70; N, 23.72%. Found C, 47.57; H, 3.79; N, 23.76%.

#### 3.1.7. General Procedure for the Synthesis of 3,6-Diarylazo-2,5,7-triaminopyrazolo[1,5-*a*]pyrimidine derivatives **8a**,**b**

##### A. Under reflux Condition:

Method A: A mixture of 2-arylazomalononitrile **2a**,**b** (0.02 mol) and hydrazine hydrate (0.01 mol) in ethanol (30 mL) in the presence of pyridine (0.5 mL) was heated under reflux for 5 h, (monitored by TLC). After completion of the reaction, the precipitates those formed were filtered off, dried, and recrystallized from 1,4-dioxane to afford **8a**,**b**.

Method B: A mixture of 4-arylazo-3,5-diaminopyrazole **4a**,**b** (0.01 mol) and 2-arylazomalononitrile **2a**,**b** (0.01 mol) in ethanol (30 mL) and in the presence of pyridine (0.5 mL) was heated under reflux for 4–5 h, (monitored by TLC). After completion of the reaction, the precipitates those formed were filtered off, dried, and recrystallized from 1,4-dioxane to afford **8a** and **8b**, respectively.

##### B. Under Microwave Irradiation:

A mixture of 5-diamino-4-arylazopyrazole **4a**,**b** (0.01 mol) and 2-arylazomalononitrile **2a**,**b** (0.01 mol) was grinded carefully in a porcelain mortar using a pestle and transferred to a pyrex test tube; then, ethanol (4 mL) was added, followed by pyridine (0.5 mL). The reaction mixture was heated under microwave irradiation at 50% power for 2 min at 140 °C, monitored by TLC. After completion of the reaction, the reaction mixture was cooled to room temperature and the precipitated solid was filtered off, washed with MeOH, and recrystallized from dioxane to afford **8a** and **8b**, respectively.

##### C-Under Ultrasound Condition:

To a solution of 4-arylazo-3,5-diaminopyrazole **4a**,**b** (0.01 mol) in ethanol (30 mL), 2-arylazomalononitrile **2a**,**b** (0.01 mol) and pyridine (0.5 mL) was added. The reaction mixture was sonicated for 1 h at room temperature, (monitored by TLC). After completion of the reaction, the precipitate product was filtered off, dried, and recrystallized from dioxane to afford **8a** and **8b** respectively.

*3,6-Bis[(4-fluorophenyl)azo]-2,5,7-triaminopyrazolo[1,5-a]pyrimidine (***8a***):* Yield: 74%; (orange plates): mp >300 °C; IR (KBr) ν_max_ in cm^−1^: 3422, 3274 (NH2), 1615 (C=N), 1420 (N=N); ^1^H-NMR (500 MHz, DMSO-d_6_): δ 6.92 (s, 2H, NH_2_), 7.29–7.32 (m, 4H, Ar–H), 7.72–7.75 (m, 4H, Ar–H), 7.96 (s, br, 2H, NH_2_), 8.10 (s, 2H, NH_2_); ^13^C-NMR (125 MHz, DMSO-d_6_): δ 107.81, 115.11, 115.66, 115.70, 115.84, 115.86, 122.34, 122.40, 123.72, 123.79, 147.22, 149.08, 149.10 (Ar–C), 150.05, 152.31 (C=N), 160.52, 161.18 (C–F); MS (*m*/*z*), 409 (M^+^ + 1; 30%), 408 (M^+^; 100%), 363 (54%), Anal. Calcd. for C_18_H_14_F_2_N_10_ (408.38): C, 52.94; H, 3.46; N, 34.30%. Found C, 52.83; H, 3.52; N, 34.22%.

*3,6-Bis[4-(trifluoromethyl)phenylazo]-2,5,7-triaminopyrazolo[1,5-a]pyrimidine (***8b***):* Yield: 71%; (orange crystals): mp >300 °C; IR (KBr) ν_max_ in cm^−1^: 3460, 3259, 3103 (NH_2_), 1602 (C=N), 1417 (N=N); ^1^H-NMR (500 MHz, DMSO-*d*_6_): δ 7.06 (s, 2H, NH_2_; exchangeable with D_2_O), 7.77–7.86 (m, 10H, Ar–H+NH_2_; exchangeable with D_2_O), 8.19 (s, 2H, NH_2_; exchangeable with D_2_O); ^13^C-NMR (125 MHz, DMSO-*d*_6_): δ 108.98, 116.57, 121.82, 122.25 (Ar–C), 123.19, 125.35 (q, C–F_3_, *J* = 287.5 Hz), 125.49, 125.99, 126.02, 126.17, 126.20, 126.83, 127.08, 127.86, 128.11, 148.05 (Ar–C), 152.38, 154.71 (C=N); MS (*m*/*z*), 509 (M^+^ + 1; 25%), 508 (M^+^; 100%). Anal. Calcd. for C_20_H_14_F_6_N_10_ (508.39): C, 47.25; H, 2.78; F, 22.42; N, 27.55%. Found C, 47.34; H, 2.71; N, 27.44%.

#### 3.1.8. General Procedure for the Synthesis of 3,6-Diarylazo-2,5,7-triaminopyrazolo[1,5-*a*]pyrimidine Derivatives **9a**,**b**

##### A. Under Reflux Condition:

To a solution of 4-arylazo-3,5-diaminopyrazole **4a**,**b** (0.01 mol) in ethanol (30 mL), 2-arylazomalononitrile **2a**,**b** (0.01 mol) and pyridine (0.5 mL) were added. The reaction mixture was heated under reflux for 6–7 h (monitored by TLC). After completion of the reaction, the formed precipitates were filtered off, dried, and recrystallized from ethanol to afford **9a**,**b**.

##### B. Under Microwave Irradiation:

A mixture of 5-diamino-4-(arylazo)pyrazole **4a**,**b** (0.01 mol) and 2-arylazomalononitrile **2a**,**b** (0.01 mol) was grinded carefully in a porcelain mortar using a pestle and transferred to a pyrex test tube, and ethanol (4 mL) was added, followed by pyridine (0.5 mL). The reaction mixture was heated under microwave irradiation at 50% power for 2 min at 140 °C, monitored by TLC. After completion of the reaction, the reaction mixture was cooled to room temperature and the precipitated solid was filtered off, washed with MeOH, and recrystallized from ethanol to afford **9a**,**b**.

##### C-Under Ultrasound Condition:

To 5-diamino-4-(arylazo)pyrazole **4a**,**b** (0.01 mol) in ethanol (30 mL), 2-arylazomalononitrile **2a**,**b** (0.01 mol) and pyridine (0.5 mL) was added. The reaction mixture was sonicated for 1 h at room temperature (monitored by TLC). After completion of the reaction, the precipitate was filtered off, dried, and recrystallized from ethanol to afford **9a**,**b**.

*6-[(4-Trifluoromethyl)phenylazo]-3-[(4-fluorophenyl)azo]-2,5,7-triaminopyrazolo [1,5-a]pyrimidine (***9a***):* Yield: 76%; (orange plates): mp >300 °C; IR (KBr) ν_max_ in cm^−1^: 3474, 3276, 3123 (NH_2_), 1616 (C=N), 1423 (N=N); ^1^H-NMR (500 MHz, DMSO-*d*_6_): δ 6.98 (s, 2H, NH_2_), 7.31–7.35 (m, 4H, Ar–H), 7.76–7.82 (m, 6H, Ar–H+NH_2_), 8.22 (s, 2H, NH_2_); MS (*m*/*z*), 408 (45%), 458 (M^+^; 100%), 459 (M^+^ + 1; 22%). Anal. Calcd. for C_19_H_14_F_4_N_10_ (458.13): C, 49.79; H, 3.08; N, 30.56. Found C, 49.65; H, 3.19; N, 30.45%.

*3-[(4-Trifluoromethyl)phenylazo]-6-[(4-fluorophenyl)azo]-2,5,7-triaminopyrazolo [1,5-a]pyrimidine (***9b***):* Yield: 78%; (orange plates): mp >300 °C; IR (KBr) ν_max_ in cm^−1^: 3468, 3266, 3124 (NH_2_), 1594 (C=N), 1419 (N=N); ^1^H-NMR (500 MHz, DMSO-*d*_6_): δ 7.06 (s, 2H, NH_2_), 7.28–7.32 (m, 4H, Ar–H), 7.80 (d, 2H, Ar–H, *J* = 7.5 Hz), 8.10 (s, br, 2H, NH_2_), 8.13 (s, 2H, NH_2_), 7.84 (d, 2H, Ar–H, *J* = 7.5 Hz); ^13^C-NMR (125 MHz, DMSO-*d*_6_): δ 108.98, 116.48, 121.82, 122.25 (Ar–C), 123.19, 125.35 (q, C–F_3_, *J* = 270 Hz), 125.52, 125.99, 126.02, 126.17, 126.20, 126.90, 127.68, 128.11, 148.05 (Ar–C), 152.83, 154.90 (C=N), 161.27 (C–F). Anal. Calcd. for C_19_H_14_F_4_N_10_ (458.13): C, 49.79; H, 3.08; N, 30.56%. Found C, 49.50; H, 3.21; N, 30.46%.

#### 3.1.9. General Procedure for the Synthesis of 3,6-Diarylazo-2,5,7-triaminopyrazolo[1,5-*a*] pyrimidine Derivatives **10a**,**b**

##### A. Under reflux Condition:

To a solution of 4-arylazo-3,5-diaminopyrazole **4a**,**b** (0.01 mol) in ethanol (30 mL), *N*-[4-(*N*-butylsulfamoyl)phenyl]carbonohydrazonoyl dicyanide (3.05 g, 0.01 mol) and pyridine (0.5 mL) was added. The reaction mixture was heated under reflux for 8–9 h (monitored by TLC). After completion of the reaction, the precipitated product that formed was filtered off, dried, and recrystallized from toluene to afford 3-arylazo-2,5,7-triaminopyrazolo[1,5-*a*] pyrimidine **10a**,**b**.

##### B. Under Microwave Irradiation:

A mixture of 5-diamino-4-(arylazo)pyrazole **4a**,**b** (0.01 mol) and *N*-[4-(*N*-butylsulfamoyl)phenyl]carbonohydrazonoyl dicyanide (3.05 g, 0.01 mol) was grinded carefully in a porcelain mortar using a pestle and transferred to a pyrex test tube, and ethanol (4 mL) was added, followed by pyridine (0.5 mL). The reaction mixture was heated under microwave irradiation at 50% power for 5 min at 140 °C, monitored by TLC. After completion of the reaction, the reaction mixture was cooled to room temperature and the precipitated solid was filtered off, washed with MeOH, and recrystallized from ethanol to afford **10a**,**b**.

*N-Butyl-4-{[2,5,7-triamino-3-(4-fluorophenylazo)pyrazolo[1,5-a]pyrimidin-6-yl]azo}benzenesulfonamide (***10a***):* Yield: 76%; (orange plates): mp >300 °C; IR (KBr) ν_max_ in cm^−1^ 3474, 3279 (NH_2_), 2932, 2870 (C–H aliphatic), 1611 (C=N), 1420 (N=N), 1369 (SO_2_); ^1^H-NMR (500 MHz, DMSO-*d*_6_): δ 0.80 (t, 3H, CH_3_), 1.22–1.27 (m, 2H, CH_2_), 1.34–1.37 (m, 2H, CH_2_), 2.75–2.77 (t, 2H, CH_2_), 6.94 (s, 2H, NH_2_), 7.29–7.33 (m, 4H, Ar–H), 7.60 (br, s, 1H, NH), 7.74–7.76 (m, 4H, Ar–H), 7.83 (s, 2H, NH_2_), 8.18 (s, 2H, NH_2_); ^13^C-NMR (125 MHz, DMSO-*d*_6_): δ 13.43 (CH_3_), 19.19, 31.02 (CH_2_), 42.20 (N–CH_2_), 108.95, 115.28, 115.73, 115.91, 116.65, 120.81, 122.06, 122.43, 122.50, 123.91, 127.44, 127.68, 139.29, 147.51, 150.01 (Ar–C), 152.58, 154.48 (C=N), 160.63 (C–F); MS (*m*/*z*), 525 (M^+^; 11%), 526 (M^+^ + 1; 4%), 403 (100%). Anal. Calcd. for C_22_H_24_FN_11_O_2_S (525.57): C, 50.28; H, 4.60; N, 29.32; S, 6.10%. Found C, 50.39; H, 4.78; N, 29.39; S, 6.01%.

*N-Butyl-4-{[2,5,7-triamino-3-((4-(trifluoromethyl)phenyl)azo)pyrazolo[1,5-a]pyrimidin-6-yl]azo}benzene-sulfonamide (***10b***):* Yield: 84%; (reddish brown powder): mp 289–291 °C; IR (KBr) ν_max_ in cm^−1^: 3446, 3315 (NH_2_) 2960, 2870 (C-H aliphatic), 1611 (C=N), 1412 (N=N), 1366 (SO_2_); ^1^H-NMR (500 MHz, DMSO-*d*_6_): δ 0.79 (t, 3H, CH_3_), 1.23–1.26 (m, 2H, CH_2_), 1.34–1.37 (m, 2H, CH_2_), 2.76–2.77 (t, 2H, CH_2_), 7.07 (s, 2H, NH_2_), 7.61 (br, s, 1H, NH), 7.82–7.89 (m, 8H, Ar–H), 8.21 (br, s, 4H, 2NH_2_); ^13^C-NMR (125 MHz, DMSO-*d*_6_): δ 13.40 (CH_3_), 19.19, 31.01 (2CH_2_), 42.19 (N–CH_2_), 109.06, 116.58, 120.72, 120.89, 121.03 (Ar–C), 123.32, 125.48 (q, C–F_3_, *J* = 270 Hz), 126.22, 126.85, 127.10, 127.45, 127.69, 139.47, 148.04 (Ar–C), 152.40, 154.41 (C=N); MS (*m*/*z*), 576 (M^+^ + 1; 3%), 575 (M^+^; 9%), 398 (100%). Anal. Calcd. for C_23_H_24_F_3_N_11_O_2_S (575.58): C, 48.00; H, 4.20; N, 26.77%. Found C, 48.24; H, 4.14; N, 26.86%.

#### 3.1.10. General Procedure for the Synthesis of 3,6-Diarylazo-2,5,7-triaminopyrazolo[1,5-*a*] pyrimidine Derivatives **11a**,**b**

##### A. Under Reflux Condition:

To a solution of 5-diamino-4-(arylazo)pyrazole **4a**,**b** (0.01 mol) in ethanol (30 mL), *N*-{4-[*N*-(4,6-dimethylpyrimidin-2-yl)sulfamoyl]phenyl} carbonohydrazonoyl dicyanide (3.55 g, 0.01 mol) and pyridine (0.5 mL) were added. The reaction mixture was heated under reflux for 6–7 h, (monitored by TLC). After completion of the reaction, the precipitated product that formed was filtered off, dried, and recrystallized from toluene to afford 3-arylazo-2,5,7-triamino pyrazolo[1,5-*a*]pyrimidine **11a**,**b**.

##### B. Under Microwave Irradiation:

A mixture of 5-diamino-4-(arylazo)pyrazole **4a**,**b** (0.01 mol) and *N*-{4-[*N*-(4,6-dimethylpyrimidin-2-yl)sulfamoyl]phenyl}carbonohydrazonoyl dicyanide (3.55 g, 0.01 mol) was grinded carefully in a porcelain mortar using a pestle and transferred to a pyrex test tube, and ethanol (4 mL) was added, followed by pyridine (0.5 mL). The reaction mixture was heated under microwave irradiation at power 50% for 3 min at 140 °C, monitored by TLC. After completion of the reaction, the reaction mixture was cooled to room temperature and the precipitated solid was filtered off, washed with MeOH, and recrystallized from ethanol to afford **11a**,**b**.

*N-(4,6-Dimethylpyrimidin-2-yl)-4-{[2,5,7-triamino-3-(4-fluorophenylazo)pyrazolo [1,5-a]pyrimidin-6-yl]azo}benzenesulfonamide (***11a***):* Yield: 92%; (reddish-brown powder): mp > 300 °C; IR (KBr) ν_max_ in cm^−1^: 3403, 3307 (NH_2_), 2959 (C–H aliphatic), 1616 (C=N), 1421 (N=N), 1347 (SO_2_); ^1^H-NMR (500 MHz, DMSO-d_6_): δ 2.26 (s, 6H, 2CH_3_), 6.75 (s, 1H, CH–pyrimidine), 6.94 (s, 2H, NH_2_), 7.20–7.32 (m, 5H, Ar–H+NH), 7.73–7.76 (m, 4H, Ar–H), 8.03, 8.13 (2br, s, 4H, 2NH_2_); MS (*m*/*z*), 575 (M^+^; 41%), 408 (100%). Anal. Calcd. for C_24_H_22_FN_13_O_2_S (575.59): C, 50.08; H, 3.85; N, 31.64. Found C, 50.27; H, 3.97; N, 31.51%.

*N-(4,6-Dimethylpyrimidin-2-yl)-4-{[2,5,7-triamino-3-(4-(trifluoromethyl) phenylazo)pyrazolo[1,5-a]pyrimidin-6-yl]azo}benzenesulfonamide (***11b***):* Yield: 78%; (reddish orange powder): mp >300 °C; IR (KBr) ν_max_ in cm^−1^: 3418, 3324 (NH_2_), 1600 (C=N), 1418 (N=N), 1355 (SO_2_); ^1^H-NMR (500 MHz, DMSO-*d*_6_): δ 2.26 (s, 6H, 2CH_3_), 6.75 (s, 1H, CH–pyrimidine), 7.07 (s, 2H, NH_2_), 7.80 (d, 2H, Ar–H, *J* = 8.5 Hz), 7.85 (d, 2H, Ar–H, *J* = 8.5 Hz), 8.04 (d, 2H, Ar–H, *J* = 7.5 Hz), 8.14 (d, 2H, ArvH, *J* = 7.5 Hz), 8.24 (br, 4H, 2NH_2_), 9.5 (br, 1H, NH); ^13^C-NMR (125 MHz, DMSO-*d*_6_): δ 22.73 (CH_3_), 109.08, 113.25, 116.52, 120.70, 121.03, 121.36, 122.26 (Ar–C), 123.32, 125.48 (q, C–F_3_, *J* = 270 Hz), 126.20, 126.22, 126.86, 127.11, 127.64, 128.98, 139.73, 148.03 (Ar–C), 152.40, 154.50, 155.80, 156.08 (C=N). Anal. Calcd. C_25_H_22_F_3_N_13_O_2_S (625.60): C, 48.00; H, 3.54; N, 29.11. Found C, 48.18; H, 3.46; N, 29.08%.

#### 3.1.11. General Procedure for the Synthesis of 3,6-Diarylazo-2,5,7-triaminopyrazolo[1,5-*a*]pyrimidine Derivatives **12a**,**b**

##### A. Under Reflux Condition:

*N*-[4-(Piperidin-1-ylsulfonyl)phenyl]carbonhydrazonoyl dicyanide (3.17 g, 0.01 mol) and pyridine (0.5 mL) were added to a solution of 4-arylazo-3,5-diaminopyrazole **4a**,**b** (0.01 mol) in ethanol (30 mL). The reaction mixture was heated under reflux for 7 h (monitored by TLC). After completion of the reaction, the precipitates which formed were filtered off, dried, and recrystallized from ethanol to afford 3-arylazo-2,5,7-triaminopyrazolo[1,5-*a*]pyrimidine **12a**,**b**.

##### B. Under Microwave Irradiation:

A mixture of 4-arylazo-3,5-diaminopyrazole **4a**,**b** (0.01 mol) and *N*-[4-(piperidin-1-ylsulfonyl)phenyl]carbonhydrazonoyl dicyanide (3.17 g, 0.01 mol) was grinded carefully in a porcelain mortar using a pestle and transferred to a pyrex test tube, and ethanol (4 mL) was added, followed by pyridine (0.5 mL). The reaction mixture was heated under microwave irradiation at power 50% for 3–7 min at 140 °C (monitored by TLC). After completion of the reaction, the reaction mixture was cooled to room temperature and the precipitated solid was filtered off, washed with MeOH, and recrystallized from ethanol to afford **12a**,**b**.

*3-[(4-Fluorophenyl)azo]-6-[4-(piperidin-1-ylsulfonyl)phenylazo]-2,5,7-triamino pyrazolo[1,5-a]pyrimidine (***12a***):* Yield: 80%; (orange plates): mp >300 °C; IR (KBr) ν_max_ in cm^−1^: 3404, 3266 (NH_2_), 2940, 2835 (C-H aliphatic), 1611 (C=N), 1420 (N=N), 1371 (SO_2_); ^1^H-NMR (500 MHz, DMSO-*d*_6_): δ 1.36–1.37 (m, 2H, CH_2_), 1.54–1.55 (m, 4H, 2CH_2_), 2.91–2.93 (m, 4H, 2NCH_2_), 6.95 (s, 2H, NH_2_), 7.29–7.33 (m, 4H, Ar–H), 7.74–7.77 (m, 6H, Ar–H+NH_2_), 8.21 (br, s, 2H, NH_2_); ^13^C-NMR (125 MHz, DMSO-*d*_6_): δ 22.85 (CH_2_), 24.67 (CH_2_), 46.58 (N–CH_2_), 109.17, 115.30, 115.74, 115.92, 120.88, 122.14, 122.45, 122.51, 128.38, 128.67, 134.07, 147.54, 149.99 (Ar–C), 152.62, 154.97 (C=N), 160.64 (C–F). Anal. Calcd. for C_23_H_24_FN_11_O_2_S (537.58): C, 51.39; H, 4.50; N, 28.66. Found C, 51.32; H, 4.73; N, 28.49%.

*6-[(4-Piperidin-1-ylsulfonyl)phenylazo]-3-[4-(trifluoromethyl)phenylazo]-2,5,7-triaminopyrazolo[1,5-a]pyrimidine (***12b***):* Yield: 81%; (reddish brown crystals): mp > 300 °C; IR (KBr) ν_max_ in cm^−1^: 3439, 3273 (NH_2_), 2941, 2855 (C-H aliphatic), 1607 (C=N), 1409 (N=N), 1361 (SO_2_); ^1^H-NMR (500 MHz, DMSO-*d*_6_): δ 1.36 (m, 2H, CH_2_), 1.55 (m, 4H, 2CH_2_), 2.91 (m, 4H, 2N–CH_2_), 7.07 (s, 2H, NH_2_), 7.76–7.87 (m, 10H, Ar–H+NH_2_), 8.23 (br, s, 2H, NH_2_); ^13^C-NMR (125 MHz, DMSO-*d*_6_): δ 22.85 (CH_2_), 24.66 (CH_2_), 46.58 (N–CH_2_), 109.27, 116.59, 120.62, 120.97, 121.06, 122.24 (Ar–C), 123.33, 125.50, (q, C–F_3_, *J* = 271.2 Hz), 126.22, 126.25, 126.87, 127.13, 128.38, 128.66, 134.28, 148.10 (Ar–C), 152.43, 154.90 (C=N); MS (*m*/*z*), 588 (M^+^ + 1; 21%), 587 (M^+^; 44%), 442 (100%), 54 (99%). Anal. Calcd. for C_24_H_24_F_3_N_11_O_2_S (587.59): C, 49.06; H, 4.12; N, 26.22. Found C, 49.21; H, 4.32; N, 26.41%.

#### 3.1.12. Synthesis of 6-(Thiazol-2-yldiazenyl)-3-(4-(trifluoro methyl)phenylazo)-2,5,7-triaminopyrazolo[1,5-*a*] pyrimidine (**13**)

##### A. Under reflux Condition:

*N*-(Thiazol-2-yl)carbonohydrazonoyl dicyanide (1.77 g, 0.01 mol) and pyridine (0.5 mL) were added to a solution of 3,5-diamino-4-[4-(trifluoromethyl)phenylazo]-1*H*-pyrazole (**4b**) (2.7 g, 0.01 mol) in ethanol (30 mL). The reaction mixture was heated under reflux for 12 h (monitored by TLC). After completion of the reaction, the precipitate which formed was filtered off, dried, and recrystallized from ethanol to afford 6-(thiazol-2-yldiazenyl)-3-(4-(trifluoromethyl)phenylazo)-2,5,7-triaminopyrazolo[1,5-*a*] pyrimidine (**13**).

##### B. Under Microwave Irradiation:

A mixture of 3,5-diamino-4-[4-(trifluoromethyl)phenylazo]-1*H*-pyrazole (**4b**) (2.7 g, 0.01 mol) and *N*-(thiazol-2-yl)carbonohydrazonoyl dicyanide (1.77 g, 0.01 mol) was grinded carefully in a porcelain mortar using a pestle and transferred to a pyrex test tube, and ethanol (4 mL) was added, followed by pyridine (0.5 mL). The reaction mixture was heated under microwave irradiation at 50% power for 8 min at 140 °C, monitored by TLC. After completion of the reaction, the reaction mixture was cooled to room temperature and the precipitated solid was filtered off, washed with MeOH, and recrystallized from ethanol to give **13**.

Yield: 78%; (brown powder): mp > 300 °C; IR (KBr) ν_max_ in cm^−1^: 3415, 3309 (NH_2_), 1608 (C=N), 1419 (N=N); ^1^H-NMR (500 MHz, DMSO-*d*_6_): δ 7.11 (s, 2H, NH_2_), 7.46 (d, 1H, CH–thiazole, *J* = 3.5 Hz), 7.65 (d, 1H, CH–thiazol, *J* = 3.5 Hz), 7.77–7.89 (m, 6H, Ar–H+NH_2_), 8.24 (br, s, 2H, NH_2_); ^13^C-NMR (125 MHz, DMSO-*d*_6_): δ 109.10, 109.20, 116.78, 119.63, 121.15, 121.34, 122.43 (Ar–C), 123.30, 125.46 (q, C–F_3_, *J* = 270 Hz), 126.04, 126.24, 126.27, 142.47 (Ar–C), 152.76, 155.58, (2C=N), 167.33 (C=N thiazole); MS (*m*/*z*), 448 (M^+^ + 1; 24%), 447 (M^+^; 100%). Anal. Calcd. for C_16_H_12_F_3_N_11_S (447.40): C, 42.95; H, 2.70; N, 34.44. Found C, 42.81; H, 2.49; N, 34.38%.

### 3.2. Biological Evaluation

#### 3.2.1. In Vitro Antimicrobial Evaluation

Antimicrobial activities were carried out against highly pathogenic strains—three strains of Gram-positive bacteria (*Staphylococcus aureus, Bacillus subtilis*, and *Staphylococcus mutants*), three strains of Gram-negative bacteria (*Enterococcus faecalis*, *proteus vulgaris*, and *Escherichia coli*) using the standard antibiotic Gentamycin (5 mg/mL) as reference drugs, and fungi (*Aspergillus fumigates, Aspergillus flavus*, and *Candida Albicans*) using the standard antibiotic Ketoconazol (5 mg/mL). Antimicrobial activity of the tested samples was determined by using the agar diffusion method using Mueller–Hinton agar medium for bacteria and Sabouraud’s agar medium for fungi [19,20].

The tested microorganisms were obtained from the Regional Center for Mycology and Biotechnology (RCMP), Al-Azhar University. The assayed collection included Gram-positive (*Staphylococcus aureus*, *Bacillus subtilis*, and *Staphylococcus mutants*), Gram-negative bacteria (*Enterococcus faecalis*, *proteus vulgaris*, and *Escherichia coli*) using the standard antibiotic Gentamycin (5 mg/mL) as reference drugs, and fungi (*Aspergillus fumigates*, *Aspergillus flavus*, and *Candida Albicans*) using the standard antibiotic Ketoconazole (5 mg/mL). The mean zone of inhibition in mm ± standard deviation beyond the diameter (6 mm) was determined using a 5 µg/mL concentration of the tested compounds. The inhibitory effects of the synthetic compounds against these organisms are listed in Table 3.

#### 3.2.2. In Vitro Cytotoxic Screening

Cell culture: Human hepatocellular carcinoma cell line (HepG-2), colorectal adenocarcinoma cell line (HCT-116), and breast adenocarcinoma cell line (MCF-7 cell) were obtained from the American type culture collection (ATCC). Cells were maintained in RPMI-1640 supplemented with (100 μg/mL), penicillin (100 units/mL), and heat-inactivated fetal bovine serum (10% *v*/*v*) in a humidified, 5% (*v*/*v*) CO_2_ atmosphere at 37 °C [24].

Cytotoxicity assessment: The cytotoxicity of different compounds was tested against human tumor cells using Sulphorhodamine B assay (SRB). Healthy growing cells were cultured in a 96-well tissue culture plate (3000 cells/well) for 24 h before treatment with the synthesized compounds to allow attachment of the cells to the plate. Cells were exposed to the five different concentrations of tested compounds (0.01, 0.1, 1, 10, and 100 μM/mL); untreated cells (control) were added. Triplicate wells were incubated with the different concentrations for 72 h and subsequently fixed with TCA (10% *w*/*v*) for 1 h at 4 °C. After several washing, cells were stained by 0.4% (*w*/*v*) SRB solution for 10 min in a dark place. Excess stain was washed with 1% (*v*/*v*) glacial acetic acid. After drying overnight, the SRB-stained cells were dissolved with tris–HCl and the color intensity was measured in a microplate reader at 540 nm. The linear relation between viability percentage of each tumor cell line and extracts concentrations were analyzed to get the IC_50_ (dose of the drug which reduces survival to 50%) using Sigma Plot 12.0 software [25].

## 4. Conclusions

In conclusion, 4-arylazo-3,5-diaminopyrazole **4**–**6** was synthesized and used as a building block for the synthesis of pyrazolopyrimidine derivatives such as 3,6-diarylazo-2,5,7-triaminopyrazolo[1,5-*a*]pyrimidine, **8** and **9**, 3,6-Diarylazo-2,5,7-triaminopyrazolo[1,5-*a*]pyrimidine **10–12** and **13**. The structures of newly synthesized compounds were confirmed by spectral data (IR, ^1^H-NMR, ^13^C-NMR, and mass spectra) and elemental analysis. Most of the newly synthesized compounds were assessed as antimicrobial agents against a number of Gram-positive, Gram-negative, and fungal strains. Some of the tested compounds showed excellent antimicrobial activity compared to the standard antibiotics. The obtained data reflected that compound **2b** exhibited the best antimicrobial activity against most tested microorganisms and the rest of the compounds were moderately active or inactive for all the microorganisms. Most of the synthesized compounds exhibited good cytotoxic activity towards the examined three cell lines. From the obtained results, compounds **2a**,**b**, **4b**, **5b**, **8b**, and **12b** showed the most potent cytotoxic profile on cancer cells (MCF-7, HepG2, and HCT-116) with IC_50_ values ranging from 0.3 to 3.4 µg, while the compound **8b** is most effective among all of them on all types of cancer cells.

## Figures and Tables

**Table 1:** Comparison between methods for the synthesis of compounds **8a**,**b** and **9a**,**b**.

Compound No.	Reflux		MW		US	
	Time	Yield%	Time	Yield%	Time	Yield%
**8a**	300 min.	62	2 min.	74	60 min.	52
**8b**	240 min.	60	2 min.	71	60 min.	40
**9a**	360 min.	63	2 min.	76	60 min.	60
**9b**	420 min.	67	2 min.	78	60 min.	43

**Table 2:** Comparison between methods for the synthesis of compounds **10a**,**b**–**13**.

Compound No.	Reflux		MW	
	Time	Yield%	Time	Yield%
**10a**	540 min.	70	5 min.	76
**10b**	480 min.	78	5 min.	84
**11a**	360 min.	88	3 min.	92
**11b**	420 min.	72	3 min.	78
**12a**	420 min.	73	3 min.	80
**12b**	420 min.	75	7 min.	81
**13**	720 min.	69	8 min.	78

**Table 3:** Inhibition zone in (mm) as a criterion of antimicrobial activity of some newly synthesized compounds.

Microorganism Inhibition Zone Diameter (mm)									
Compound No.	Gram +ve Bacteria			Gram -ve Bacteria			Fungi		
	*Staphylococcus aureus*	*Bacillus subtilis*	*Strepotococcus mutants*	*Enterococcus faecalis*	*proteus vulgaris*	*Escherichia coli*	*Aspergillus fumigates*	*Aspergillus flavus*	*Candida Albicans*
**2a**	25	40	22	35	40	28	24	20	21
**2c**	22	24	18	25	26	14	NA	NA	NA
**4b**	23	23	24	22	23	20	20	13	15
**5a**	12	8	NA	11	8	12	NA	NA	NA
**5b**	18	25	20	22	28	20	15	10	16
**7a**	14	16	14	13	20	16	15	9	11
**7b**	15	17	19	18	16	13	NA	NA	NA
**8a**	NA	NA	NA	NA	NA	10	NA	NA	NA
**8b**	NA	8	NA	NA	NA	NA	NA	NA	NA
**9a**	NA	NA	NA	NA	NA	NA	NA	NA	NA
**9b**	10	NA	NA	NA	NA	NA	NA	NA	NA
**10a**	NA	NA	NA	NA	NA	NA	NA	NA	NA
**10b**	NA	NA	NA	NA	NA	NA	NA	NA	NA
**11a**	18	12	9	13	9	11	NA	NA	NA
**11b**	22	23	29	25	26	13	20	17	15
**12a**	NA	NA	NA	NA	NA	NA	NA	NA	NA
**12b**	NA	NA	NA	NA	NA	NA	NA	NA	NA
**13**	NA	NA	NA	NA	NA	NA	NA	NA	NA
**Gentamycin**	24	26	20	26	25	30	-	-	-
**Ketoconazole**	-	-	-	-	-	-	17	16	20

NA = not active; Diameter of hole = 6 mm; data are expressed in the form of mean ± SD.

**Table 4:** The IC_50_ (µg) of the new tested chemicals compounds against different tumor cell lines.

Compound	IC_50_ (μg/mL)		
	MCF-7	HepG2	HCT-116
**2a**	1.3 ± 0.7	0.5 ± 0.2	1.2 ± 0.1
**2b**	0.5 ± 0.04	1.1 ± 0.3	0.5 ± 0.2
**2c**	5.9 ± 0.2	9.8 ± 0.5	1.2 ± 0.12
**3a**	18.8 ± 1.5	6.1 ± 1.1	19.1 ± 3.1
**3b**	19.4 ± 2.6	19.3 ± 5.7	27.8 ± 2.6
**4a**	32.1 ± 1.8	6.7 ± 0.9	11.5 ± 1.1
**4b**	1.7 ± 0.2	1.1 ± 0.1	1.1 ± 0.08
**5a**	24.6 ± 3.2	73.6 ± 3.1	24.1 ± 0.7
**5b**	2.8 ± 0.7	0.9 ± 0.1	3.7 ± 0.7
**7a**	16.3 ± 0.6	14.3 ± 2.6	14.1 ± 1.9
**7b**	20.8 ± 1.7	21.3 ± 3.1	15.7 ± 1.7
**8a**	10.7 ± 0.3	4.9 ± 0.6	11.1 ± 0.5
**8b**	0.3 ± 0.01	0.6 ± 0.2	0.4 ± 0.02
**9a**	106 ± 4.3	226.9 ± 3.2	103.8 ± 5.1
**9b**	38.5 ± 0.5	18.9 ± 0.8	31.1 ± 2.5
**10a**	10.1 ± 2.6	7.5 ± 0.7	12.9 ± 1.6
**10b**	43.9 ± 1.1	85.9 ± 3.4	29.4 ± 2.4
**11a**	19.8 ± 1.9	26.6 ± 4.4	25.9 ± 1.7
**11b**	4.5 ± 0.4	3.9 ± 0.4	2.7 ± 0.6
**12a**	18.8 ± 1.7	31.4 ± 2.7	46.1 ± 5.9
**12b**	1.4 ± 0.03	3.4 ± 0.6	2.4 ± 0.4
**13**	4.2 ± 0.2	4.1 ± 0.3	5.3 ± 0.8
**Doxorubicin**	1.2 ± 0.2	0.9 ± 0.3	1.6 ± 0.2

## Supplementary Materials

The Supplementary Materials are available online.
