# Supplementary material for: Synthesis, In Vitro Antimicrobial and Cytotoxic Activities of Some New Pyrazolo[1,5-a]pyrimidine Derivatives
Source: Molecules. 2019 Mar 19;24(6):1080. doi: 10.3390/molecules24061080 (PMC6471749; doi:10.3390/molecules24061080)
Supplement: Supplementary file 1 [file molecules-24-01080-s001.pdf]

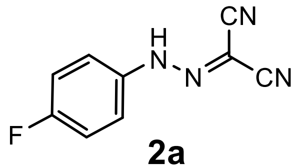
CN
H
N
N
CN
F

2a

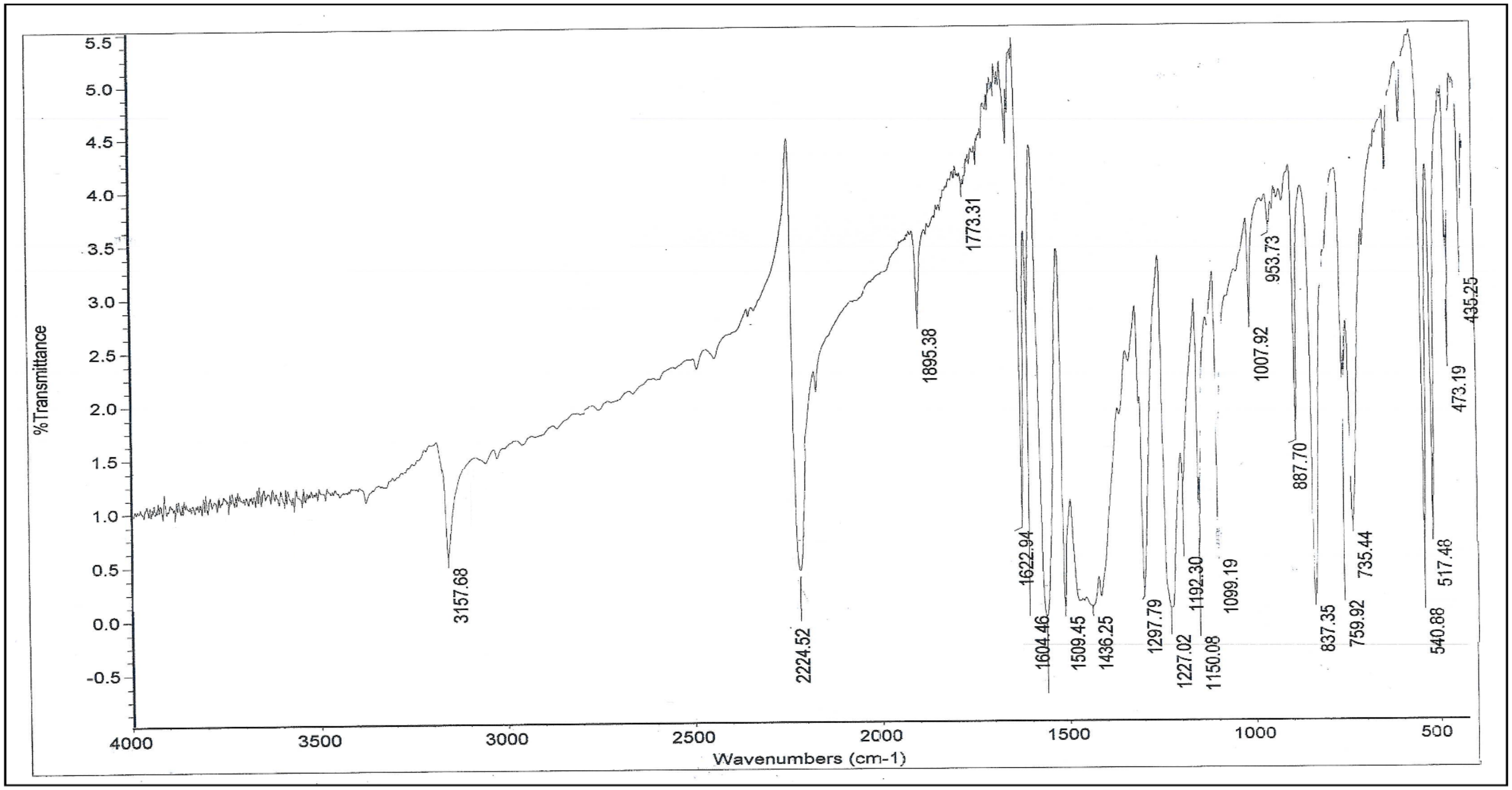
%Transmittance
Wavenumbers (cm-1)
3157.68
2224.52
1895.38
1773.31
1622.94
1604.46
1509.45
1436.25
1297.79
1227.02
1192.30
1150.08
1099.19
1007.92
953.73
887.70
837.35
759.92
735.44
540.88
517.48
473.19
435.25

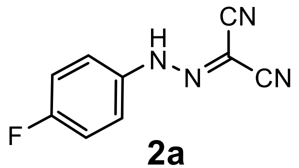

CN
H
N
N
CN
F

2a

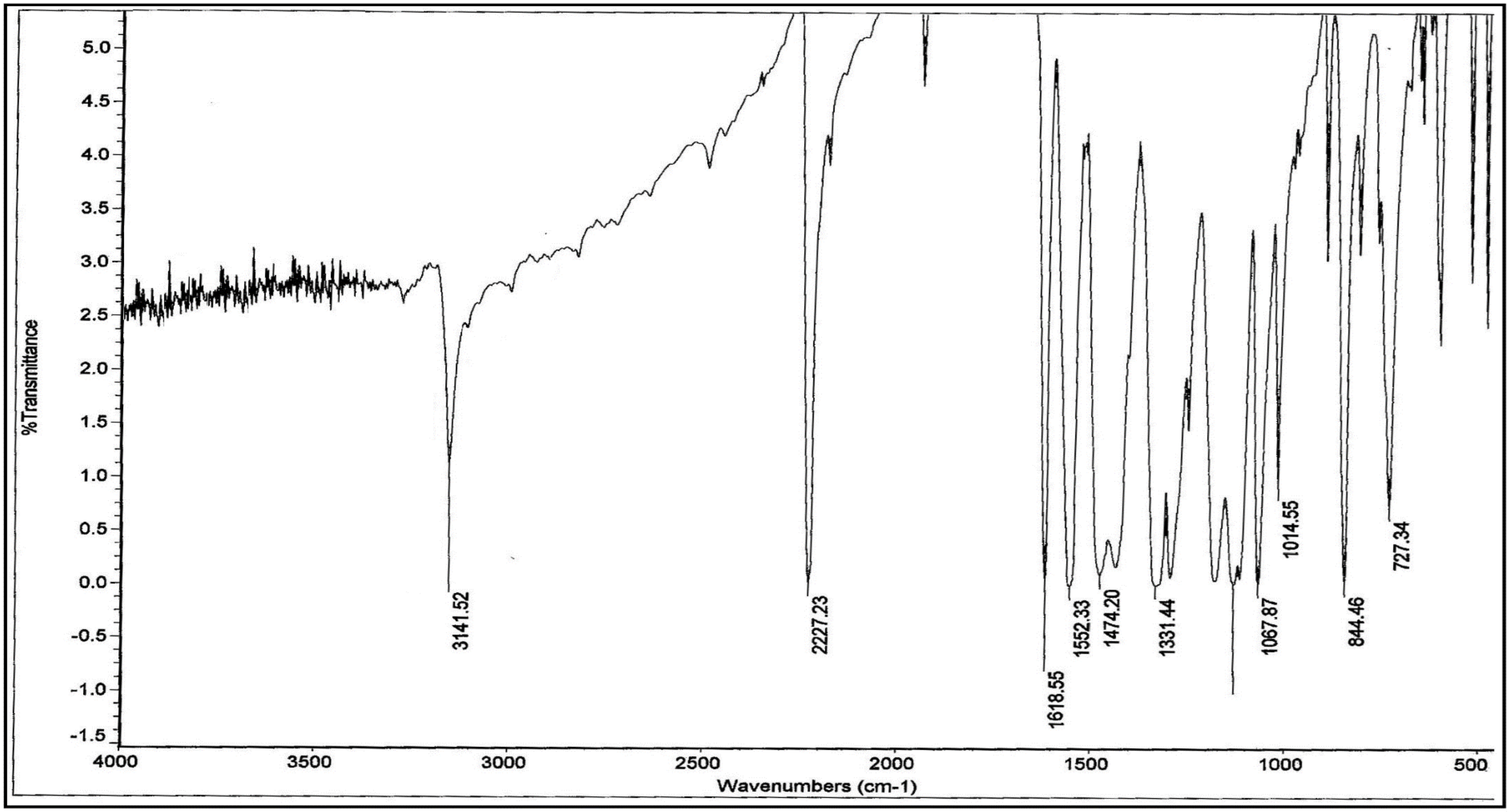

%Transmittance
5.0
4.5
4.0
3.5
3.0
2.5
2.0
1.5
1.0
0.5
0.0
-0.5
-1.0
-1.5
3141.52
2227.23
1618.55
1552.33
1474.20
1331.44
1067.87
1014.55
844.46
727.34
4000
3500
3000
2500
2000
1500
1000
500
Wavenumbers (cm-1)

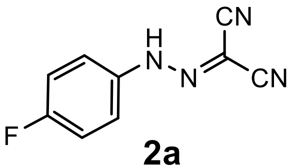
CN
H
N
N
CN
F

2a

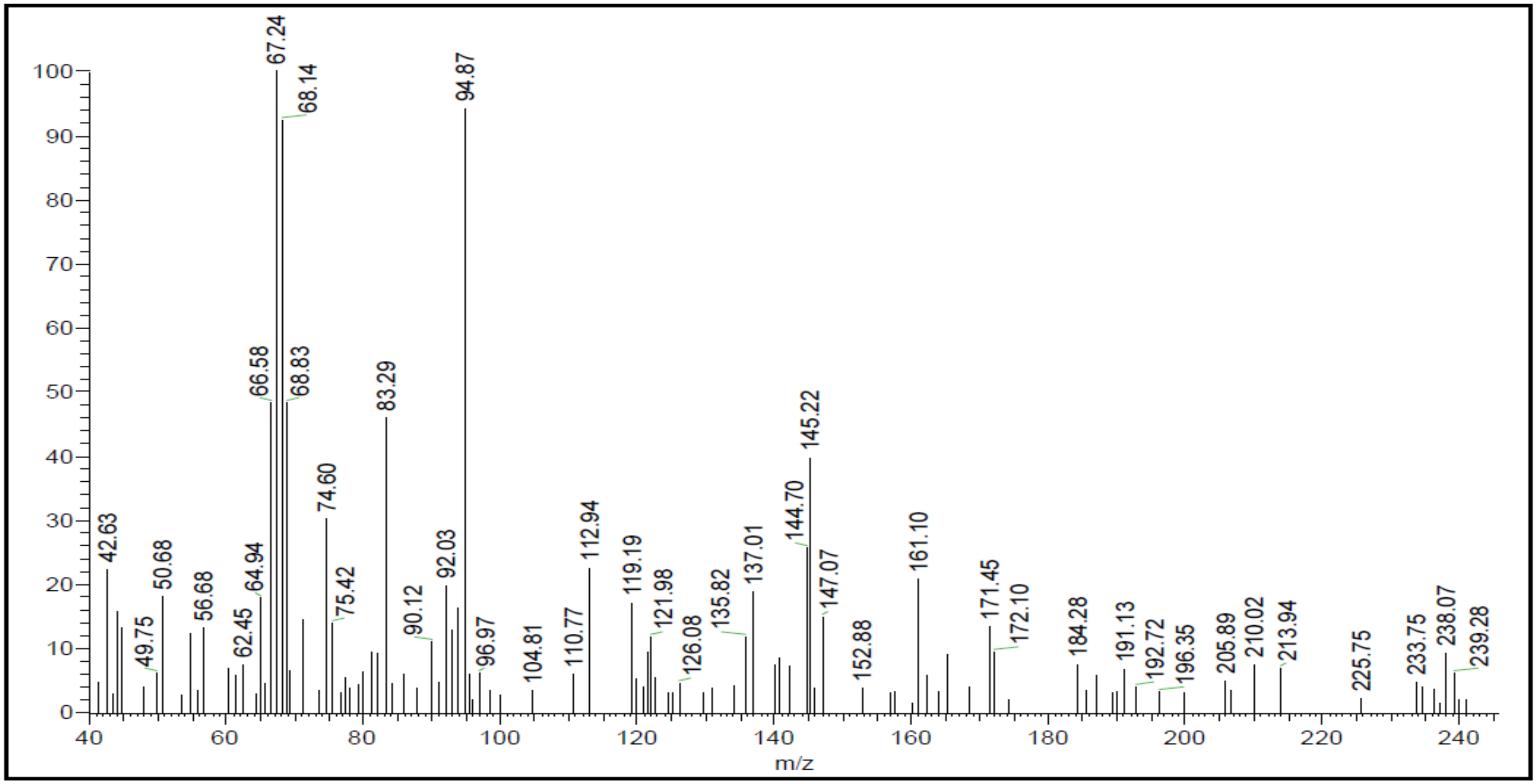
m/z
67.24
68.14
94.87
66.58
68.83
83.29
74.60
42.63
50.68
56.68
64.94
49.75
62.45
75.42
90.12
92.03
96.97
104.81
110.77
112.94
119.19
121.98
126.08
135.82
137.01
144.70
145.22
147.07
152.88
161.10
171.45
172.10
184.28
191.13
192.72
196.35
205.89
210.02
213.94
225.75
233.75
238.07
239.28

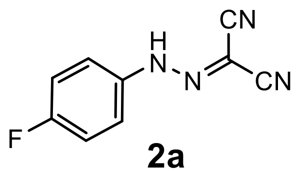

2a

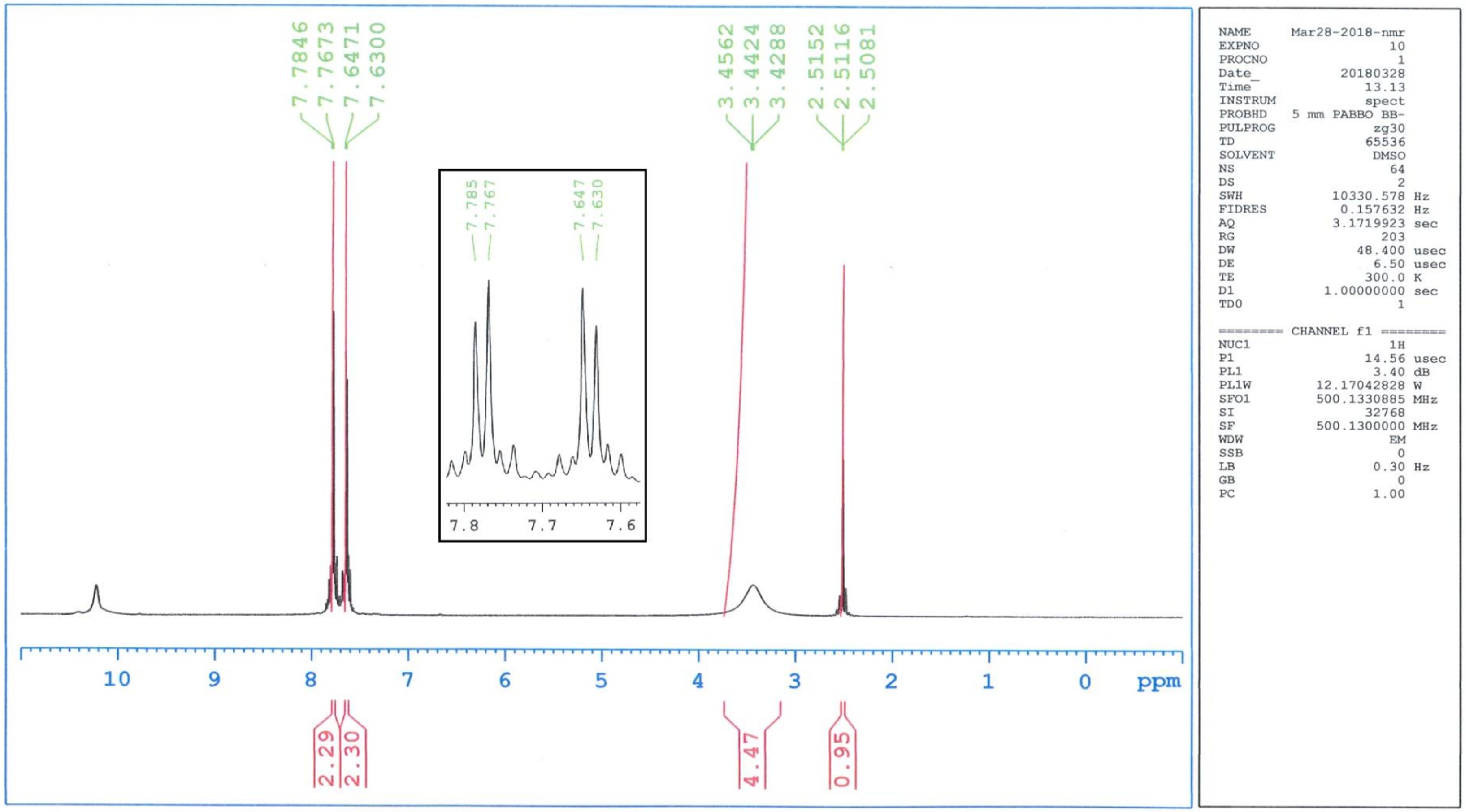

NAME	Mar28-2018-nmr
EXPNO	10
PROCNO	1
Date_	20180328
Time	13.13
INSTRUM	spect
PROBHD	5 mm PABBO BB-
PULPROG	zg30
TD	65536
SOLVENT	DMSO
NS	64
DS	2
SWH	10330.578 Hz
FIDRES	0.157632 Hz
AQ	3.1719923 sec
RG	203
DW	48.400 usec
DE	6.50 usec
TE	300.0 K
D1	1.00000000 sec
TD0	1
======== CHANNEL f1 ========	
NUC1	1H
P1	14.56 usec
PL1	3.40 dB
PL1W	12.17042828 W
SFO1	500.1330885 MHz
SI	32768
SF	500.1300000 MHz
WDW	EM
SSB	0
LB	0.30 Hz
GB	0
PC	1.00

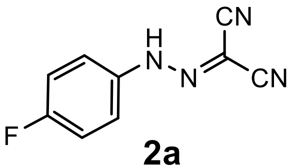
CN
H
N
N
CN
F

2a

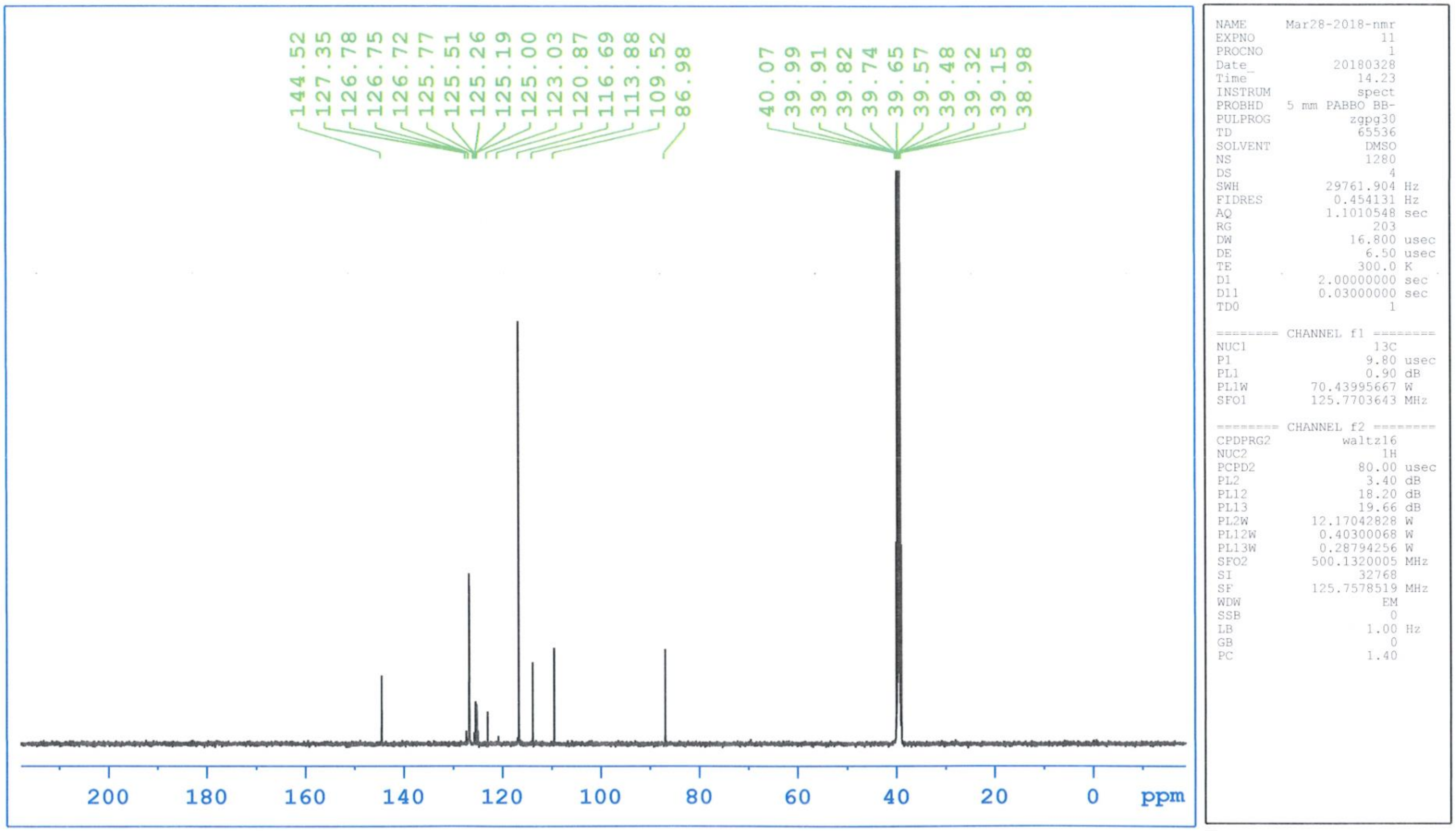
144.52
127.35
126.78
126.75
126.72
125.77
125.51
125.26
125.19
125.00
123.03
120.87
116.69
113.88
109.52
86.98
40.07
39.99
39.91
39.82
39.74
39.65
39.57
39.48
39.32
39.15
38.98
200 180 160 140 120 100 80 60 40 20 0 ppm
NAME Mar28-2018-nmr
EXPNO 11
PROCNO 1
Date_ 20180328
Time 14.23
INSTRUM spect
PROBHD 5 mm PABBO BB-
PULPROG zgpg30
TD 65536
SOLVENT DMSO
NS 1280
DS 4
SWH 29761.904 Hz
FIDRES 0.454131 Hz
AQ 1.1010548 sec
RG 203
DW 16.800 usec
DE 6.50 usec
TE 300.0 K
D1 2.00000000 sec
D11 0.03000000 sec
TD0 1
======== CHANNEL f1 ========
NUC1 13C
P1 9.80 usec
PL1 0.90 dB
PL1W 70.43995667 W
SFO1 125.7703643 MHz
======== CHANNEL f2 ========
CPDPRG2 waltz16
NUC2 1H
PCPD2 80.00 usec
PL2 3.40 dB
PL12 18.20 dB
PL13 19.66 dB
PL2W 12.17042828 W
PL12W 0.40300068 W
PL13W 0.28794256 W
SFO2 500.1320005 MHz
SI 32768
SF 125.7578519 MHz
WDW EM
SSB 0
LB 1.00 Hz
GB 0
PC 1.40

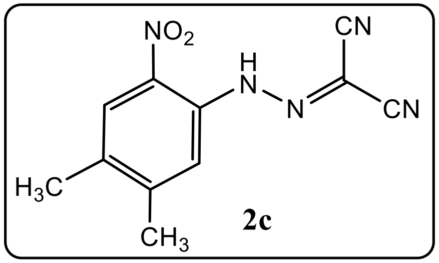
NO2
H
N
N
CN
CN
H3C
CH3

2c

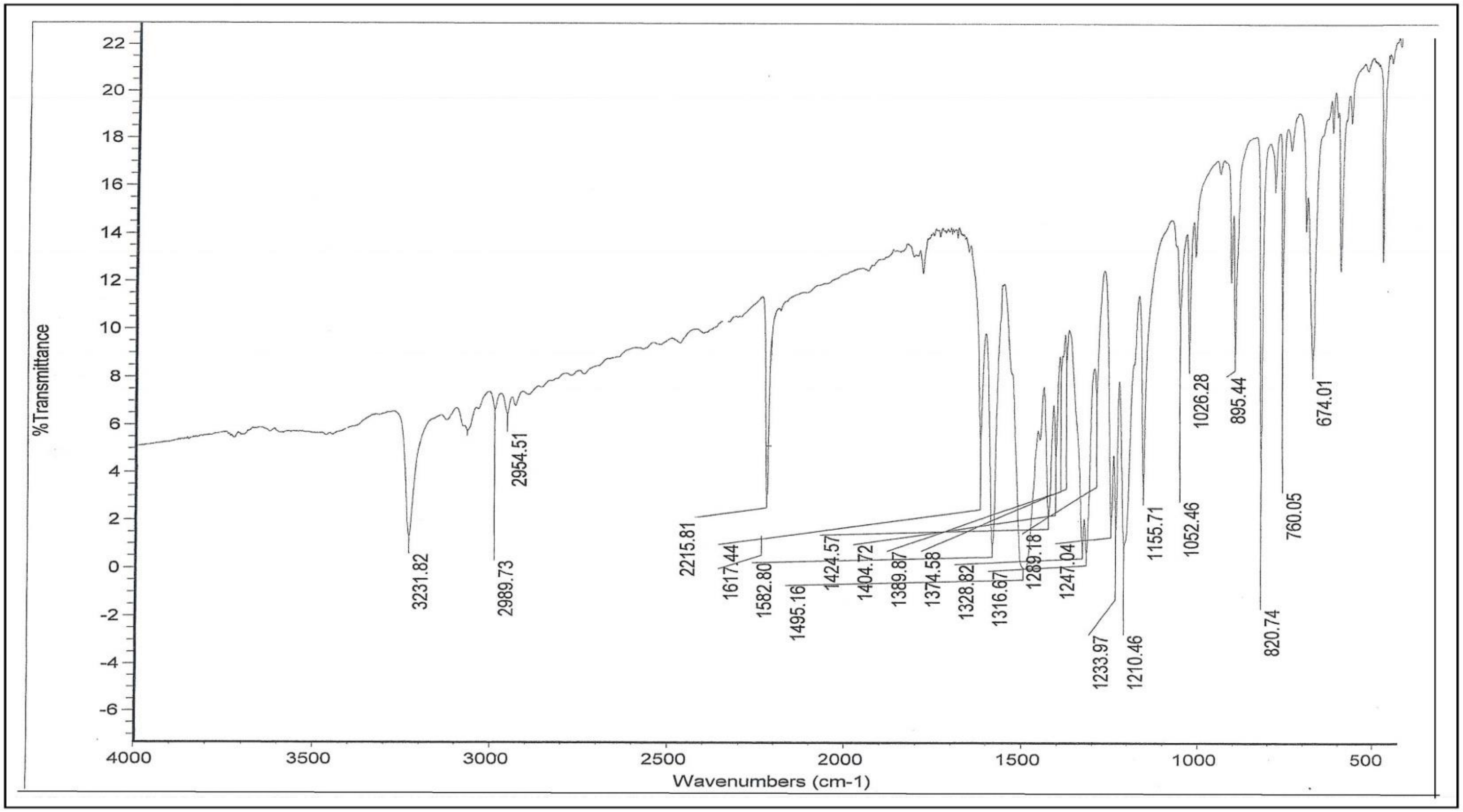
%Transmittance
Wavenumbers (cm-1)
4000
3500
3000
2500
2000
1500
1000
500
22
20
18
16
14
12
10
8
6
4
2
0
-2
-4
-6
3231.82
2989.73
2954.51
2215.81
1617.44
1582.80
1495.16
1424.57
1404.72
1389.87
1374.58
1328.82
1316.67
1289.18
1247.04
1233.97
1210.46
1155.71
1052.46
1026.28
895.44
820.74
760.05
674.01

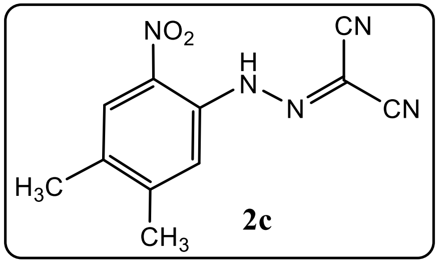
NO2
H
N
N
CN
CN
H3C
CH3

2c

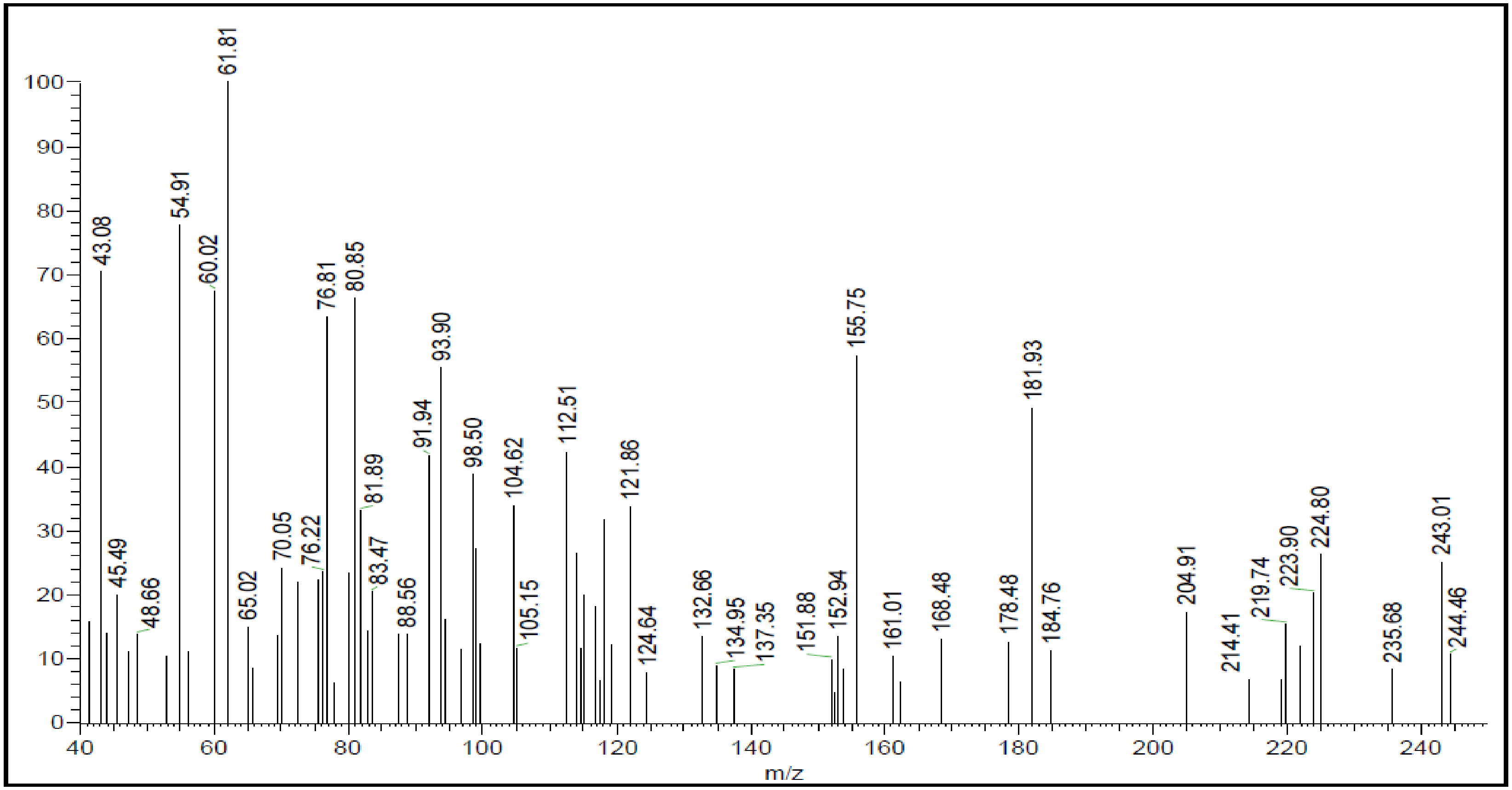
43.08
45.49
48.66
54.91
60.02
61.81
65.02
70.05
76.22
76.81
80.85
81.89
83.47
88.56
91.94
93.90
98.50
104.62
105.15
112.51
121.86
124.64
132.66
134.95
137.35
151.88
152.94
155.75
161.01
168.48
178.48
181.93
184.76
204.91
214.41
219.74
223.90
224.80
235.68
243.01
244.46
m/z

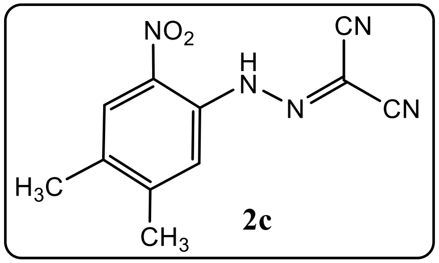

2c

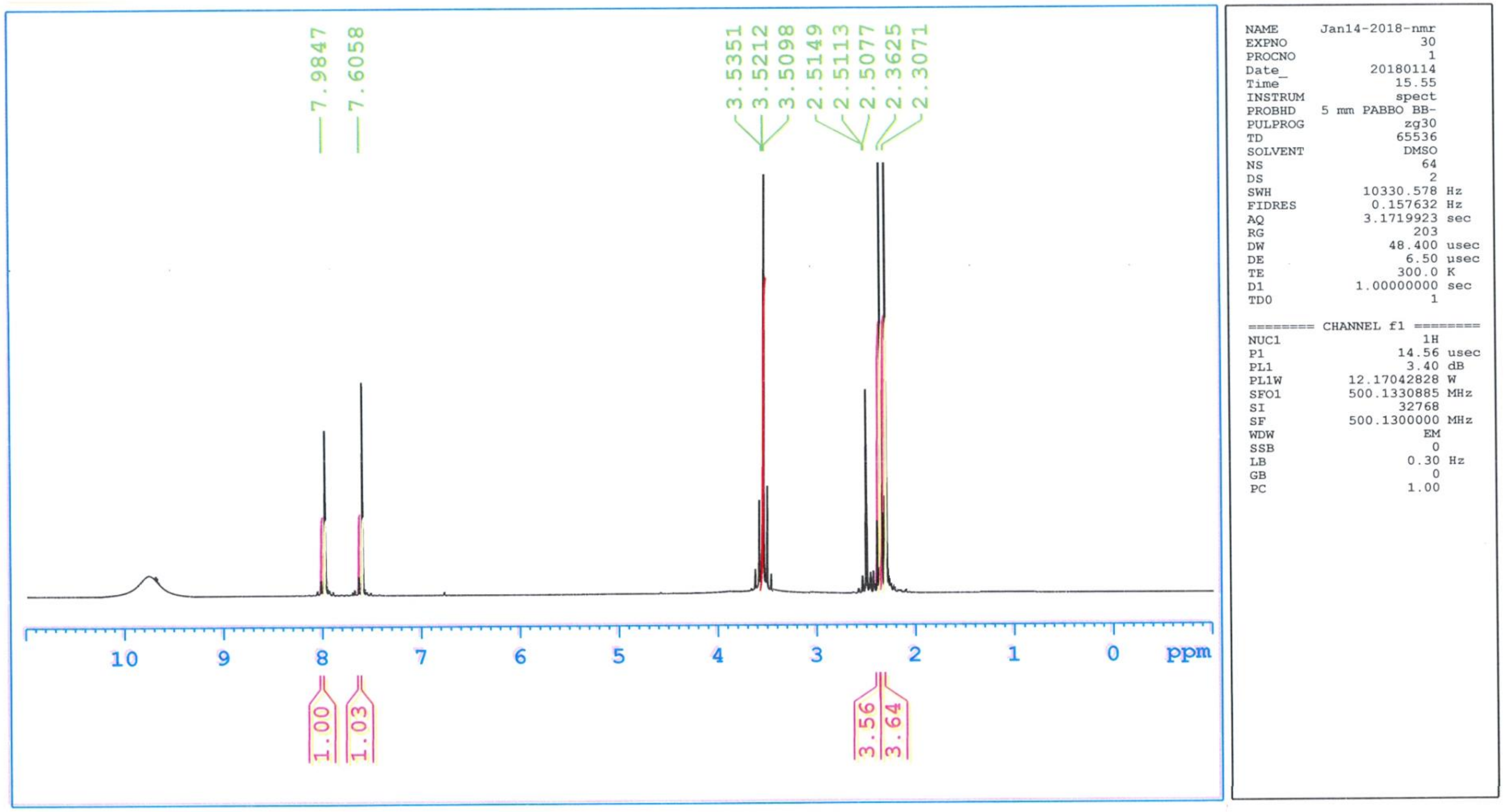

NAME	Jan14-2018-nmr
EXPNO	30
PROCNO	1
Date_	20180114
Time	15.55
INSTRUM	spect
PROBHD	5 mm PABBO BB-
PULPROG	zg30
TD	65536
SOLVENT	DMSO
NS	64
DS	2
SWH	10330.578 Hz
FIDRES	0.157632 Hz
AQ	3.1719923 sec
RG	203
DW	48.400 usec
DE	6.50 usec
TE	300.0 K
D1	1.00000000 sec
TD0	1
======== CHANNEL f1 ========	
NUC1	1H
P1	14.56 usec
PL1	3.40 dB
PL1W	12.17042828 W
SFO1	500.1330885 MHz
SI	32768
SF	500.1300000 MHz
WDW	EM
SSB	0
LB	0.30 Hz
GB	0
PC	1.00

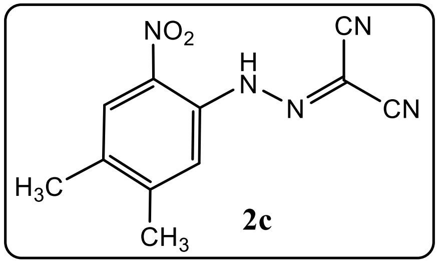
NO2
H
N
N
CN
CN
H3C
CH3

2c

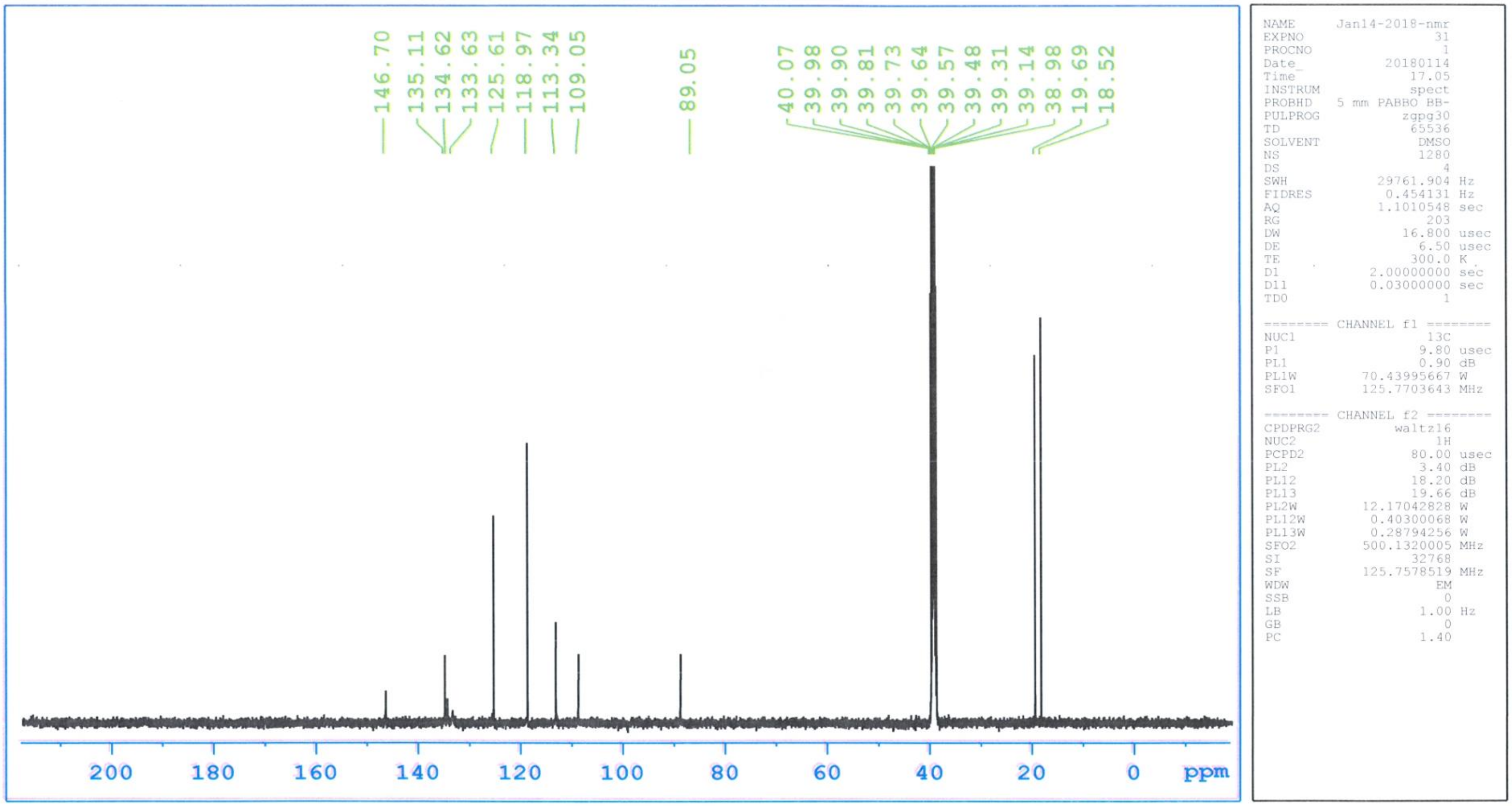
146.70
135.11
134.62
133.63
125.61
118.97
113.34
109.05
89.05
40.07
39.98
39.90
39.81
39.73
39.64
39.57
39.48
39.31
39.14
38.98
19.69
18.52
200 180 160 140 120 100 80 60 40 20 0 ppm
NAME Jan14-2018-nmr
EXPNO 31
PROCNO 1
Date_ 20180114
Time 17.05
INSTRUM spect
PROBHD 5 mm PABBO BB-
PULPROG zgpg30
TD 65536
SOLVENT DMSO
NS 1280
DS 4
SWH 29761.904 Hz
FIDRES 0.454131 Hz
AQ 1.1010548 sec
RG 203
DW 16.800 usec
DE 6.50 usec
TE 300.0 K
D1 2.00000000 sec
D11 0.03000000 sec
TD0 1
======== CHANNEL f1 ========
NUC1 13C
P1 9.80 usec
PL1 0.90 dB
PL1W 70.43995667 W
SFO1 125.7703643 MHz
======== CHANNEL f2 ========
CPDPRG2 waltz16
NUC2 1H
PCPD2 80.00 usec
PL2 3.40 dB
PL12 18.20 dB
PL13 19.66 dB
PL2W 12.17042828 W
PL12W 0.40300068 W
PL13W 0.28794256 W
SFO2 500.1320005 MHz
SI 32768
SF 125.7578519 MHz
WDW EM
SSB 0
LB 1.00 Hz
GB 0
PC 1.40

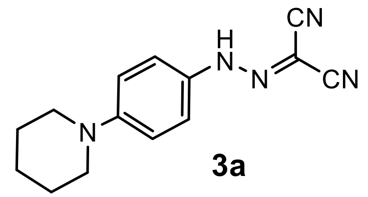

H
N
N
CN
CN
N

3a

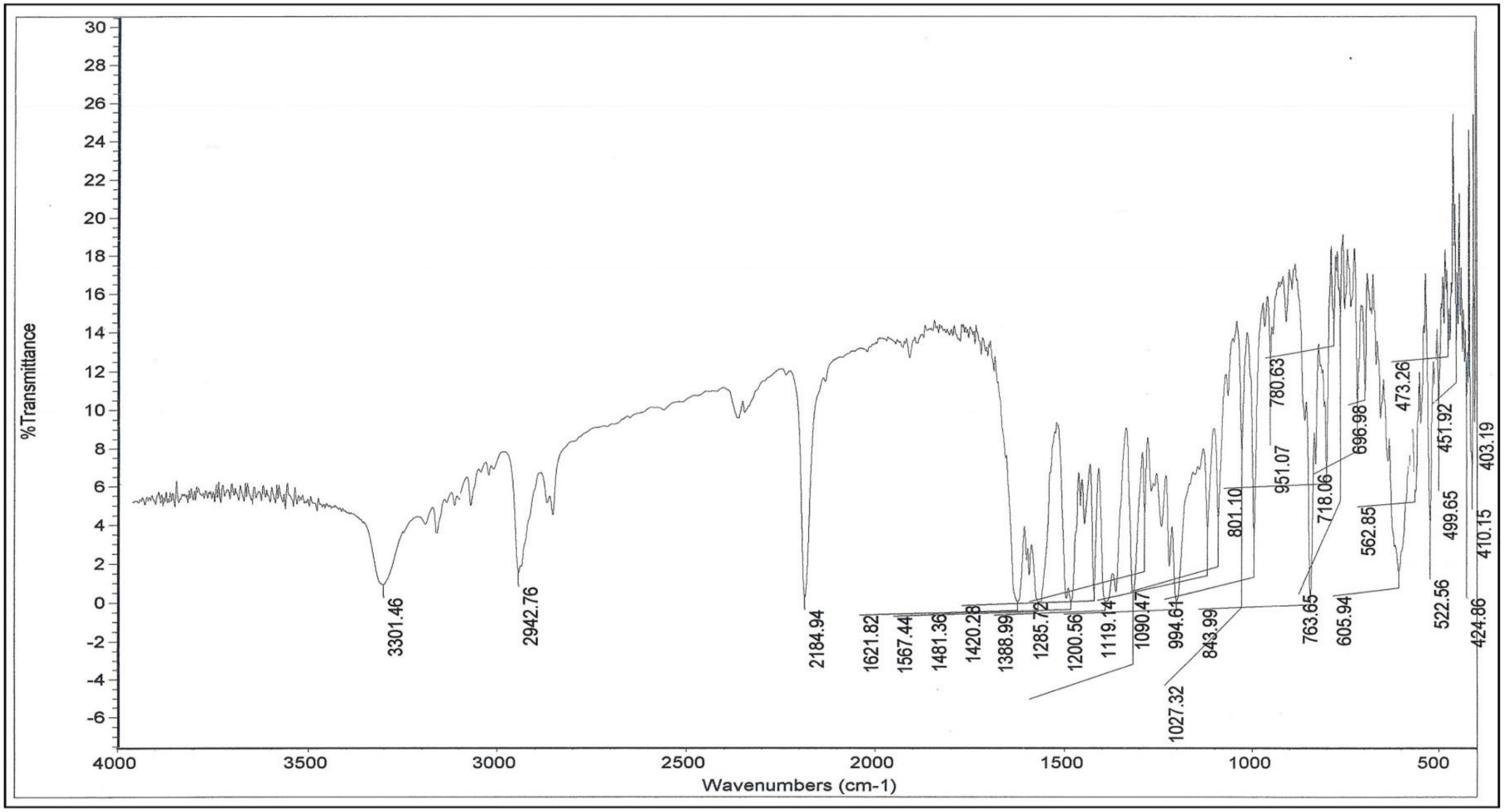

%Transmittance
Wavenumbers (cm-1)
3301.46
2942.76
2184.94
1621.82
1567.44
1481.36
1420.28
1388.99
1285.72
1200.56
1119.14
1090.47
1027.32
994.61
843.99
801.10
951.07
780.63
763.65
718.06
696.98
605.94
562.85
522.56
499.65
473.26
451.92
424.86
410.15
403.19

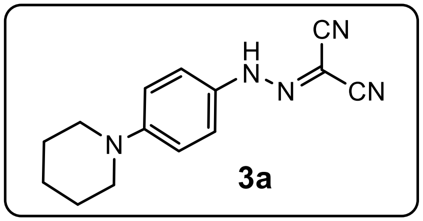
CN
H
N
N
CN
N

3a

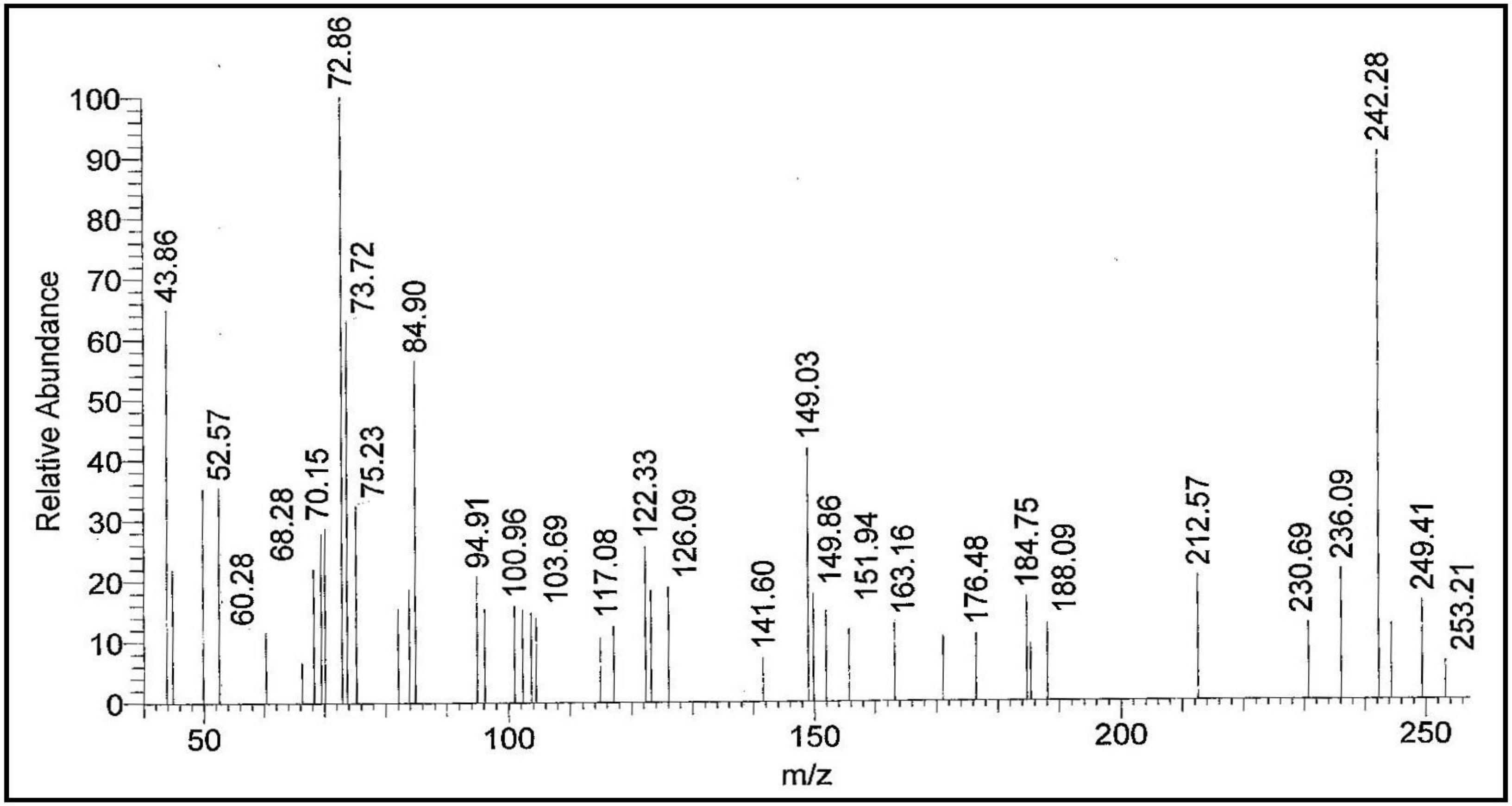
Relative Abundance
m/z
72.86
242.28
43.86
73.72
84.90
149.03
52.57
70.15
75.23
68.28
60.28
94.91
100.96
103.69
117.08
122.33
126.09
141.60
149.86
151.94
163.16
176.48
184.75
188.09
212.57
230.69
236.09
249.41
253.21
0
10
20
30
40
50
60
70
80
90
100
50
100
150
200
250

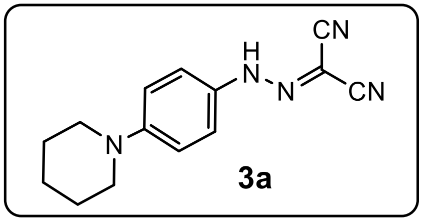

H
N
N
CN
CN
N

3a

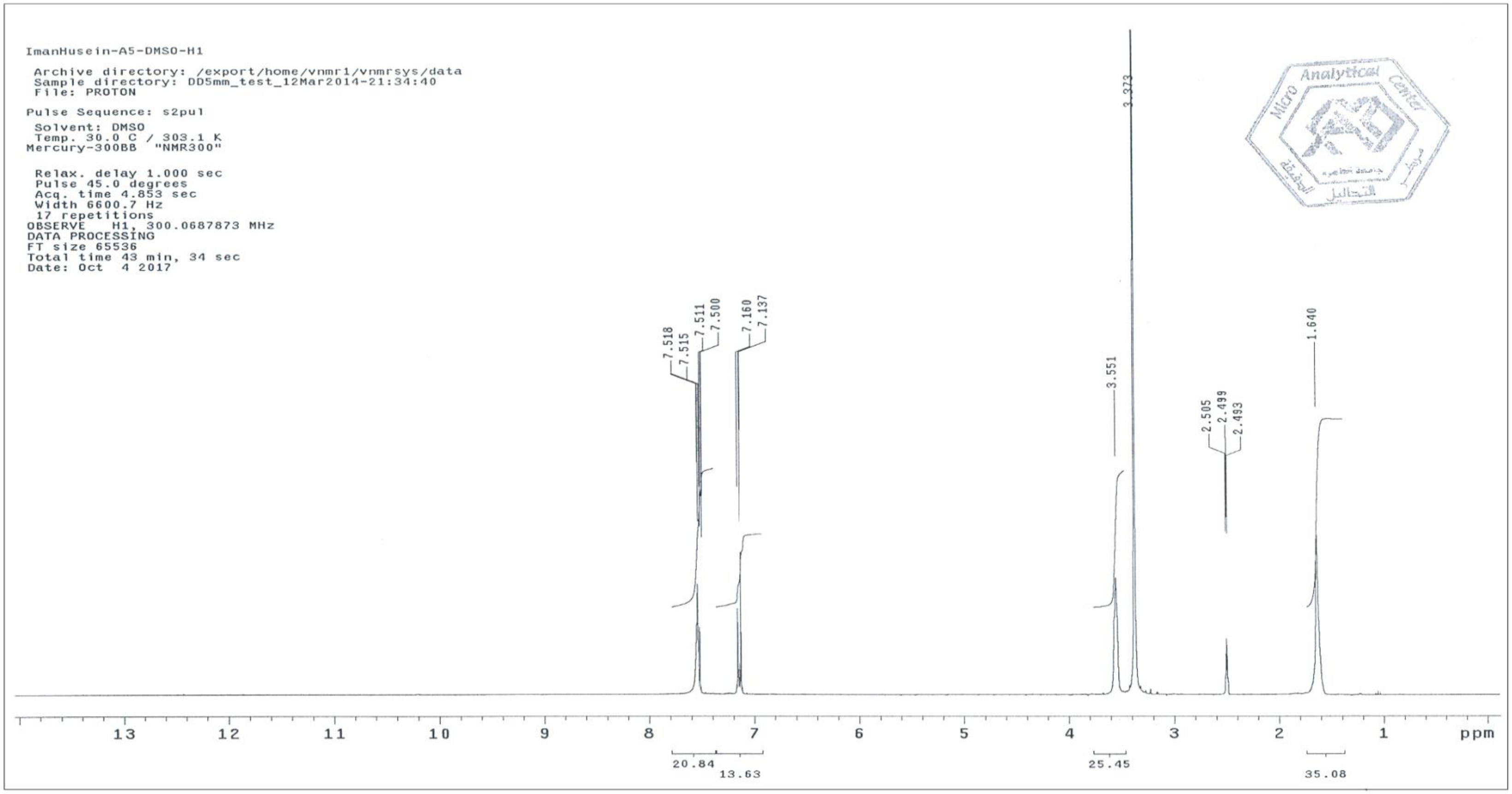

ImanHusein-A5-DMSO-H1
Archive directory: /export/home/vnmr1/vnmrsys/data
Sample directory: DD5mm_test_12Mar2014-21:34:40
File: PROTON
Pulse Sequence: s2pul
Solvent: DMSO
Temp. 30.0 C / 303.1 K
Mercury-300BB "NMR300"
Relax. delay 1.000 sec
Pulse 45.0 degrees
Acq. time 4.853 sec
Width 6600.7 Hz
17 repetitions
OBSERVE H1, 300.0687873 MHz
DATA PROCESSING
FT size 65536
Total time 43 min, 34 sec
Date: Oct 4 2017
7.518
7.515
7.511
7.500
7.160
7.137
3.551
3.373
2.505
2.499
2.493
1.640
13 12 11 10 9 8 7 6 5 4 3 2 1 ppm
20.84
13.63
25.45
35.08

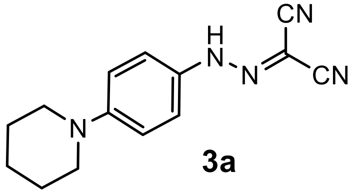
CN
CN
3a

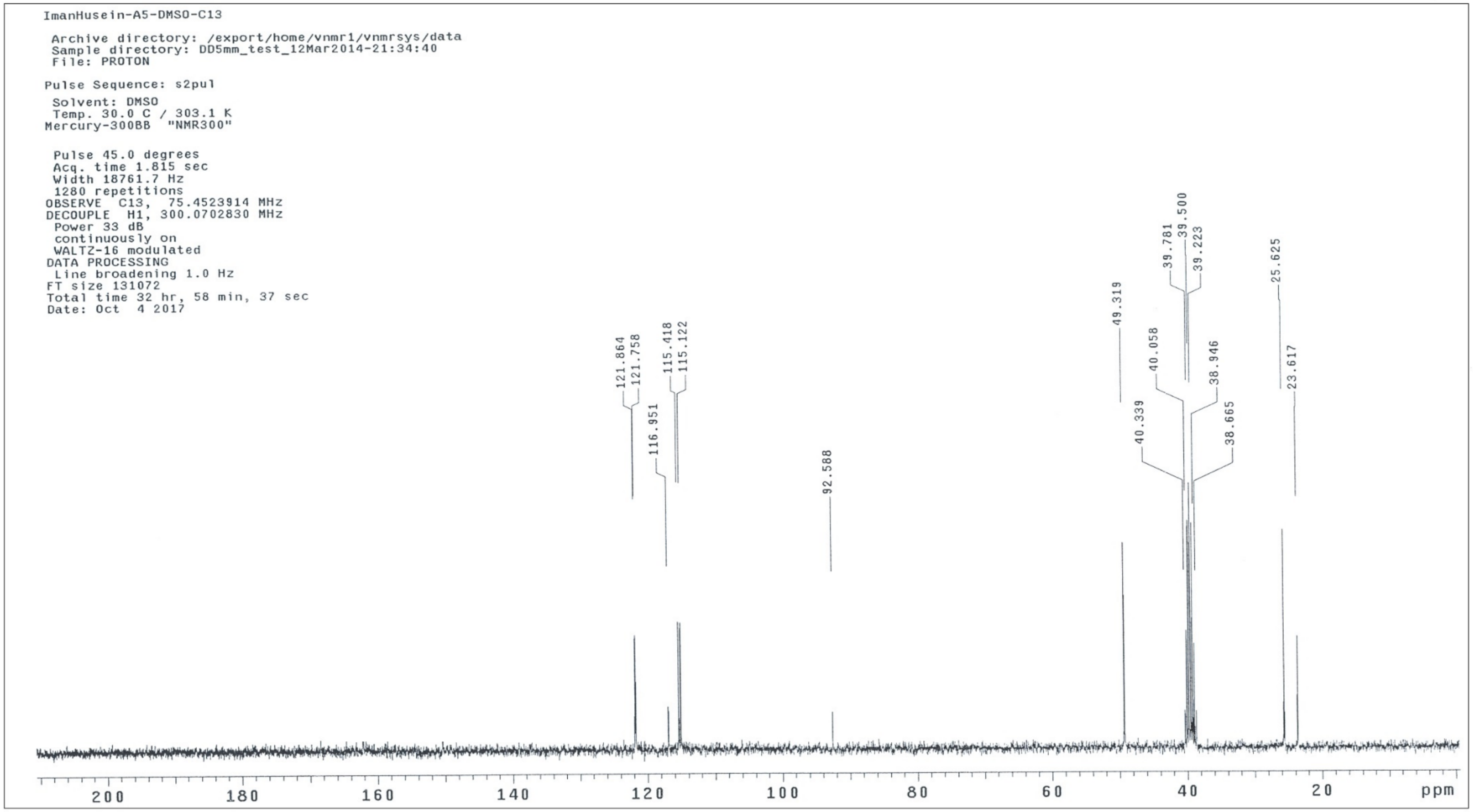
ImanHusein-A5-DMSO-C13
Archive directory: /export/home/vnmr1/vnmrsys/data
Sample directory: DD5mm_test_12Mar2014-21:34:40
File: PROTON
Pulse Sequence: s2pul
Solvent: DMSO
Temp. 30.0 C / 303.1 K
Mercury-300BB "NMR300"
Pulse 45.0 degrees
Acq. time 1.815 sec
Width 18761.7 Hz
1280 repetitions
OBSERVE C13, 75.4523914 MHz
DECOUPLE H1, 300.0702830 MHz
Power 33 dB
continuously on
WALTZ-16 modulated
DATA PROCESSING
Line broadening 1.0 Hz
FT size 131072
Total time 32 hr, 58 min, 37 sec
Date: Oct 4 2017
121.864
121.758
116.951
115.418
115.122
92.588
49.319
40.339
40.058
39.781
39.500
39.223
38.946
38.665
25.625
23.617
200
180
160
140
120
100
80
60
40
20
ppm

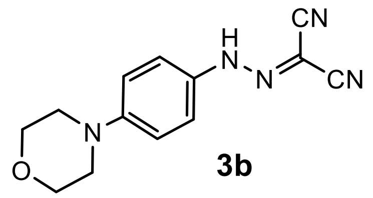
CN
H
N
N
CN
N
O

3b

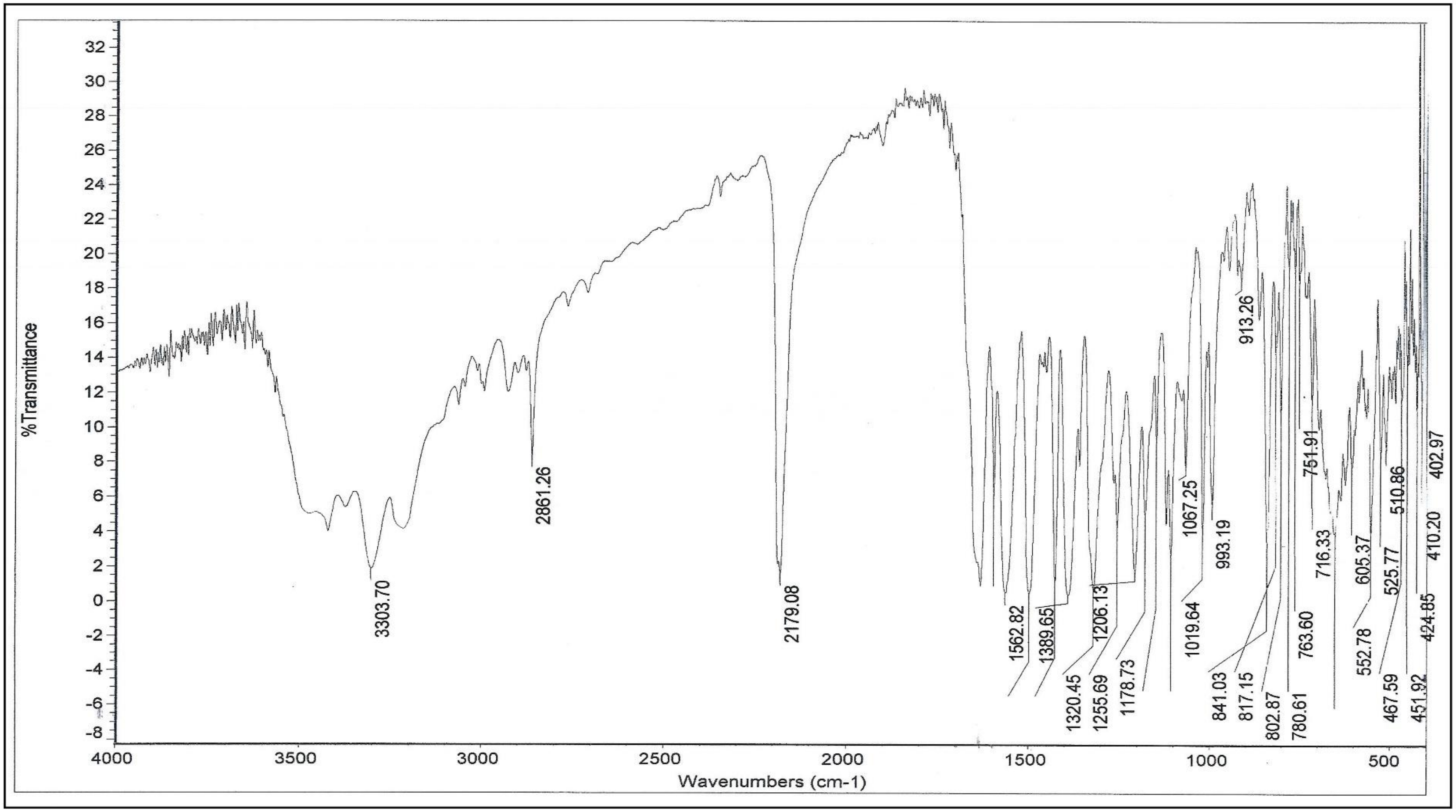
%Transmittance
Wavenumbers (cm-1)
3303.70
2861.26
2179.08
1562.82
1389.65
1320.45
1255.69
1206.13
1178.73
1067.25
1019.64
993.19
913.26
841.03
817.15
802.87
780.61
763.60
751.91
716.33
605.37
552.78
525.77
510.86
467.59
451.92
424.85
410.20
402.97

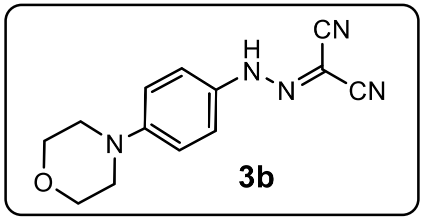

H
N
N
CN
CN
N
O

3b

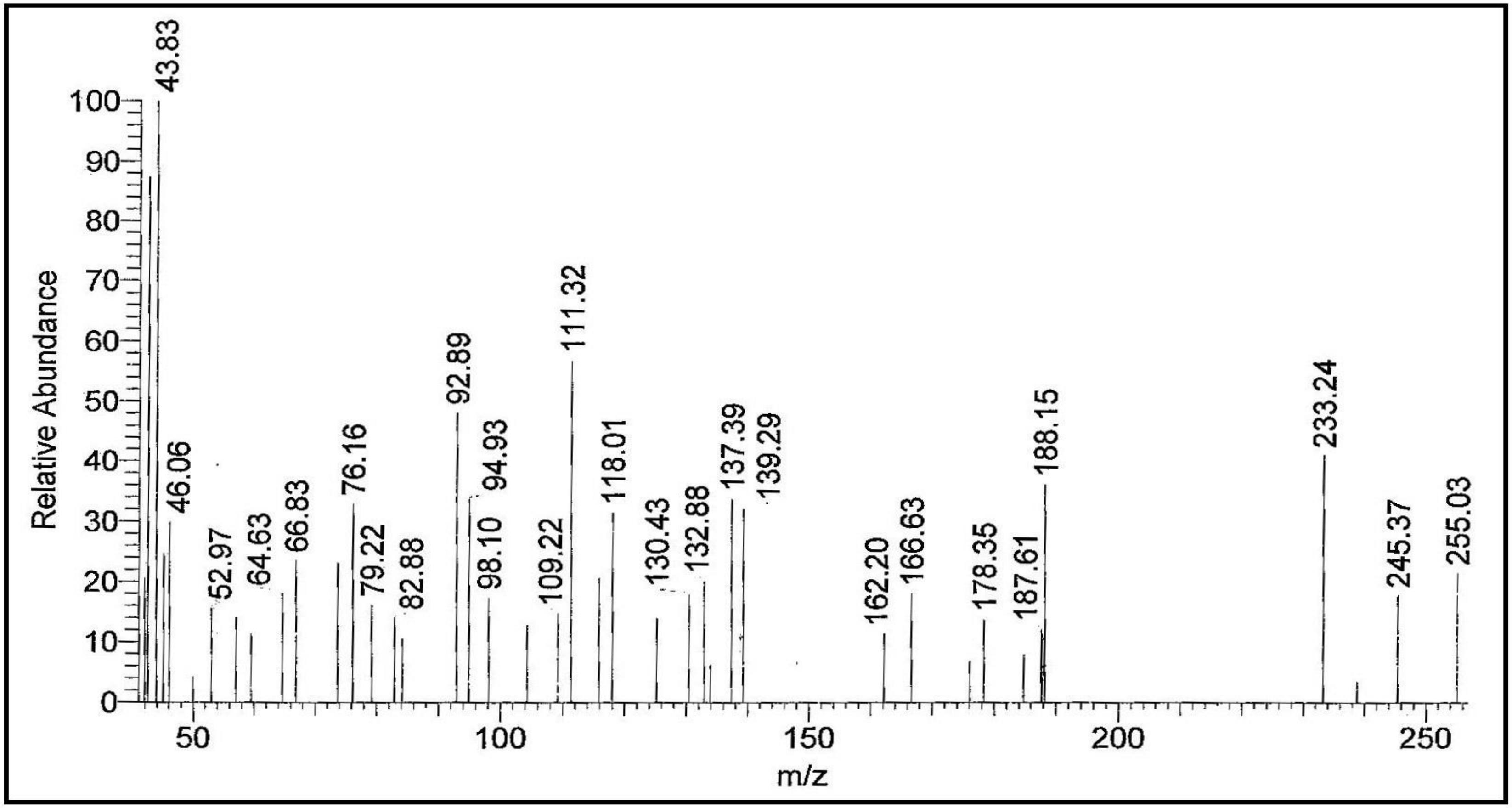

Relative Abundance
m/z
0
10
20
30
40
50
60
70
80
90
100
50
100
150
200
250
43.83
46.06
52.97
64.63
66.83
76.16
79.22
82.88
92.89
94.93
98.10
109.22
111.32
118.01
130.43
132.88
137.39
139.29
162.20
166.63
178.35
187.61
188.15
233.24
245.37
255.03

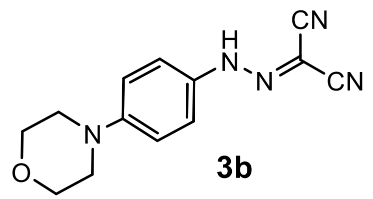

3b

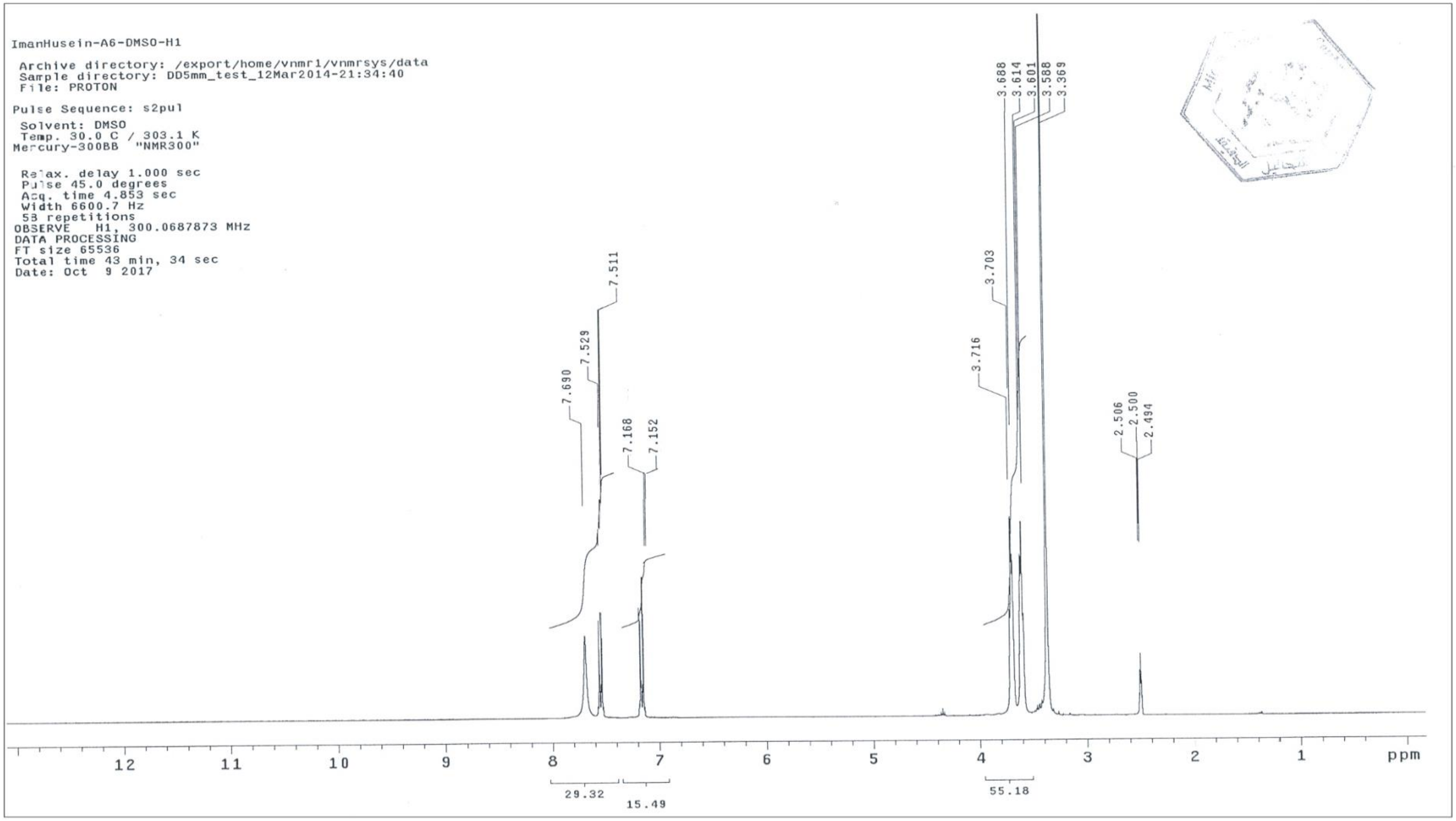
ImanHusein-A6-DMSO-H1
Archive directory: /export/home/vnmr1/vnmrsys/data
Sample directory: DD5mm_test_12Mar2014-21:34:40
File: PROTON
Pulse Sequence: s2pul
Solvent: DMSO
Temp. 30.0 C / 303.1 K
Mercury-300BB "NMR300"
Relax. delay 1.000 sec
Pulse 45.0 degrees
Acq. time 4.853 sec
Width 6600.7 Hz
58 repetitions
OBSERVE H1, 300.0687873 MHz
DATA PROCESSING
FT size 65536
Total time 43 min, 34 sec
Date: Oct 9 2017
7.690
7.529
7.511
7.168
7.152
3.716
3.703
3.688
3.614
3.601
3.588
3.369
2.506
2.500
2.494
29.32
15.49
55.18
ppm

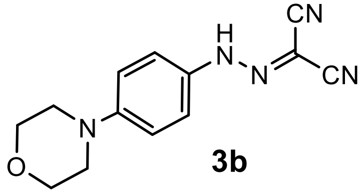
H
N
N
CN
CN
N
O

3b

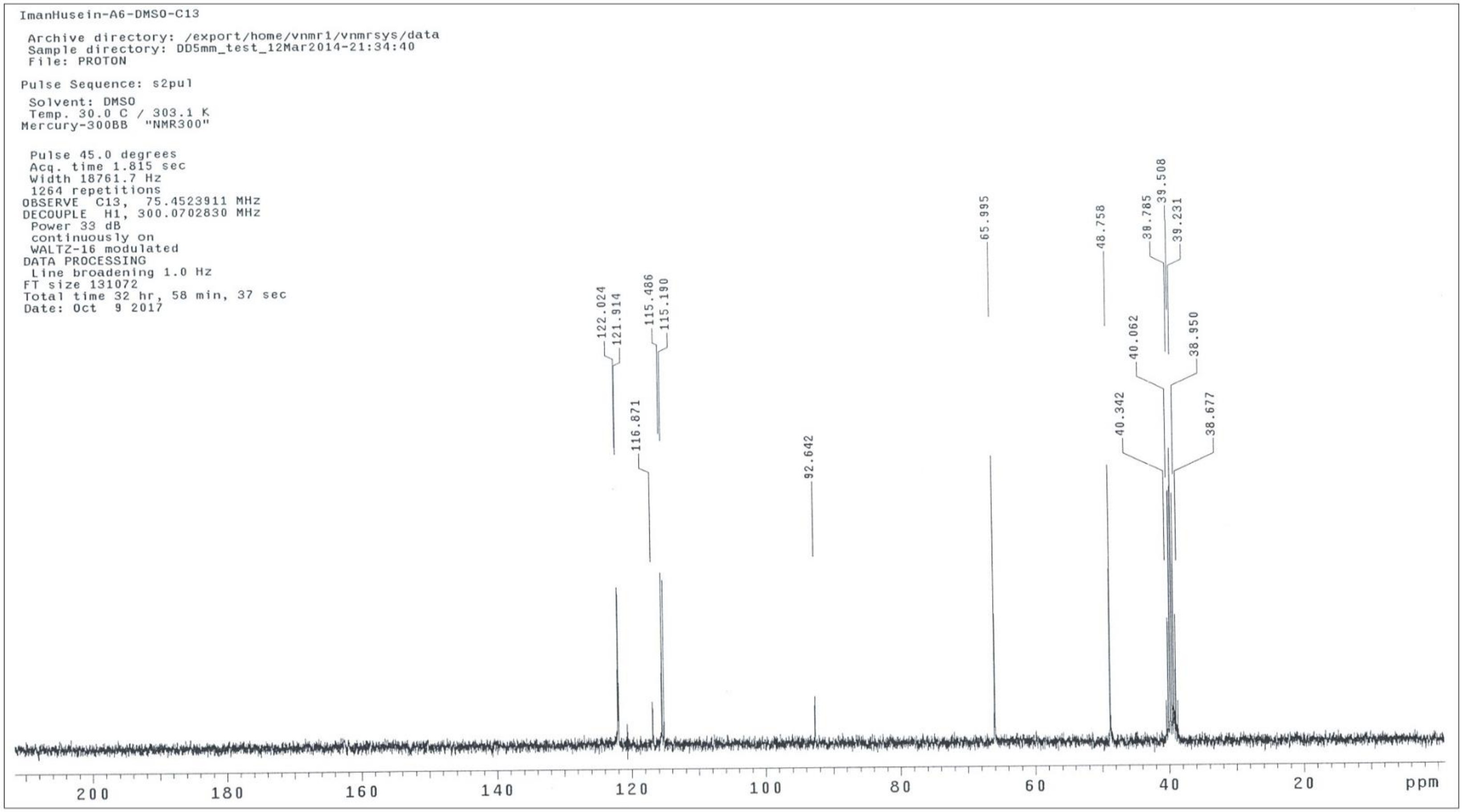
ImanHusein-A6-DMSO-C13
Archive directory: /export/home/vnmr1/vnmrsys/data
Sample directory: DD5mm_test_12Mar2014-21:34:40
File: PROTON
Pulse Sequence: s2pul
Solvent: DMSO
Temp. 30.0 C / 303.1 K
Mercury-300BB "NMR300"
Pulse 45.0 degrees
Acq. time 1.815 sec
Width 18761.7 Hz
1264 repetitions
OBSERVE C13, 75.4523911 MHz
DECOUPLE H1, 300.0702830 MHz
Power 33 dB
continuously on
WALTZ-16 modulated
DATA PROCESSING
Line broadening 1.0 Hz
FT size 131072
Total time 32 hr, 58 min, 37 sec
Date: Oct 9 2017
122.024
121.914
116.871
115.486
115.190
92.642
65.995
48.758
40.342
40.062
39.785
39.508
39.231
38.950
38.677
200
180
160
140
120
100
80
60
40
20
ppm

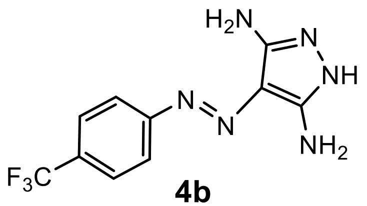

H_2N
N
NH
N
N
NH_2
F_3C

4b

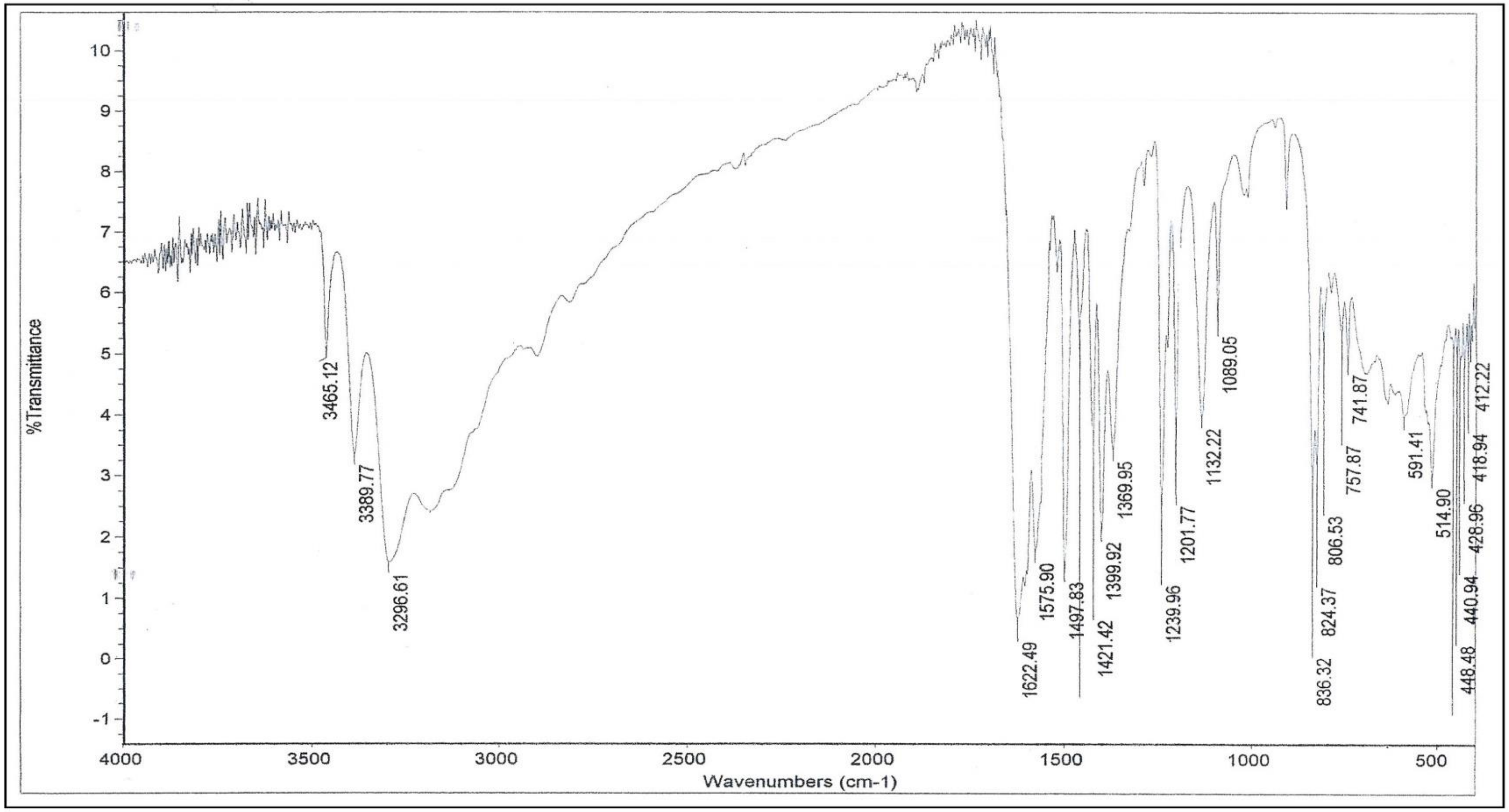

%Transmittance
Wavenumbers (cm-1)
3465.12
3389.77
3296.61
1622.49
1575.90
1497.83
1421.42
1399.92
1369.95
1239.96
1201.77
1132.22
1089.05
836.32
824.37
806.53
757.87
741.87
591.41
514.90
448.48
440.94
428.96
418.94
412.22

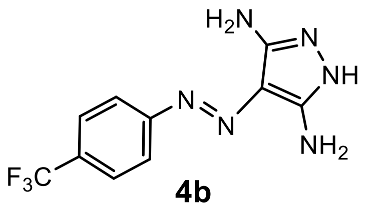

H2N
N
NH
N
N
NH2
F3C

4b

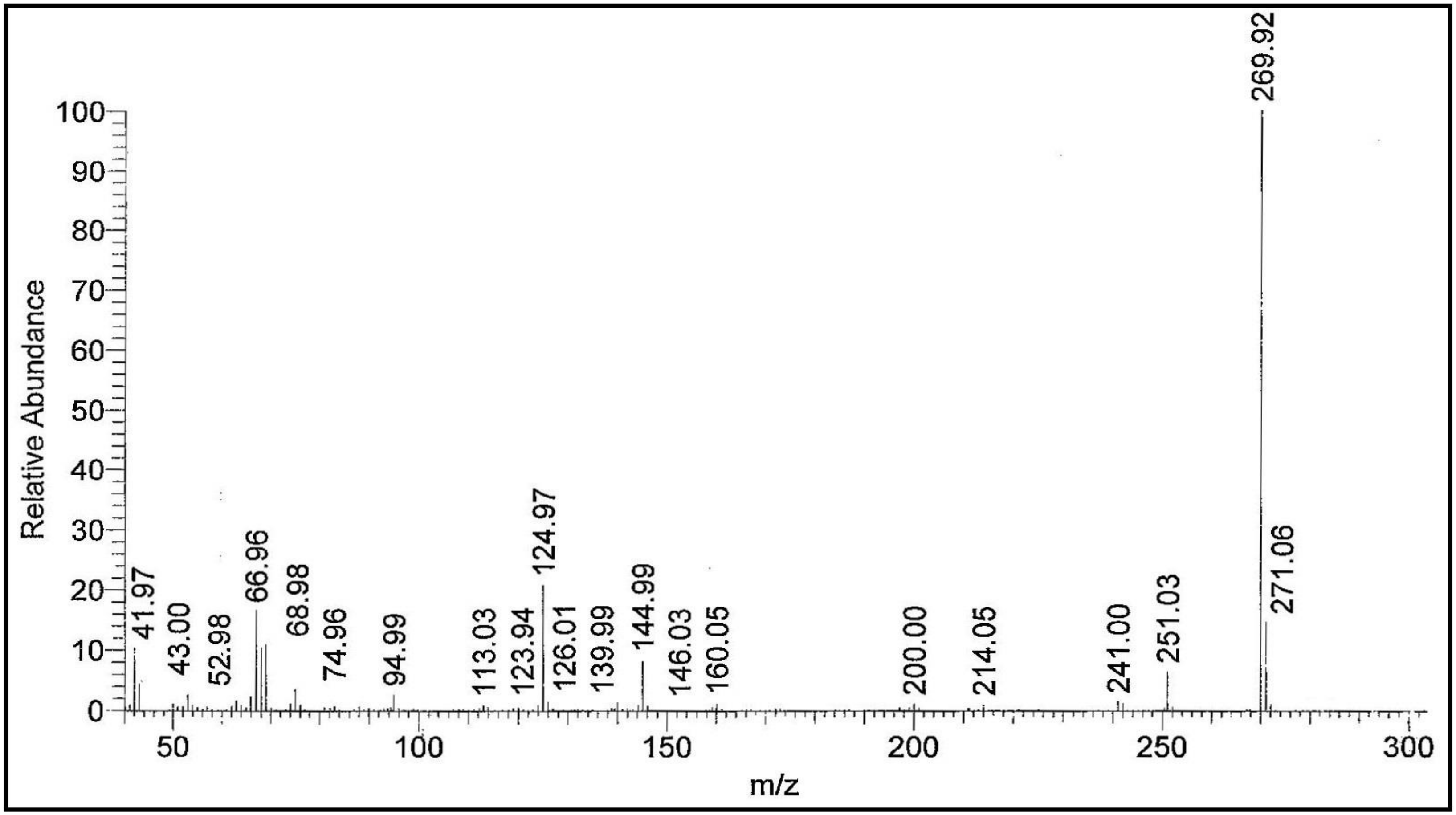

Relative Abundance
m/z
269.92
271.06
251.03
241.00
214.05
200.00
160.05
146.03
144.99
139.99
126.01
124.97
123.94
113.03
94.99
74.96
68.98
66.96
52.98
43.00
41.97

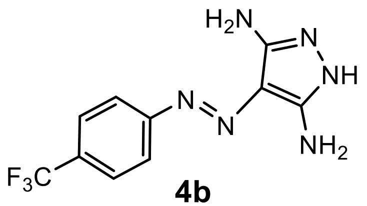

4b

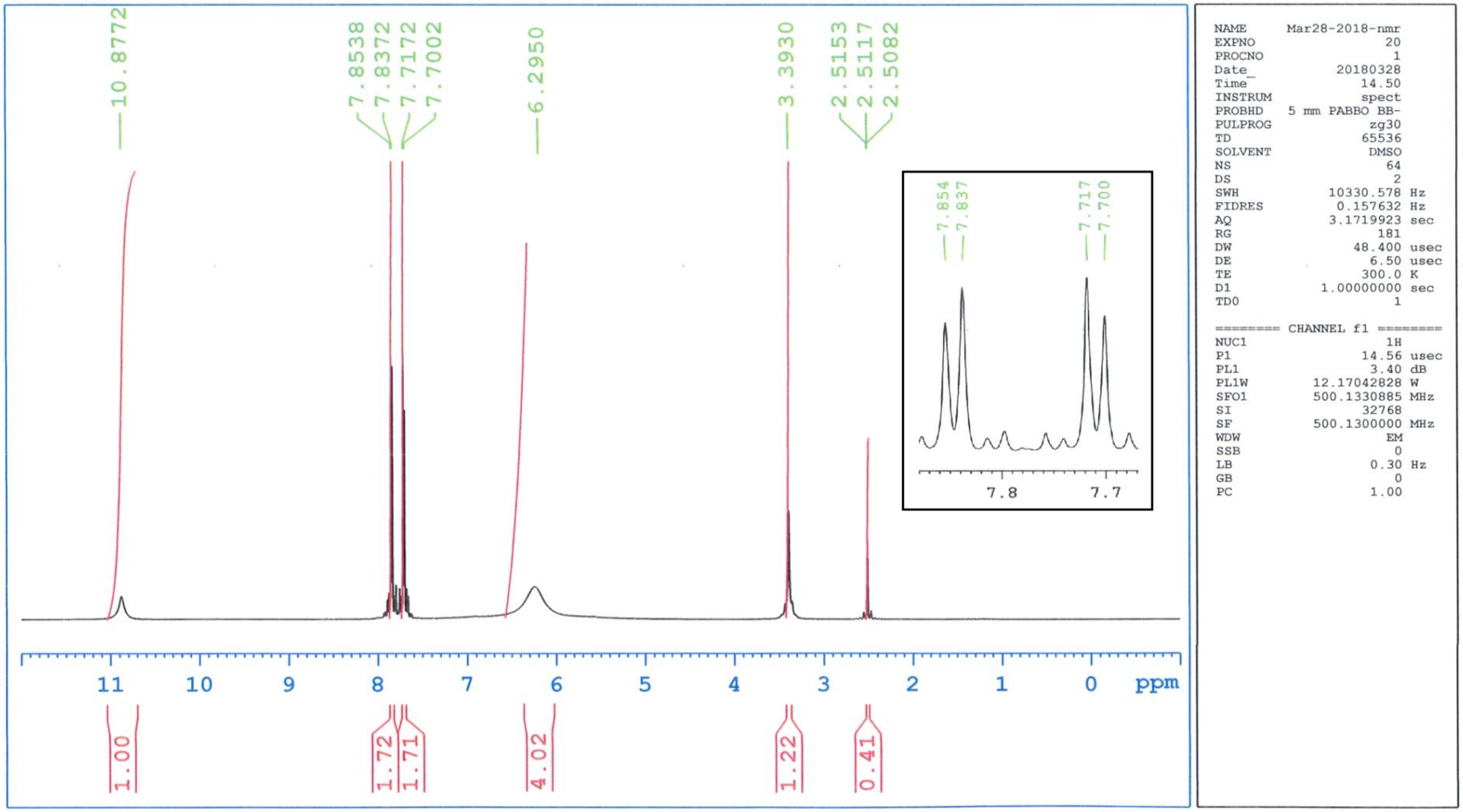
10.8772
7.8538
7.8372
7.7172
7.7002
6.2950
3.3930
2.5153
2.5117
2.5082
7.854
7.837
7.717
7.700
7.8
7.7
11
10
9
8
7
6
5
4
3
2
1
0
ppm
1.00
1.72
1.71
4.02
1.22
0.41
NAME Mar28-2018-nmr
EXPNO 20
PROCNO 1
Date_ 20180328
Time 14.50
INSTRUM spect
PROBHD 5 mm PABBO BB-
PULPROG zg30
TD 65536
SOLVENT DMSO
NS 64
DS 2
SWH 10330.578 Hz
FIDRES 0.157632 Hz
AQ 3.1719923 sec
RG 181
DW 48.400 usec
DE 6.50 usec
TE 300.0 K
D1 1.00000000 sec
TD0 1
======== CHANNEL f1 ========
NUC1 1H
P1 14.56 usec
PL1 3.40 dB
PL1W 12.17042828 W
SFO1 500.1330885 MHz
SI 32768
SF 500.1300000 MHz
WDW EM
SSB 0
LB 0.30 Hz
GB 0
PC 1.00

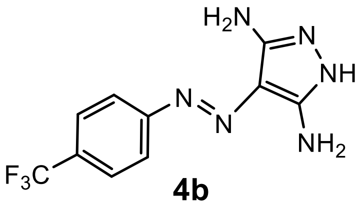
H2N
N
NH
N
N
NH2
F3C

4b

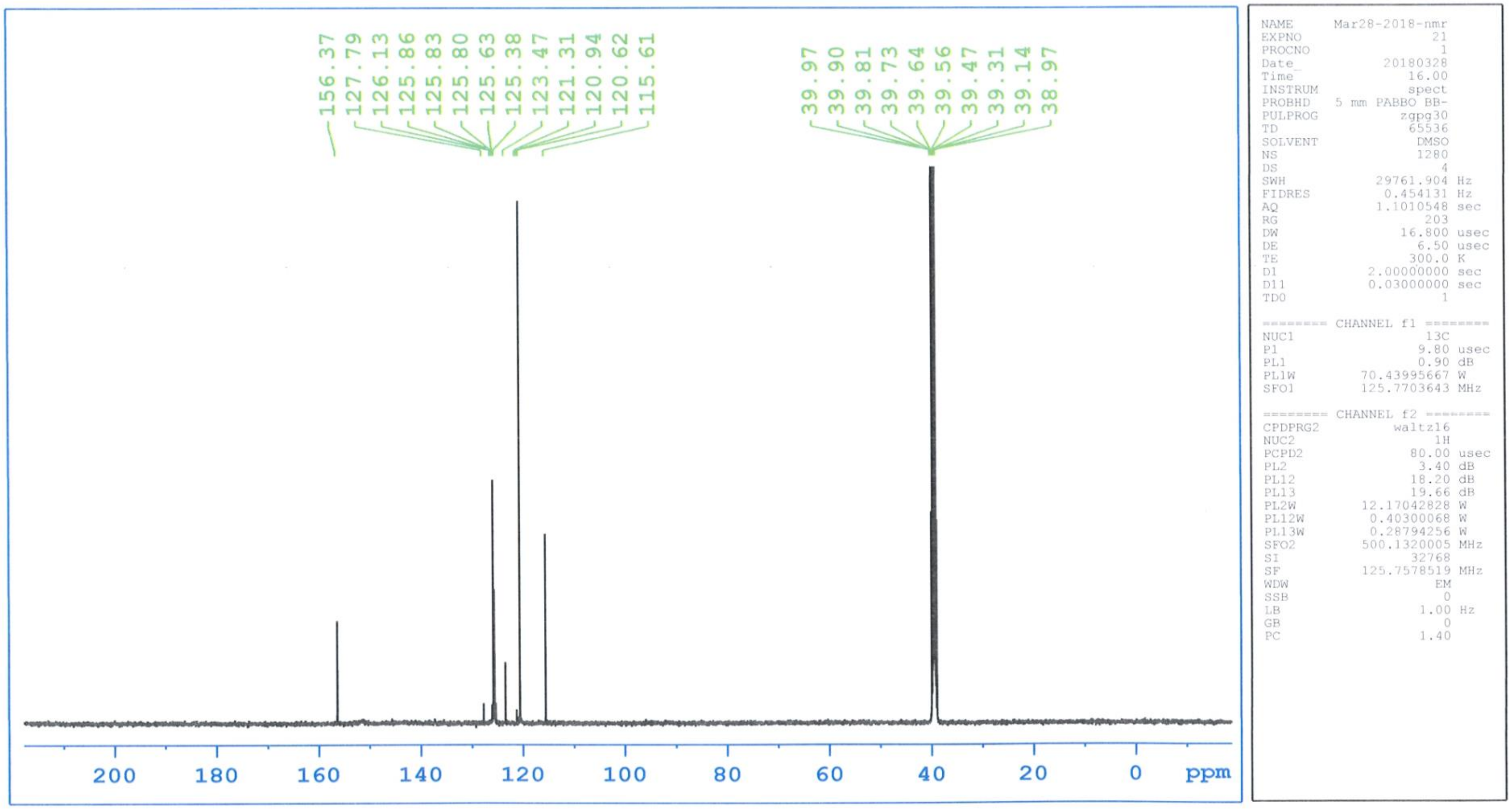
156.37
127.79
126.13
125.86
125.83
125.80
125.63
125.38
123.47
121.31
120.94
120.62
115.61
39.97
39.90
39.81
39.73
39.64
39.56
39.47
39.31
39.14
38.97
200
180
160
140
120
100
80
60
40
20
0
ppm
NAME Mar28-2018-nmr
EXPNO 21
PROCNO 1
Date_ 20180328
Time 16.00
INSTRUM spect
PROBHD 5 mm PABBO BB-
PULPROG zgpg30
TD 65536
SOLVENT DMSO
NS 1280
DS 4
SWH 29761.904 Hz
FIDRES 0.454131 Hz
AQ 1.1010548 sec
RG 203
DW 16.800 usec
DE 6.50 usec
TE 300.0 K
D1 2.00000000 sec
D11 0.03000000 sec
TD0 1
======== CHANNEL f1 ========
NUC1 13C
P1 9.80 usec
PL1 0.90 dB
PL1W 70.43995667 W
SFO1 125.7703643 MHz
======== CHANNEL f2 ========
CPDPRG2 waltz16
NUC2 1H
PCPD2 80.00 usec
PL2 3.40 dB
PL12 18.20 dB
PL13 19.66 dB
PL2W 12.17042828 W
PL12W 0.40300068 W
PL13W 0.28794256 W
SFO2 500.1320005 MHz
SI 32768
SF 125.7578519 MHz
WDW EM
SSB 0
LB 1.00 Hz
GB 0
PC 1.40

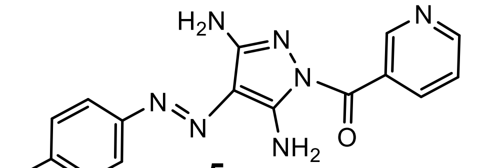
H2N
N
N
N
N
N
NH2
O
F
N

5a

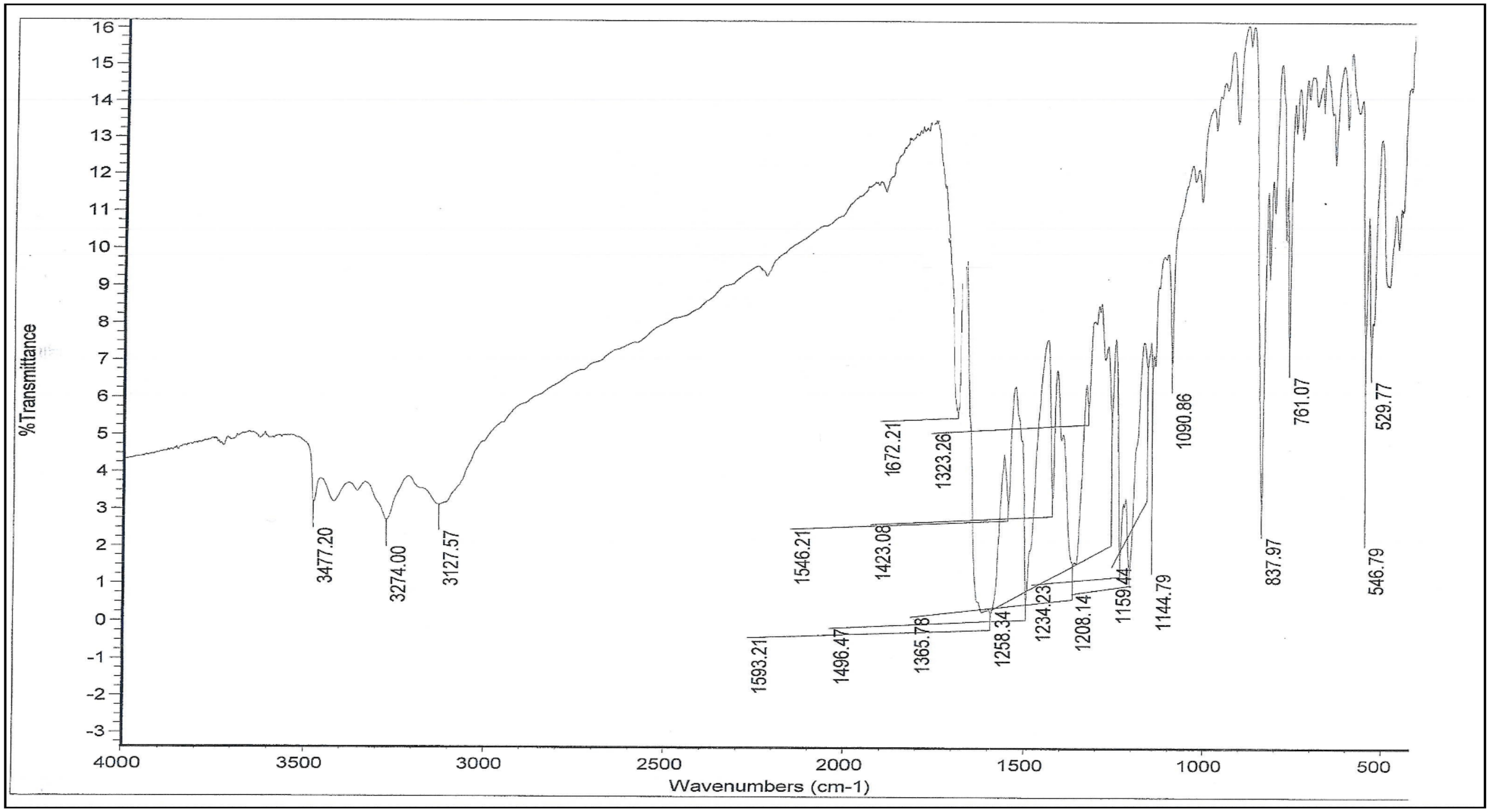
%Transmittance
Wavenumbers (cm-1)
3477.20
3274.00
3127.57
1672.21
1323.26
1546.21
1423.08
1593.21
1496.47
1365.78
1258.34
1234.23
1208.14
1159.44
1144.79
1090.86
837.97
761.07
546.79
529.77

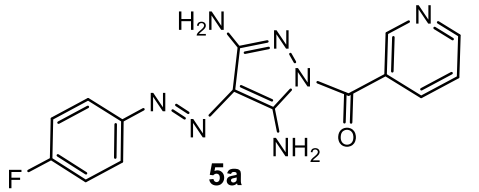

5a

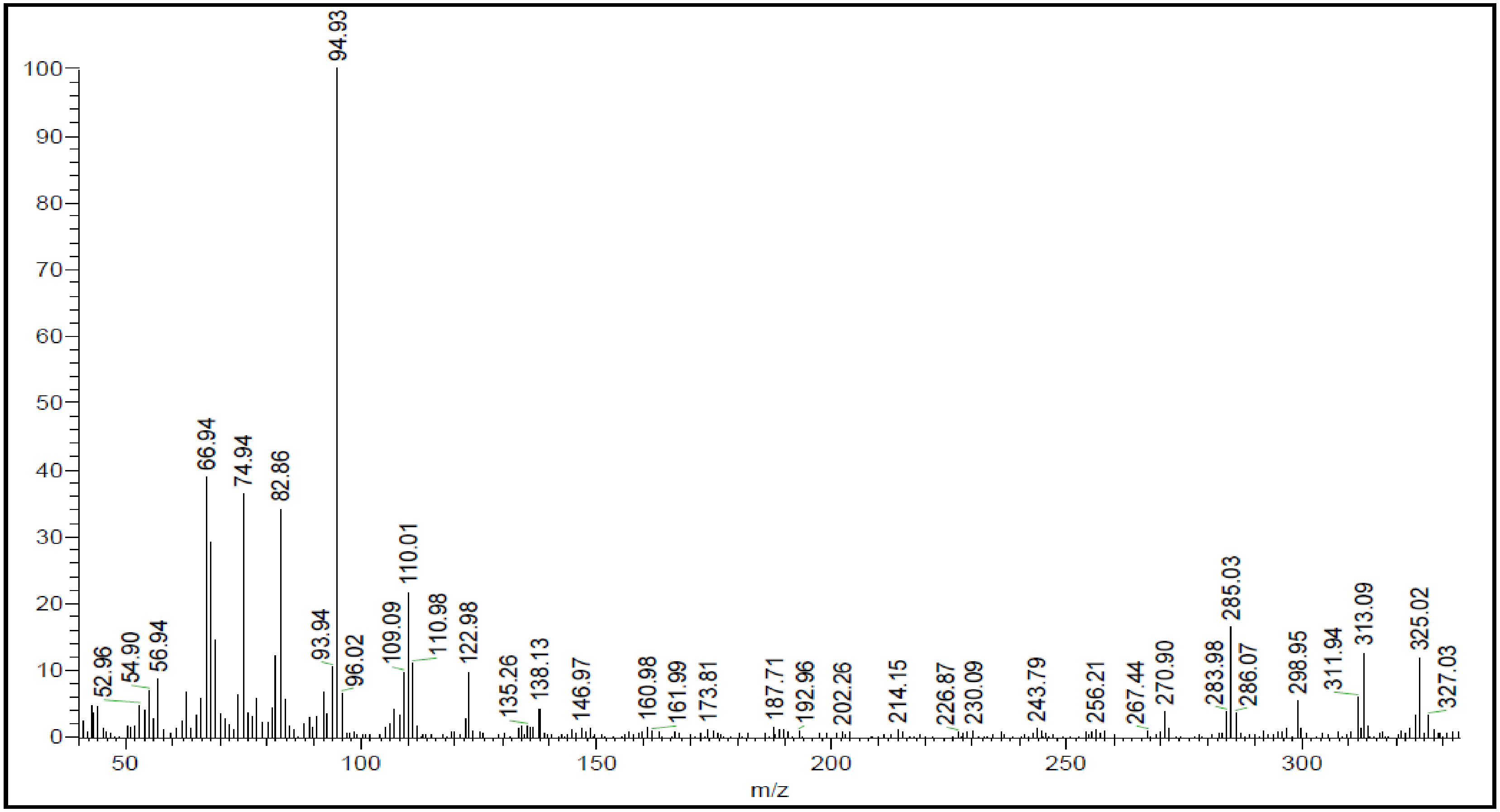
m/z
94.93
66.94
74.94
82.86
110.01
110.98
122.98
109.09
96.02
93.94
52.96
54.90
56.94
135.26
138.13
146.97
160.98
161.99
173.81
187.71
192.96
202.26
214.15
226.87
230.09
243.79
256.21
267.44
270.90
283.98
285.03
286.07
298.95
311.94
313.09
325.02
327.03

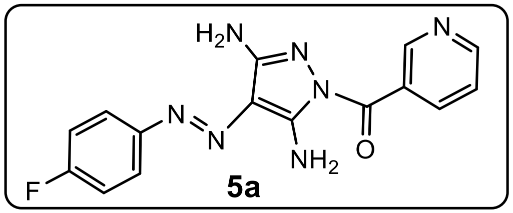
H2N
N
N
N
N
N
NH2
O
F

5a

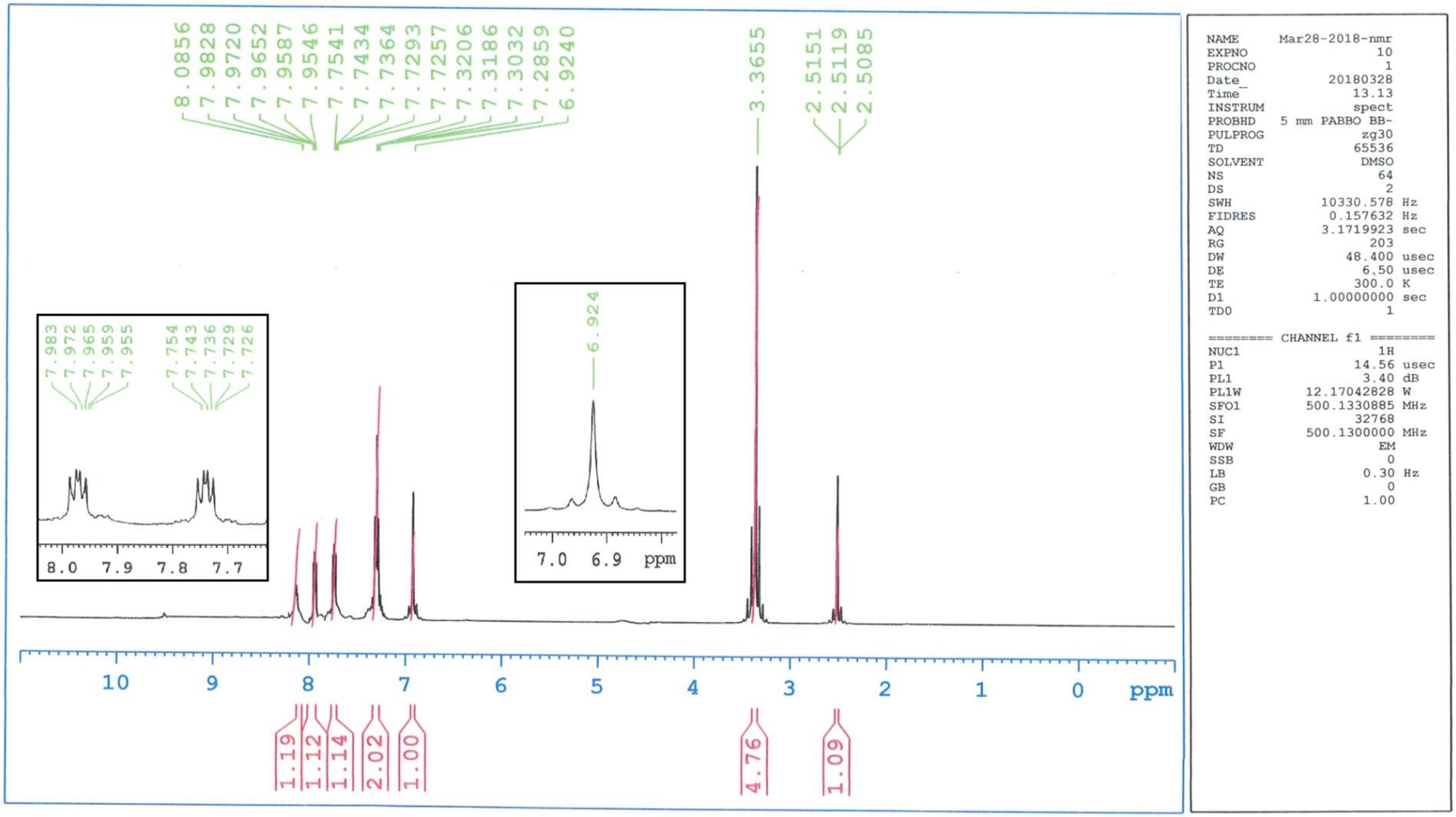
8.0856
7.9828
7.9720
7.9652
7.9587
7.9546
7.7541
7.7434
7.7364
7.7293
7.7257
7.3206
7.3186
7.3032
7.2859
6.9240
3.3655
2.5151
2.5119
2.5085
7.983
7.972
7.965
7.959
7.955
7.754
7.743
7.736
7.729
7.726
8.0 7.9 7.8 7.7
6.924
7.0 6.9 ppm
10 9 8 7 6 5 4 3 2 1 0 ppm
1.19
1.12
1.14
2.02
1.00
4.76
1.09
NAME Mar28-2018-nmr
EXPNO 10
PROCNO 1
Date_ 20180328
Time 13.13
INSTRUM spect
PROBHD 5 mm PABBO BB-
PULPROG zg30
TD 65536
SOLVENT DMSO
NS 64
DS 2
SWH 10330.578 Hz
FIDRES 0.157632 Hz
AQ 3.1719923 sec
RG 203
DW 48.400 usec
DE 6.50 usec
TE 300.0 K
D1 1.00000000 sec
TD0 1
======== CHANNEL f1 ========
NUC1 1H
P1 14.56 usec
PL1 3.40 dB
PL1W 12.17042828 W
SFO1 500.1330885 MHz
SI 32768
SF 500.1300000 MHz
WDW EM
SSB 0
LB 0.30 Hz
GB 0
PC 1.00

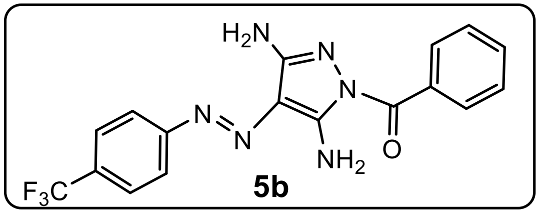

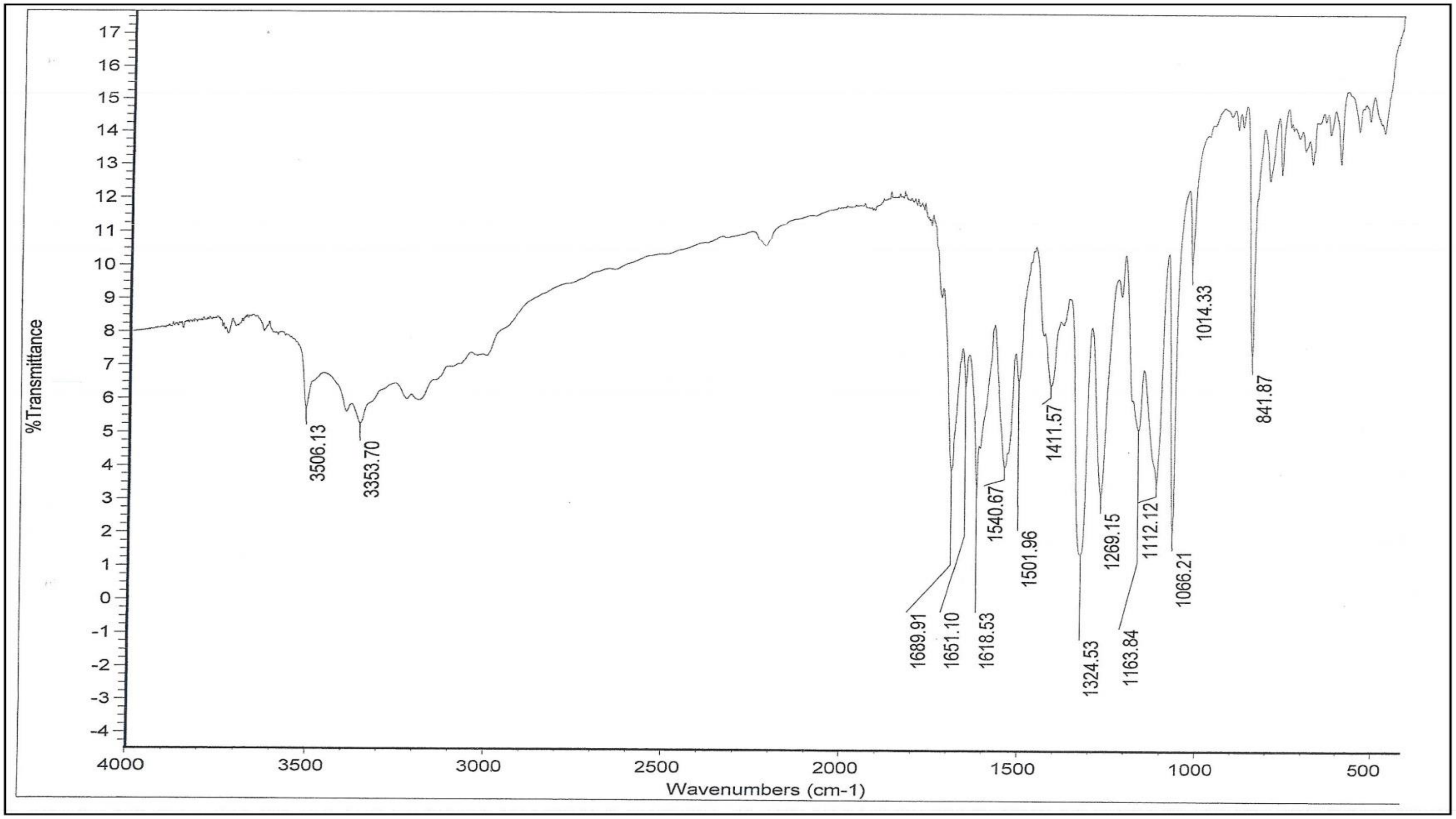
%Transmittance
Wavenumbers (cm-1)
3506.13
3353.70
1689.91
1651.10
1618.53
1540.67
1501.96
1411.57
1324.53
1269.15
1163.84
1112.12
1066.21
1014.33
841.87

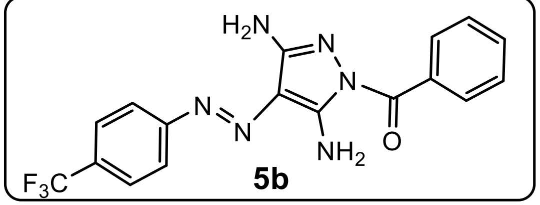
H2N
N
N
N
N
NH2
O
F3C

5b

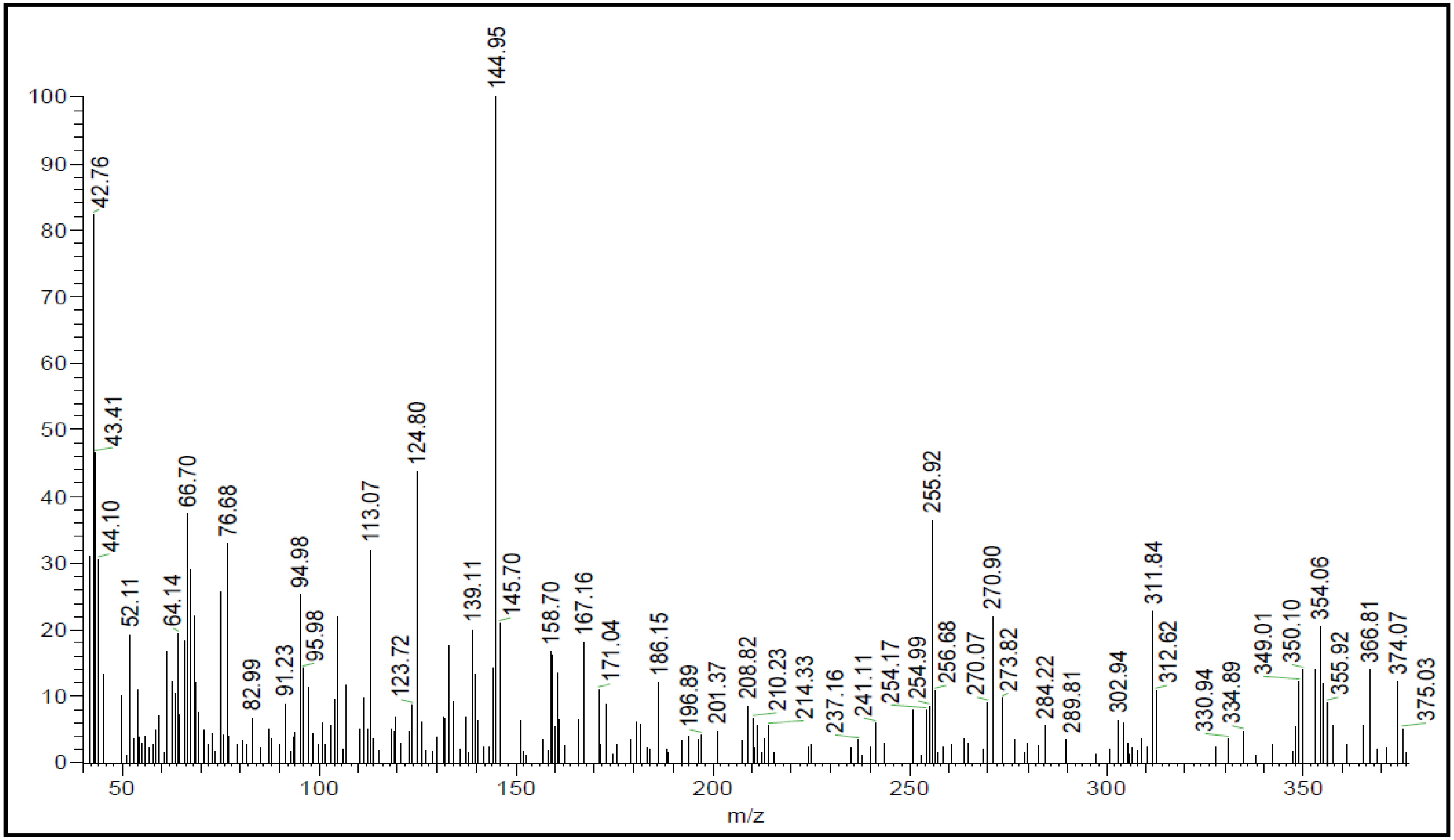
144.95
42.76
43.41
44.10
52.11
64.14
66.70
76.68
82.99
91.23
94.98
95.98
113.07
123.72
124.80
139.11
145.70
158.70
167.16
171.04
186.15
196.89
201.37
208.82
210.23
214.33
237.16
241.11
254.17
254.99
255.92
256.68
270.07
270.90
273.82
284.22
289.81
302.94
311.84
312.62
330.94
334.89
349.01
350.10
354.06
355.92
366.81
374.07
375.03
100
90
80
70
60
50
40
30
20
10
0
50
100
150
200
250
300
350
m/z

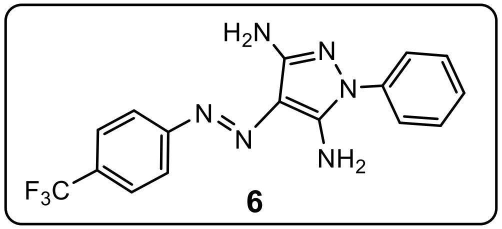
H2N
N
N
N
N
NH2
F3C
6

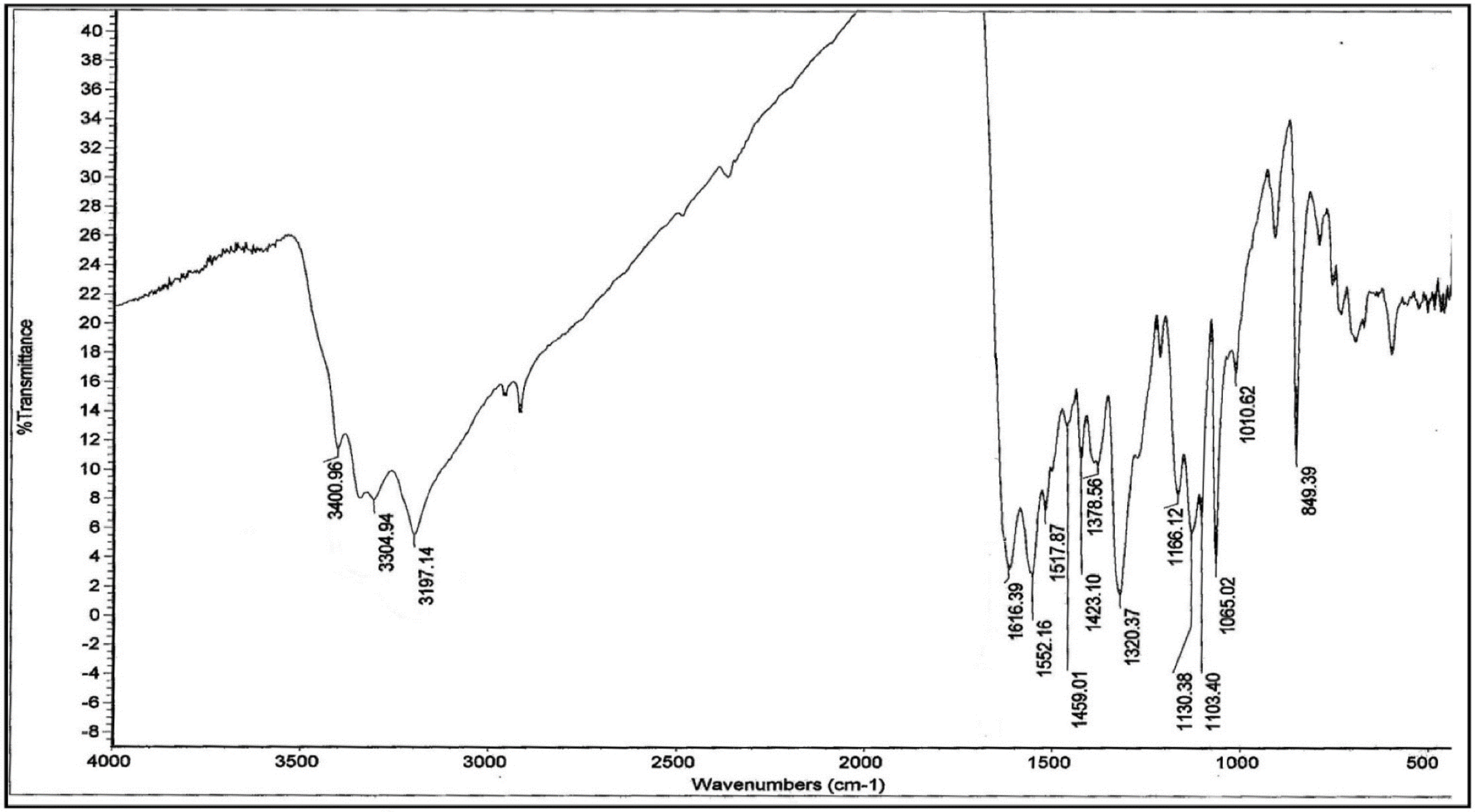
%Transmittance
Wavenumbers (cm-1)
3400.96
3304.94
3197.14
1616.39
1552.16
1517.87
1459.01
1423.10
1378.56
1320.37
1166.12
1130.38
1103.40
1065.02
1010.62
849.39

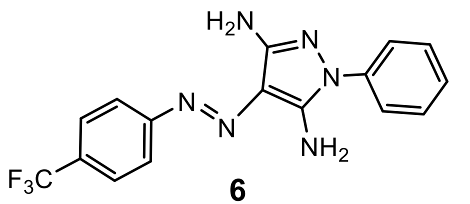
H2N
N
N
N
N
NH2
F3C

6

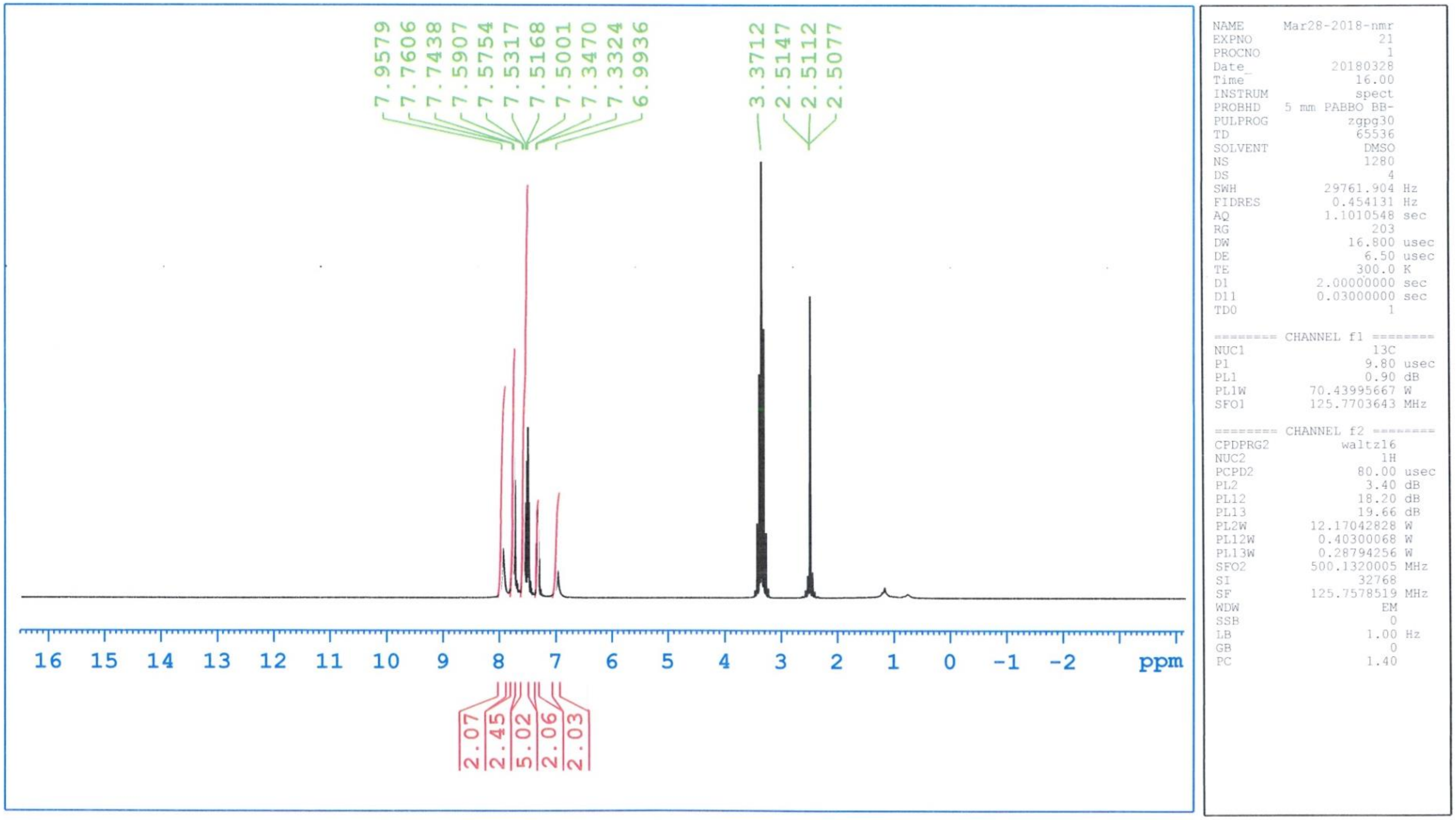
7.9579
7.7606
7.7438
7.5907
7.5754
7.5317
7.5168
7.5001
7.3470
7.3324
6.9936
3.3712
2.5147
2.5112
2.5077
16 15 14 13 12 11 10 9 8 7 6 5 4 3 2 1 0 -1 -2 ppm
2.07
2.45
5.02
2.06
2.03
NAME Mar28-2018-nmr
EXPNO 21
PROCNO 1
Date_ 20180328
Time 16.00
INSTRUM spect
PROBHD 5 mm PABBO BB-
PULPROG zgpg30
TD 65536
SOLVENT DMSO
NS 1280
DS 4
SWH 29761.904 Hz
FIDRES 0.454131 Hz
AQ 1.1010548 sec
RG 203
DW 16.800 usec
DE 6.50 usec
TE 300.0 K
D1 2.00000000 sec
D11 0.03000000 sec
TD0 1
======== CHANNEL f1 ========
NUC1 13C
P1 9.80 usec
PL1 0.90 dB
PL1W 70.43995667 W
SFO1 125.7703643 MHz
======== CHANNEL f2 ========
CPDPRG2 waltz16
NUC2 1H
PCPD2 80.00 usec
PL2 3.40 dB
PL12 18.20 dB
PL13 19.66 dB
PL2W 12.17042828 W
PL12W 0.40300068 W
PL13W 0.28794256 W
SFO2 500.1320005 MHz
SI 32768
SF 125.7578519 MHz
WDW EM
SSB 0
LB 1.00 Hz
GB 0
PC 1.40

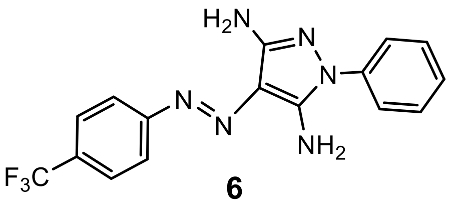

6

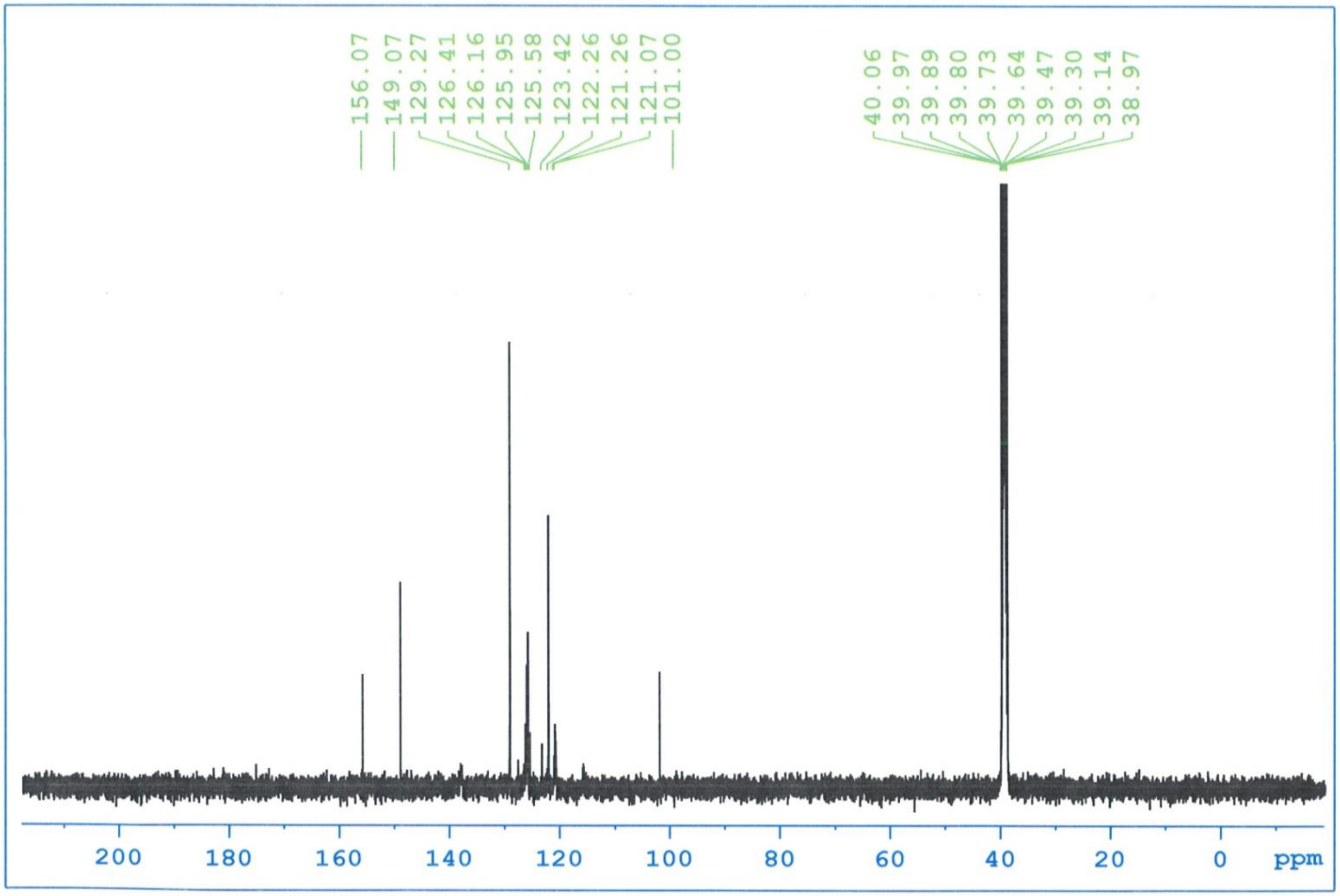

NAME	Mar28-2018-nmr	
EXPNO	21	
PROCNO	1	
Date_	20180328	
Time	16.00	
INSTRUM	spect	
PROBHD	5 mm PABBO BB-	
PULPROG	zgpg30	
TD	65536	
SOLVENT	DMSO	
NS	1280	
DS	4	
SWH	29761.904	Hz
FIDRES	0.454131	Hz
AQ	1.1010548	sec
RG	203	
DW	16.800	usec
DE	6.50	usec
TE	300.0	K
D1	2.00000000	sec
D11	0.03000000	sec
TD0	1	
======== CHANNEL f1 ========		
NUC1	13C	
P1	9.80	usec
PL1	0.90	dB
PL1W	70.43995667	W
SFO1	125.7703643	MHz
======== CHANNEL f2 ========		
CPDPRG2	waltz16	
NUC2	1H	
PCPD2	80.00	usec
PL2	3.40	dB
PL12	18.20	dB
PL13	19.66	dB
PL2W	12.17042828	W
PL12W	0.40300068	W
PL13W	0.28794256	W
SFO2	500.1320005	MHz
SI	32768	
SF	125.7578519	MHz
WDW	EM	
SSB	0	
LB	1.00	Hz
GB	0	
PC	1.40	

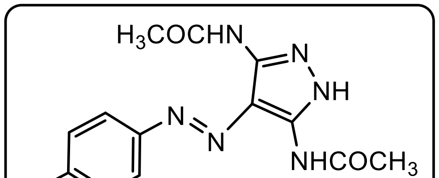
H3COCHN
N
NH
N
N
NHCOCH3
F

7a

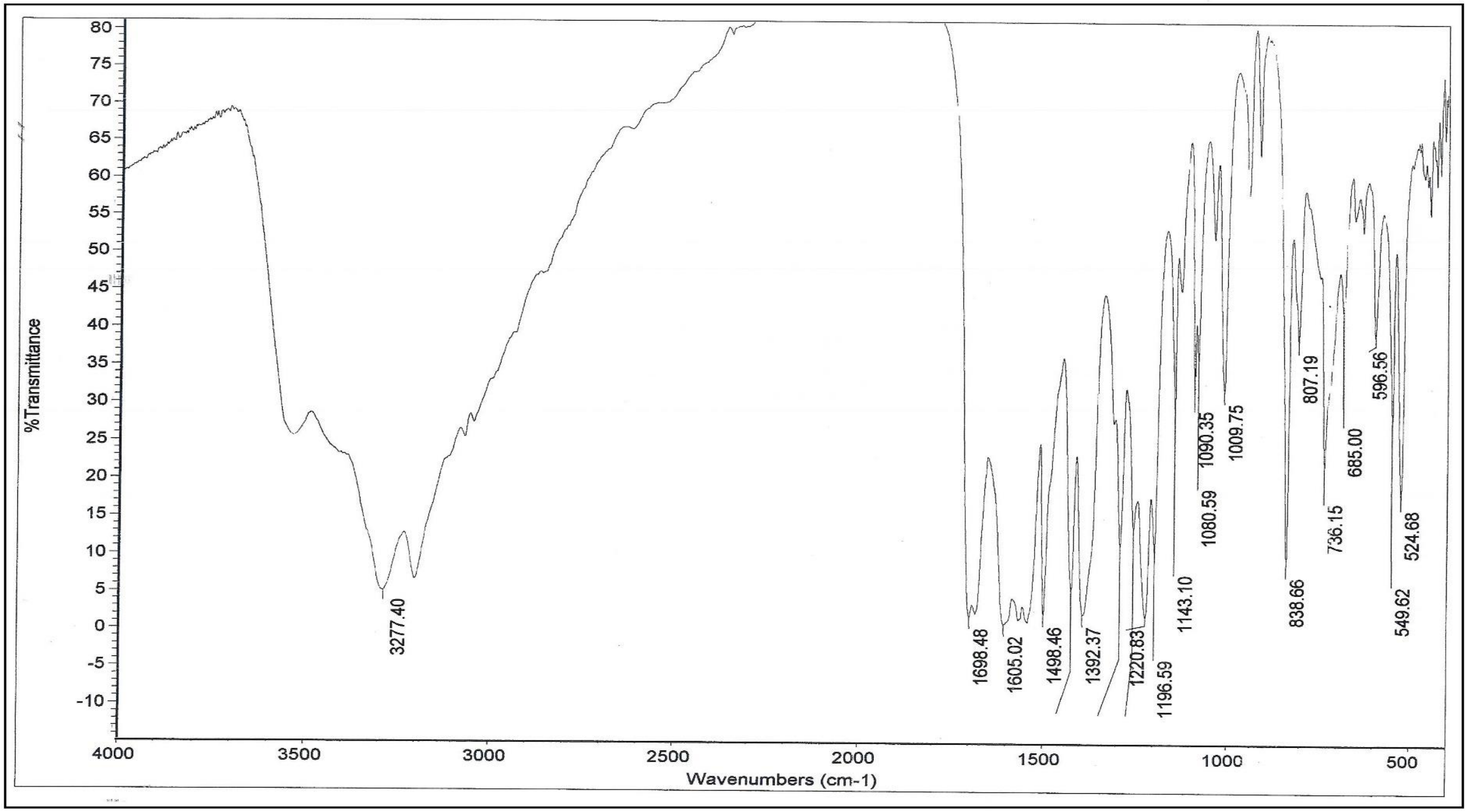
%Transmittance
Wavenumbers (cm-1)
3277.40
1698.48
1605.02
1498.46
1392.37
1220.83
1196.59
1143.10
1090.35
1080.59
1009.75
838.66
807.19
736.15
685.00
596.56
549.62
524.68

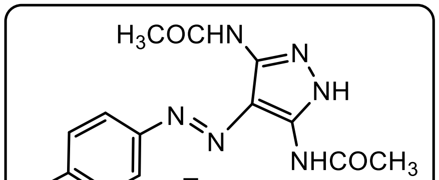

7a

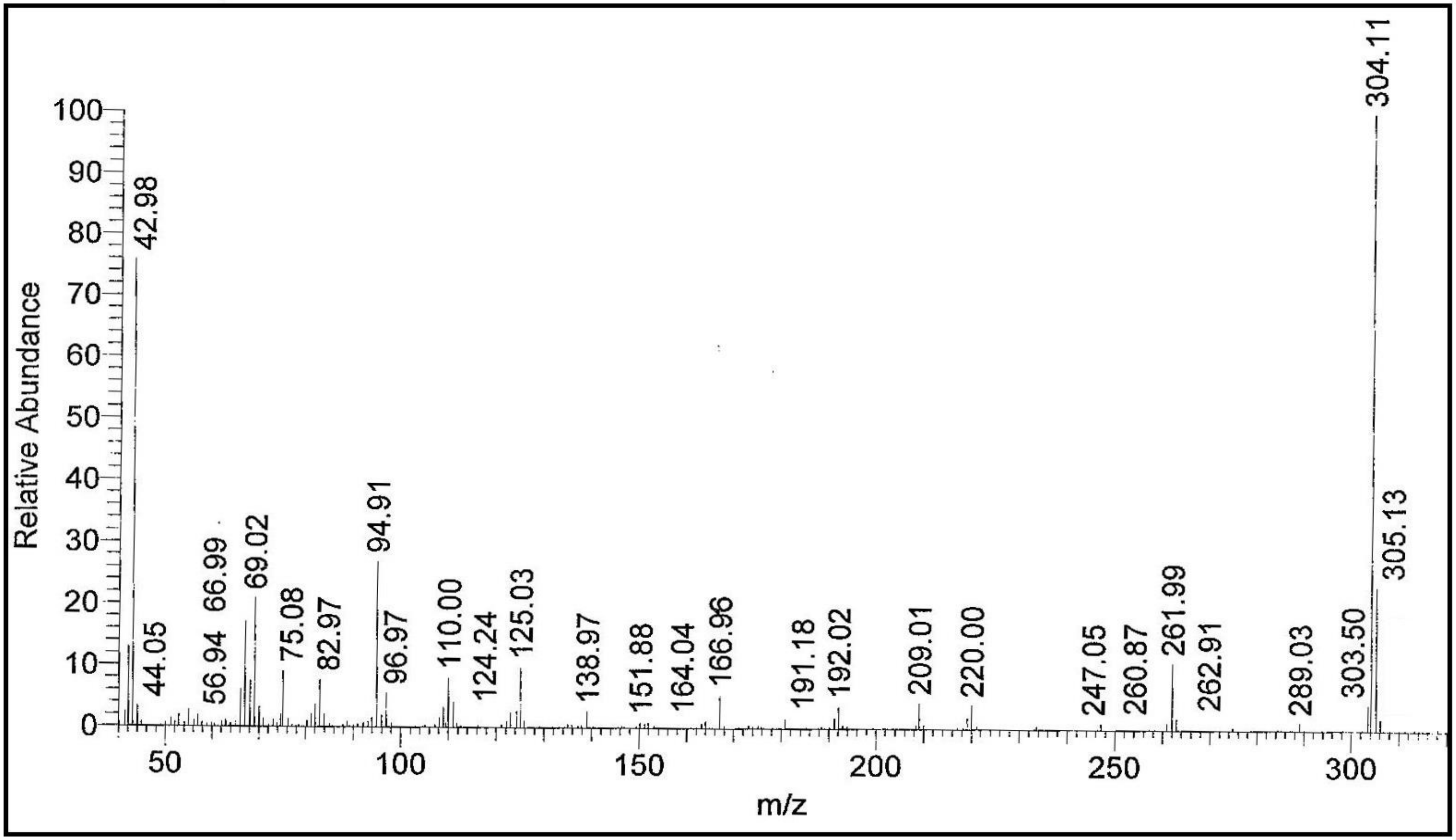
Relative Abundance
m/z
304.11
42.98
305.13
94.91
69.02
66.99
56.94
44.05
75.08
82.97
96.97
110.00
124.24
125.03
138.97
151.88
164.04
166.96
191.18
192.02
209.01
220.00
247.05
260.87
261.99
262.91
289.03
303.50

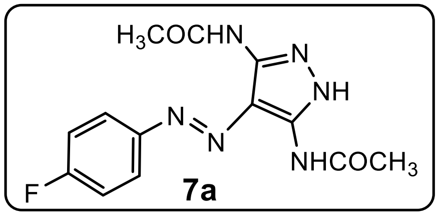
H3COCHN
N
NH
N
N
NHCOCH3
F

7a

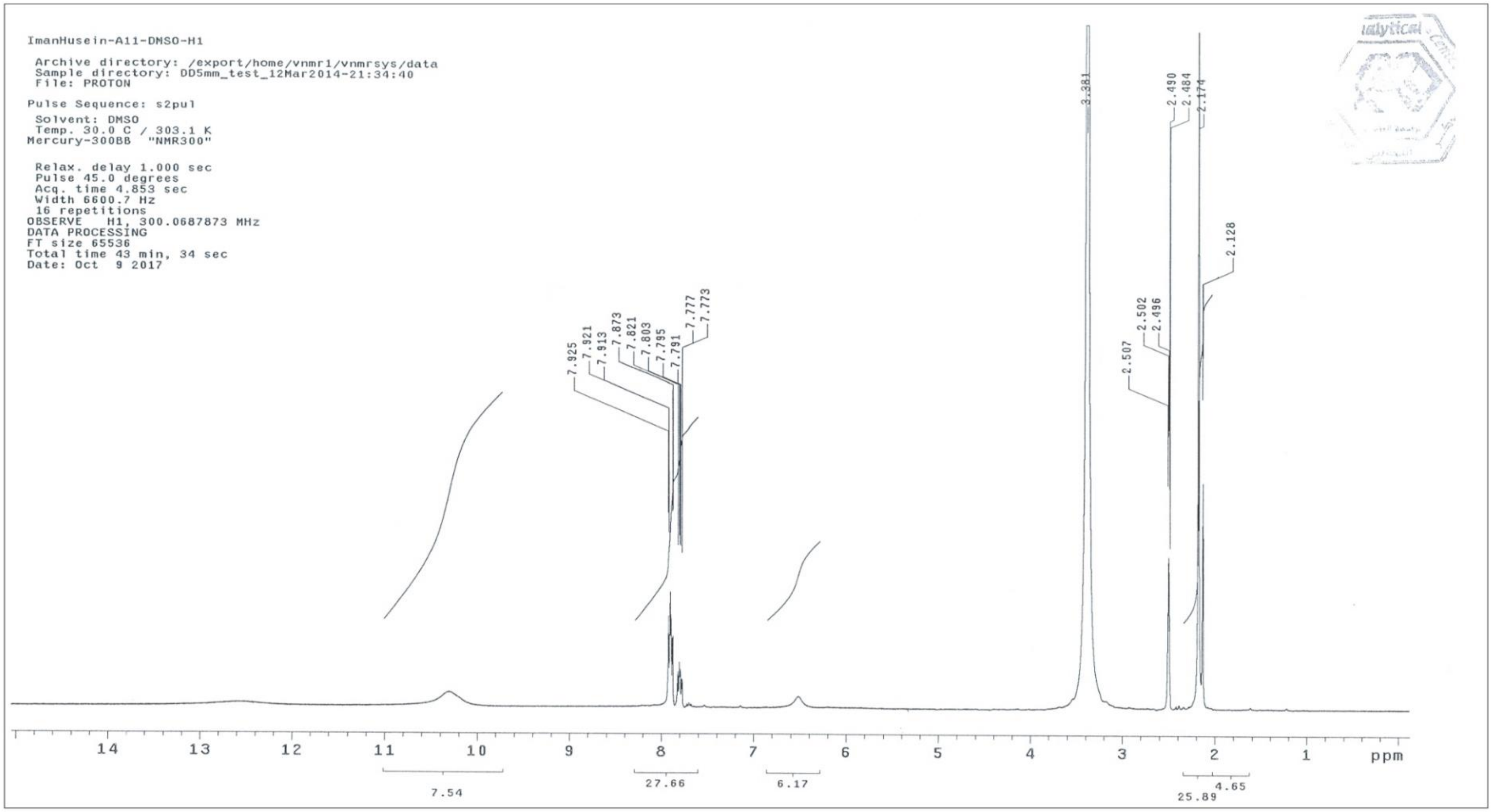
ImanHusein-A11-DMSO-H1
Archive directory: /export/home/vnmr1/vnmrsys/data
Sample directory: DD5mm_test_12Mar2014-21:34:40
File: PROTON
Pulse Sequence: s2pul
Solvent: DMSO
Temp. 30.0 C / 303.1 K
Mercury-300BB "NMR300"
Relax. delay 1.000 sec
Pulse 45.0 degrees
Acq. time 4.853 sec
Width 6600.7 Hz
16 repetitions
OBSERVE H1, 300.0687873 MHz
DATA PROCESSING
FT size 65536
Total time 43 min, 34 sec
Date: Oct 9 2017
7.925
7.921
7.913
7.873
7.821
7.803
7.795
7.791
7.777
7.773
3.381
2.507
2.502
2.496
2.490
2.484
2.174
2.128
ppm
7.54
27.66
6.17
25.89
4.65

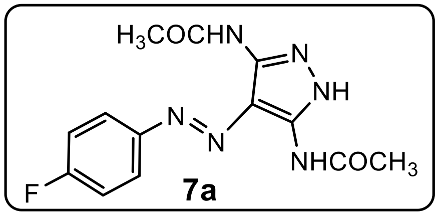
H3COCHN
N
NH
N
N
NHCOCH3
F
7a

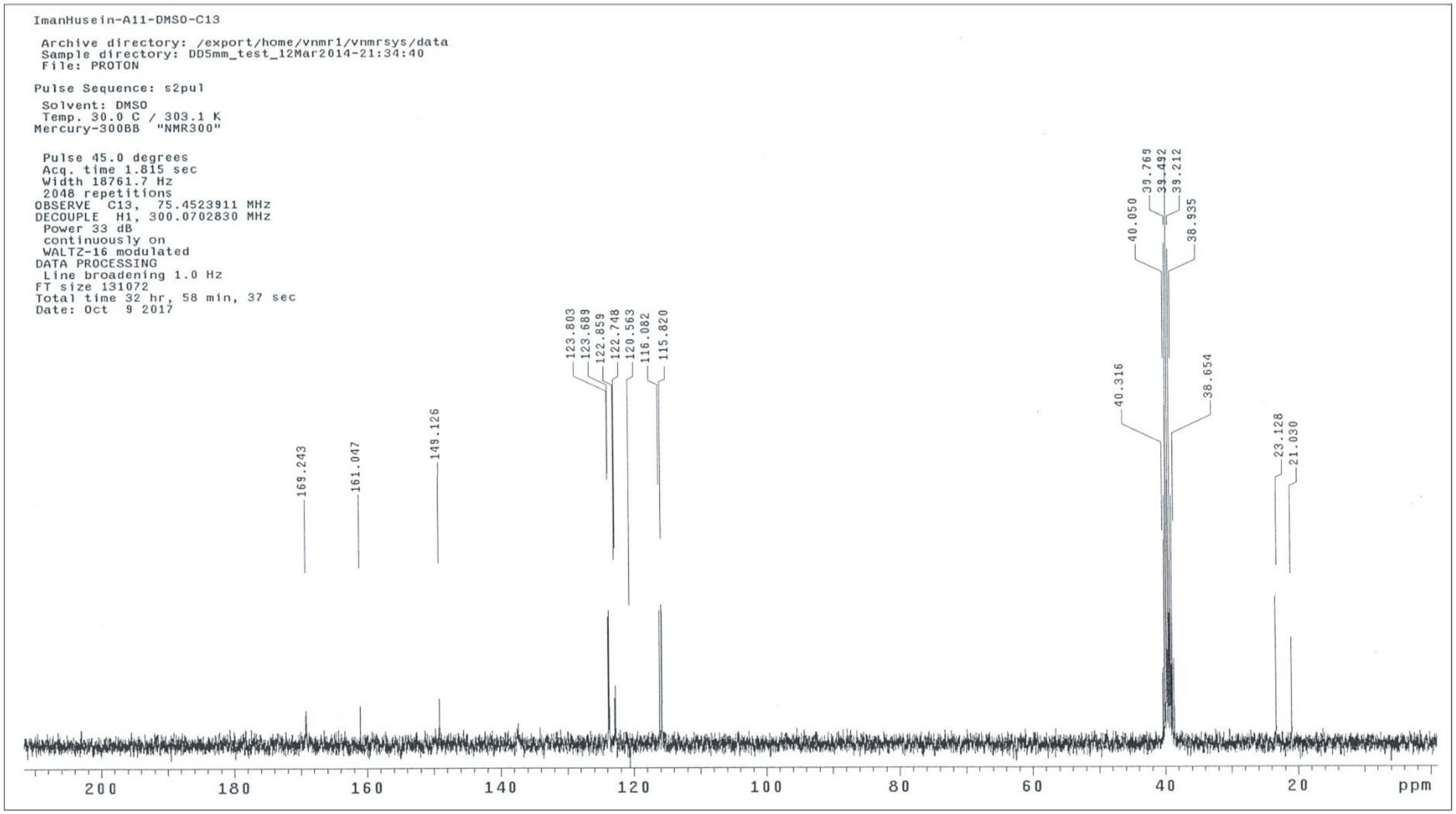
ImanHusein-A11-DMSO-C13
Archive directory: /export/home/vnmr1/vnmrsys/data
Sample directory: DD5mm_test_12Mar2014-21:34:40
File: PROTON
Pulse Sequence: s2pul
Solvent: DMSO
Temp. 30.0 C / 303.1 K
Mercury-300BB "NMR300"
Pulse 45.0 degrees
Acq. time 1.815 sec
Width 18761.7 Hz
2048 repetitions
OBSERVE C13, 75.4523911 MHz
DECOUPLE H1, 300.0702830 MHz
Power 33 dB
continuously on
WALTZ-16 modulated
DATA PROCESSING
Line broadening 1.0 Hz
FT size 131072
Total time 32 hr, 58 min, 37 sec
Date: Oct 9 2017
169.243
161.047
149.126
123.803
123.689
122.859
122.748
120.563
116.082
115.820
40.316
40.050
39.769
39.492
39.212
38.935
38.654
23.128
21.030
200
180
160
140
120
100
80
60
40
20
ppm

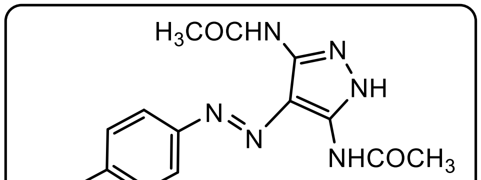
H3COCHN
N
NH
N
N
NHCOCH3
F3C

7b

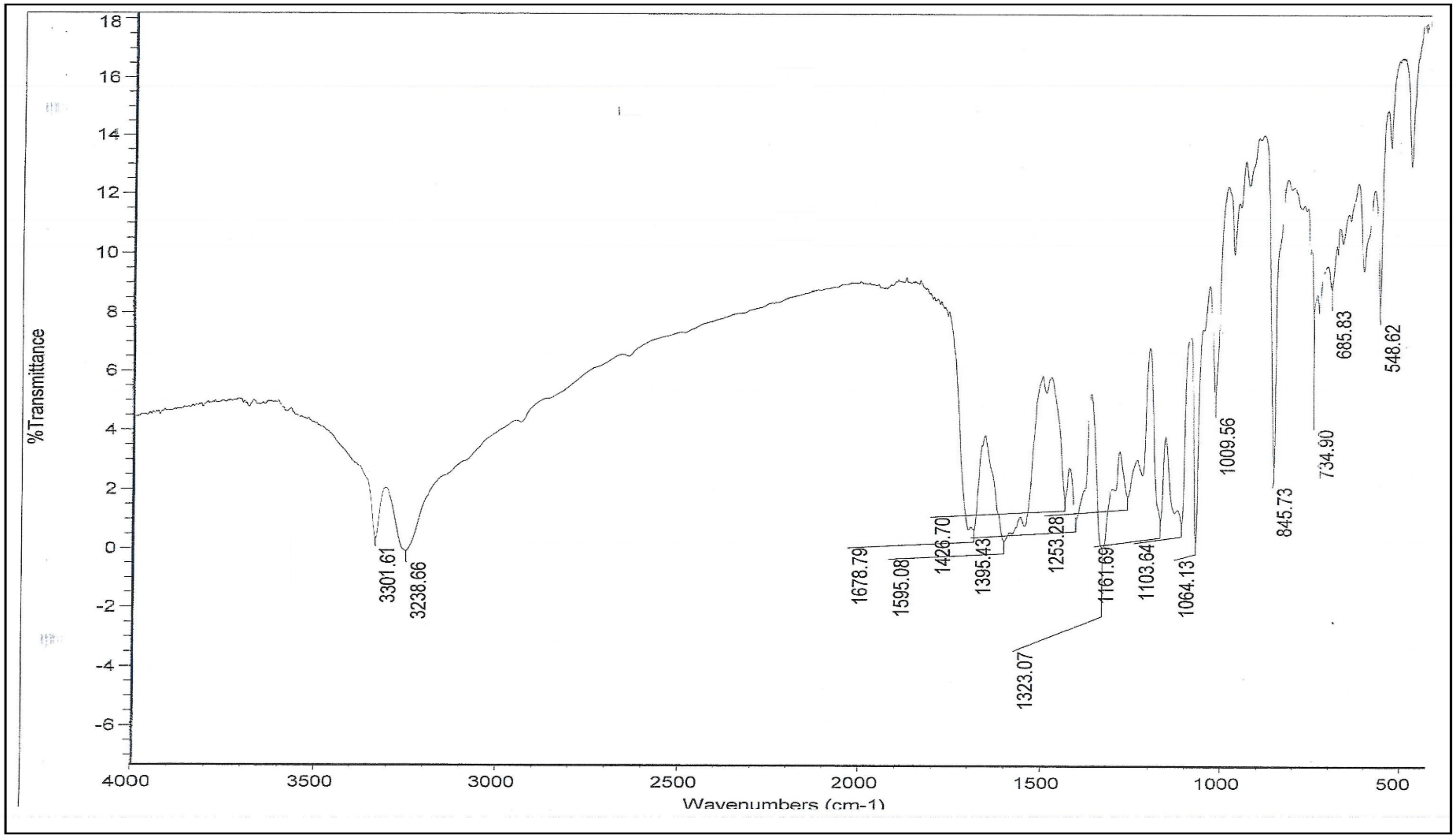
%Transmittance
Wavenumbers (cm-1)
3301.61
3238.66
1678.79
1595.08
1426.70
1395.43
1323.07
1253.28
1161.69
1103.64
1064.13
1009.56
845.73
734.90
685.83
548.62

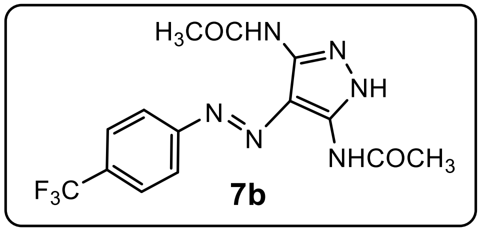
H3COCHN
N
NH
N
N
NHCOCH3
F3C
7b

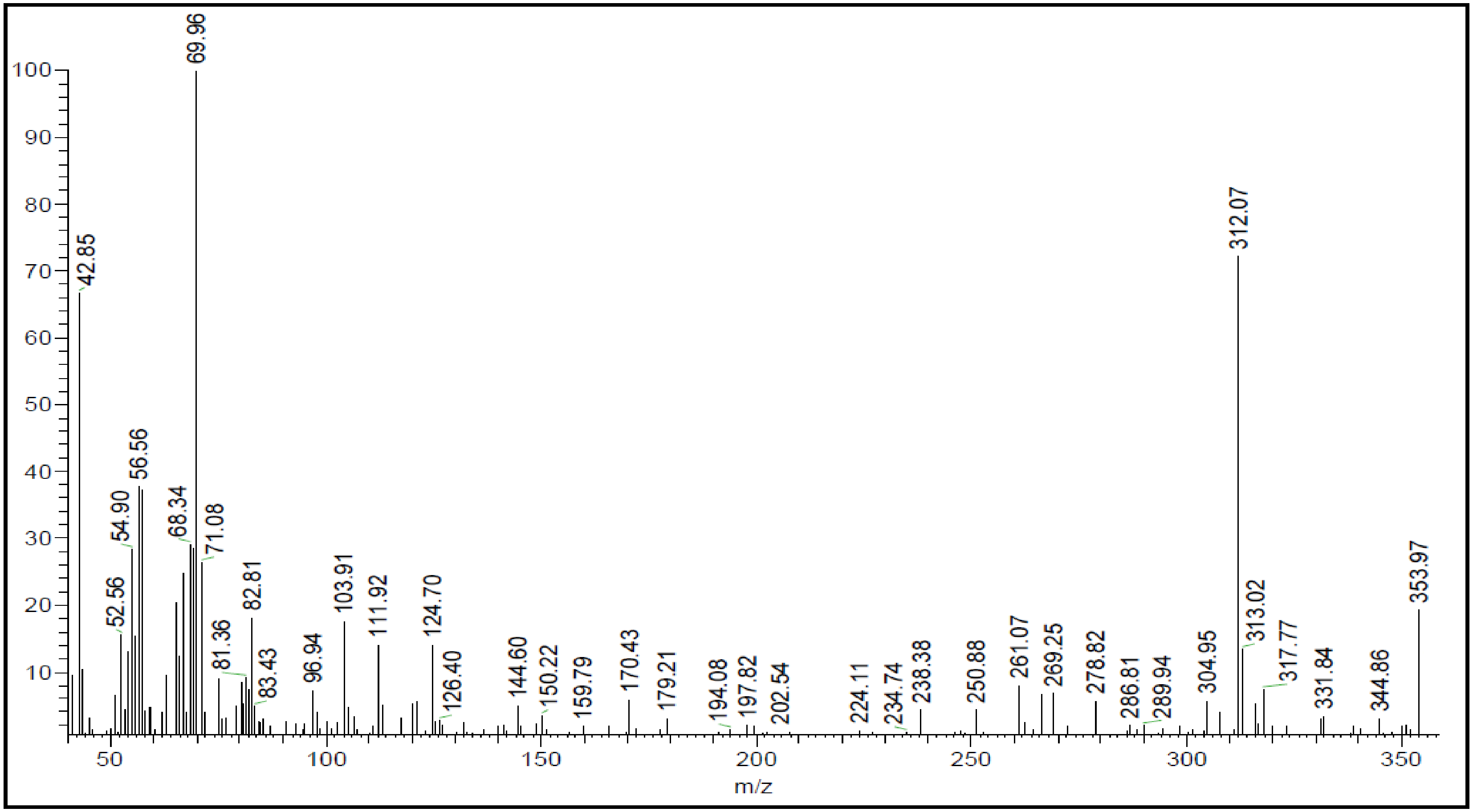
100
90
80
70
60
50
40
30
20
10
69.96
42.85
312.07
56.56
54.90
68.34
71.08
52.56
82.81
103.91
111.92
124.70
81.36
83.43
96.94
126.40
144.60
150.22
159.79
170.43
179.21
194.08
197.82
202.54
224.11
234.74
238.38
250.88
261.07
269.25
278.82
286.81
289.94
304.95
313.02
317.77
331.84
344.86
353.97
50
100
150
200
250
300
350
m/z

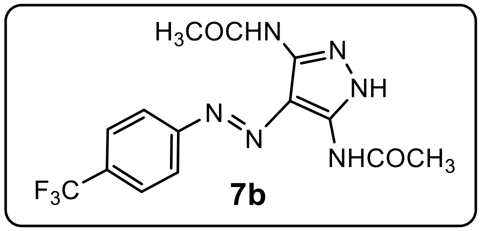
H3COCHN
N
NH
N
N
NHCOCH3
F3C

7b

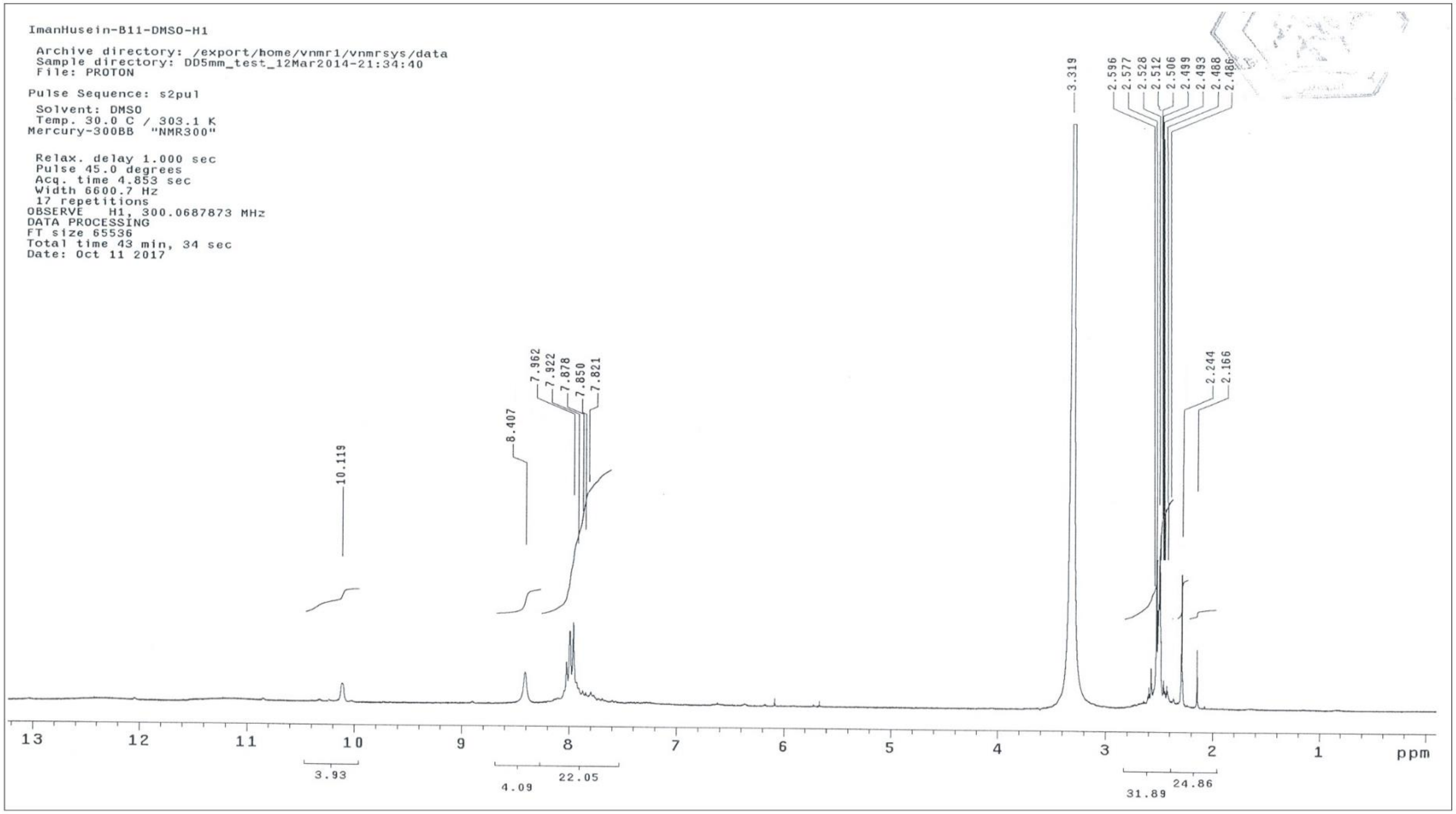
ImanHusein-B11-DMSO-H1
Archive directory: /export/home/vnmr1/vnmrsys/data
Sample directory: DD5mm_test_12Mar2014-21:34:40
File: PROTON
Pulse Sequence: s2pul
Solvent: DMSO
Temp. 30.0 C / 303.1 K
Mercury-300BB "NMR300"
Relax. delay 1.000 sec
Pulse 45.0 degrees
Acq. time 4.853 sec
Width 6600.7 Hz
17 repetitions
OBSERVE H1, 300.0687873 MHz
DATA PROCESSING
FT size 65536
Total time 43 min, 34 sec
Date: Oct 11 2017
10.119
8.407
7.962
7.922
7.878
7.850
7.821
3.319
2.596
2.577
2.528
2.512
2.506
2.499
2.493
2.488
2.486
2.244
2.166
13
12
11
10
9
8
7
6
5
4
3
2
1
ppm
3.93
4.09
22.05
31.89
24.86

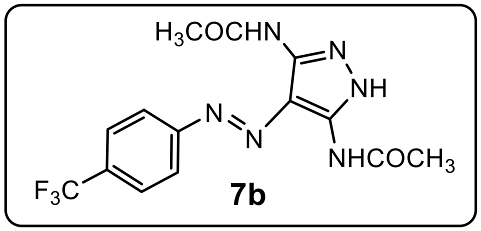
H3COCHN
N
NH
N
N
NHCOCH3
F3C

7b

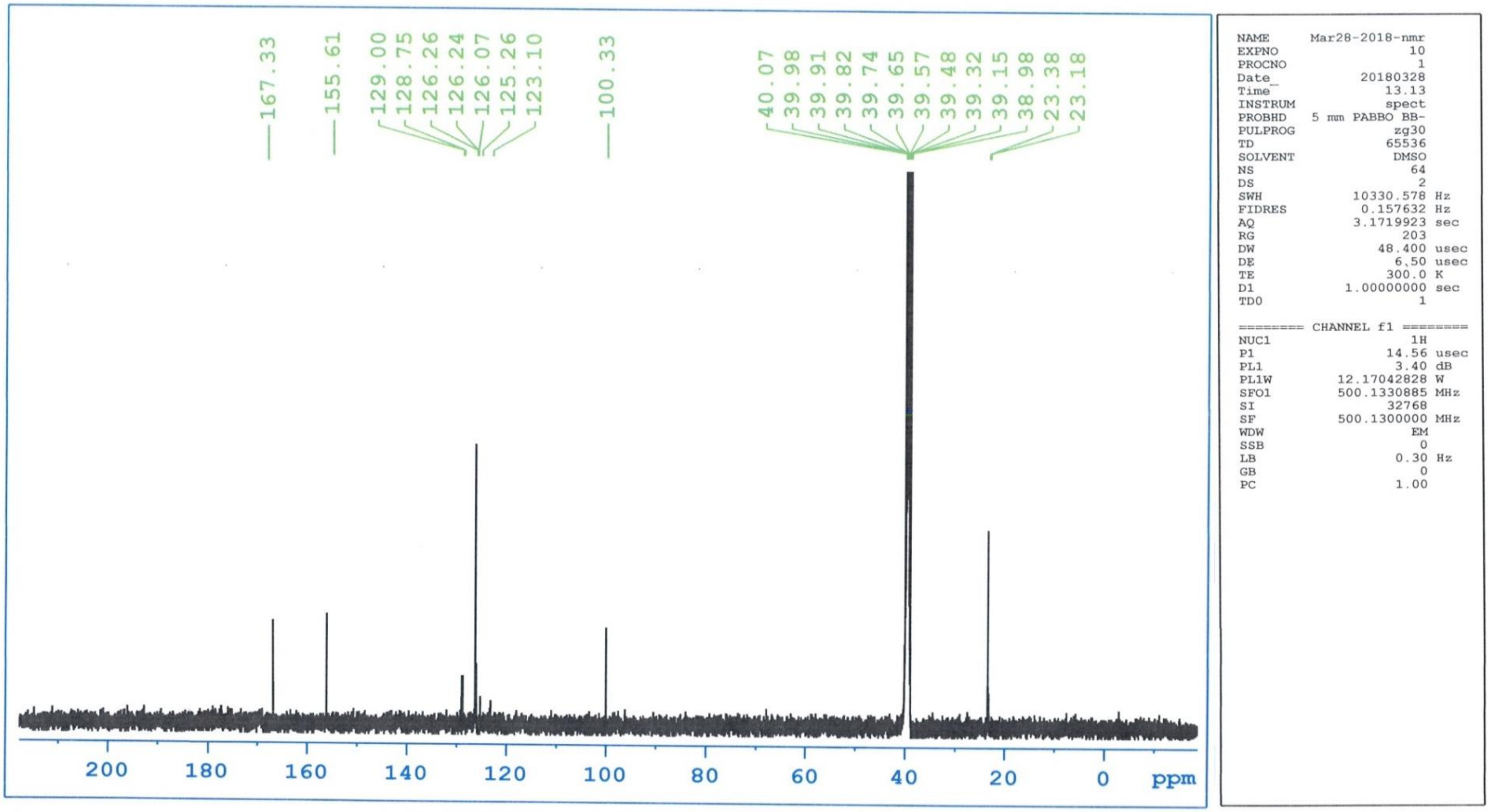
167.33
155.61
129.00
128.75
126.26
126.24
126.07
125.26
123.10
100.33
40.07
39.98
39.91
39.82
39.74
39.65
39.57
39.48
39.32
39.15
38.98
23.38
23.18
200 180 160 140 120 100 80 60 40 20 0 ppm
NAME Mar28-2018-nmr
EXPNO 10
PROCNO 1
Date_ 20180328
Time 13.13
INSTRUM spect
PROBHD 5 mm PABBO BB-
PULPROG zg30
TD 65536
SOLVENT DMSO
NS 64
DS 2
SWH 10330.578 Hz
FIDRES 0.157632 Hz
AQ 3.1719923 sec
RG 203
DW 48.400 usec
DE 6.50 usec
TE 300.0 K
D1 1.00000000 sec
TD0 1
======== CHANNEL f1 ========
NUC1 1H
P1 14.56 usec
PL1 3.40 dB
PL1W 12.17042828 W
SFO1 500.1330885 MHz
SI 32768
SF 500.1300000 MHz
WDW EM
SSB 0
LB 0.30 Hz
GB 0
PC 1.00

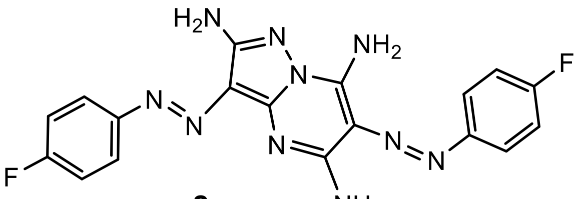
H2N
N
N
NH2
F
N
N
N
N
N
F
NH2

8a

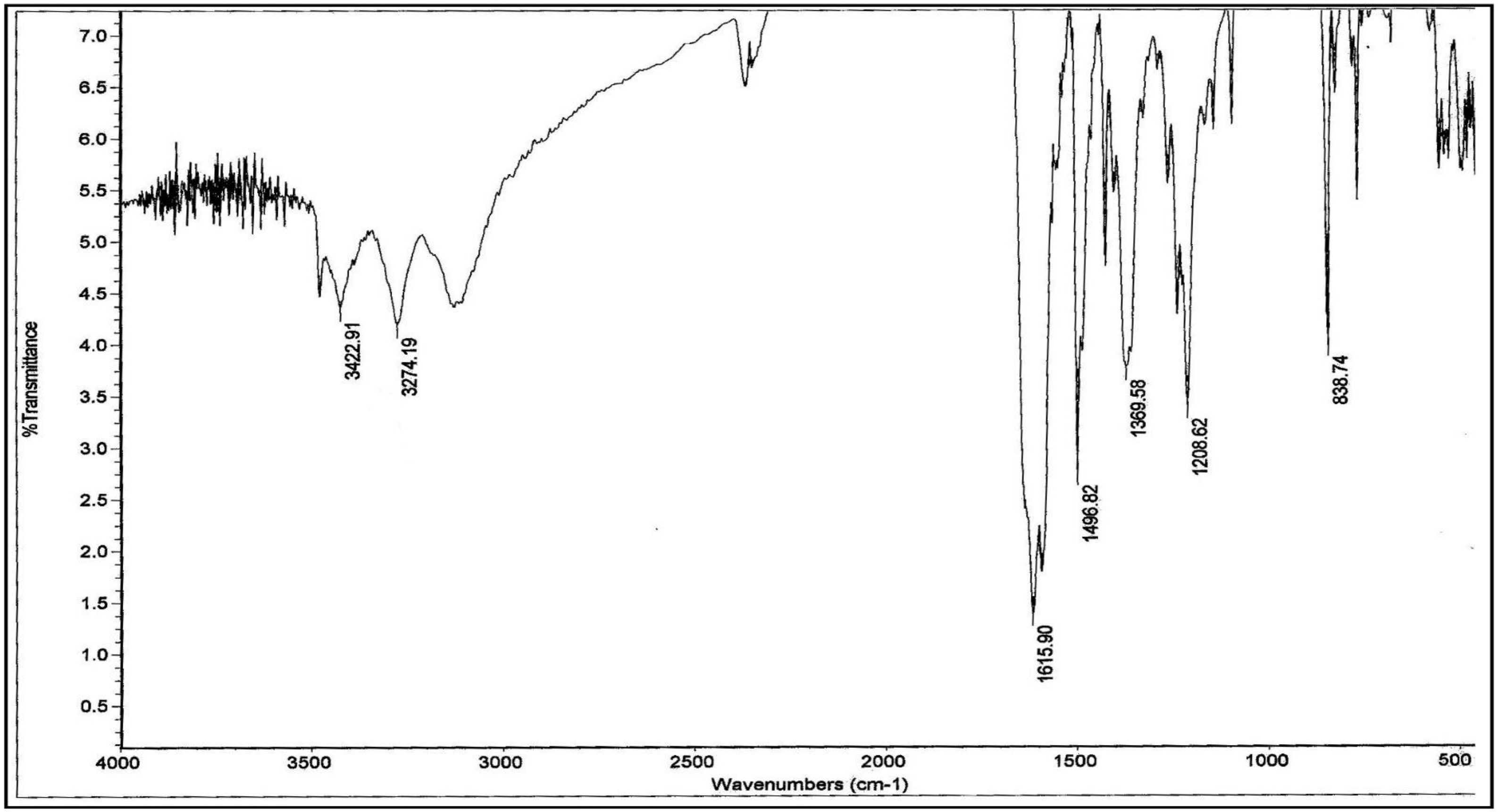
%Transmittance
Wavenumbers (cm-1)
3422.91
3274.19
1615.90
1496.82
1369.58
1208.62
838.74

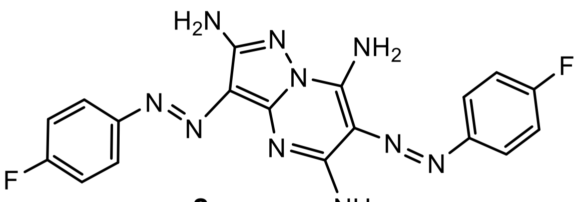

H2N
N
N
NH2
F
N
N
N
N
N
F
NH2

8a

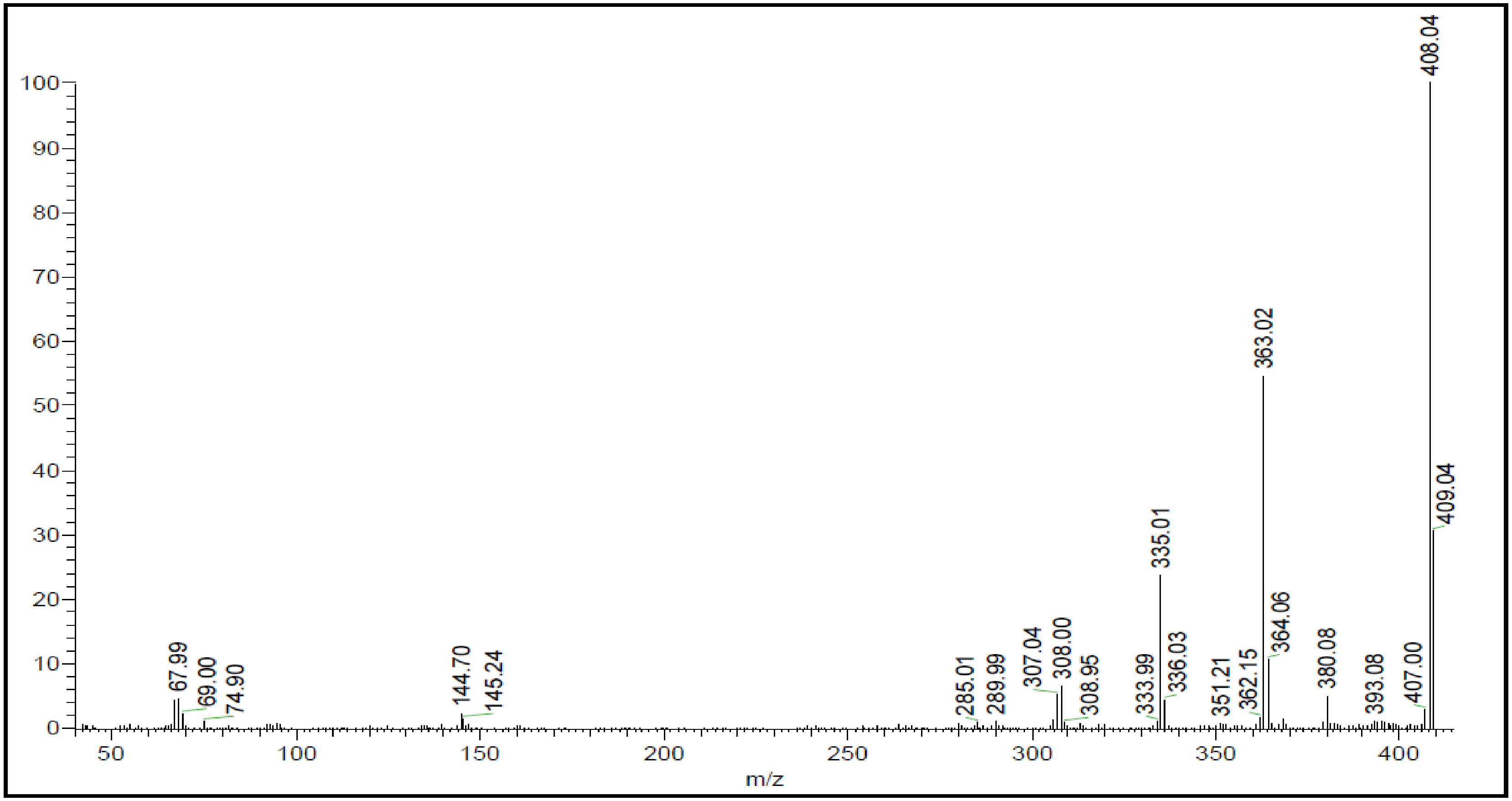

100
90
80
70
60
50
40
30
20
10
0
50
100
150
200
250
300
350
400
m/z
408.04
409.04
363.02
364.06
335.01
336.03
67.99
69.00
74.90
144.70
145.24
285.01
289.99
307.04
308.00
308.95
333.99
351.21
362.15
380.08
393.08
407.00

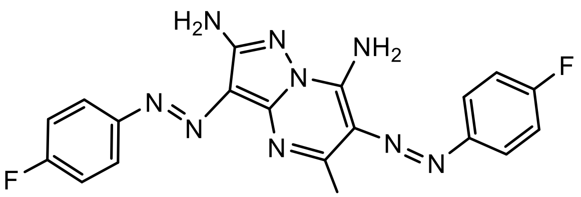
H2N
N
N
NH2
F
N
N
N
N
N
N
F
NH2

8a

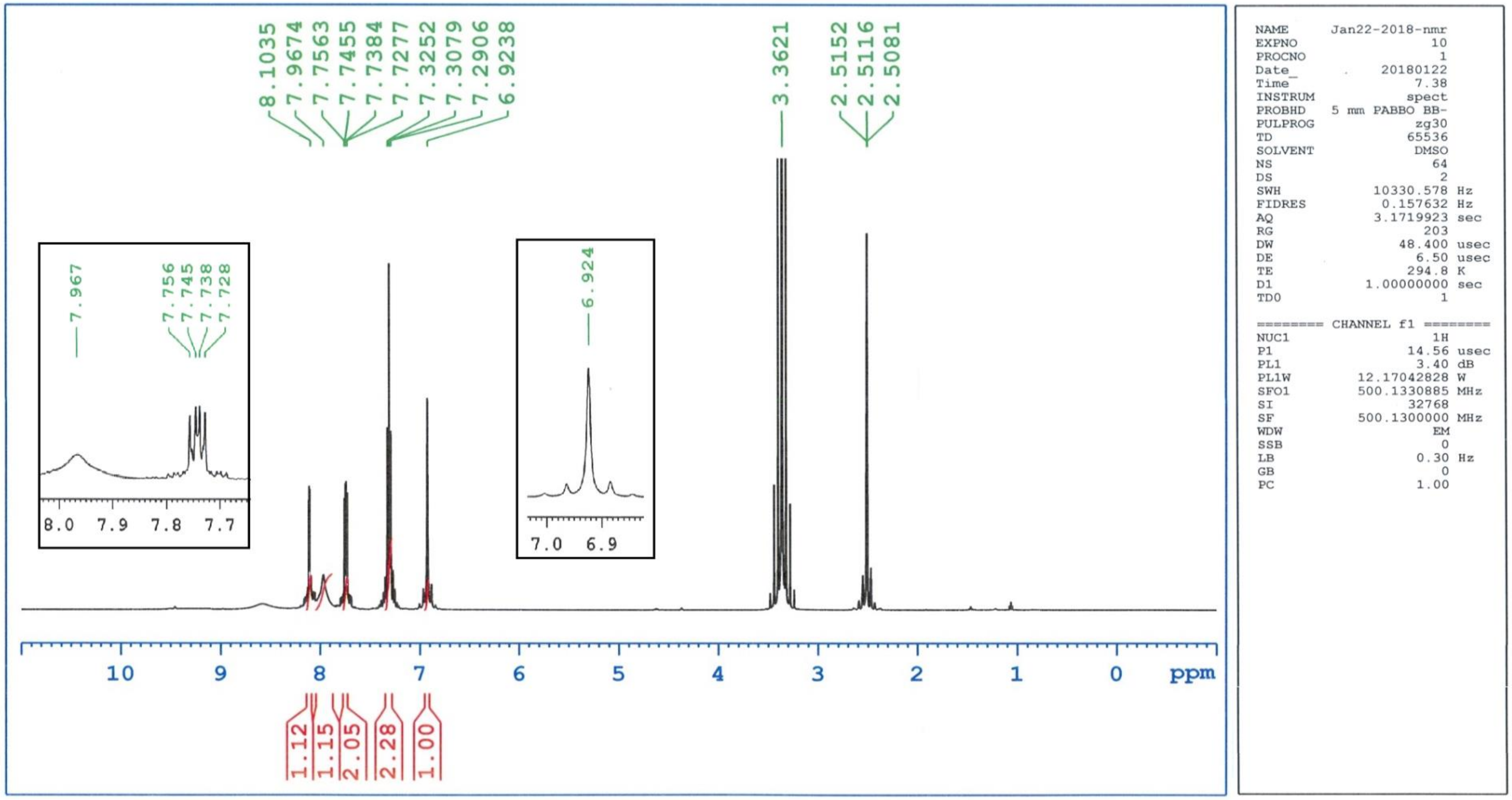
8.1035
7.9674
7.7563
7.7455
7.7384
7.7277
7.3252
7.3079
7.2906
6.9238
3.3621
2.5152
2.5116
2.5081
7.967
7.756
7.745
7.738
7.728
8.0 7.9 7.8 7.7
6.924
7.0 6.9
10 9 8 7 6 5 4 3 2 1 0 ppm
1.12
1.15
2.05
2.28
1.00
NAME Jan22-2018-nmr
EXPNO 10
PROCNO 1
Date_ 20180122
Time 7.38
INSTRUM spect
PROBHD 5 mm PABBO BB-
PULPROG zg30
TD 65536
SOLVENT DMSO
NS 64
DS 2
SWH 10330.578 Hz
FIDRES 0.157632 Hz
AQ 3.1719923 sec
RG 203
DW 48.400 usec
DE 6.50 usec
TE 294.8 K
D1 1.00000000 sec
TD0 1
======== CHANNEL f1 ========
NUC1 1H
P1 14.56 usec
PL1 3.40 dB
PL1W 12.17042828 W
SFO1 500.1330885 MHz
SI 32768
SF 500.1300000 MHz
WDW EM
SSB 0
LB 0.30 Hz
GB 0
PC 1.00

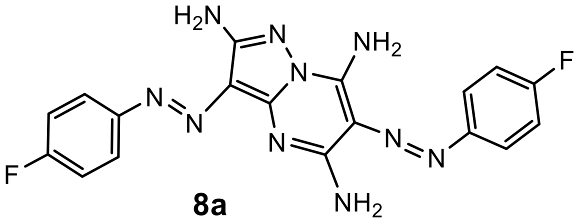

8a

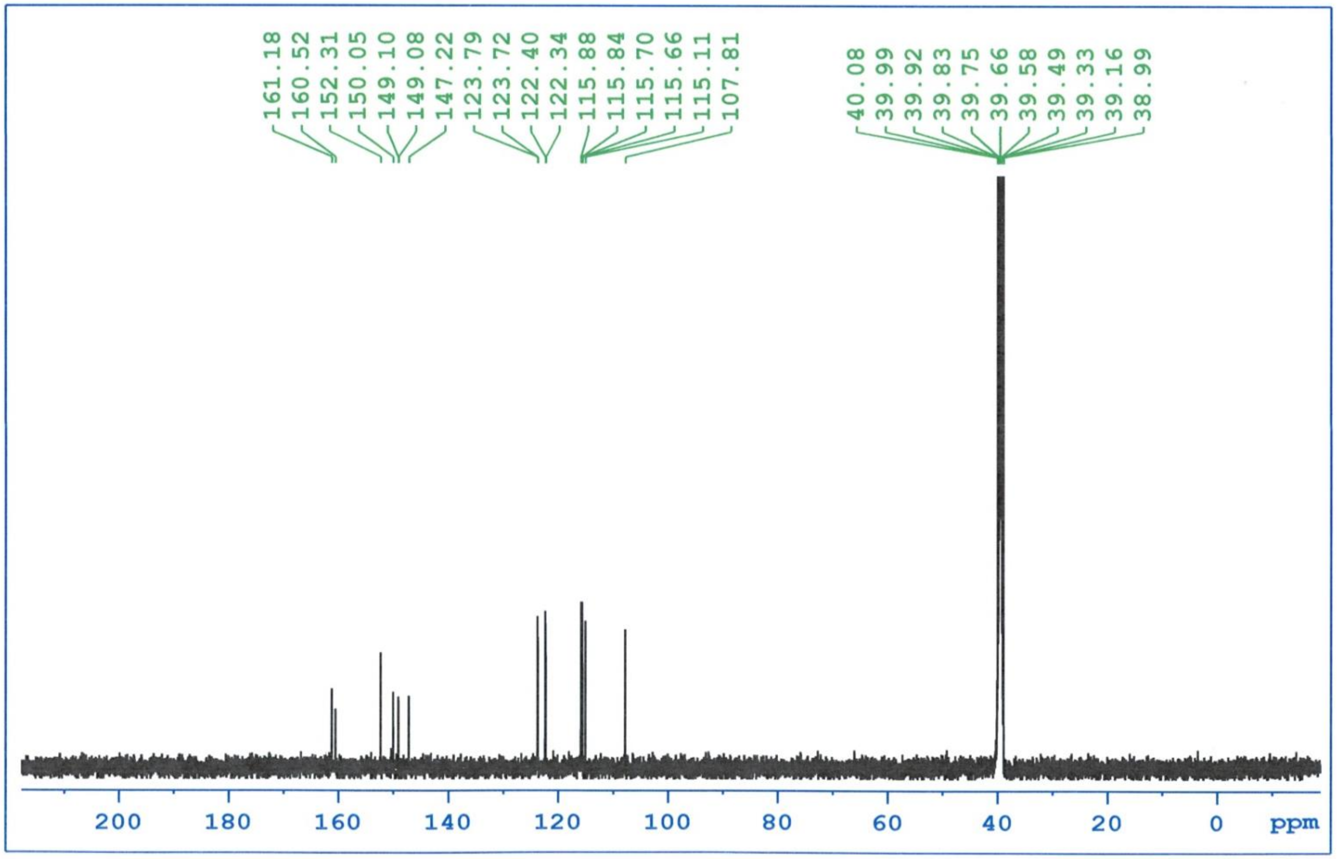

NAME Jan22-2018-nmr
EXPNO 10
PROCNO 1
Date_ 20180122
Time 7.38
INSTRUM spect
PROBHD 5 mm PABBO BB-
PULPROG zg30
TD 65536
SOLVENT DMSO
NS 64
DS 2
SWH 10330.578 Hz
FIDRES 0.157632 Hz
AQ 3.1719923 sec
RG 203
DW 48.400 usec
DE 6.50 usec
TE 294.8 K
D1 1.00000000 sec
TD0 1

======== CHANNEL f1 ========
NUC1 1H
P1 14.56 usec
PL1 3.40 dB
PL1W 12.17042828 W
SFO1 500.1330885 MHz
SI 32768
SF 500.1300000 MHz
WDW EM
SSB 0
LB 0.30 Hz
GB 0
PC 1.00

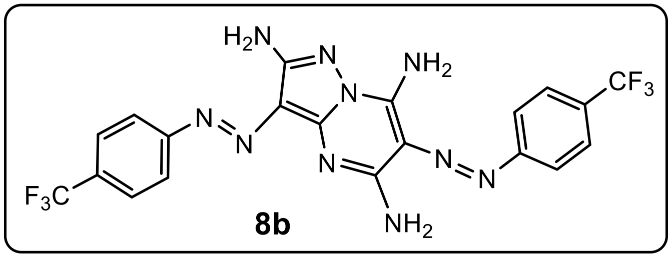

H_2N
NH_2
CF_3
F_3C
NH_2

8b

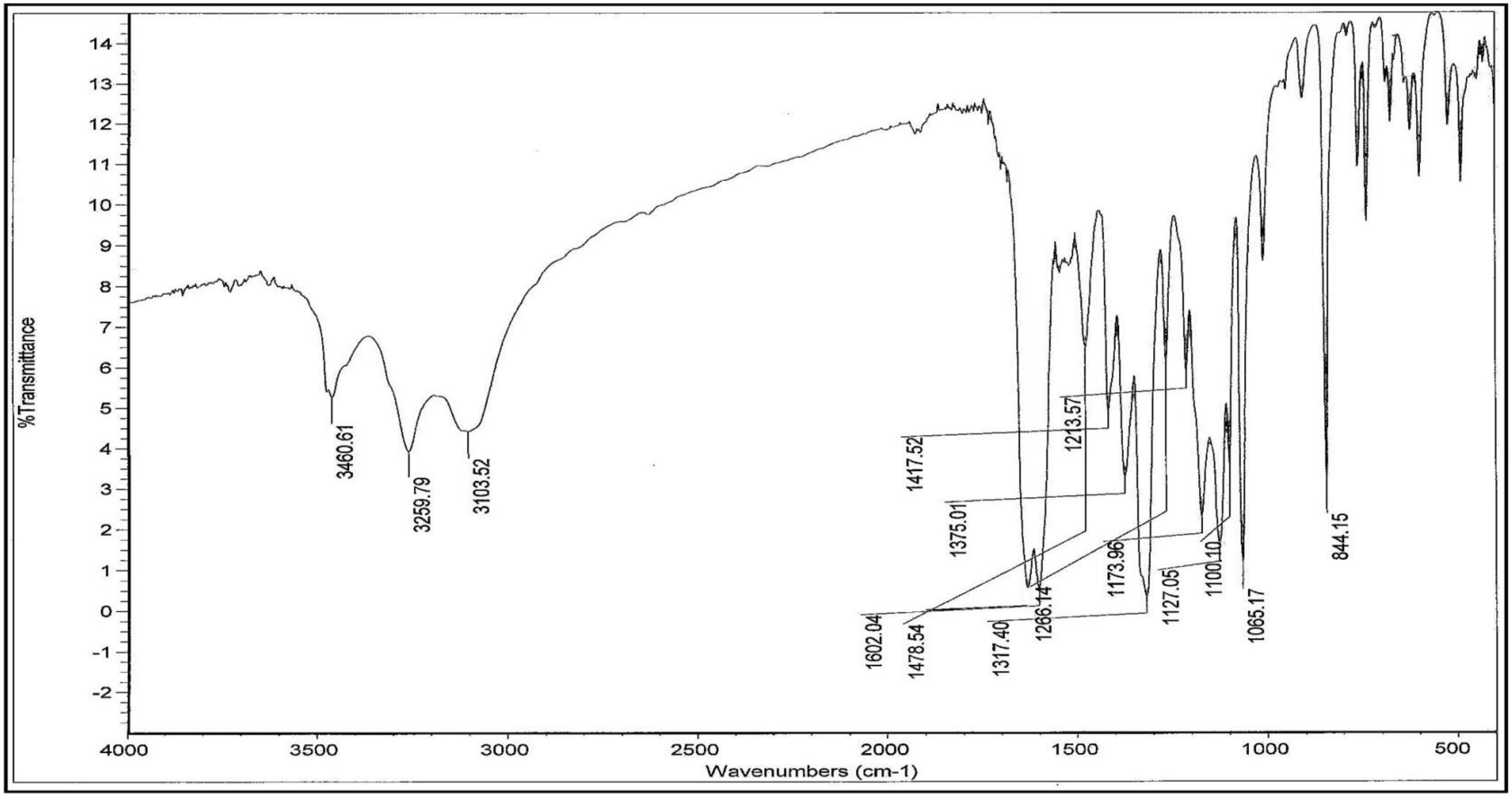

%Transmittance
Wavenumbers (cm-1)
3460.61
3259.79
3103.52
1417.52
1213.57
1375.01
1173.96
1127.05
1100.10
1065.17
844.15
1602.04
1478.54
1317.40
1266.14

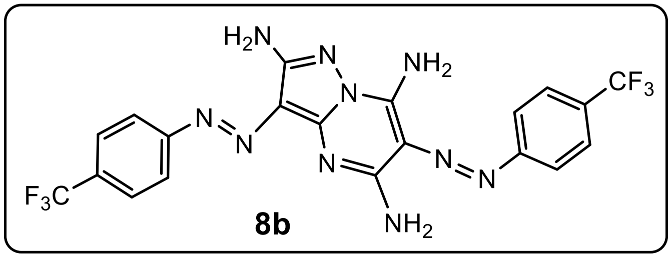

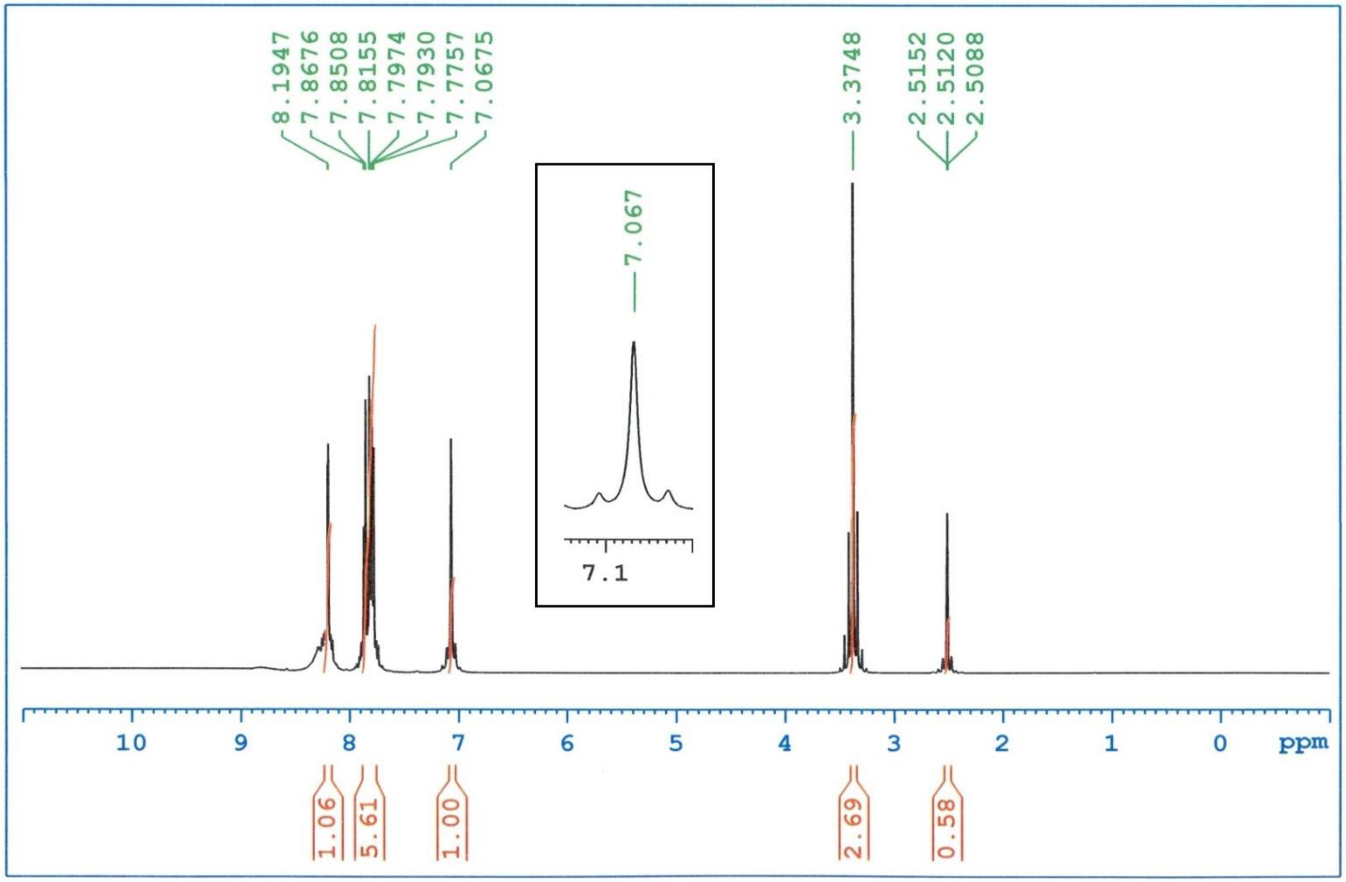

NAME	Jan22-2018-nmr
EXPNO	10
PROCNO	1
Date_	20180122
Time	7.38
INSTRUM	spect
PROBHD	5 mm PABBO BB-
PULPROG	zg30
TD	65536
SOLVENT	DMSO
NS	64
DS	2
SWH	10330.578 Hz
FIDRES	0.157632 Hz
AQ	3.1719923 sec
RG	203
DW	48.400 usec
DE	6.50 usec
TE	294.8 K
D1	1.00000000 sec
TD0	1
======== CHANNEL f1 ========	
NUC1	1H
P1	14.56 usec
PL1	3.40 dB
PL1W	12.17042828 W
SFO1	500.1330885 MHz
SI	32768
SF	500.1300000 MHz
WDW	EM
SSB	0
LB	0.30 Hz
GB	0
PC	1.00

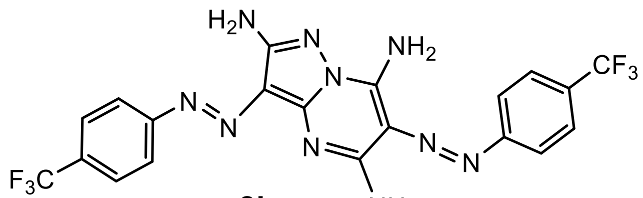

8b

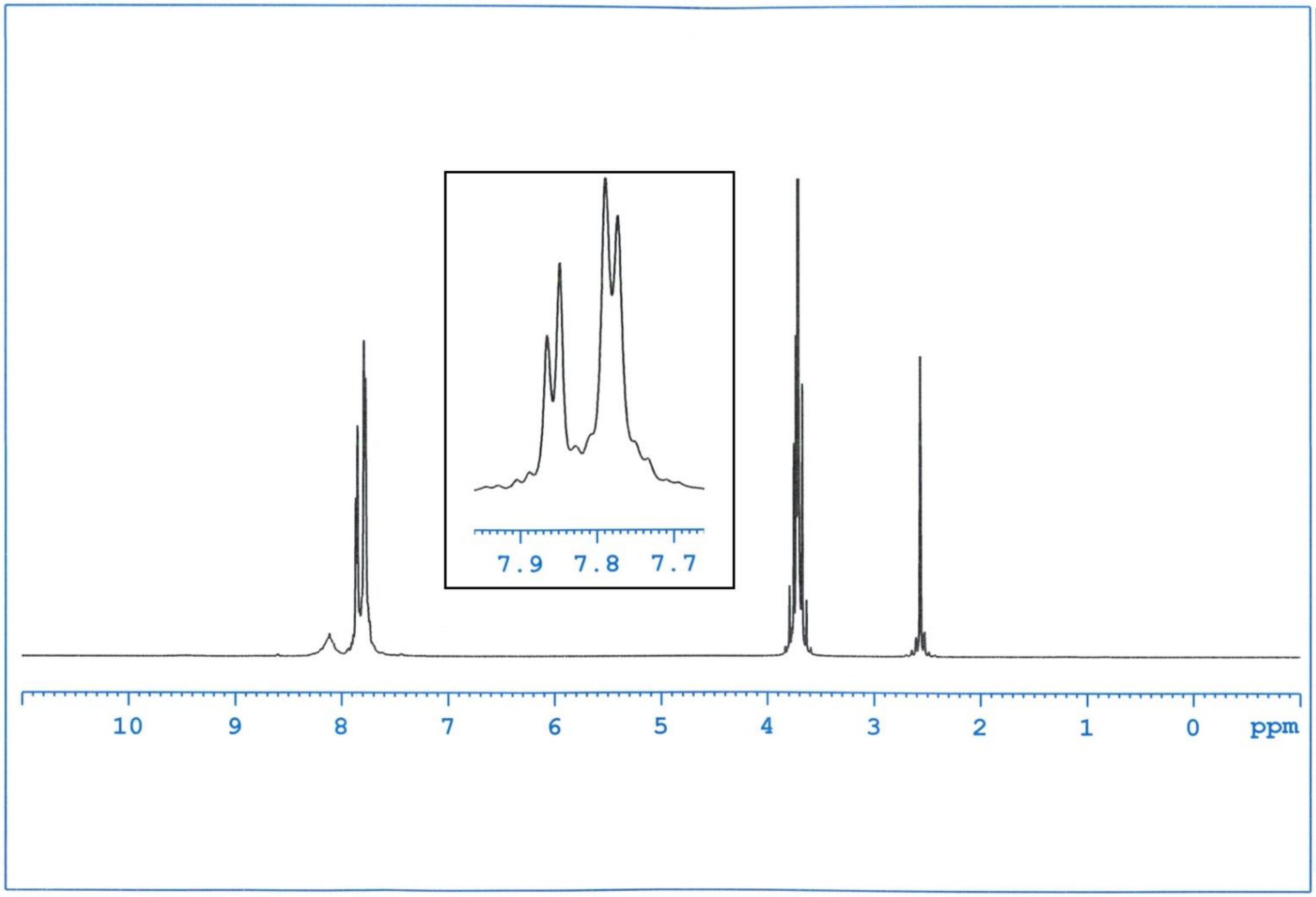

NAME	Jan22-2018-nmr
EXPNO	10
PROCNO	1
Date_	20180122
Time	7.38
INSTRUM	spect
PROBHD	5 mm PABBO BB-
PULPROG	zg30
TD	65536
SOLVENT	DMSO
NS	64
DS	2
SWH	10330.578 Hz
FIDRES	0.157632 Hz
AQ	3.1719923 sec
RG	203
DW	48.400 usec
DE	6.50 usec
TE	294.8 K
D1	1.00000000 sec
TD0	1
======== CHANNEL f1 ========	
NUC1	1H
P1	14.56 usec
PL1	3.40 dB
PL1W	12.17042828 W
SFO1	500.1330885 MHz
SI	32768
SF	500.1300000 MHz
WDW	EM
SSB	0
LB	0.30 Hz
GB	0
PC	1.00

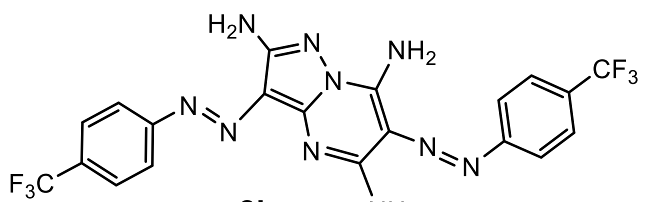

8b

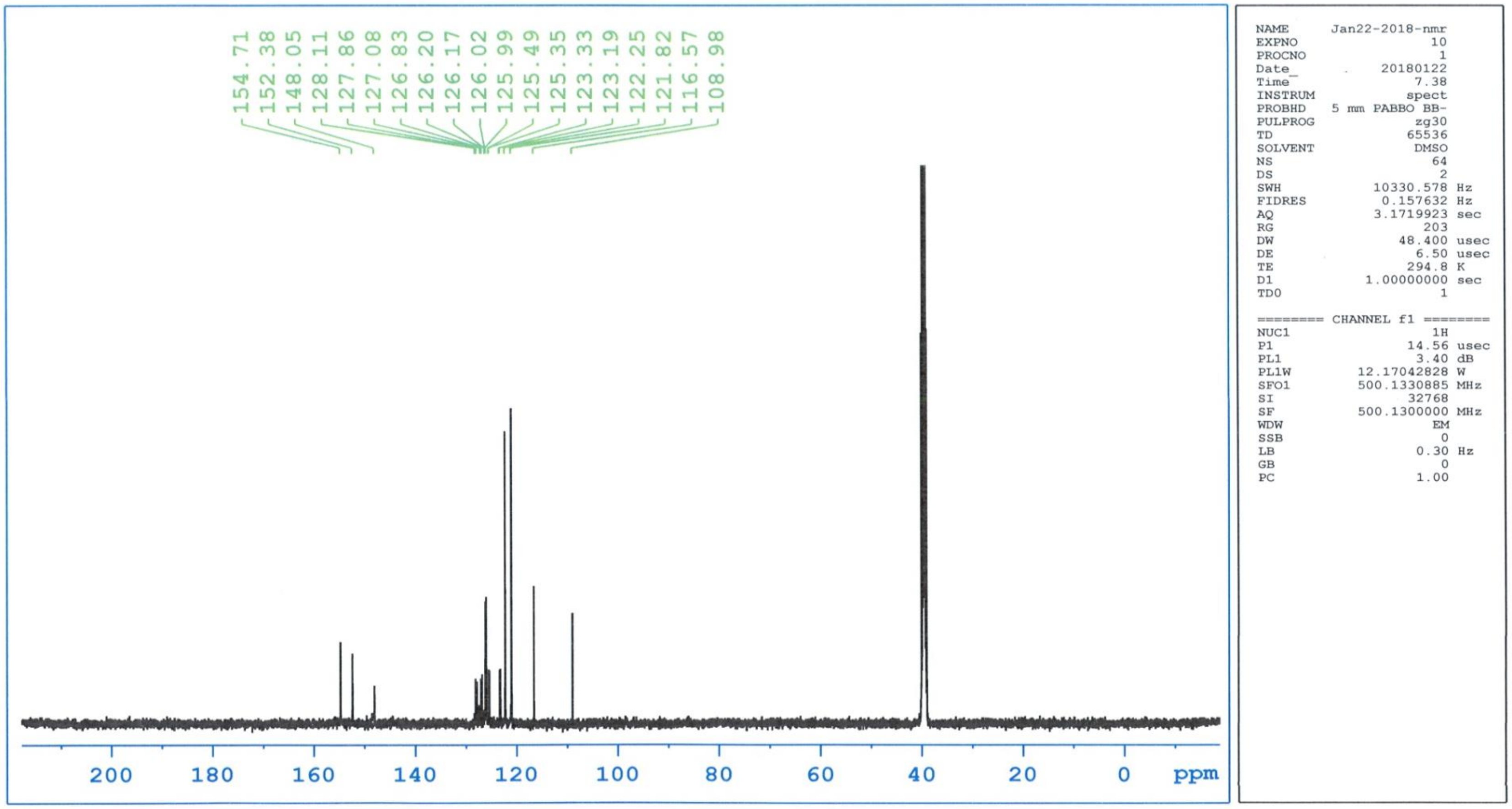

NAME	Jan22-2018-nmr
EXPNO	10
PROCNO	1
Date_	20180122
Time	7.38
INSTRUM	spect
PROBHD	5 mm PABBO BB-
PULPROG	zg30
TD	65536
SOLVENT	DMSO
NS	64
DS	2
SWH	10330.578 Hz
FIDRES	0.157632 Hz
AQ	3.1719923 sec
RG	203
DW	48.400 usec
DE	6.50 usec
TE	294.8 K
D1	1.00000000 sec
TD0	1
======== CHANNEL f1 ========	
NUC1	1H
P1	14.56 usec
PL1	3.40 dB
PL1W	12.17042828 W
SFO1	500.1330885 MHz
SI	32768
SF	500.1300000 MHz
WDW	EM
SSB	0
LB	0.30 Hz
GB	0
PC	1.00

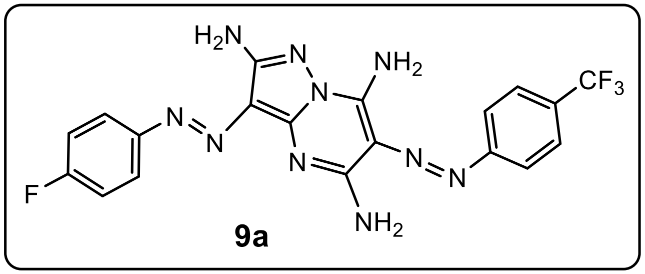

H2N
N
N
NH2
CF3
N
N
F
N
N
N
NH2

9a

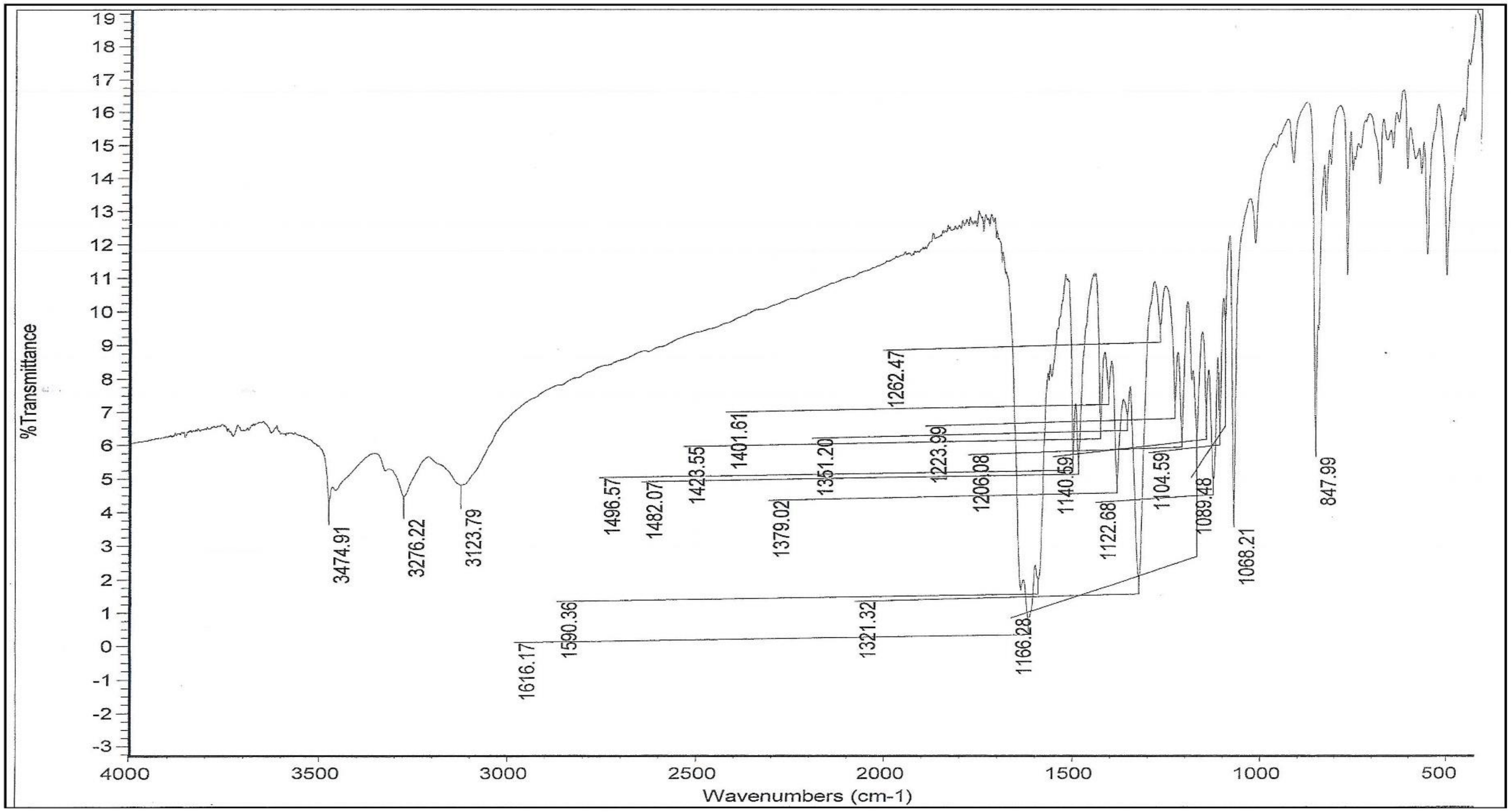

%Transmittance
Wavenumbers (cm-1)
3474.91
3276.22
3123.79
1616.17
1590.36
1496.57
1482.07
1423.55
1401.61
1379.02
1351.20
1321.32
1262.47
1223.99
1206.08
1166.28
1140.59
1122.68
1104.59
1089.48
1068.21
847.99

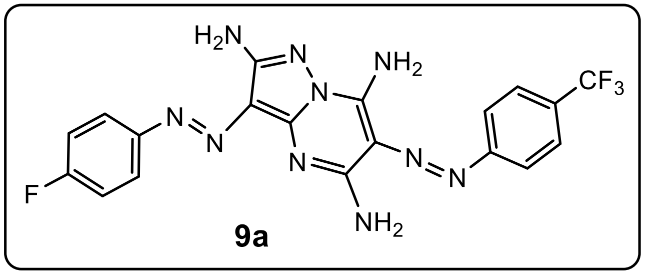

9a

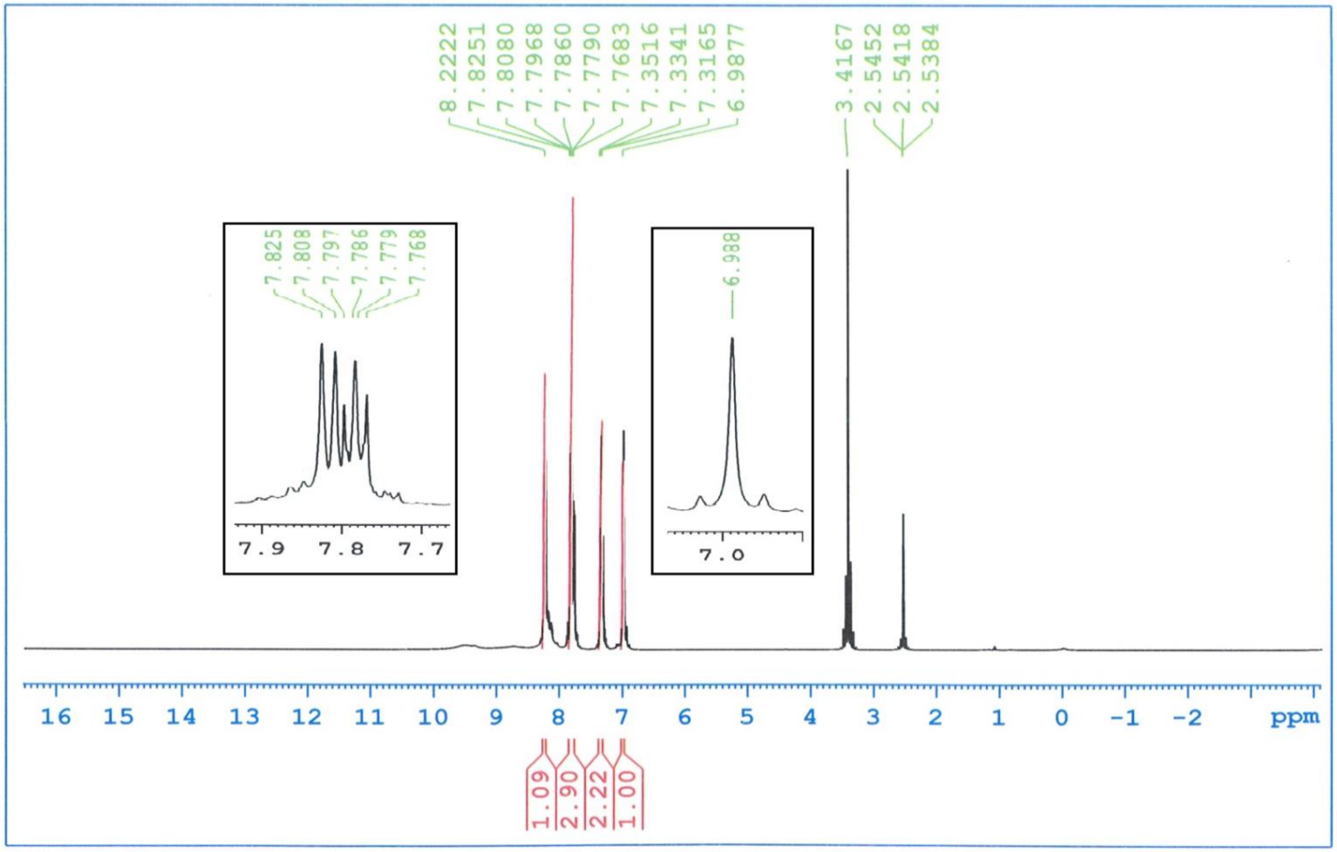

NAME	Jan14-2018-nmr
EXPNO	30
PROCNO	1
Date_	20180114
Time	15.55
INSTRUM	spect
PROBHD	5 mm PABBO BB-
PULPROG	zg30
TD	65536
SOLVENT	DMSO
NS	64
DS	2
SWH	10330.578 Hz
FIDRES	0.157632 Hz
AQ	3.1719923 sec
RG	203
DW	48.400 usec
DE	6.50 usec
TE	300.0 K
D1	1.00000000 sec
TD0	1
======== CHANNEL f1 ========	
NUC1	1H
P1	14.56 usec
PL1	3.40 dB
PL1W	12.17042828 W
SFO1	500.1330885 MHz
SI	32768
SF	500.1300000 MHz
WDW	EM
SSB	0
LB	0.30 Hz
GB	0
PC	1.00

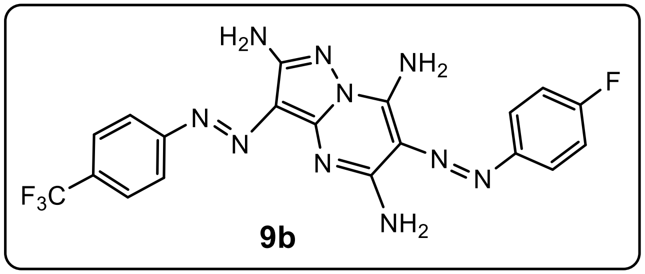
9b

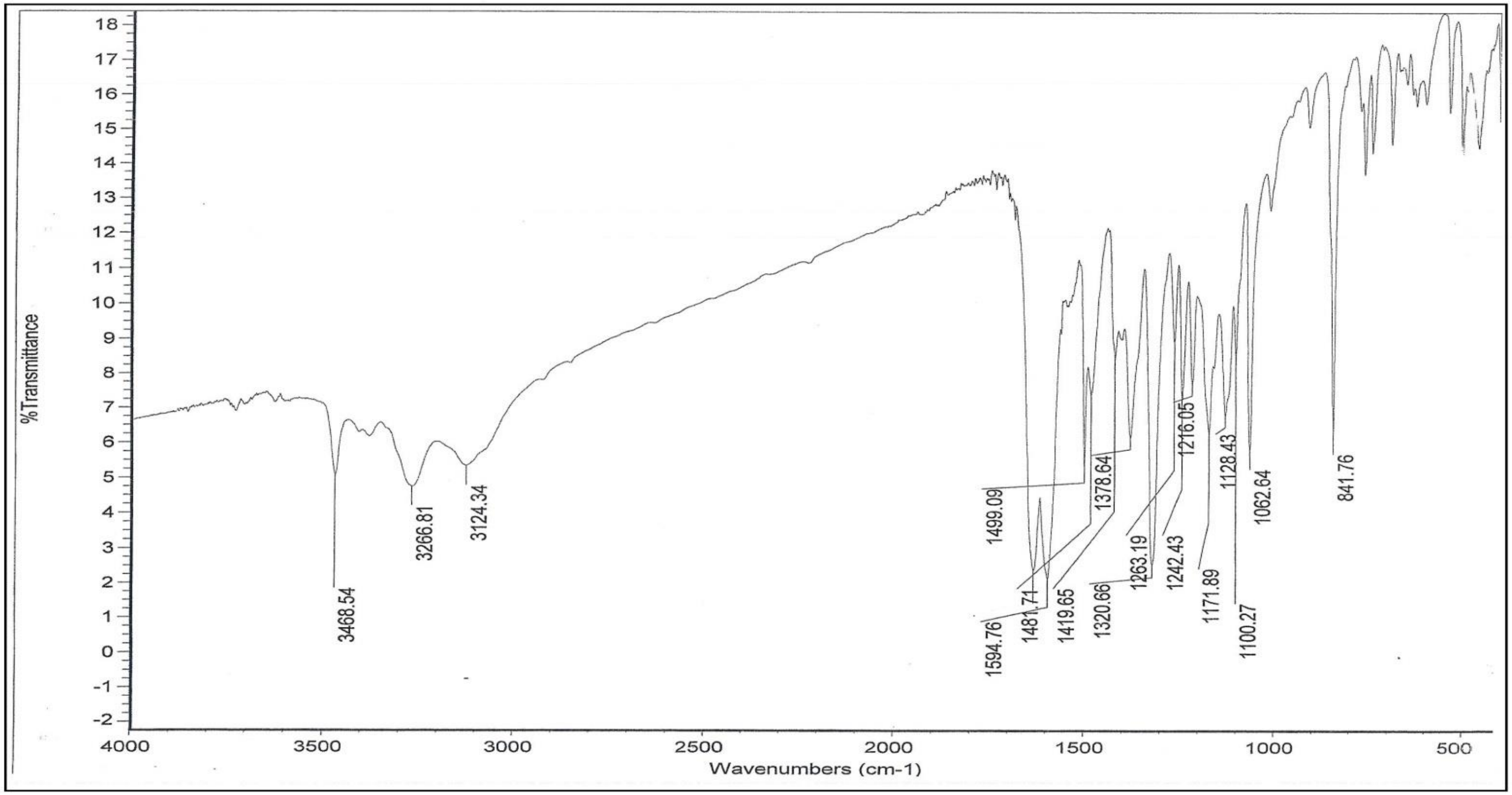
%Transmittance
Wavenumbers (cm-1)
3468.54
3266.81
3124.34
1594.76
1499.09
1481.71
1419.65
1378.64
1320.66
1263.19
1242.43
1216.05
1171.89
1128.43
1100.27
1062.64
841.76

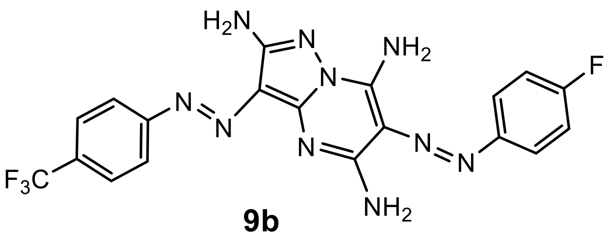
H2N
N
N
NH2
F
N
N
N
F3C
N
N
NH2

9b

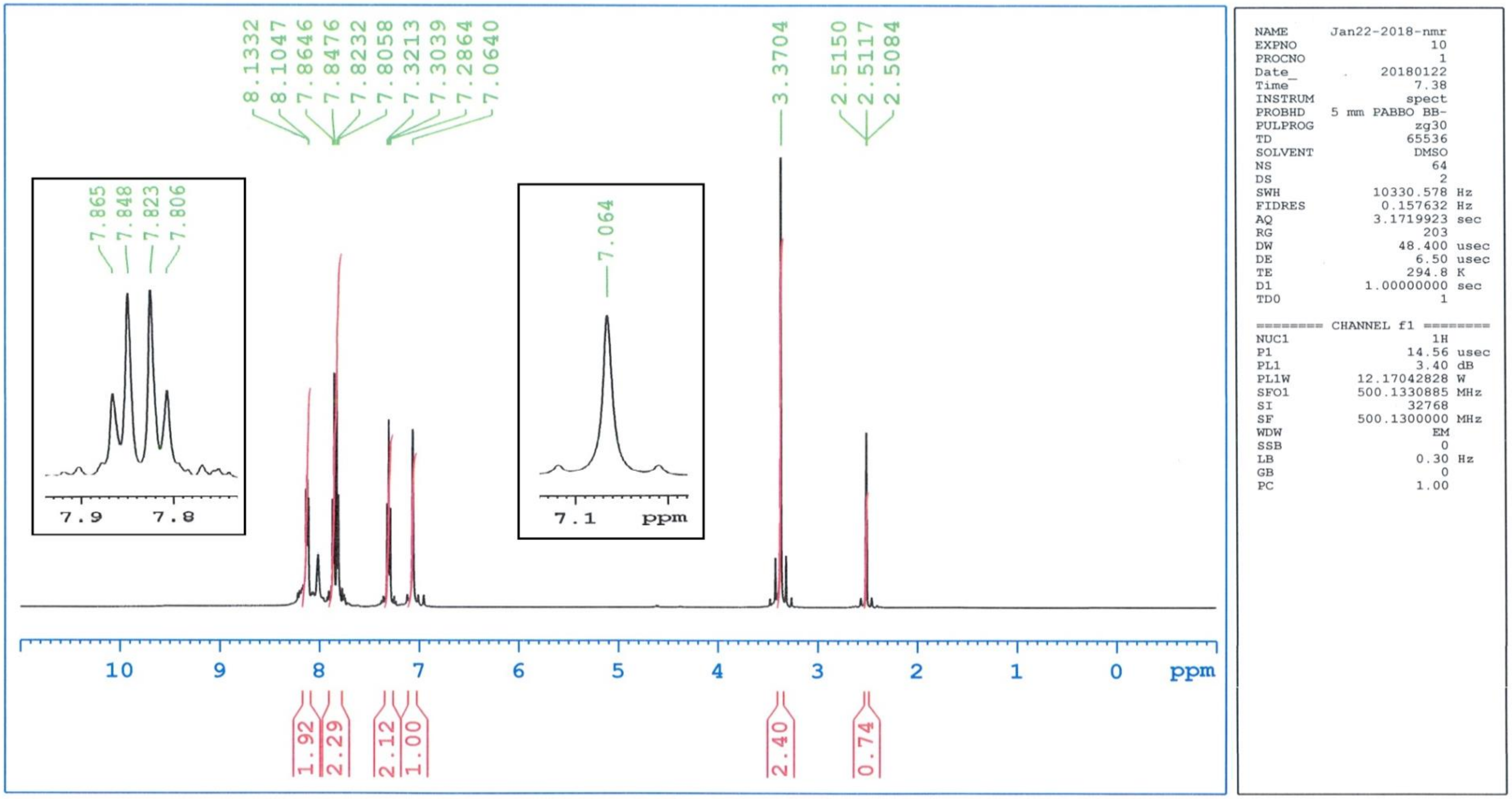
8.1332
8.1047
7.8646
7.8476
7.8232
7.8058
7.3213
7.3039
7.2864
7.0640
3.3704
2.5150
2.5117
2.5084
7.865
7.848
7.823
7.806
7.9
7.8
7.064
7.1
ppm
10
9
8
7
6
5
4
3
2
1
0
ppm
1.92
2.29
2.12
1.00
2.40
0.74
NAME Jan22-2018-nmr
EXPNO 10
PROCNO 1
Date_ 20180122
Time 7.38
INSTRUM spect
PROBHD 5 mm PABBO BB-
PULPROG zg30
TD 65536
SOLVENT DMSO
NS 64
DS 2
SWH 10330.578 Hz
FIDRES 0.157632 Hz
AQ 3.1719923 sec
RG 203
DW 48.400 usec
DE 6.50 usec
TE 294.8 K
D1 1.00000000 sec
TD0 1
======== CHANNEL f1 ========
NUC1 1H
P1 14.56 usec
PL1 3.40 dB
PL1W 12.17042828 W
SFO1 500.1330885 MHz
SI 32768
SF 500.1300000 MHz
WDW EM
SSB 0
LB 0.30 Hz
GB 0
PC 1.00

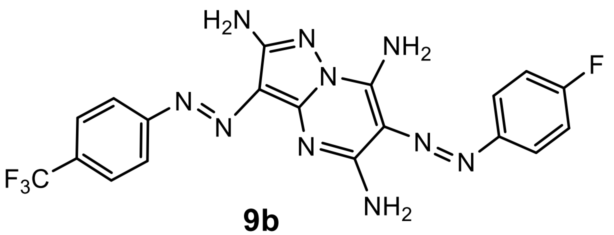
H2N
N
N
NH2
F
N
N
N
N
N
F3C
NH2

9b

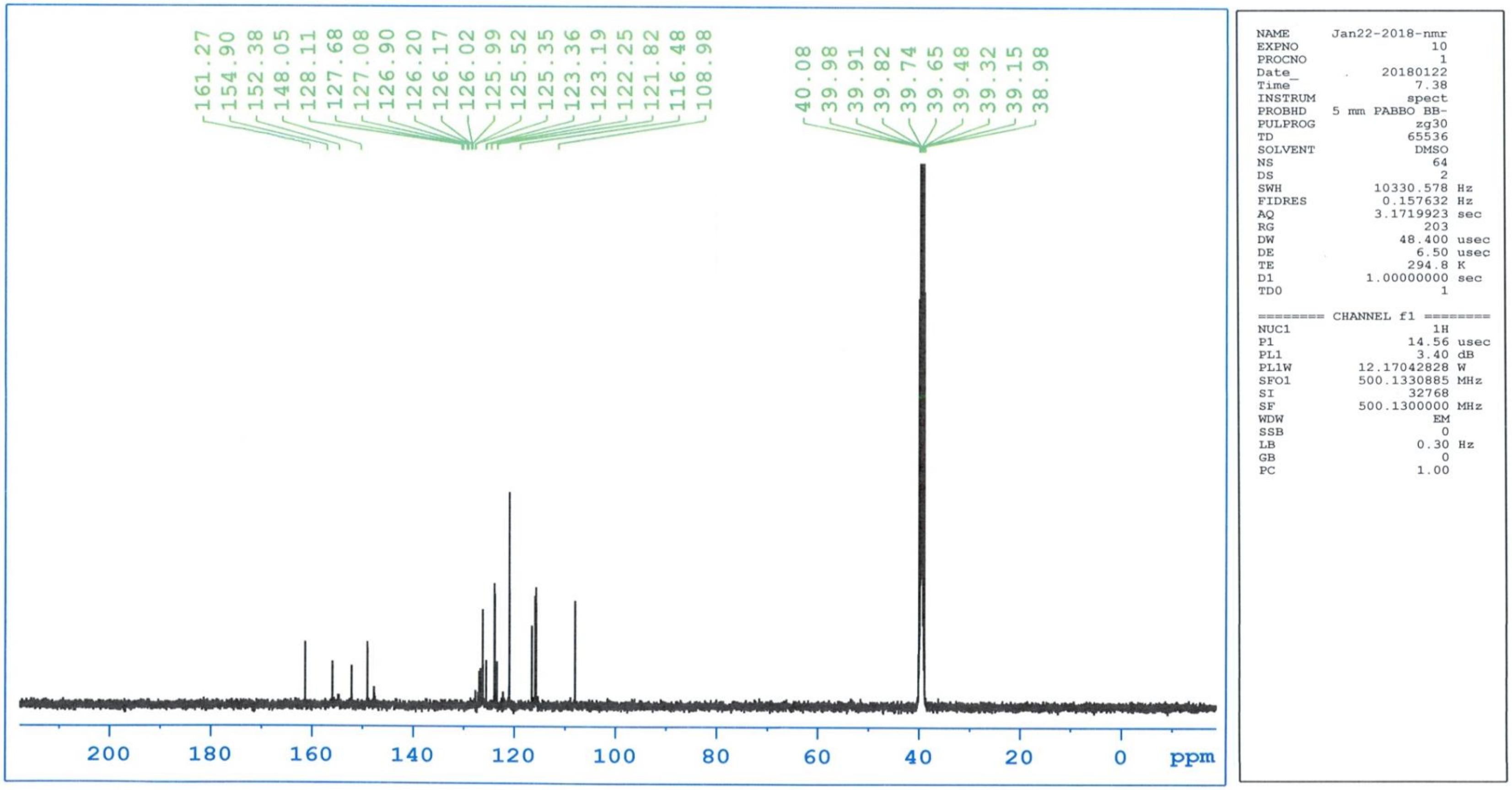
161.27
154.90
152.38
148.05
128.11
127.68
127.08
126.90
126.20
126.17
126.02
125.99
125.52
125.35
123.36
123.19
122.25
121.82
116.48
108.98
40.08
39.98
39.91
39.82
39.74
39.65
39.48
39.32
39.15
38.98
200
180
160
140
120
100
80
60
40
20
0
ppm
NAME Jan22-2018-nmr
EXPNO 10
PROCNO 1
Date_ 20180122
Time 7.38
INSTRUM spect
PROBHD 5 mm PABBO BB-
PULPROG zg30
TD 65536
SOLVENT DMSO
NS 64
DS 2
SWH 10330.578 Hz
FIDRES 0.157632 Hz
AQ 3.1719923 sec
RG 203
DW 48.400 usec
DE 6.50 usec
TE 294.8 K
D1 1.00000000 sec
TD0 1
======== CHANNEL f1 ========
NUC1 1H
P1 14.56 usec
PL1 3.40 dB
PL1W 12.17042828 W
SFO1 500.1330885 MHz
SI 32768
SF 500.1300000 MHz
WDW EM
SSB 0
LB 0.30 Hz
GB 0
PC 1.00

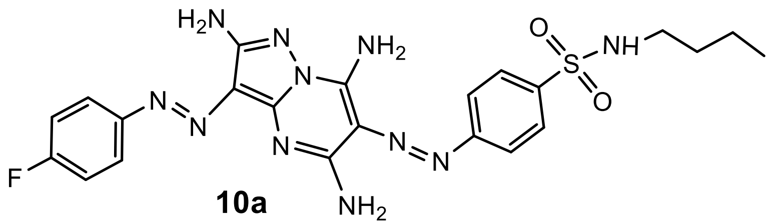
H2N
N
NH2
O
S
NH
O
N
N
F
N
N
N
N
NH2

10a

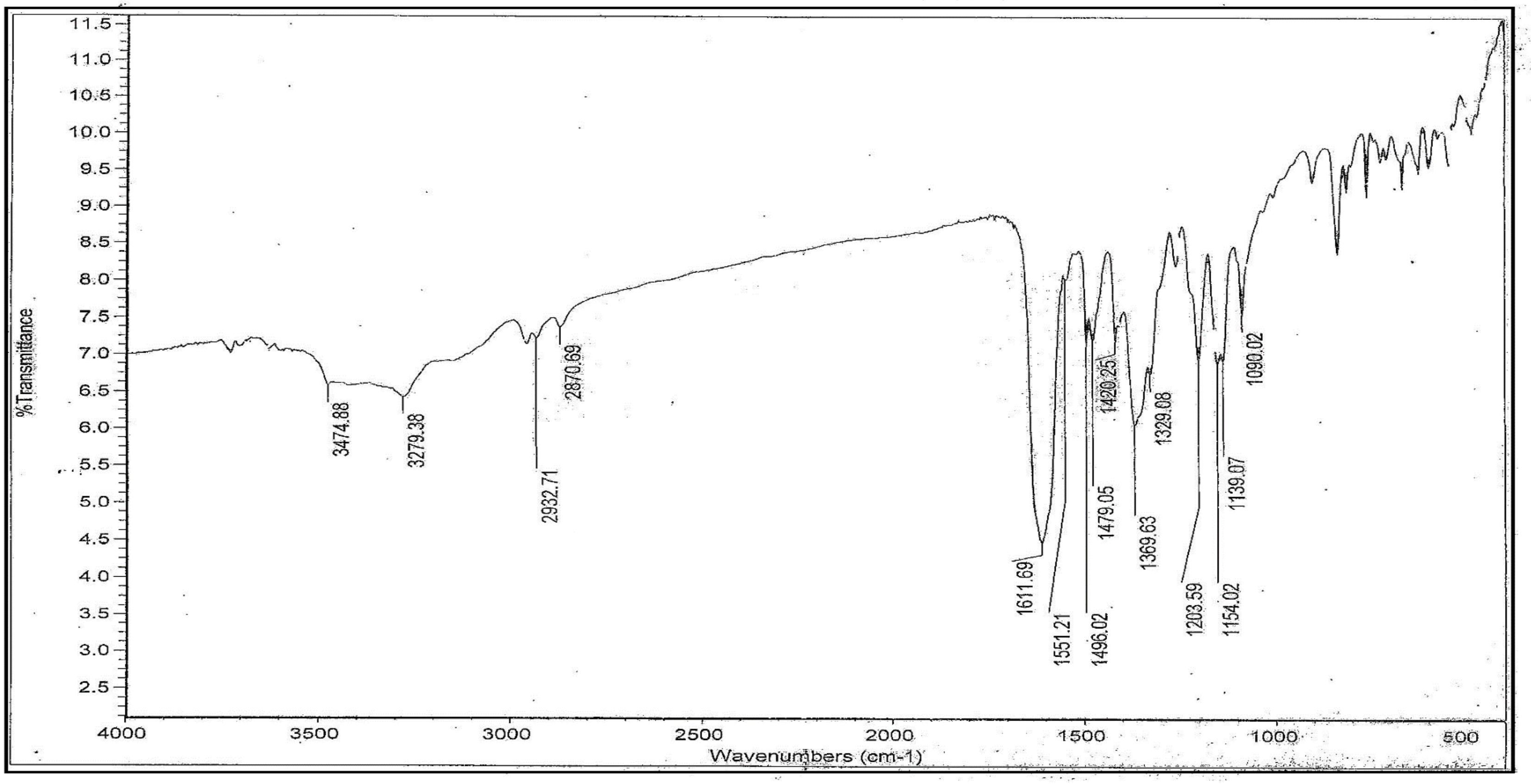
%Transmittance
Wavenumbers (cm-1)
3474.88
3279.38
2932.71
2870.69
1611.69
1551.21
1496.02
1479.05
1420.25
1369.63
1329.08
1203.59
1154.02
1139.07
1090.02
4000
3500
3000
2500
2000
1500
1000
500

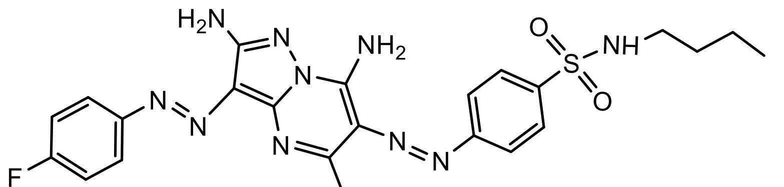
H2N
N
NH2
N
N
N
N
N
N
N
O
S
NH
O
F
NH2

10a

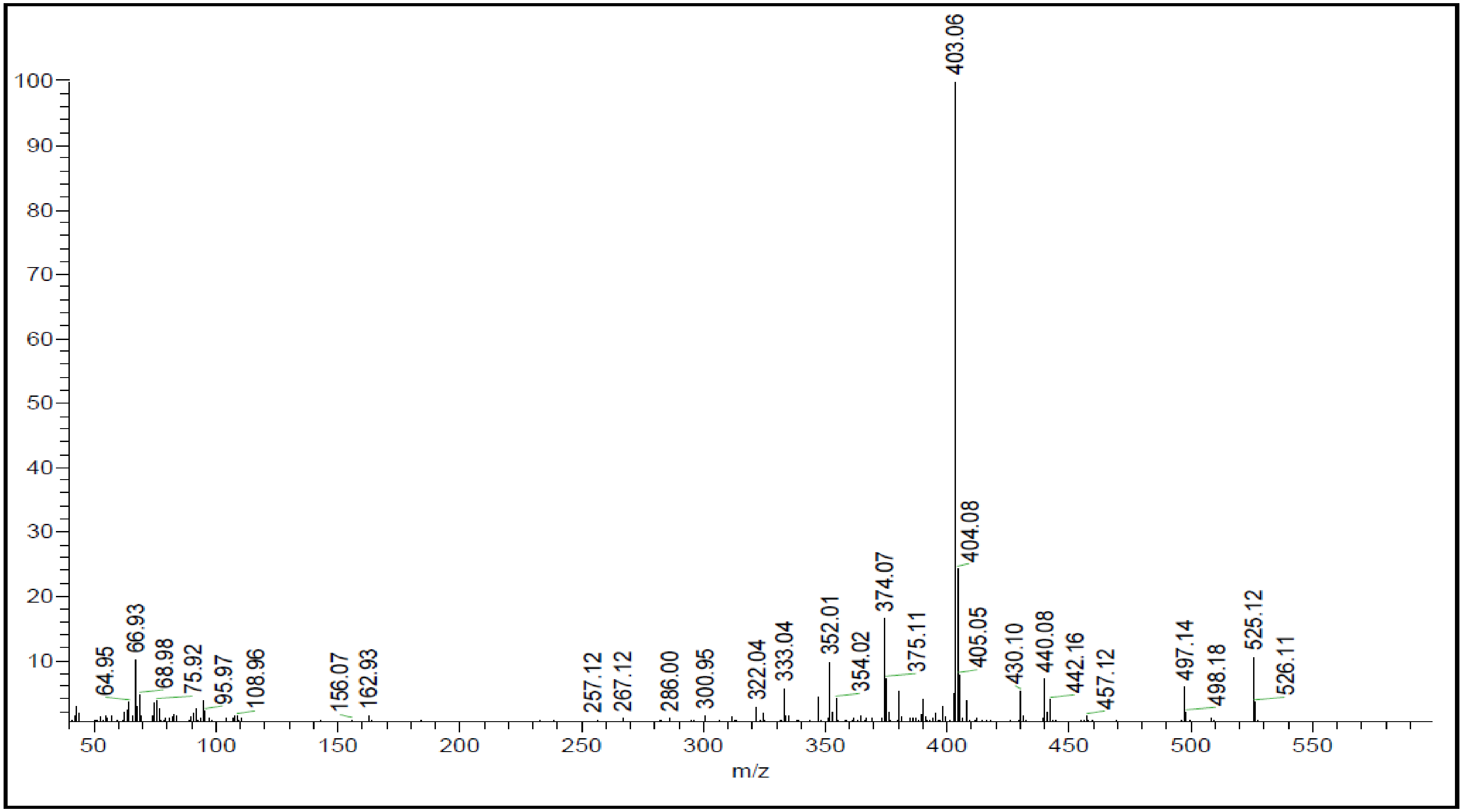
403.06
404.08
374.07
525.12
66.93
352.01
333.04
322.04
405.05
430.10
440.08
442.16
457.12
497.14
498.18
526.11
375.11
354.02
64.95
68.98
75.92
95.97
108.96
156.07
162.93
257.12
267.12
286.00
300.95
m/z

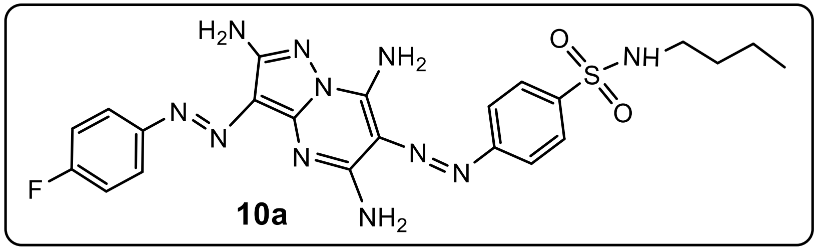

H2N
N
N
NH2
O
S
NH
O
F
N
N
N
N
N
10a
NH2

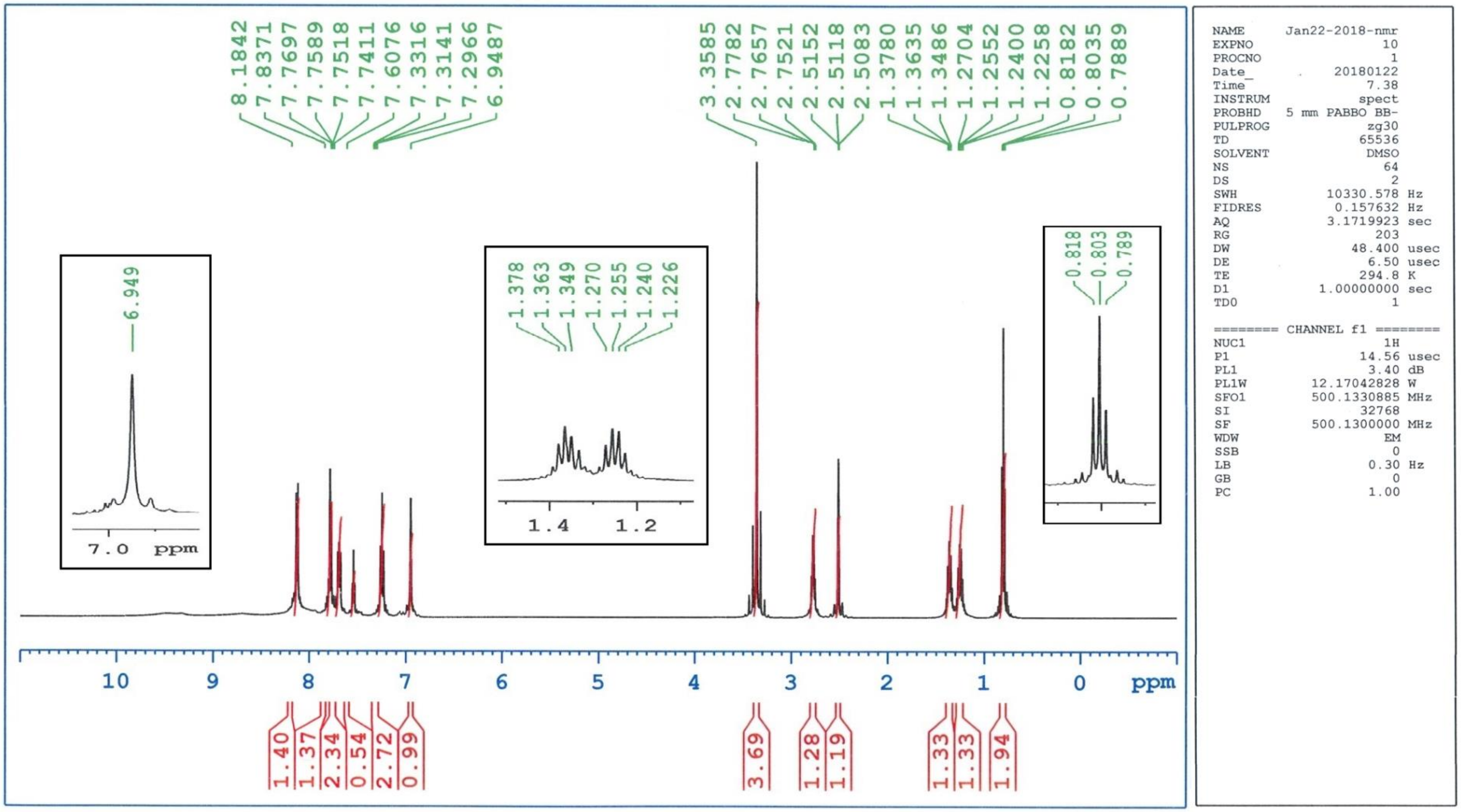

8.1842
7.8371
7.7697
7.7589
7.7518
7.7411
7.6076
7.3316
7.3141
7.2966
6.9487
3.3585
2.7782
2.7657
2.7521
2.5152
2.5118
2.5083
1.3780
1.3635
1.3486
1.2704
1.2552
1.2400
1.2258
0.8182
0.8035
0.7889
6.949
7.0 ppm
1.378
1.363
1.349
1.270
1.255
1.240
1.226
1.4
1.2
0.818
0.803
0.789
10
9
8
7
6
5
4
3
2
1
0
ppm
1.40
1.37
2.34
0.54
2.72
0.99
3.69
1.28
1.19
1.33
1.33
1.94
NAME Jan22-2018-nmr
EXPNO 10
PROCNO 1
Date_ 20180122
Time 7.38
INSTRUM spect
PROBHD 5 mm PABBO BB-
PULPROG zg30
TD 65536
SOLVENT DMSO
NS 64
DS 2
SWH 10330.578 Hz
FIDRES 0.157632 Hz
AQ 3.1719923 sec
RG 203
DW 48.400 usec
DE 6.50 usec
TE 294.8 K
D1 1.00000000 sec
TD0 1
======== CHANNEL f1 ========
NUC1 1H
P1 14.56 usec
PL1 3.40 dB
PL1W 12.17042828 W
SFO1 500.1330885 MHz
SI 32768
SF 500.1300000 MHz
WDW EM
SSB 0
LB 0.30 Hz
GB 0
PC 1.00

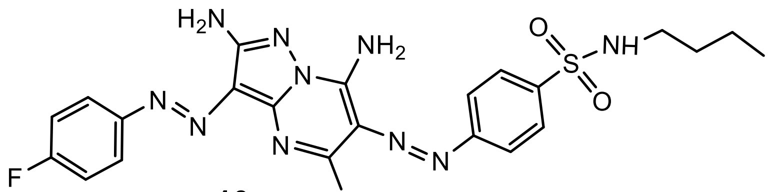

10a

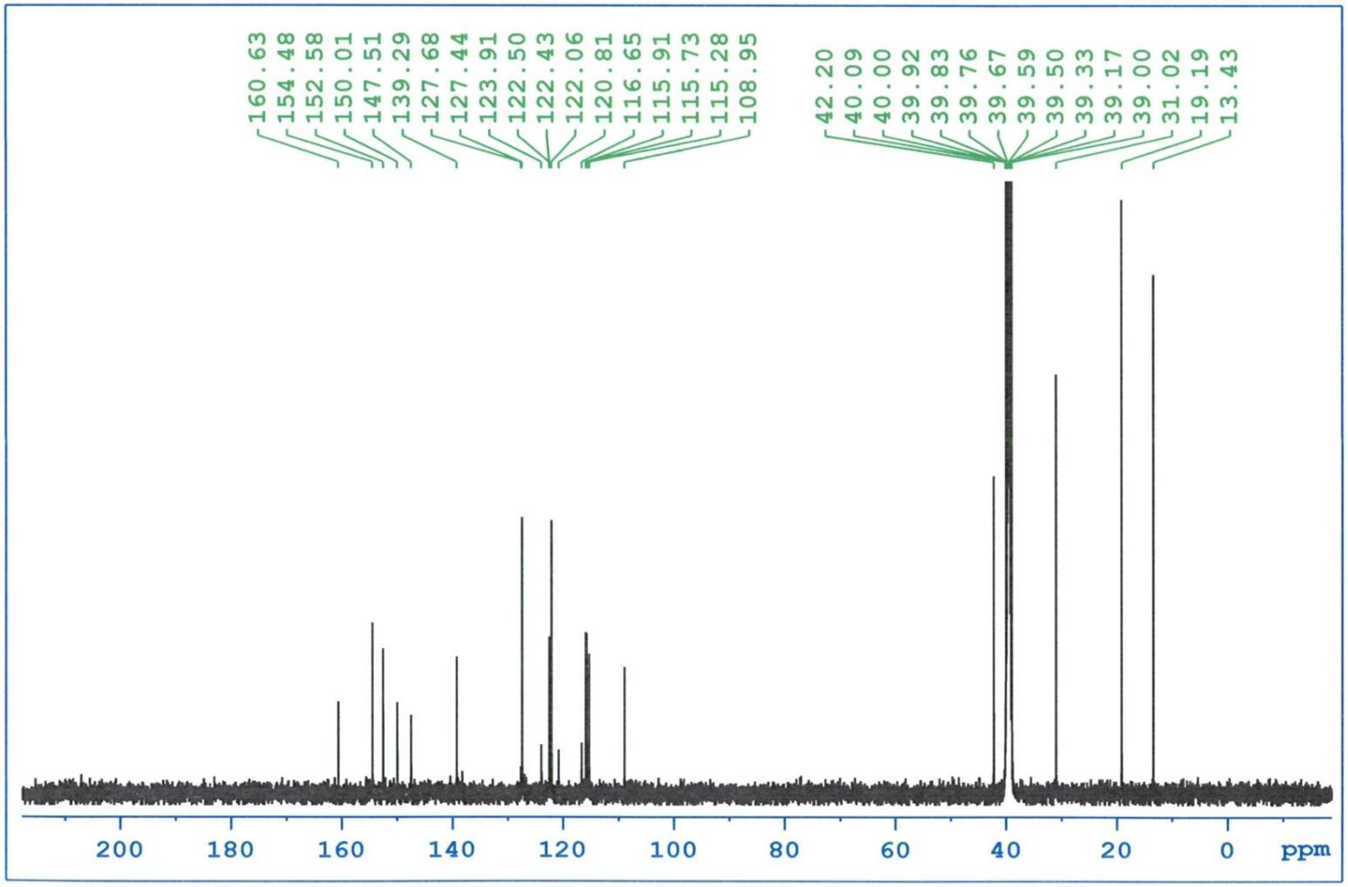

NAME	Jan22-2018-nmr	
EXPNO	10	
PROCNO	1	
Date_	20180122	
Time	7.38	
INSTRUM	spect	
PROBHD	5 mm PABBO BB-	
PULPROG	zg30	
TD	65536	
SOLVENT	DMSO	
NS	64	
DS	2	
SWH	10330.578	Hz
FIDRES	0.157632	Hz
AQ	3.1719923	sec
RG	203	
DW	48.400	usec
DE	6.50	usec
TE	294.8	K
D1	1.00000000	sec
TD0	1	
======== CHANNEL f1 ========		
NUC1	1H	
P1	14.56	usec
PL1	3.40	dB
PL1W	12.17042828	W
SFO1	500.1330885	MHz
SI	32768	
SF	500.1300000	MHz
WDW	EM	
SSB	0	
LB	0.30	Hz
GB	0	
PC	1.00	

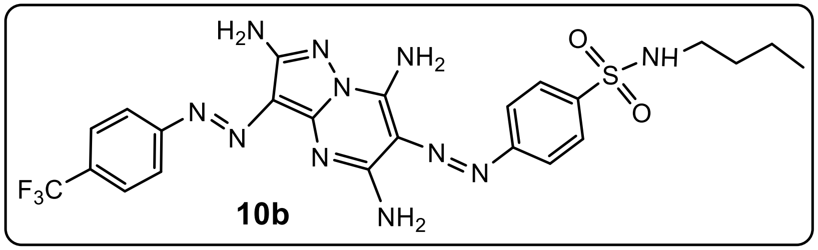

H_2N
N
N
NH_2
O
S
NH
O
N
N
N
N
F_3C
N
N
NH_2

10b

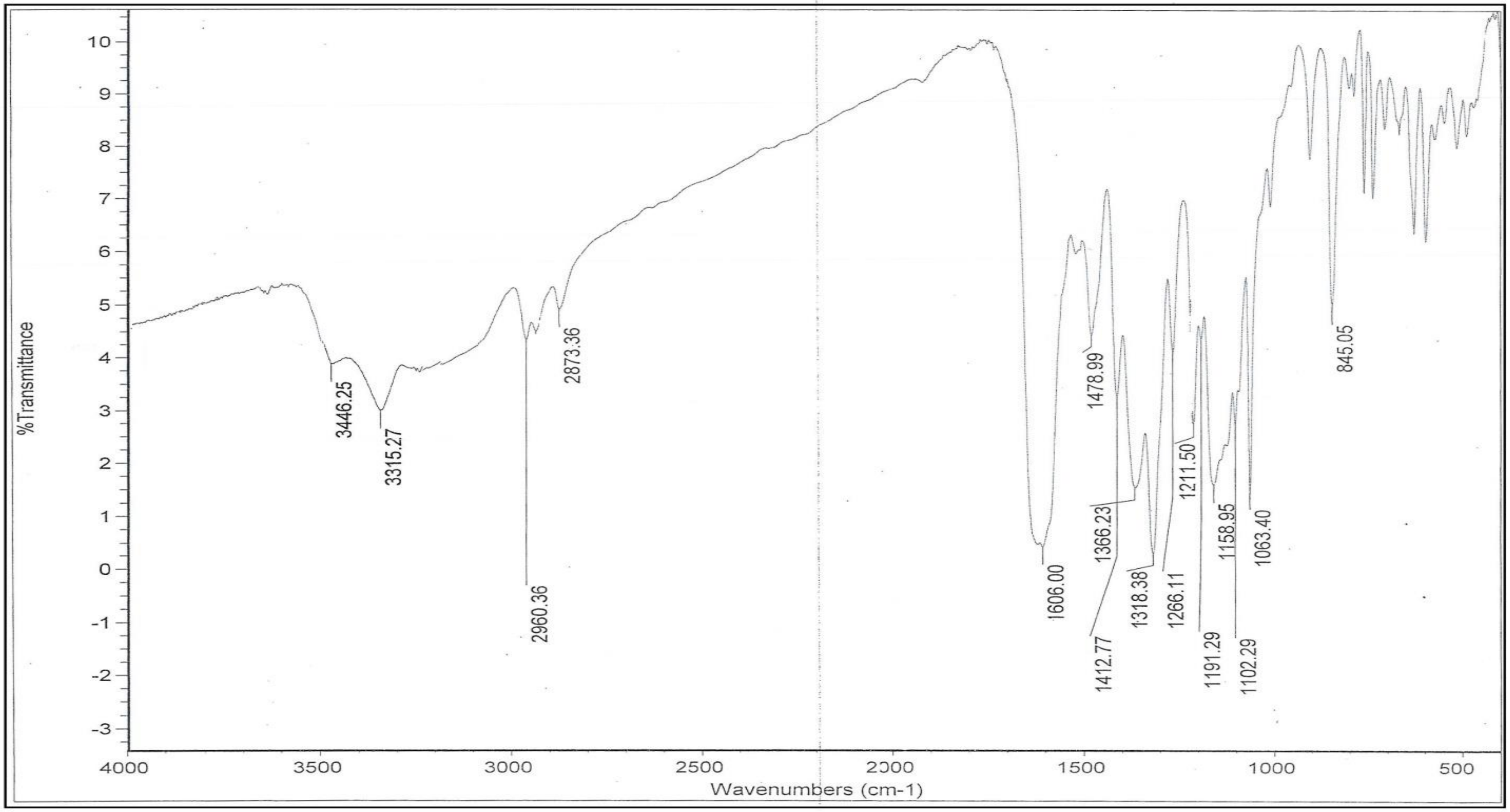

%Transmittance
Wavenumbers (cm-1)
3446.25
3315.27
2960.36
2873.36
1606.00
1478.99
1412.77
1366.23
1318.38
1266.11
1211.50
1191.29
1158.95
1102.29
1063.40
845.05

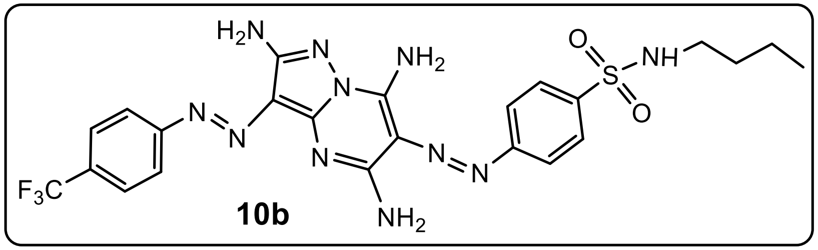
H2N
N
N
NH2
O
S
NH
O
N
N
N
N
F3C
N
N
10b
NH2

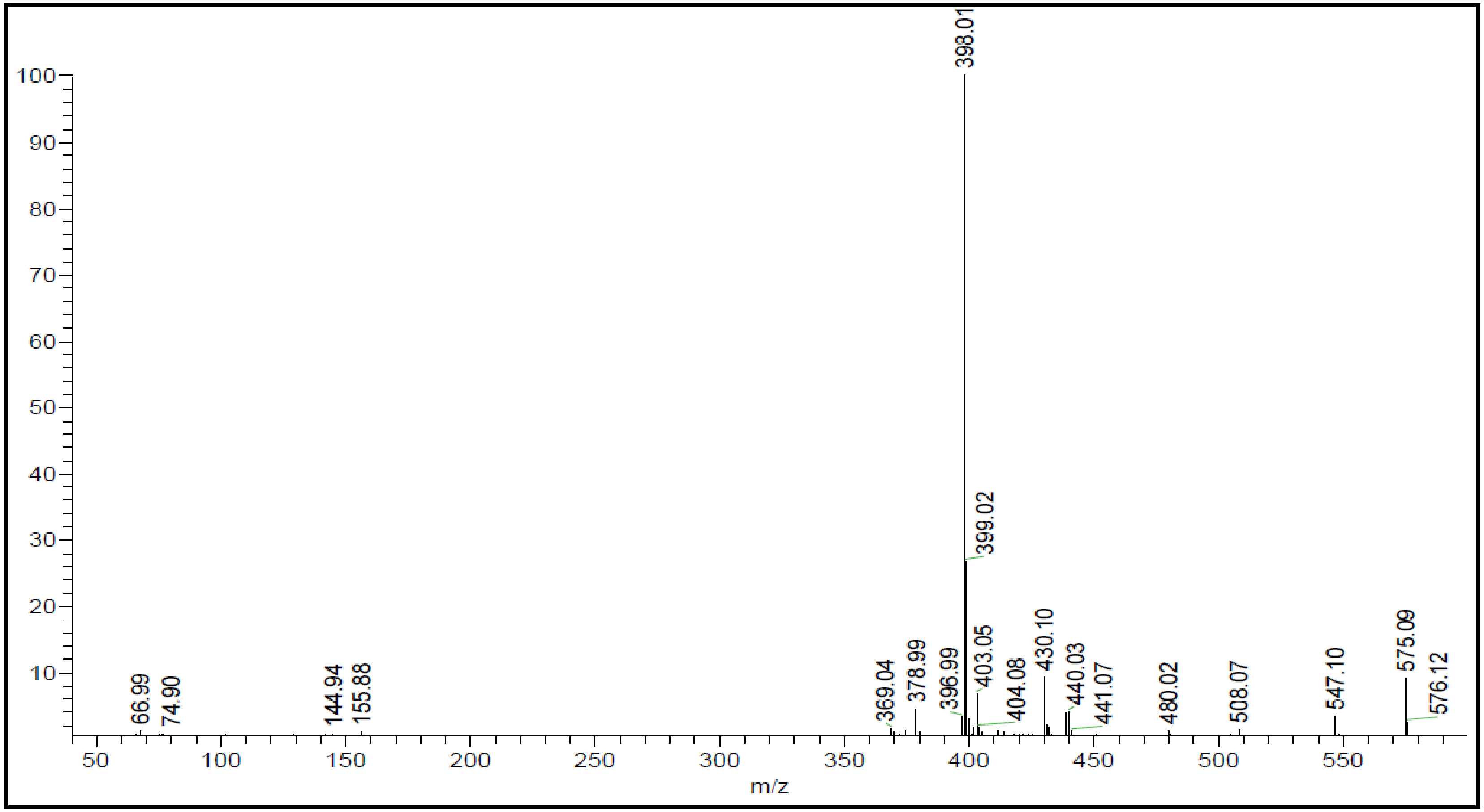
398.01
399.02
66.99
74.90
144.94
155.88
369.04
378.99
396.99
403.05
404.08
430.10
440.03
441.07
480.02
508.07
547.10
575.09
576.12
100
90
80
70
60
50
40
30
20
10
50
100
150
200
250
300
350
400
450
500
550
m/z

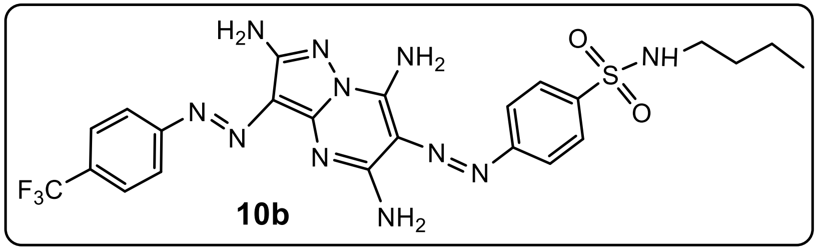

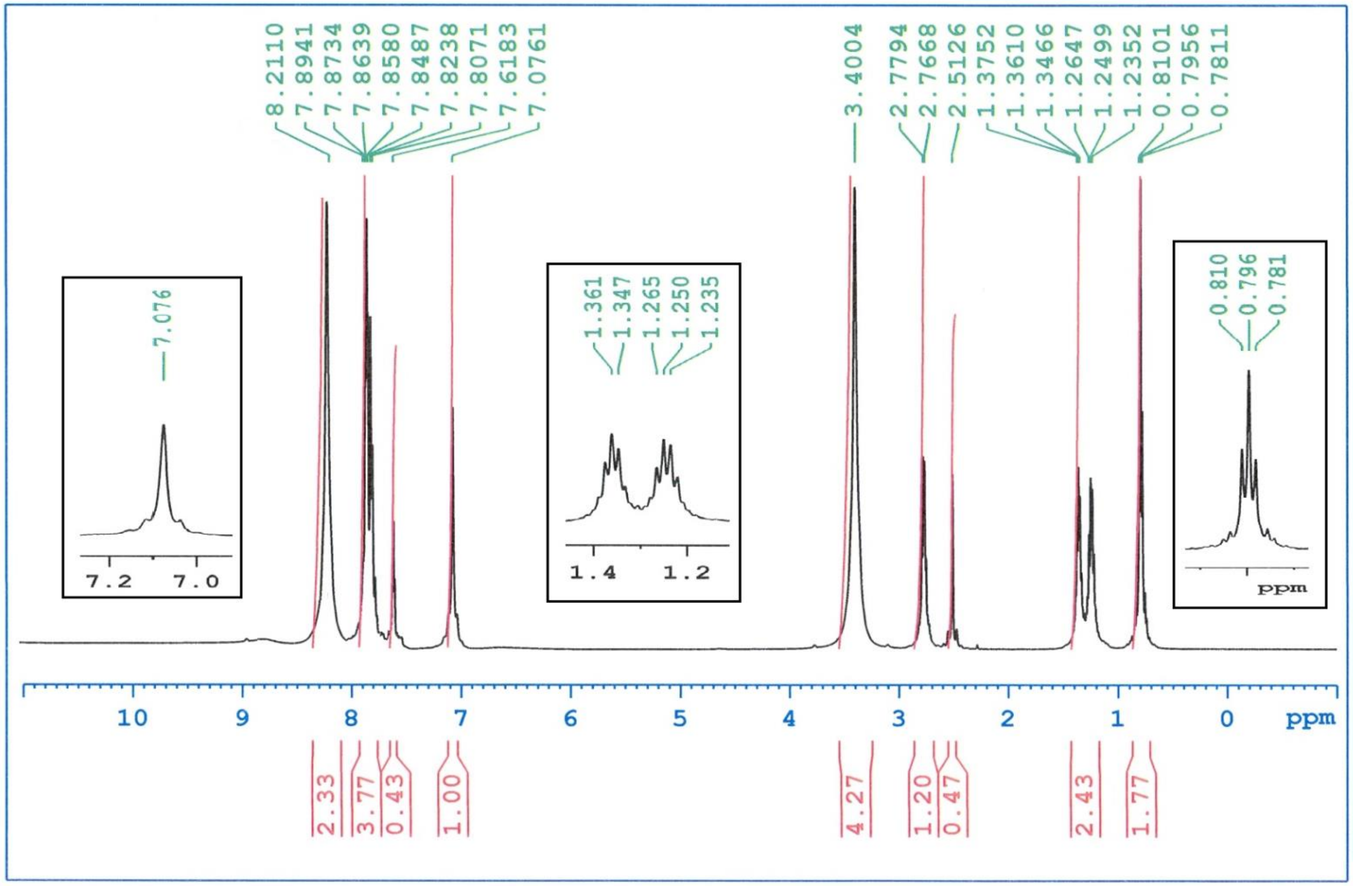

NAME	Jan22-2018-nmr
EXPNO	10
PROCNO	1
Date_	20180122
Time	7.38
INSTRUM	spect
PROBHD	5 mm PABBO BB-
PULPROG	zg30
TD	65536
SOLVENT	DMSO
NS	64
DS	2
SWH	10330.578 Hz
FIDRES	0.157632 Hz
AQ	3.1719923 sec
RG	203
DW	48.400 usec
DE	6.50 usec
TE	294.8 K
D1	1.00000000 sec
TD0	1

======== CHANNEL f1 ========

NUC1	1H
P1	14.56 usec
PL1	3.40 dB
PL1W	12.17042828 W
SFO1	500.1330885 MHz
SI	32768
SF	500.1300000 MHz
WDW	EM
SSB	0
LB	0.30 Hz
GB	0
PC	1.00

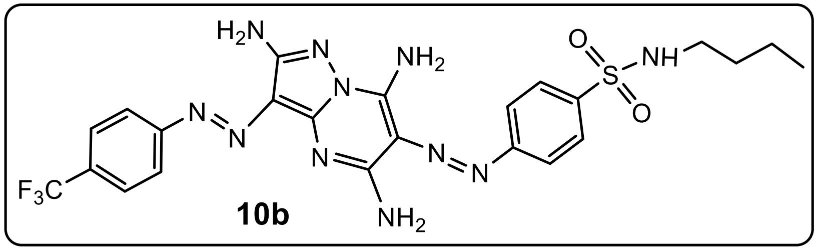

10b

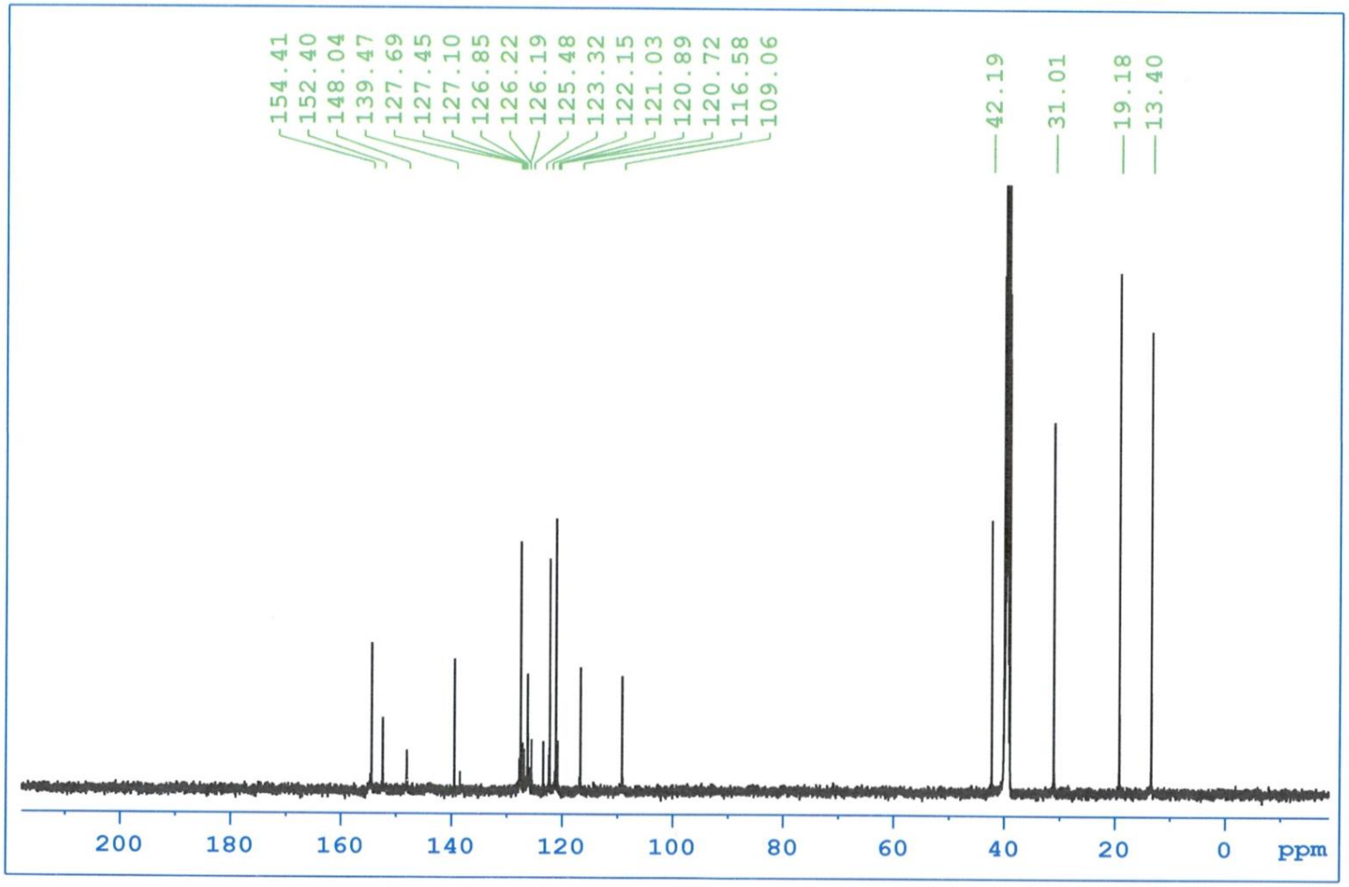

NAME	Jan22-2018-nmr
EXPNO	10
PROCNO	1
Date_	20180122
Time	7.38
INSTRUM	spect
PROBHD	5 mm PABBO BB-
PULPROG	zg30
TD	65536
SOLVENT	DMSO
NS	64
DS	2
SWH	10330.578 Hz
FIDRES	0.157632 Hz
AQ	3.1719923 sec
RG	203
DW	48.400 usec
DE	6.50 usec
TE	294.8 K
D1	1.00000000 sec
TD0	1
======== CHANNEL f1 ========	
NUC1	1H
P1	14.56 usec
PL1	3.40 dB
PL1W	12.17042828 W
SFO1	500.1330885 MHz
SI	32768
SF	500.1300000 MHz
WDW	EM
SSB	0
LB	0.30 Hz
GB	0
PC	1.00

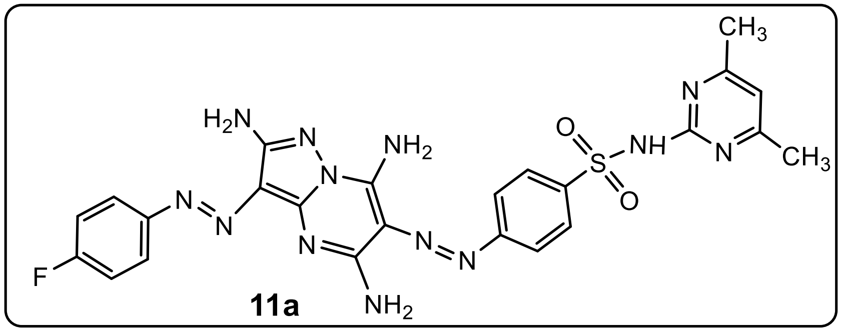

11a

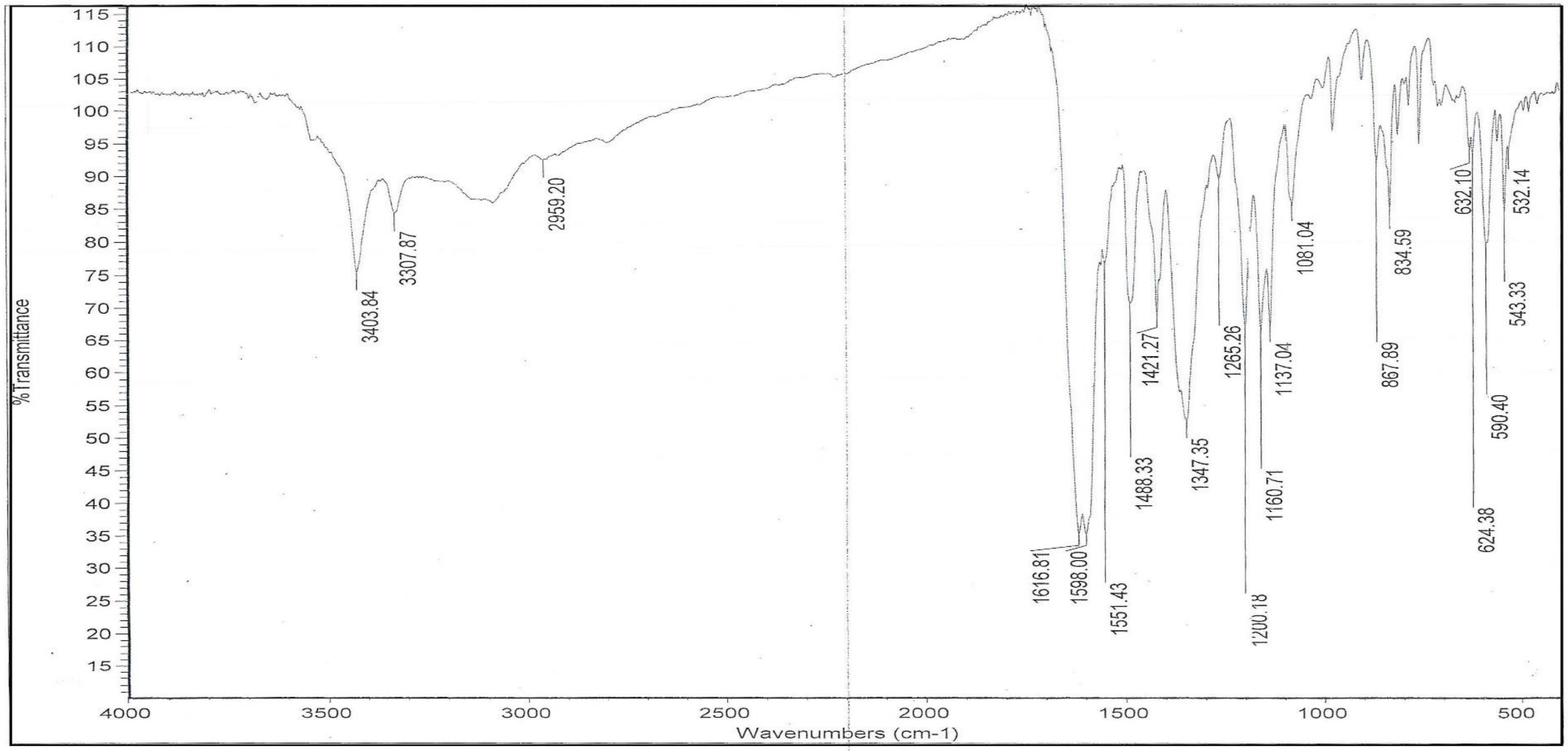
%Transmittance
Wavenumbers (cm-1)
3403.84
3307.87
2959.20
1616.81
1598.00
1551.43
1488.33
1421.27
1347.35
1265.26
1200.18
1160.71
1137.04
1081.04
867.89
834.59
632.10
624.38
590.40
543.33
532.14

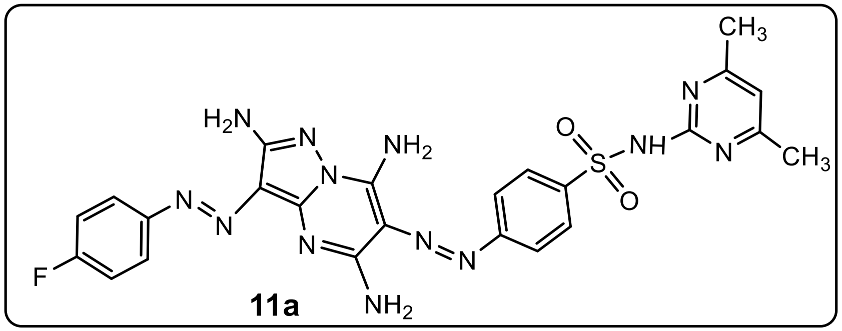

11a

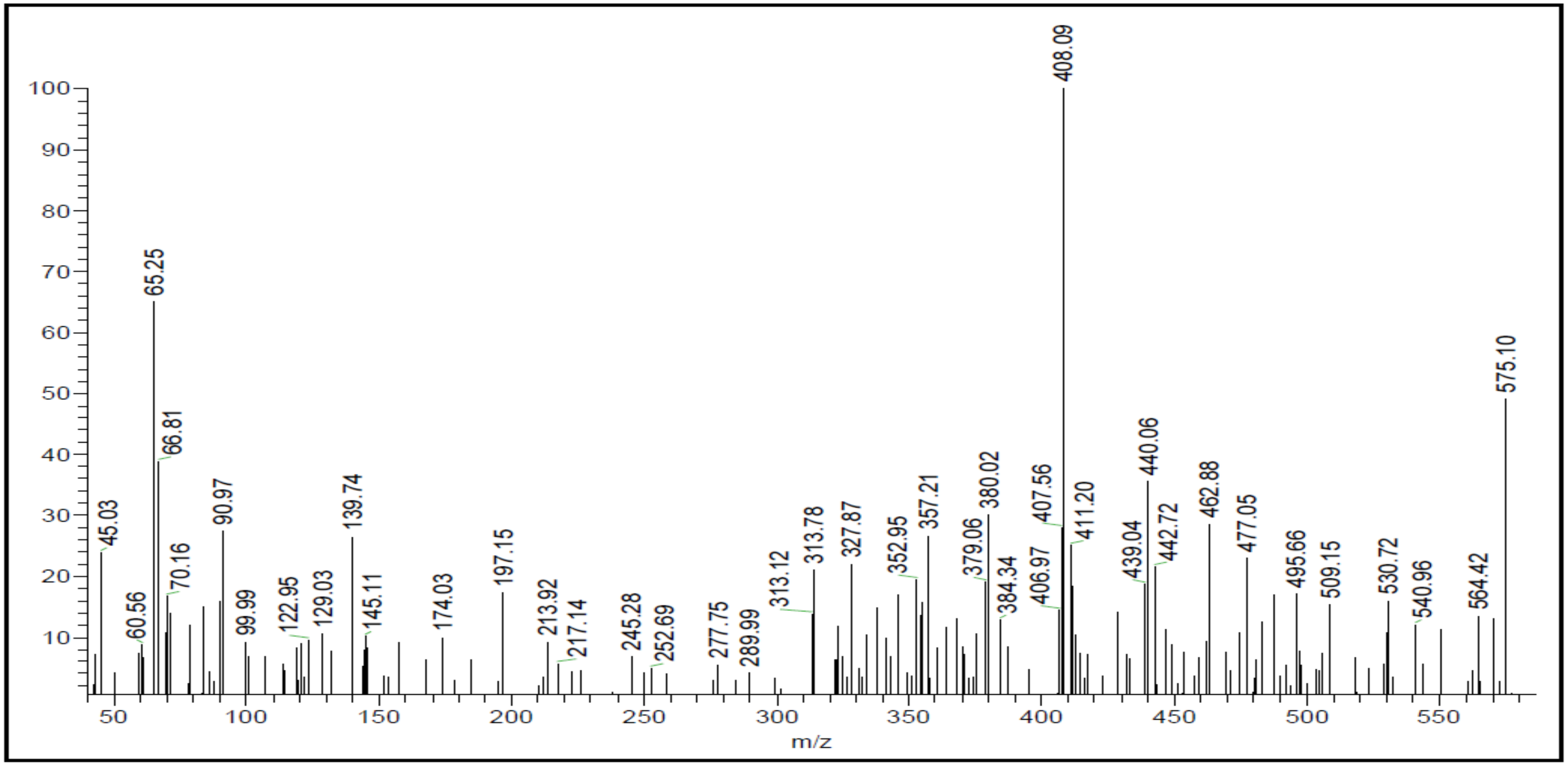

408.09
65.25
575.10
66.81
440.06
462.88
45.03
90.97
139.74
380.02
357.21
407.56
411.20
327.87
313.78
477.05
70.16
197.15
352.95
379.06
439.04
442.72
495.66
509.15
530.72
540.96
564.42
60.56
99.99
122.95
129.03
145.11
174.03
213.92
217.14
245.28
252.69
277.75
289.99
313.12
384.34
406.97
m/z

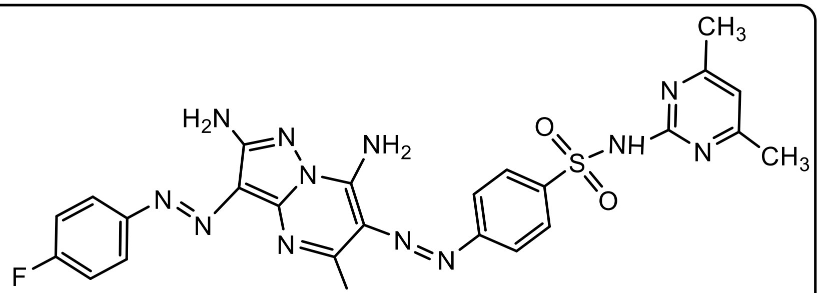

11a

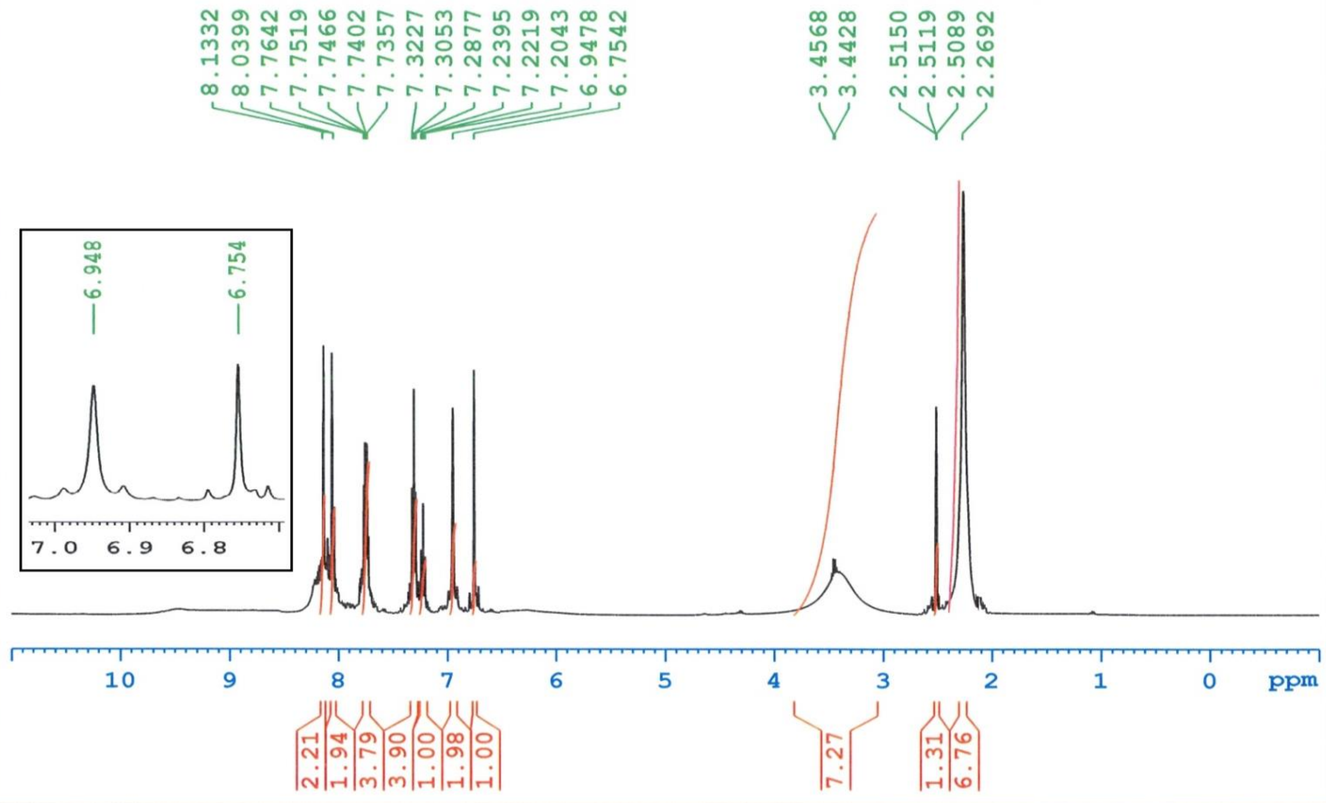

NAME	Jan22-2018-nmr
EXPNO	10
PROCNO	1
Date_	20180122
Time	7.38
INSTRUM	spect
PROBHD	5 mm PABBO BB-
PULPROG	zg30
TD	65536
SOLVENT	DMSO
NS	64
DS	2
SWH	10330.578 Hz
FIDRES	0.157632 Hz
AQ	3.1719923 sec
RG	203
DW	48.400 usec
DE	6.50 usec
TE	294.8 K
D1	1.00000000 sec
TD0	1
======== CHANNEL f1 ========	
NUC1	1H
P1	14.56 usec
PL1	3.40 dB
PL1W	12.17042828 W
SFO1	500.1330885 MHz
SI	32768
SF	500.1300000 MHz
WDW	EM
SSB	0
LB	0.30 Hz
GB	0
PC	1.00

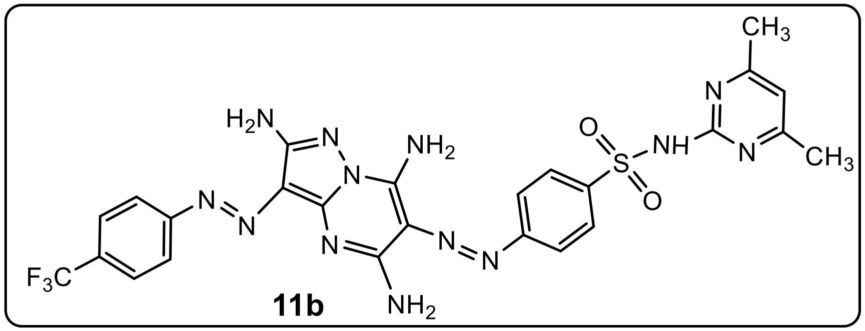
H2N
N
N
NH2
O
S
NH
N
N
CH3
CH3
O
N
N
N
N
N
N
F3C
11b
NH2

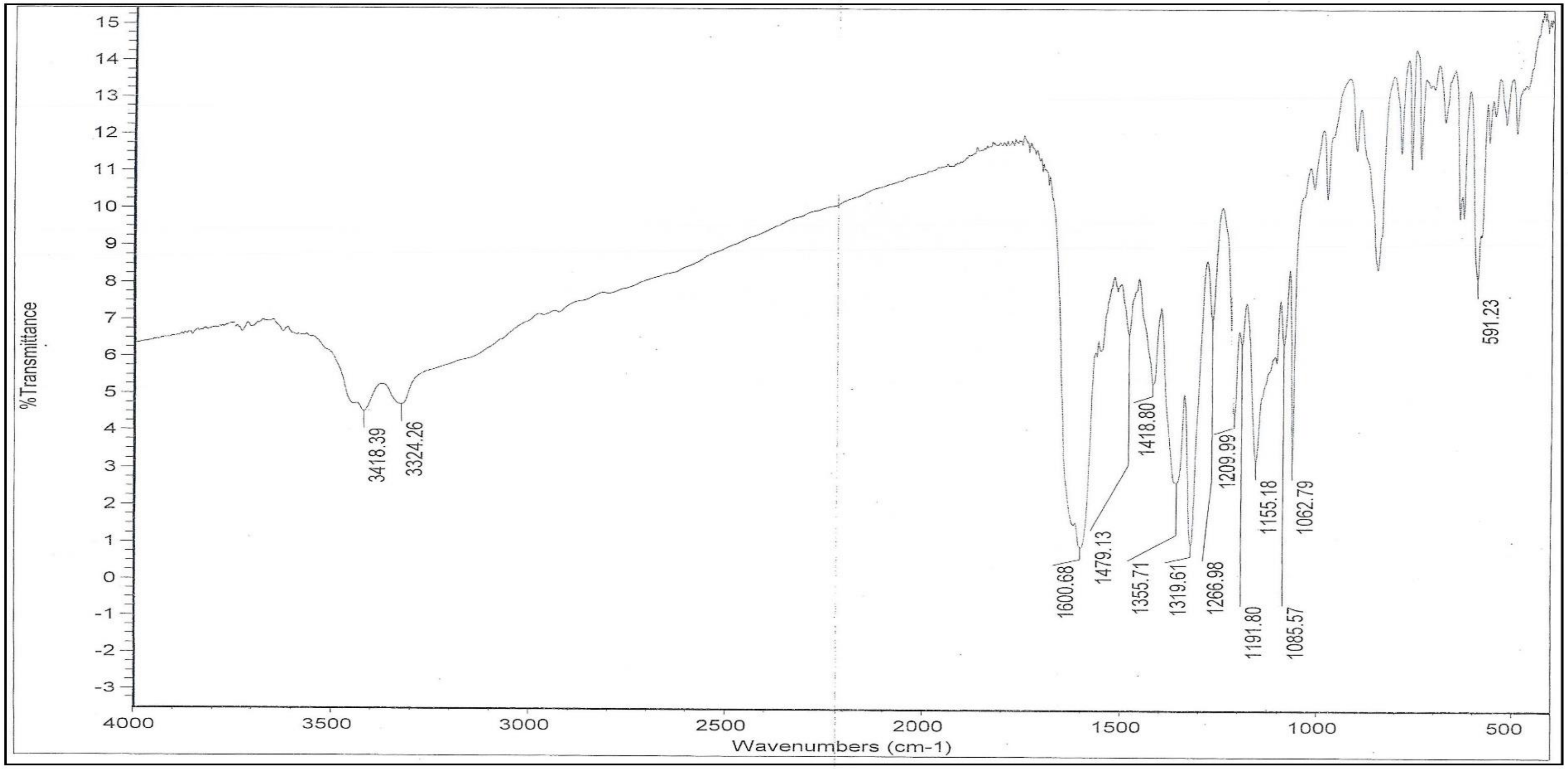
%Transmittance
Wavenumbers (cm-1)
3418.39
3324.26
1600.68
1479.13
1418.80
1355.71
1319.61
1266.98
1209.99
1191.80
1155.18
1085.57
1062.79
591.23

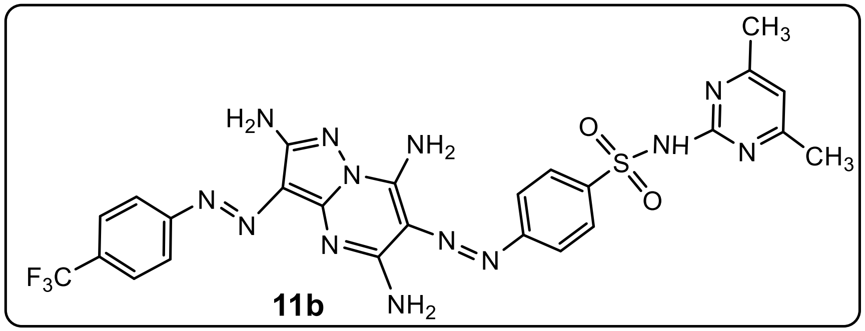

11b

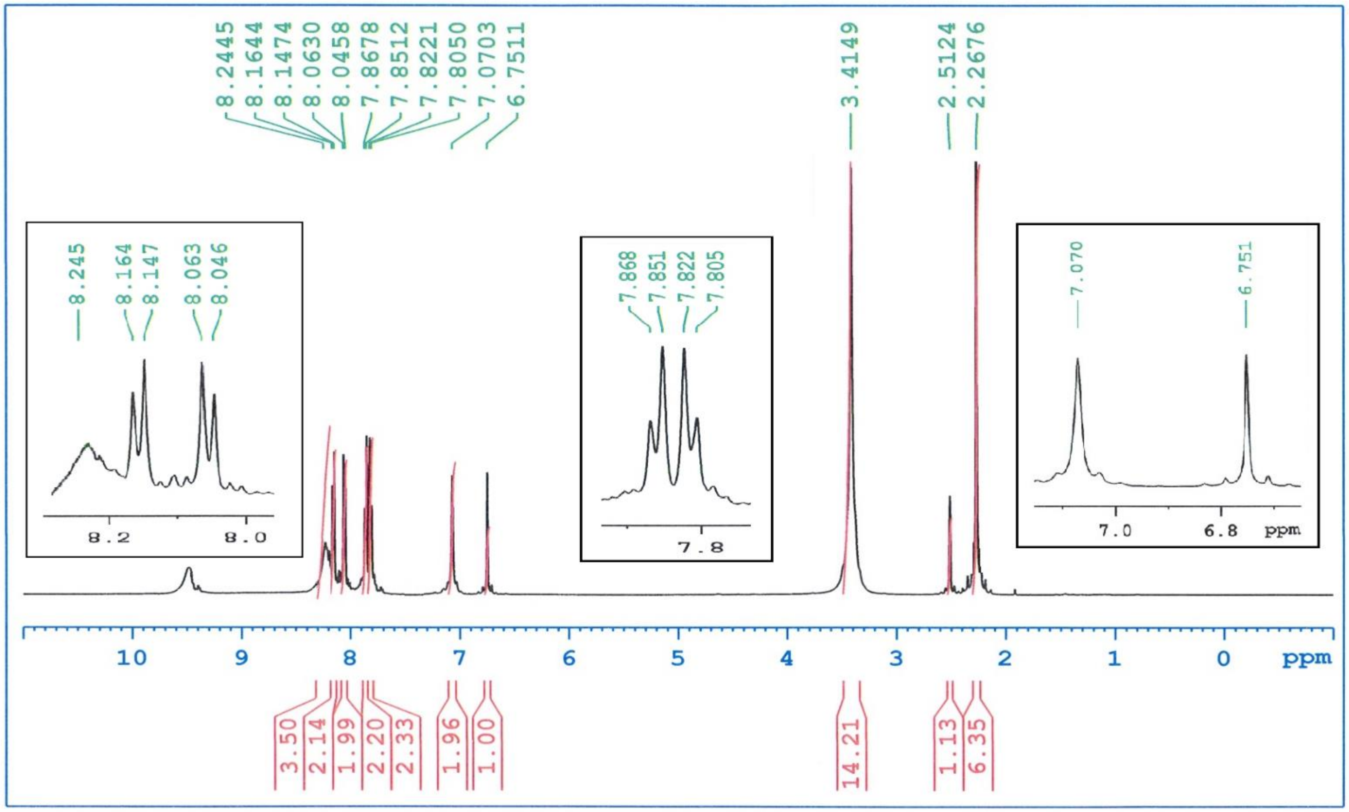

NAME	Jan22-2018-nmr	
EXPNO	10	
PROCNO	1	
Date_	20180122	
Time	7.38	
INSTRUM	spect	
PROBHD	5 mm PABBO BB-	
PULPROG	zg30	
TD	65536	
SOLVENT	DMSO	
NS	64	
DS	2	
SWH	10330.578	Hz
FIDRES	0.157632	Hz
AQ	3.1719923	sec
RG	203	
DW	48.400	usec
DE	6.50	usec
TE	294.8	K
D1	1.00000000	sec
TD0	1	
======== CHANNEL f1 ========		
NUC1	1H	
P1	14.56	usec
PL1	3.40	dB
PL1W	12.17042828	W
SFO1	500.1330885	MHz
SI	32768	
SF	500.1300000	MHz
WDW	EM	
SSB	0	
LB	0.30	Hz
GB	0	
PC	1.00	

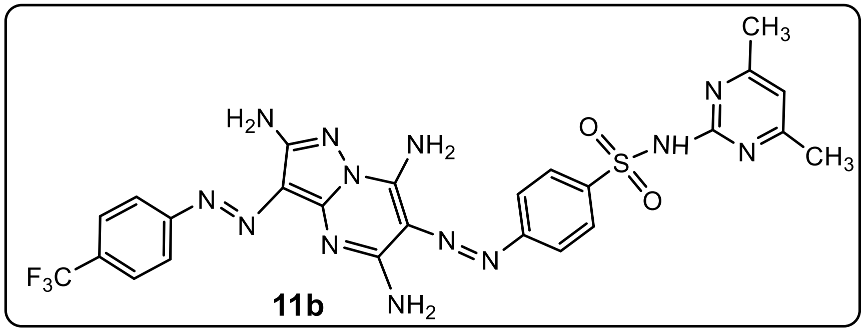

11b

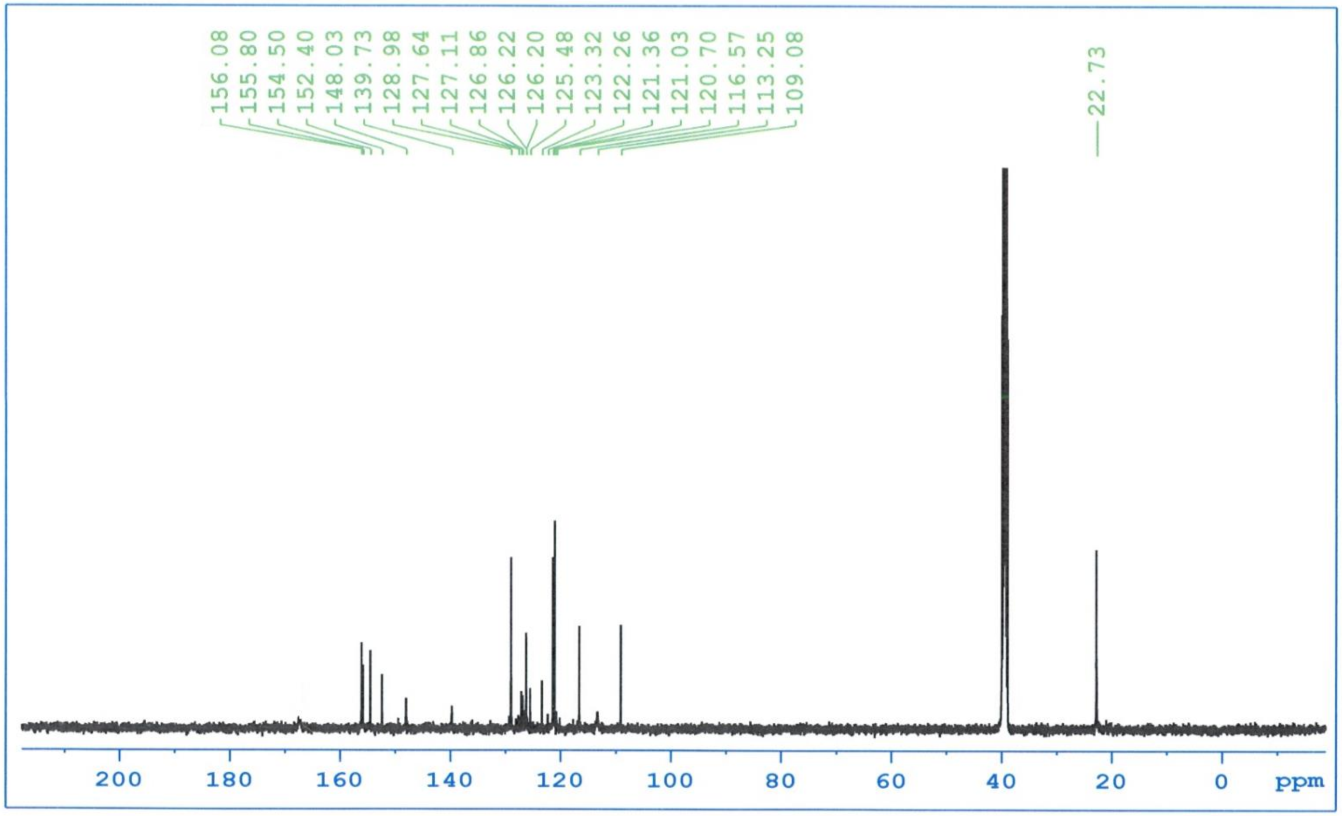

NAME Jan22-2018-nmr
EXPNO 10
PROCNO 1
Date_ 20180122
Time 7.38
INSTRUM spect
PROBHD 5 mm PABBO BB-
PULPROG zg30
TD 65536
SOLVENT DMSO
NS 64
DS 2
SWH 10330.578 Hz
FIDRES 0.157632 Hz
AQ 3.1719923 sec
RG 203
DW 48.400 usec
DE 6.50 usec
TE 294.8 K
D1 1.00000000 sec
TD0 1

======== CHANNEL f1 ========
NUC1 1H
P1 14.56 usec
PL1 3.40 dB
PL1W 12.17042828 W
SFO1 500.1330885 MHz
SI 32768
SF 500.1300000 MHz
WDW EM
SSB 0
LB 0.30 Hz
GB 0
PC 1.00

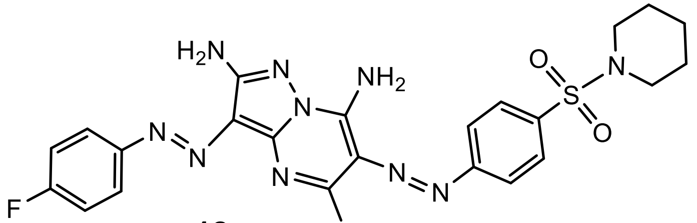

H_2N
NH_2
NH_2
F
O
S
O
N

12a

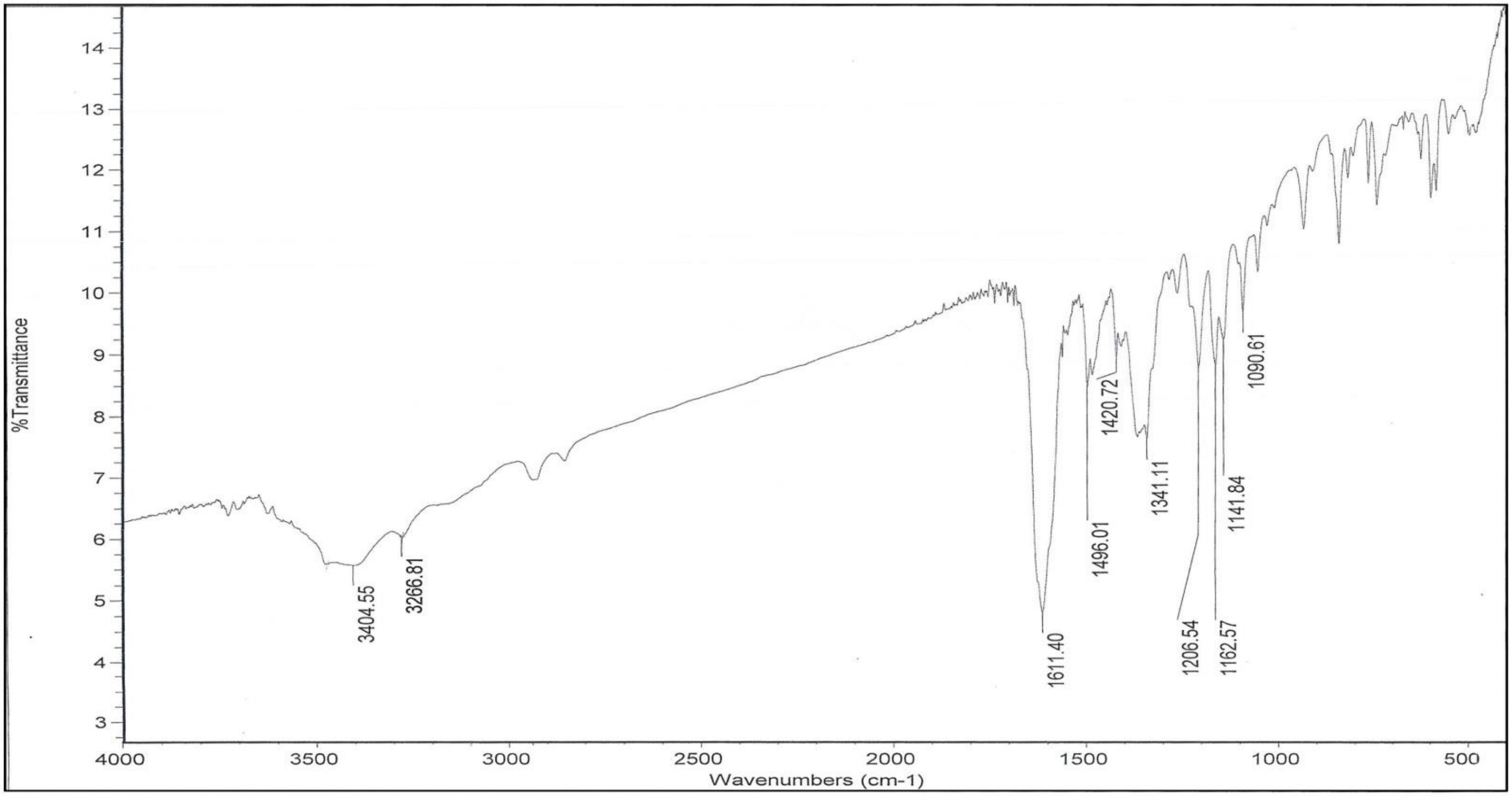

%Transmittance
Wavenumbers (cm-1)
3404.55
3266.81
1611.40
1496.01
1420.72
1341.11
1206.54
1162.57
1141.84
1090.61

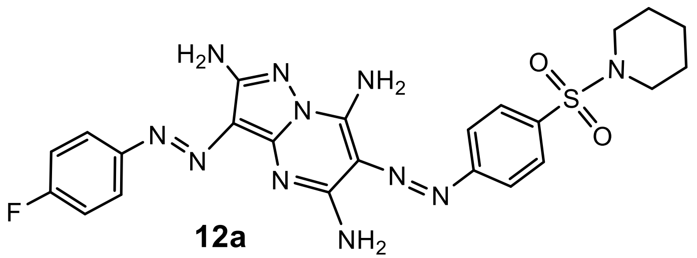

12a

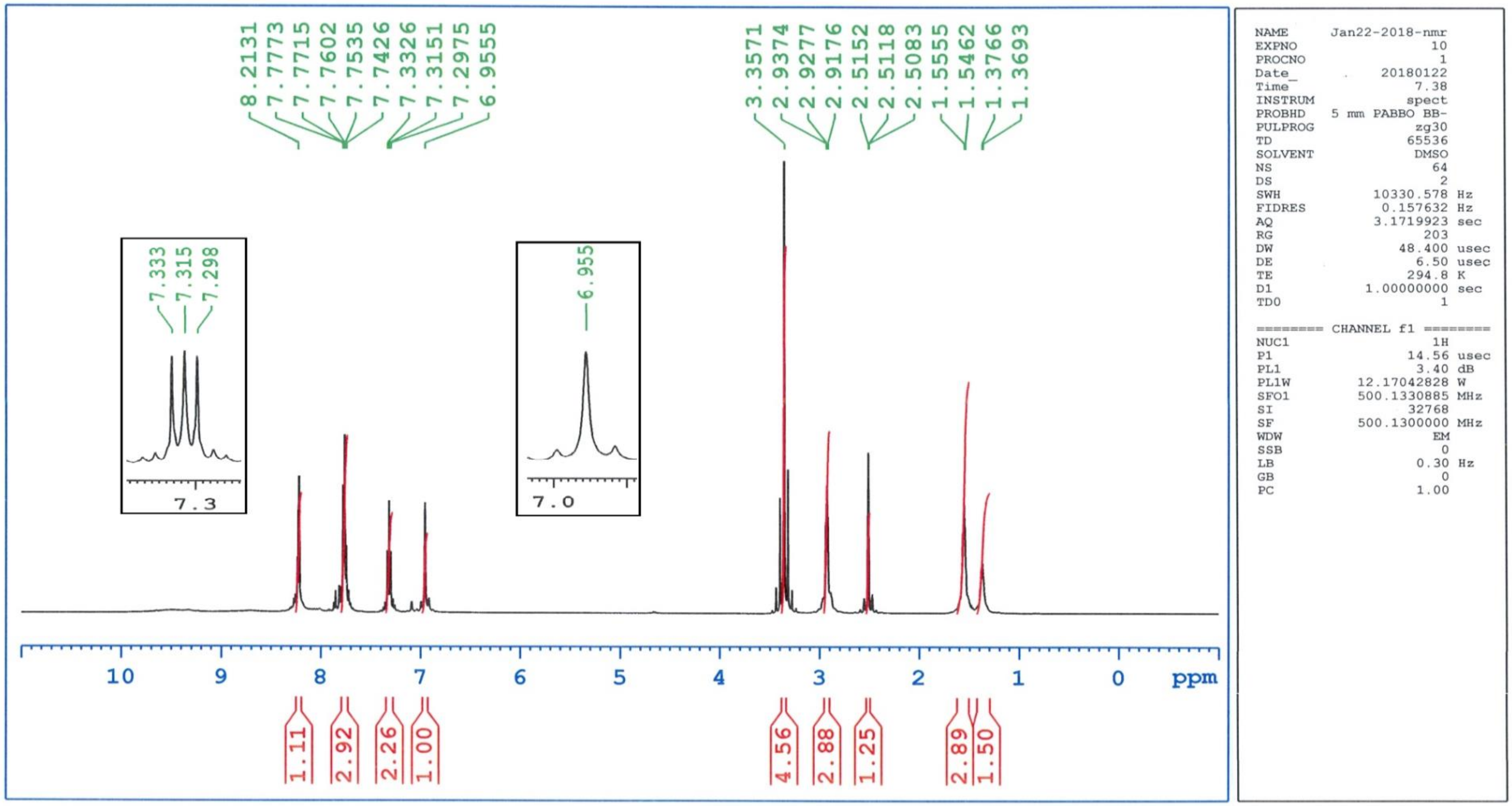

NAME Jan22-2018-nmr
EXPNO 10
PROCNO 1
Date_ 20180122
Time 7.38
INSTRUM spect
PROBHD 5 mm PABBO BB-
PULPROG zg30
TD 65536
SOLVENT DMSO
NS 64
DS 2
SWH 10330.578 Hz
FIDRES 0.157632 Hz
AQ 3.1719923 sec
RG 203
DW 48.400 usec
DE 6.50 usec
TE 294.8 K
D1 1.00000000 sec
TD0 1

======== CHANNEL f1 ========
NUC1 1H
P1 14.56 usec
PL1 3.40 dB
PL1W 12.17042828 W
SFO1 500.1330885 MHz
SI 32768
SF 500.1300000 MHz
WDW EM
SSB 0
LB 0.30 Hz
GB 0
PC 1.00

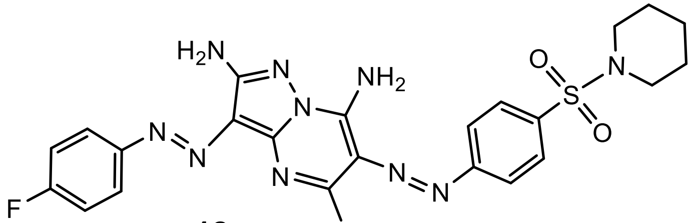

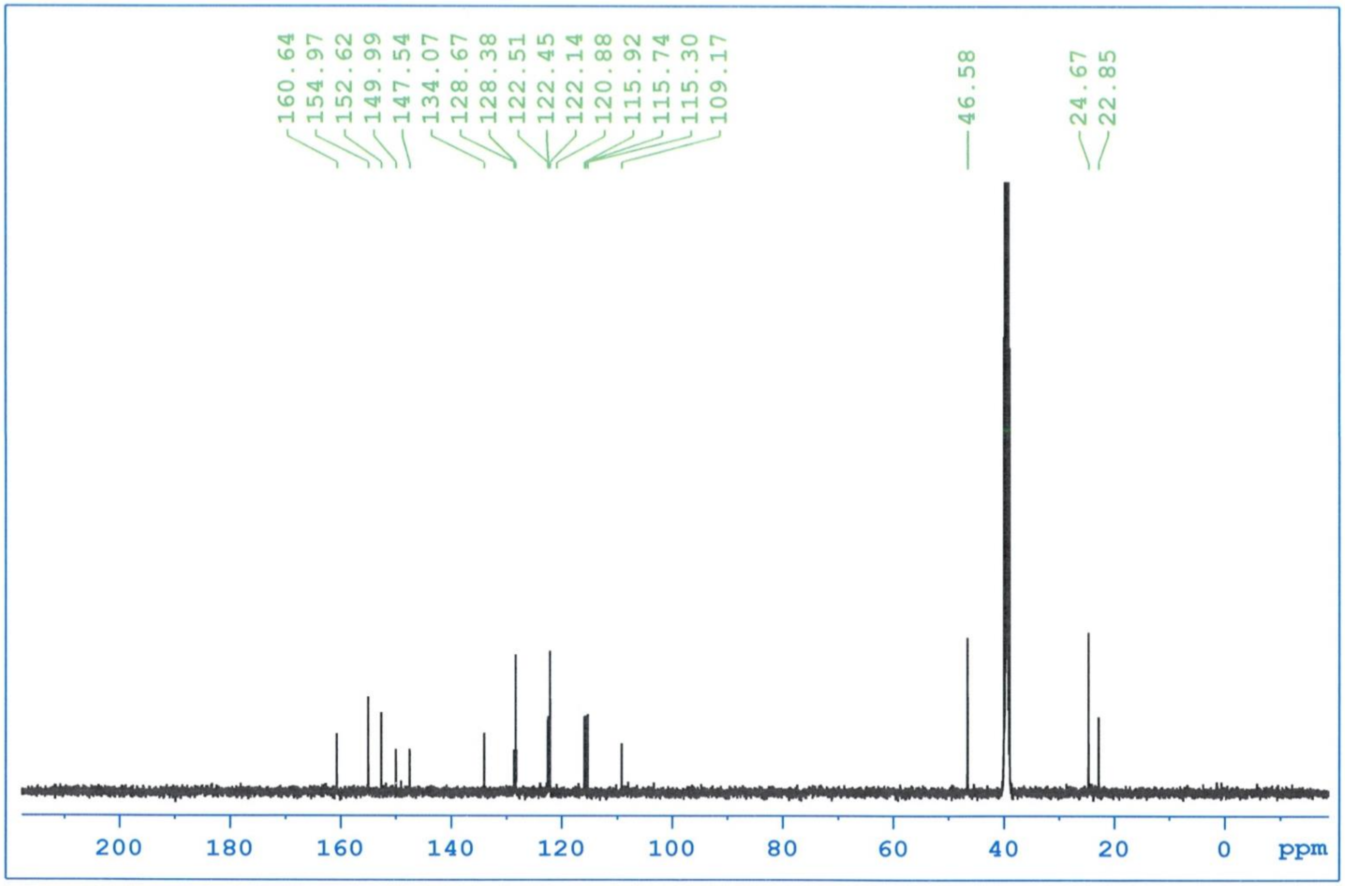

NAME	Jan22-2018-nmr
EXPNO	10
PROCNO	1
Date_	20180122
Time	7.38
INSTRUM	spect
PROBHD	5 mm PABBO BB-
PULPROG	zg30
TD	65536
SOLVENT	DMSO
NS	64
DS	2
SWH	10330.578 Hz
FIDRES	0.157632 Hz
AQ	3.1719923 sec
RG	203
DW	48.400 usec
DE	6.50 usec
TE	294.8 K
D1	1.00000000 sec
TD0	1

======== CHANNEL f1 ========

NUC1	1H
P1	14.56 usec
PL1	3.40 dB
PL1W	12.17042828 W
SFO1	500.1330885 MHz
SI	32768
SF	500.1300000 MHz
WDW	EM
SSB	0
LB	0.30 Hz
GB	0
PC	1.00

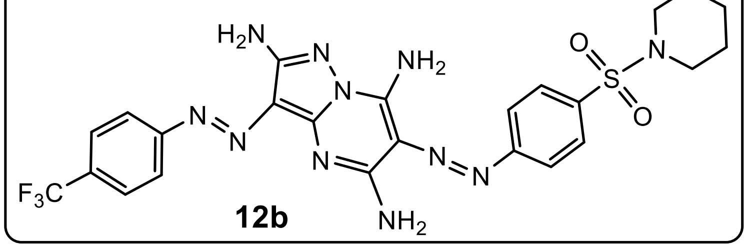

H2N
NH2
NH2
N=N
N=N
F3C
O
S
O
N

12b

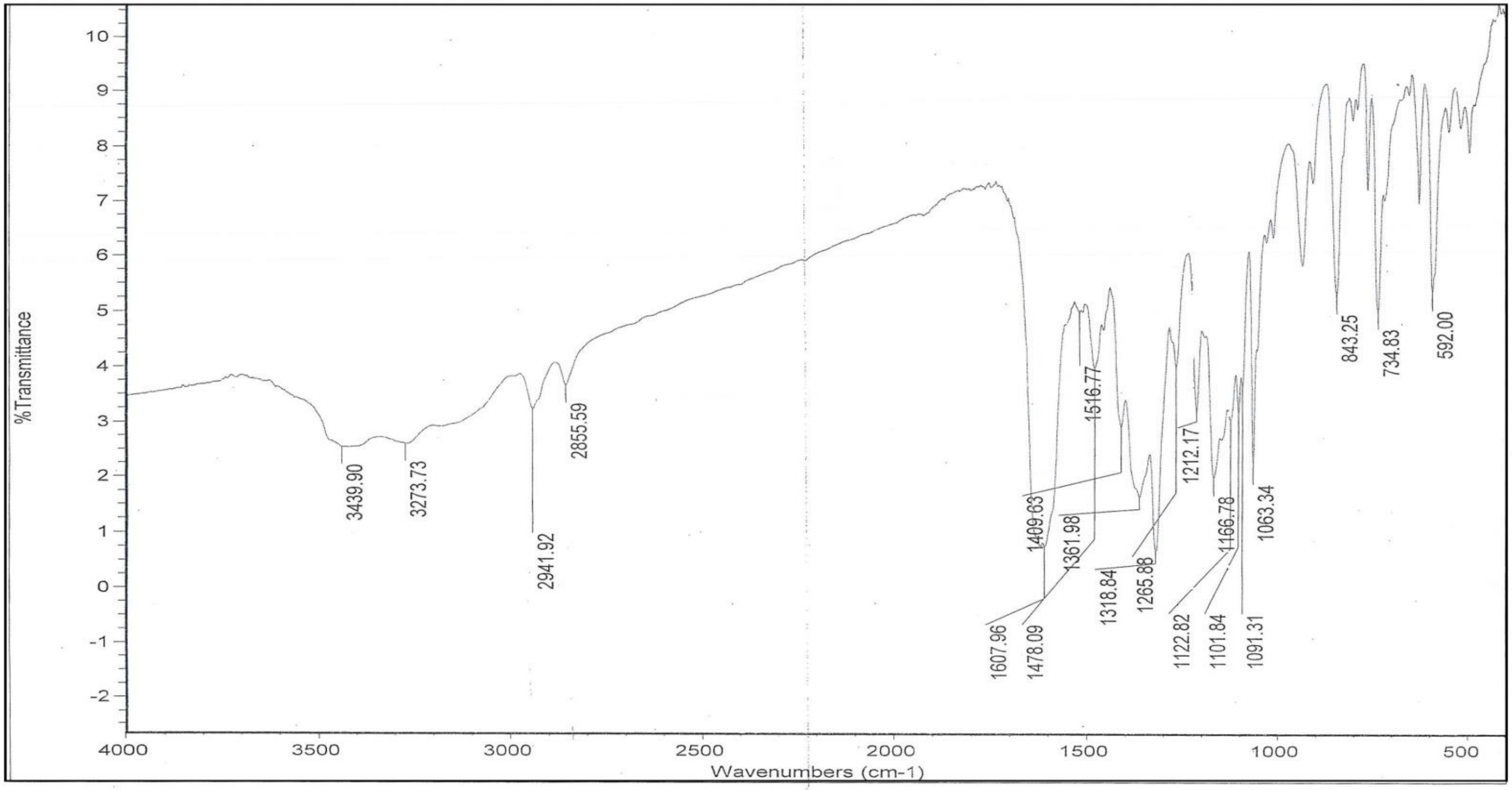

%Transmittance
Wavenumbers (cm-1)
3439.90
3273.73
2941.92
2855.59
1607.96
1478.09
1409.63
1361.98
1516.77
1318.84
1265.88
1212.17
1122.82
1101.84
1166.78
1091.31
1063.34
843.25
734.83
592.00

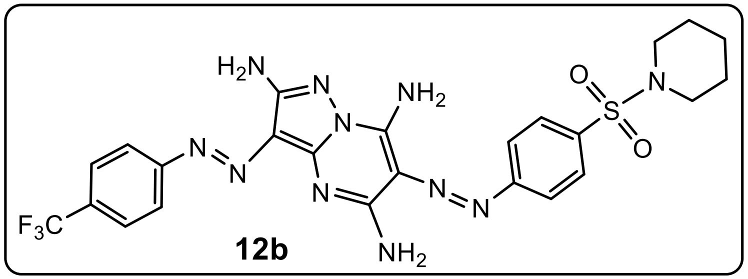

12b

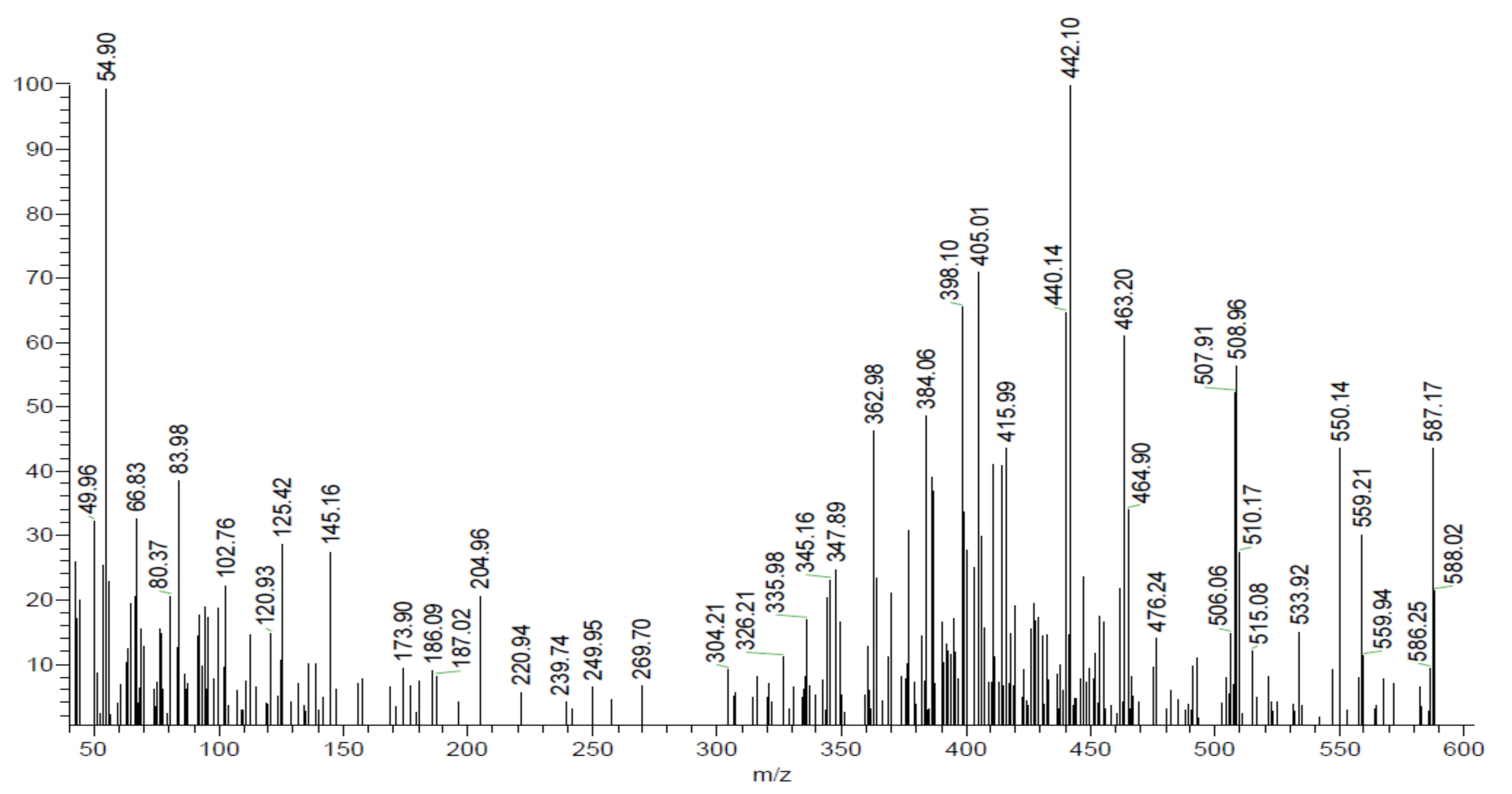
54.90
442.10
405.01
398.10
440.14
463.20
508.96
507.91
362.98
384.06
415.99
550.14
587.17
83.98
66.83
49.96
464.90
559.21
510.17
125.42
145.16
102.76
80.37
347.89
345.16
335.98
588.02
204.96
120.93
326.21
304.21
173.90
186.09
187.02
220.94
239.74
249.95
269.70
476.24
506.06
515.08
533.92
559.94
586.25
m/z

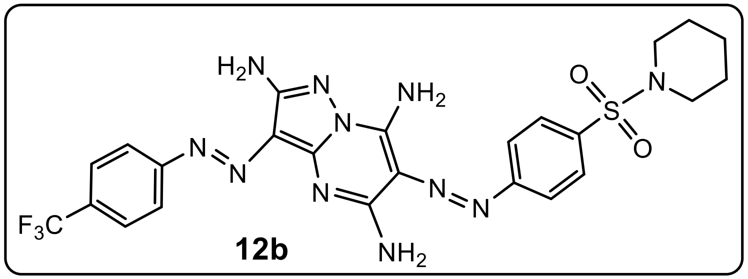

12b

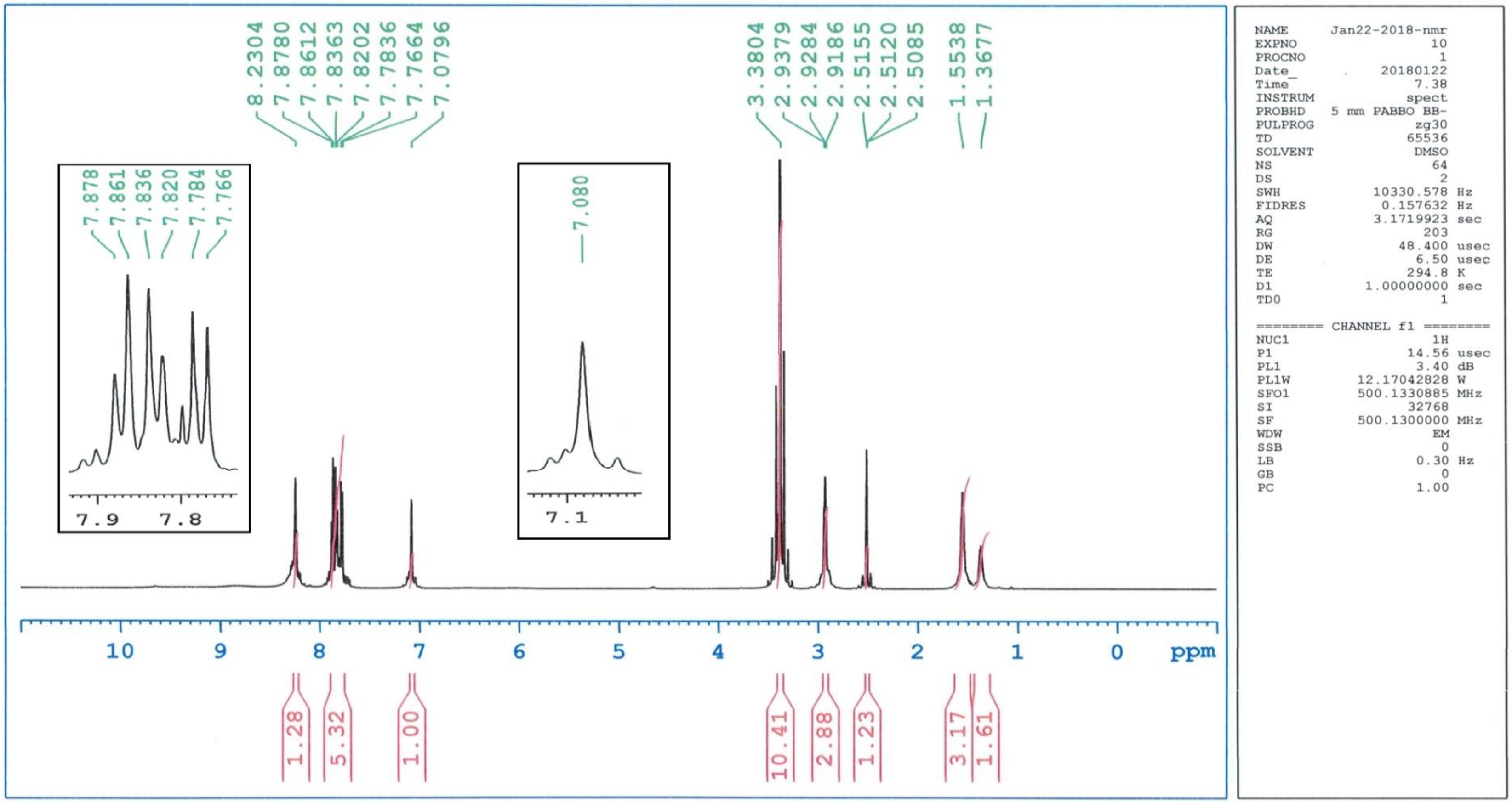
8.2304
7.8780
7.8612
7.8363
7.8202
7.7836
7.7664
7.0796
3.3804
2.9379
2.9284
2.9186
2.5155
2.5120
2.5085
1.5538
1.3677
7.878
7.861
7.836
7.820
7.784
7.766
7.9
7.8
7.080
7.1
10
9
8
7
6
5
4
3
2
1
0
ppm
1.28
5.32
1.00
10.41
2.88
1.23
3.17
1.61
NAME Jan22-2018-nmr
EXPNO 10
PROCNO 1
Date_ 20180122
Time 7.38
INSTRUM spect
PROBHD 5 mm PABBO BB-
PULPROG zg30
TD 65536
SOLVENT DMSO
NS 64
DS 2
SWH 10330.578 Hz
FIDRES 0.157632 Hz
AQ 3.1719923 sec
RG 203
DW 48.400 usec
DE 6.50 usec
TE 294.8 K
D1 1.00000000 sec
TD0 1
======== CHANNEL f1 ========
NUC1 1H
P1 14.56 usec
PL1 3.40 dB
PL1W 12.17042828 W
SFO1 500.1330885 MHz
SI 32768
SF 500.1300000 MHz
WDW EM
SSB 0
LB 0.30 Hz
GB 0
PC 1.00

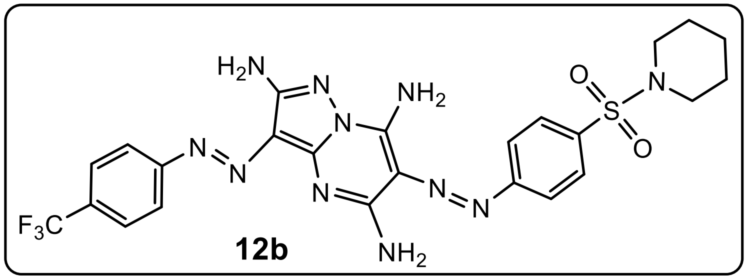

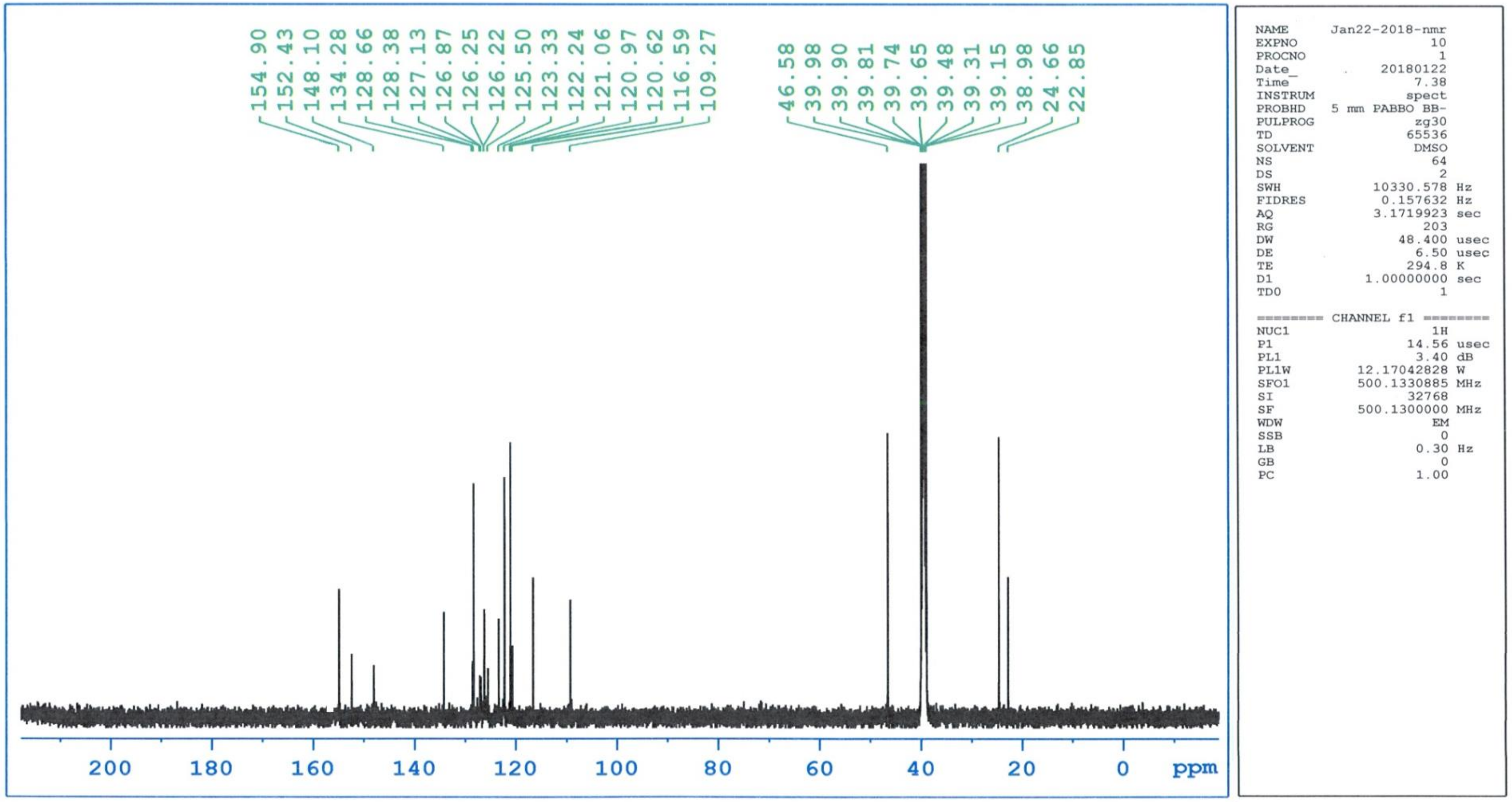


```
NAME       Jan22-2018-nmr
EXPNO                  10
PROCNO                  1
Date_            20180122
Time                 7.38
INSTRUM             spect
PROBHD    5 mm PABBO BB-
PULPROG              zg30
TD                  65536
SOLVENT              DMSO
NS                     64
DS                      2
SWH          10330.578 Hz
FIDRES        0.157632 Hz
AQ          3.1719923 sec
RG                    203
DW           48.400 usec
DE             6.50 usec
TE              294.8 K
D1         1.00000000 sec
TD0                     1

======== CHANNEL f1 ========
NUC1                   1H
P1            14.56 usec
PL1              3.40 dB
PL1W         12.17042828 W
SFO1      500.1330885 MHz
SI                  32768
SF        500.1300000 MHz
WDW                    EM
SSB                     0
LB                0.30 Hz
GB                      0
PC                   1.00
```

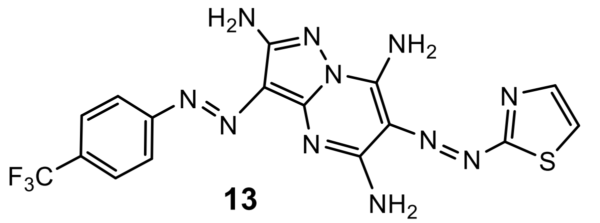
H2N
N
N
NH2
N
N
N
N
N
N
N
S
F3C
NH2

13

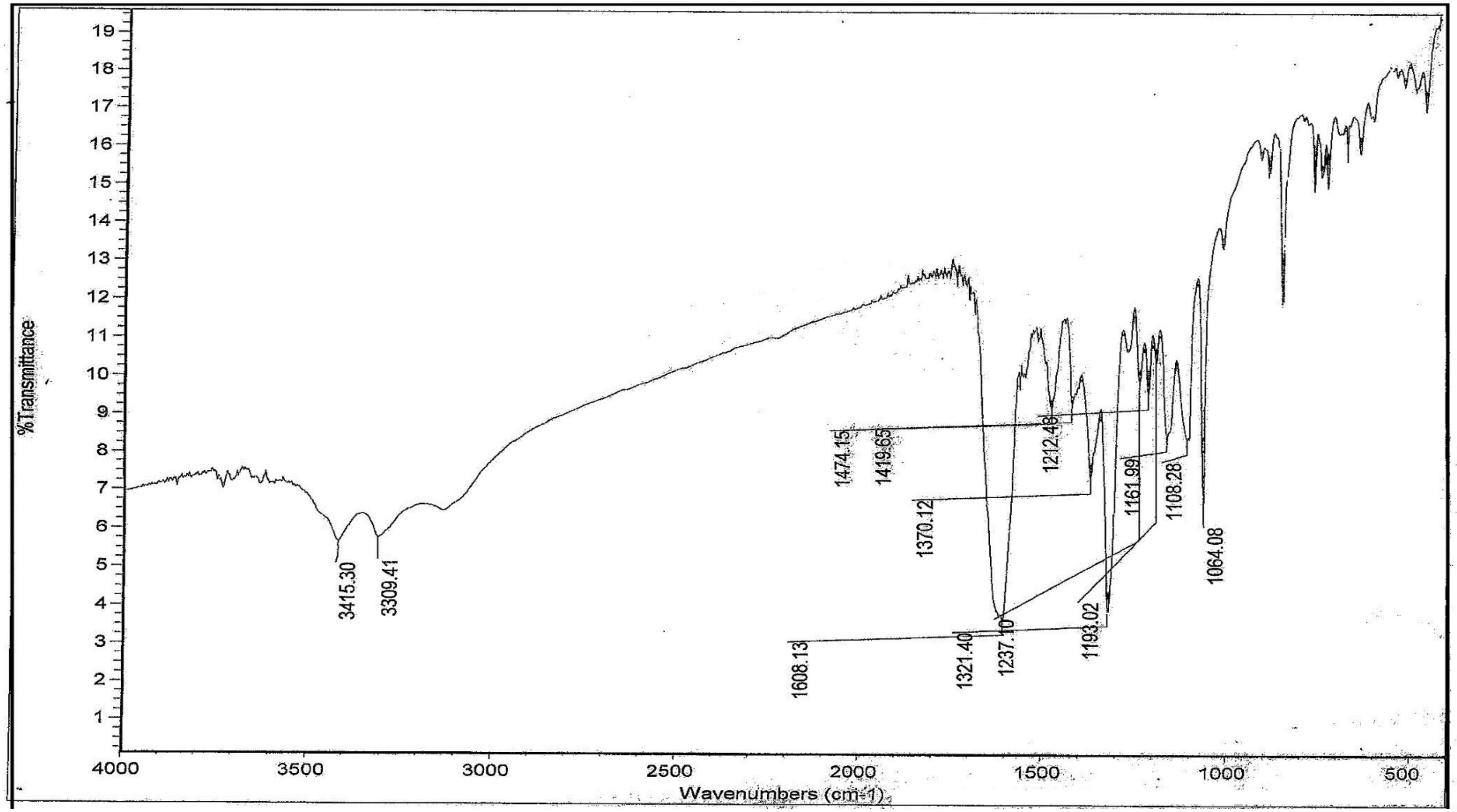
%Transmittance
19
18
17
16
15
14
13
12
11
10
9
8
7
6
5
4
3
2
1
3415.30
3309.41
1474.15
1419.65
1370.12
1608.13
1321.40
1237.10
1212.48
1193.02
1161.99
1108.28
1064.08
4000
3500
3000
2500
2000
1500
1000
500
Wavenumbers (cm-1)

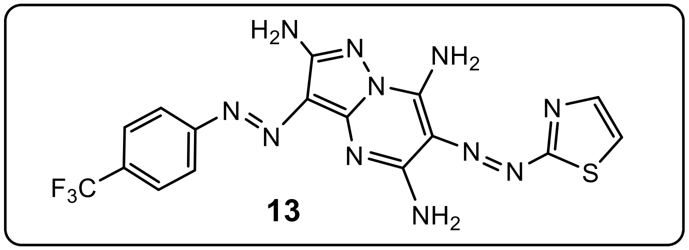

H2N
NH2
NH2
N
S
F3C

13

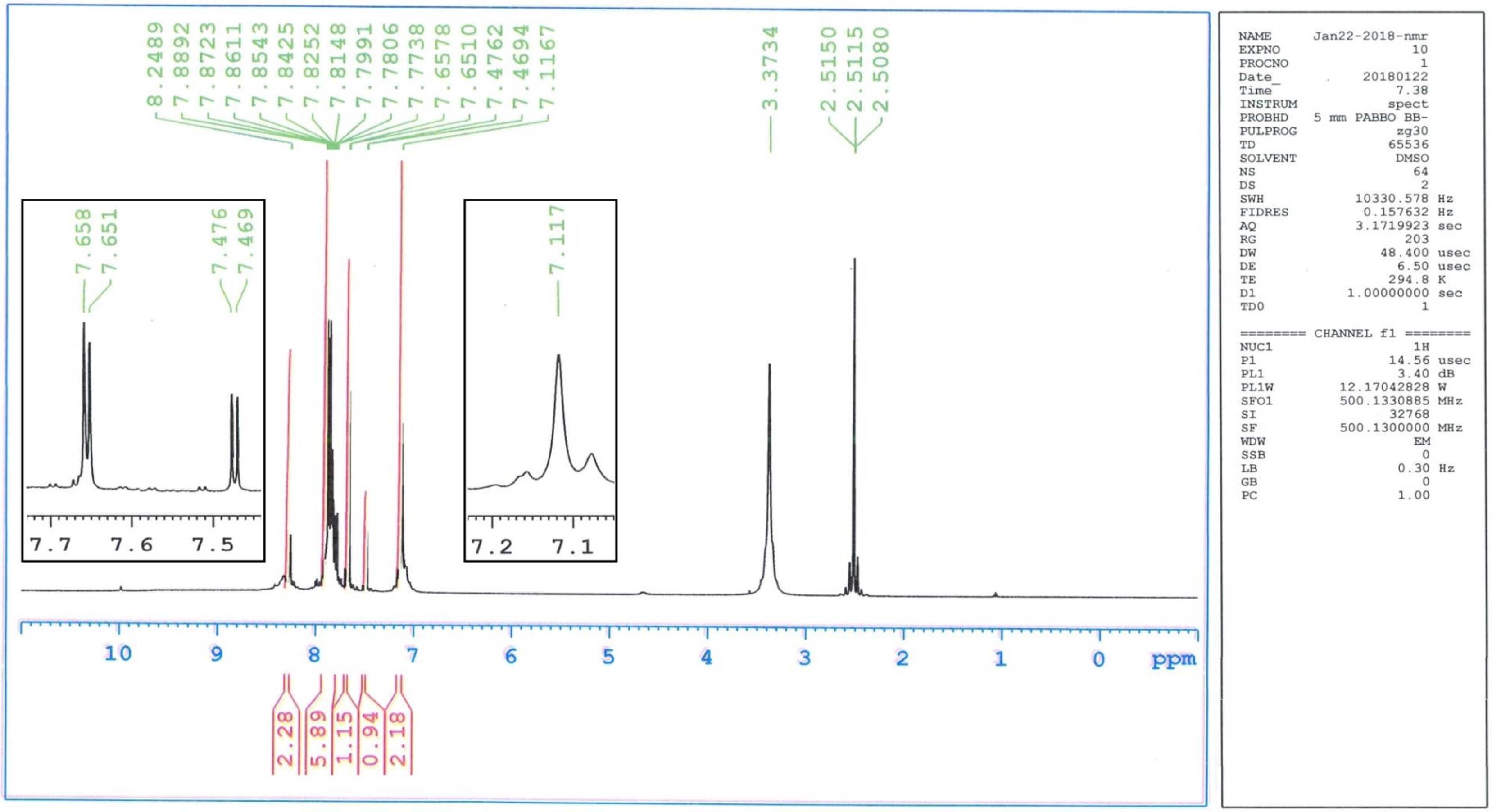

8.2489
7.8892
7.8723
7.8611
7.8543
7.8425
7.8252
7.8148
7.7991
7.7806
7.7738
7.6578
7.6510
7.4762
7.4694
7.1167
3.3734
2.5150
2.5115
2.5080
7.658
7.651
7.476
7.469
7.117
2.28
5.89
1.15
0.94
2.18
ppm
NAME Jan22-2018-nmr
EXPNO 10
PROCNO 1
Date_ 20180122
Time 7.38
INSTRUM spect
PROBHD 5 mm PABBO BB-
PULPROG zg30
TD 65536
SOLVENT DMSO
NS 64
DS 2
SWH 10330.578 Hz
FIDRES 0.157632 Hz
AQ 3.1719923 sec
RG 203
DW 48.400 usec
DE 6.50 usec
TE 294.8 K
D1 1.00000000 sec
TD0 1
======== CHANNEL f1 ========
NUC1 1H
P1 14.56 usec
PL1 3.40 dB
PL1W 12.17042828 W
SFO1 500.1330885 MHz
SI 32768
SF 500.1300000 MHz
WDW EM
SSB 0
LB 0.30 Hz
GB 0
PC 1.00

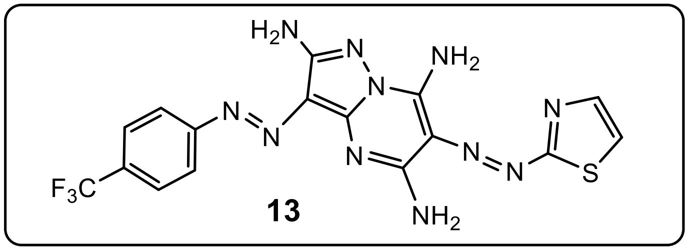

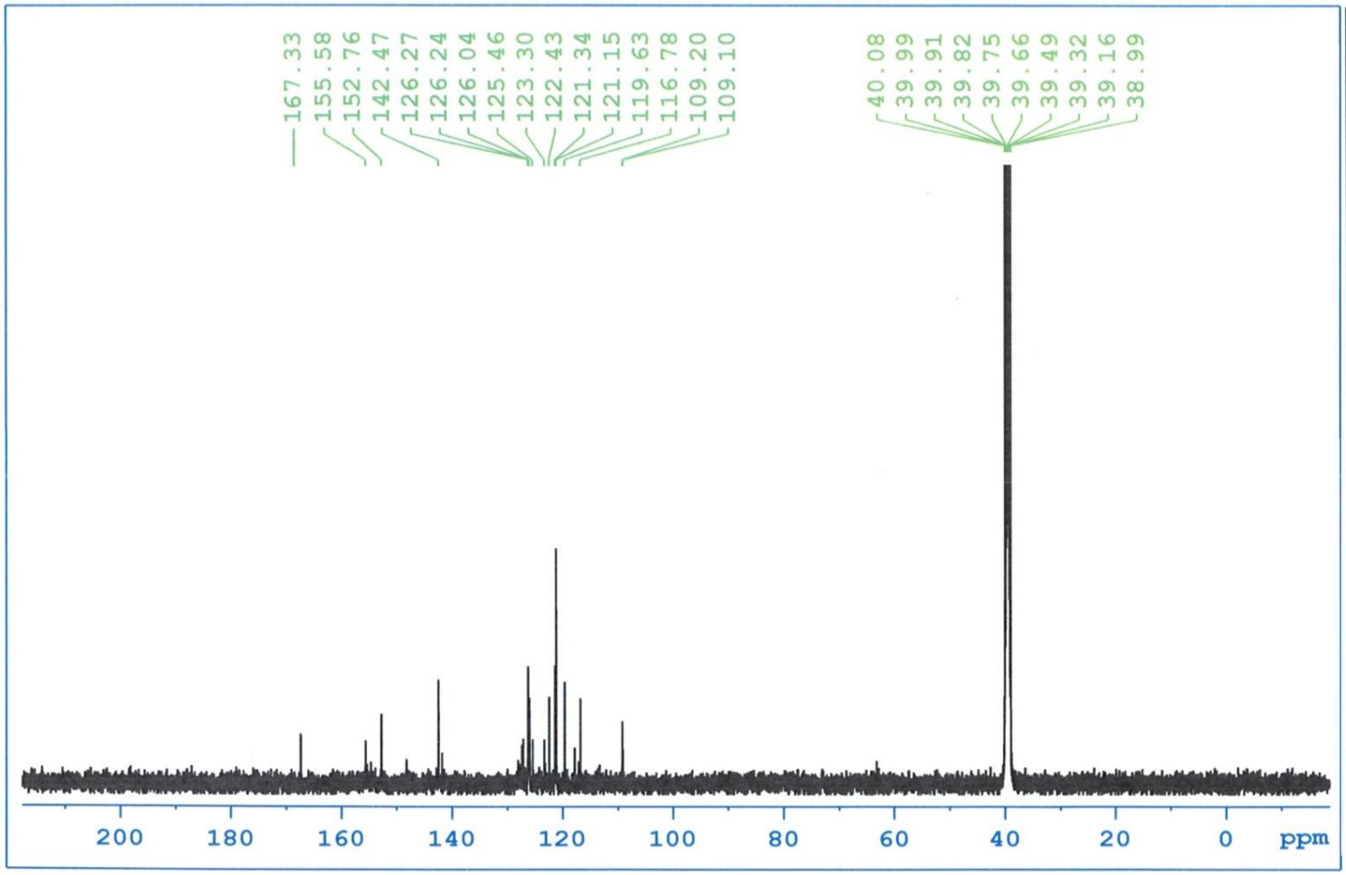

NAME	Jan22-2018-nmr
EXPNO	10
PROCNO	1
Date_	20180122
Time	7.38
INSTRUM	spect
PROBHD	5 mm PABBO BB-
PULPROG	zg30
TD	65536
SOLVENT	DMSO
NS	64
DS	2
SWH	10330.578 Hz
FIDRES	0.157632 Hz
AQ	3.1719923 sec
RG	203
DW	48.400 usec
DE	6.50 usec
TE	294.8 K
D1	1.00000000 sec
TD0	1
======== CHANNEL f1 ========	
NUC1	1H
P1	14.56 usec
PL1	3.40 dB
PL1W	12.17042828 W
SFO1	500.1330885 MHz
SI	32768
SF	500.1300000 MHz
WDW	EM
SSB	0
LB	0.30 Hz
GB	0
PC	1.00
